# Genome-Wide Identification and Expression Analysis of the Ammonium Transporter Family Genes in Soybean

**DOI:** 10.3390/ijms24043991

**Published:** 2023-02-16

**Authors:** Wei Yang, Xiaoxu Dong, Zhanxin Yuan, Yan Zhang, Xia Li, Youning Wang

**Affiliations:** 1 National Key Laboratory of Crop Genetic Improvement, College of Plant Science and Technology, Huazhong Agricultural University, Wuhan 430070, China; 2 Hubei Hongshan Laboratory, College of Plant Science and Technology, Huazhong Agricultural University, Wuhan 430070, China

**Keywords:** soybean, nitrogen, ammonium transporter (AMT), gene expression

## Abstract

Ammonium transporters (AMTs) are responsible for ammonium absorption and utilization in plants. As a high-nitrogen-demand crop and a legume, soybean can also obtain ammonium from symbiotic root nodules in which nitrogen-fixing rhizobia convert atmospheric nitrogen (N_2_) into ammonium. Although increasing evidence implicates vital roles of ammonium transport in soybean, no systematic analyses of AMTs in soybean (named GmAMTs) or functional analyses of GmAMTs are available. In this study, we aimed to identify all GmAMT family genes and gain a better understanding of the characteristics of *GmAMT* genes in soybean. Here, due to the improved genome assembly and annotation of soybean, we tried to generate a phylogenetic tree of 16 *GmAMTs* based on new information. Consistent with reported data, GmAMT family members can be divided into two subfamilies of *GmAMT1* (6 genes) and *GmAMT2* (10 genes). Interestingly, unlike *Arabidopsis*, which has only one *AMT2*, soybean has substantially increased the number of *GmAMT2s*, suggesting enhanced demand for ammonium transport. These genes were distributed on nine chromosomes, of which *GmAMT1.3, GmAMT1.4*, and *GmAMT1.5* were three tandem repeat genes. The gene structures and conserved protein motifs of the GmAMT1 and GmAMT2 subfamilies were different. All the GmAMTs were membrane proteins with varying numbers of transmembrane domains ranging from 4 to 11. Promoter analysis found that these *GmAMT* genes have phytohormone-, circadian control-, and organ expression-related *cis*-elements in their promoters, and notably, there were nodulation-specific and nitrogen-responsive elements in the promoters of the *GmAMT1* and *GmAMT2* genes. Further expression data showed that these GmAMT family genes exhibited different spatiotemporal expression patterns across tissues and organs. In addition, *GmAMT1.1, GmAMT1.2, GmAMT2.2*, and *GmAMT2.3* were responsive to nitrogen treatment, while *GmAMT1.2*, *GmAMT1.3*, *GmAMT1.4*, *GmAMT1.5*, *GmAMT1.6*, *GmAMT2.1*, *GmAMT2.2*, *GmAMT2.3*, *GmAMT3.1*, and *GmAMT4.6* showed circadian rhythms in transcription. RT-qPCR validated the expression patterns of *GmAMTs* in response to different forms of nitrogen and exogenous ABA treatments. Gene expression analysis also confirmed that *GmAMTs* are regulated by key nodulation gene *GmNINa*, indicating a role of *GmAMTs* in symbiosis. Together, these data indicate that GmAMTs may differentially and/or redundantly regulate ammonium transport during plant development and in response to environmental factors. These findings provide a basis for future research on the functions of GmAMTs and the mechanisms through which GmAMTs regulate ammonium metabolism and nodulation in soybean.

## 1. Introduction

Nitrogen is an essential nutrient element for plant growth and development, and an important component of amino acids and nucleic acids. Ammonium and nitrate are two main nitrogen sources for plants in soils. As sessile organisms, plants have evolved a variety of adaptive mechanisms that enable them to respond to their internal nitrogen status and external nitrogen availability [1]. Plants prefer to take up ammonium nitrogen as a nutrient source in the presence of both ammonium and nitrate or in ammonium-rich flooded or acidic soils [2,3,4]. Ammonium is sensed by plants, and a signal transduction cascade is then activated to link nutrient availability with the proper plant response. The ammonium transporter is an important carrier for plants to absorb and utilize ammonium, and can balance the concentrations of ammonium in plants by regulating ammonium uptake from the environments [5] and mediating the transport of ammonium in the organs and tissues of plants [6,7]. The first plant ammonium transporter (AMT) was identified in *Arabidopsis* and could functionally complement a yeast mutant defective in ammonium uptake [8]. The ammonium transporters in plants are mainly divided into two subfamilies: AMT1 and AMT2. AMT2 subfamily members are less homologous to the members of the AMT1 subfamily, but they have high homology with AmtB in *Escherichia coli* and Mep in yeast, belonging to the MEP subfamily [9,10]. Plant AMT proteins are mainly localized on the cell membrane and generally have 11–12 transmembrane domains [11]. According to kinetic experiments, AMTs in plants can be divided into high- and low- affinity ammonium transporters [12,13,14], which tightly and dynamically regulate ammonium uptake in plant growth and development.

The concentrations of ammonium in soils are generally less than 1 mM [3]. Thus, the absorption of ammonium nitrogen by plants occurs mainly through a high-affinity ammonium transport system. In *Arabidopsis*, all six AMTs (5 in the AMT1 subfamily and 1 in the AMT2 subfamily) are high-affinity ammonium transporters [12,15]. Among them, *AtAMT1.1*, *AtAMT1.2*, *AtAMT1.3*, and *AtAMT1.5* are mainly expressed in roots, including root hairs, and their expression is induced by low nitrogen [12,13,16,17]. Loss-of-function mutations in *AMT1.1*, *AMT1.2* and *AMT1.3* reduced the ammonium uptake capacity by 90% in *Arabidopsis* roots, demonstrating that these AMT1s are the main uptake system for plants to obtain ammonium nitrogen from the environment [12]. In contrast, AMT2 is not involved in the acquisition of ammonium nitrogen from soil by *Arabidopsis* plants. Upon ammonium treatment, *AMT2* is mainly expressed in the stele sheath and participates in the transport of ammonium from roots to shoots [6]. *AMT* expression is also detected in leaves, petioles, and stems in various plants, such as tomato (*Solanum lycopersicum* L.) [18], rice (*Oryza sativa* L.) [19], maize (*Zea mays* L.) [20], and poplar (*Populus* L.) [21], and losses of function in some of these genes cause great reductions in aboveground biomass [20,22,23]. It is apparent that AMTs are differentially expressed and participate in ammonium transport therein.

In addition to the spatiotemporal expression and action of these *AMTs*, some *AMT* genes appear to be regulated by the circadian clock. For example, *AtAMT1.3* showed a typical type of diurnal change pattern. The expression of *AtAMT1.3* increased significantly, and the absorption of ammonium peaked at the end of daytime light, while the absorption of ammonium gradually decreased with decreasing light intensity [2]. Rhythm regulation was also observed for *LeAMT1.2* and *LeAMT1.3* in tomato [18]. Nutrient uptake and metabolism are regulated by the biological clock [24]. These particular AMTs may be responsible for rhythmic changes in ammonium uptake and remobilization and assimilation during day and night. Notably, AMTs are also involved in other biological processes, such as plant–microbe interactions. For example, AMT1.1, AMT1.3, and AMT2.3 are involved in plant responses to pathogens in rice and wheat [25,26,27]. Furthermore, there is now increasing evidence that AMTs are involved in plant–microbe symbiosis. It has been reported that *LjAMT2.1* and *LjAMT2.2* in *Lotus japonicus* and *MtAMT2.3* in *Medicago truncatula* may participate in ammonium transport from symbiotic host plants to nitrogen-fixing rhizobia and arbuscular mycorrhizae (AM) [28,29,30]. Therefore, AMTs play essential roles in helping plants acquire sufficient ammonium for plant development and environmental adaptation.

Soybean (*Glycine max*) is the most important crop in the world, with high quality and quantity of plant proteins and oils. Soybeans can interact with rhizobia to form root nodules in which rhizobia convert N_2_ in the air into ammonium to obtain most of the nitrogen for plant growth [31]. They are also able to uptake ammonium and nitrate from soils to meet high nitrogen requirements [32]. However, the understanding of ammonium sensing, transport, and assimilation is limited. GmSAT1 (symbiotic ammonium transporter 1), which was originally identified as an ammonium transporter [33], is actually a membrane-localized basic helix-loop-helix (bHLH) transcription factor that indirectly regulates ammonium transport in nodules [34]. To date, only one study has shown that five *GmAMT* genes (*GmAMT1.4*, *GmAMT3.1*, *GmAMT4.1*, *GmAMT4.3*, and *GmAMT4.4*) were induced by AM in soybean roots, and among them GmAMT4.1 may be involved in the transport of ammonium between AM and host cells because of its specific expression and localization in the branch region of periarbuscular membranes [35]. Although soybean has a more complex regulation of ammonium acquisition and assimilation than staple crops, we know very little about ammonium sensing, uptake, and remobilization, which are the first critical steps in ammonium metabolism. There has been no systematic research on the GmAMT family genes; in particular, to date, none of the *GmAMT* genes have been functionally characterized in soybean.

In this study, we conducted a systematic analysis of ammonium transporter proteins in soybean. In addition to the conserved GmAMTs, soybean also has legume-specific AMTs. We also analyzed the structures of *GmAMT* genes and proteins and provided detailed information on *cis* elements in these *GmAMT* promoters. Furthermore, we presented the spatiotemporal expression patterns of *GmAMT* genes and their expression in response to internal and external factors. Our results provide a theoretical basis for future in-depth studies on the function and regulatory mechanisms by which GmAMTs regulate ammonium uptake and metabolism to enhance the yield and quality of soybean.

## 2. Results

### 2.1. Identification of GmAMT Family Proteins in Soybean

Due to the improved genome assembly and annotation, to identify the AMT family proteins in soybean, we performed a BLAST search for the conserved domain of Ammonium_transp in the Pfam database and identified a total of 16 AMTs (GmAMTs) in the soybean genome, which is consistent with a previous study [35]. However, the amino acid sequences of some of GmAMT family proteins, including GmAMT1.6, GmAMT4.1, GmAMT4.2, GmAMT4.4, GmAMT4.5, and GmAMT4.6, appeared to be different from those in the old version of the JGI *G. max* genome database (Glyma1.0). To gain a better understanding of the evolution of these GmAMTs, we also retrieved 6 AMTs from *Arabidopsis thaliana*, 12 from *Oryza sativa* (rice), 11 from *Medicago truncatula*, and 8 from *Lotus japonicus* (Supplementary Data 1). As shown in Figure S1, the phylogenetic relationship of AMT members in different species appeared to be consistent with that of reported data [35]. Phylogenetic tree analysis revealed that these 53 plant AMTs were divided into two subfamilies: 22 and 31 in the AMT1 and AMT2 subfamilies, respectively. The numbers of AMT1 proteins in these legumes and nonlegume plants did not vary greatly, ranging from three in *Medicago truncatula* and rice to five in *Arabidopsis thaliana* and *Lotus japonicus*, and six in soybean. Interestingly, only a single copy of AMT2 has remained in *Arabidopsis*; however, the numbers of AMT2s have increased substantially from 5 to 10 in the rest of the plant species, with soybean encoding the most (Figure S1). Among these legume and nonlegume plants, soybean has evolved more AMTs, especially GmAMT2s, suggesting that soybean may have evolved a more complex ammonium transport system to meet the needs of extensive intra- and intercellular ammonium transport in plants.

Further analysis revealed that the AMTs in each subfamily can be divided into different clades with varied numbers in different species (Figure S1). There are four clades (I–IV) for AMT1 proteins, and four clades (V–VIII) for AMT2s. Some clades have AMTs from only one species (I from *Medicago truncatula*), while others contain AMTs from multiple plants. Among them, clades IV and VIII have one or several AMT1(s) and AMT2(s) from all five species, respectively. For soybean, six GmAMT1 proteins fell mainly into II (GmAMT1.3, GmAMT1.4, GmAMT1.5, and GmAMT1.6) and IV clusters (GmAMT1.1 and GmAMT1.2), while 10 GmAMT2s fell into the clades V (GmAMT4.4), VI (GmAMT4.1, GmAMT4.2, GmAMT4.3, GmAMT4.5, and GmAMT4.6), VII (GmAMT3.1), and VIII (GmAMT2.1 to GmAMT2.3). It is worth noting that clade V contains only AMT2s from legumes, suggesting that these AMT2s may have divergent structures and functions.

### 2.2. Characteristics of GmAMT Proteins

As these GmAMTs belong to different subfamilies and clades, we hypothesized that these proteins may have varied protein sequences and structures. Indeed, the lengths of these GmAMT proteins varied from 218 aa (GmAMT1.6) to 525 aa (GmAMT1.5), with the molecular weights from 23.315 kDa to 56.108 kDa. These GmAMTs also had different predicted protein isoelectric points (PIs). The PI values of GmAMT1.4, GmAMT1.6, GmAMT2.2, and GmAMT2.3 were greater than 8; the PI values of GmAMT4.1, GmAMT4.5 and GmAMT4.6 were less than 6; and the PI values of other AMT proteins were between 6.25 and 7.71 (Table S1).

**Figure 1:**
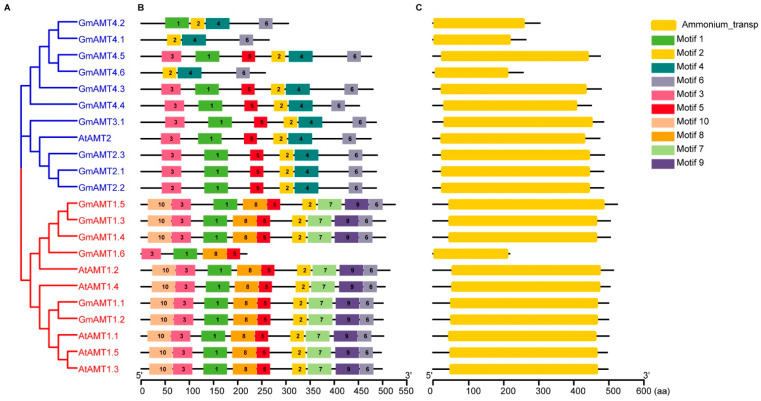
Conserved protein motifs and conserved domain analysis of AMTs in soybean and *Arabidopsis*. (**A**) An unrooted phylogenetic tree was constructed by MEGA X software using the maximum-likelihood method. The blue and red branches represent the AMT2 and AMT1 subfamilies, respectively. (**B**) Conserved motif analysis of AMT amino acid sequences by the MEME online website. Different colors represent different motifs. (**C**) Conserved domain analysis of AMT proteins by the NCBI CD-search tool.

We further analyzed the structures of GmAMT proteins, including the conserved motifs and conserved domain (Ammonium_transp) of GmAMTs according to the evolutionary relationships (Figure 1). First, we found a total of 10 conserved motifs of AMTs in these GmAMTs (Figure 1A,B and Figure S2). Among them, motifs 1, 2, 3, 4, 5, 7, 8, and 9 belong to the ammonium_transp domain, and motifs 10 and 6 belong to the N- and C-terminal conserved motifs of AMT proteins (Figure S3). Motifs 1, 2, 3, 5, and 6 are common to most AMT proteins, indicating that these motifs may be the characteristic motifs of ammonium transporters. Motifs 7, 8, 9, and 10 are unique to the AMT1 subfamily, while motif 4 is unique to the AMT2 subfamily proteins (Figure 1A,B).

We then performed a conservative domain analysis. All 16 GmAMTs contain the conserved Ammonium_transp domain, but GmAMT1.6 in the AMT1 subfamily and GmAMT4.1, GmAMT4.2, and GmAMT4.6 in the AMT2 subfamily do not have the complete Ammonium_transp domain (Figure 1C). The Ammonium_transp domain is essential for ammonium transport activity [36], and the lack of the critical Ammonium_transp domain in GmAMT1.6, GmAMT4.1, GmAMT4.2, and GmAMT4.6 suggests that they may have altered functions in soybean. Next, we analyzed the transmembrane domains, which are essential for the membrane localization and transporter activity. We found that all GmAMTs contain conserved transmembrane domains (Figure S4). Among them, GmAMT2.1, GmAMT2.2, GmAMT2.3, GmAMT3.1, and GmAMT4.5 have 11 transmembrane domains; GmAMT1.5, GmAMT4.3, and GmAMT4.4 have 10 transmembrane domains; GmAMT1.1, GmAMT1.2, GmAMT1.3, and GmAMT1.4 have 9 transmembrane domains; GmAMT4.2 has 8 transmembrane domains; GmAMT1.6 and GmAMT4.1 have 5 transmembrane domains; and GmAMT4.6 has only 4 transmembrane domains. These GmAMT proteins have variable N- and C-termini. Subcellular localization prediction analysis revealed that the majority of GmAMTs (10/16) were mainly located on the plasma membrane, while the remaining 6 may be localized on the membranes of chloroplasts (GmAMT1.1 and GmAMT1.6), cytoplasm (GmAMT1.2, GmAMT4.5, and GmAMT4.6), and vacuoles (GmAMT4.1) (Table S1). The differences in physicochemical properties and subcellular localizations indicate that these GmAMTs may be involved in ammonium transport at different sites in the cell.

### 2.3. Chromosome Distribution and Gene Replication of GmAMT Family Genes

**Figure 2:**
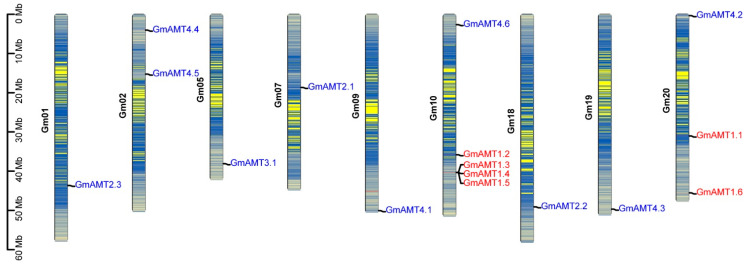
Chromosomal distribution analysis of *GmAMT* genes. The genome visualization tool of TBtools was used to map the chromosomal distribution of *GmAMT* genes. The colors of the bars indicate soybean chromosomes, and the color gradients in chromosomes represent gene density. Gene names were marked on each chromosome. GmAMT1 and GmAMT2 subfamily genes are highlighted with red and blue colors, respectively. The scale bar is presented on the left side.

Next, we analyzed the distribution of 16 *GmAMT* genes on chromosomes and found that they were distributed on only nine chromosomes (Chr), including Chr1, Chr2, Chr5, Chr7, Chr9, Chr10, Chr18, Chr19, and Chr20 (Figure 2). GmAMT1 subfamily genes were distributed on chromosomes 10 and 20. Of the four *GmAMT1* genes on chromosome 10, *GmAMT1.3*, *GmAMT1.4*, and *GmAMT1.5* are tandem repeat genes. Ten *GmAMT2* subfamily genes were evenly distributed on nine chromosomes and some of them were positioned at the ends of chromosomes. Different chromosomal distribution patterns of *GmAMT* genes may be related to their functions.

**Figure 3:**
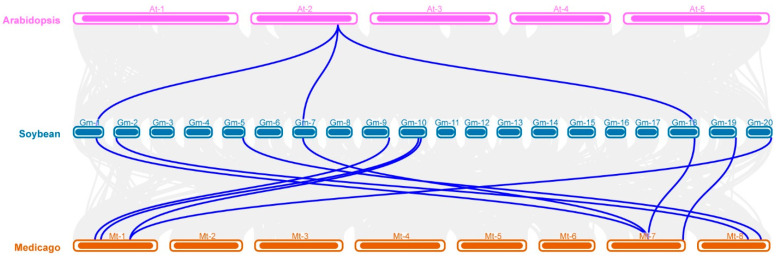
Synteny analysis of *AMT* genes among *Arabidopsis*, soybean and Medicago. One Step MCScanX on TBtools was used to analyse gene duplication. Grey lines in the background indicate the collinear blocks within the genomes of soybean, *Arabidopsis* and Medicago. The blue lines indicate the syntenic *AMT* gene pairs.

Because *Arabidopsis* has only one AMT2, while soybean has evolved 10 *GmAMT2* genes, we attempted to decipher the co-evolution relationship of these AMT2s through a collinearity analysis of *AMTs* in *Arabidopsis* and soybean (Figure 3 and Figure S5). The results showed that *GmAMT2.1*, *GmAMT2.2*, and *GmAMT2.3* in soybean were orthologous to *AtAMT2* in *Arabidopsis*, indicating that *GmAMT2.1*, *GmAMT2.2*, and *GmAMT2.3* may retain the same function as *AtAMT2*, which encodes a high-affinity plasma membrane ammonium transporter [15]. Synteny analysis also showed that *GmAMT2.1, GmAMT2.2*, and *GmAMT2.3* were orthologous to one gene in Medicago (*Medtr7g069640*) and two genes in Rice (*LOC_Os11g01410* and *LOC_Os12g01420*) and Lotus (*Lj1g0012871* and *Lj3g0009637*), respectively (Figure 3 and Figure S5). Three paralogous genes, *GmAMT2.1*, *GmAMT2.2* and *GmAMT2.3*, were distributed on Chr1, Chr7, and Chr18. The soybean genome experienced two whole-genome duplications [37]. Thus, it is likely that these paralogous genes evolved during whole-genome duplications and differentially and/or redundantly modulate ammonium transport across the plasma membrane in soybean. Notably, the remaining seven *GmAMT2* genes were distributed on six chromosomes, and there was only one gene on Chr5 (*GmAMT3.1*, *Glyma.05G196500*), Chr9 (*GmAMT4.1*, *Glyma.09G281600*), Chr10 (*GmAMT4.6*, *Glyma.10G030800*), Chr19 (*GmAMT4.3*, *Glyma.19G244400*), and Chr20 (*GmAMT4.2*, *Glyma.20G004100*), except for Chr2 with 2 genes (*GmAMT4.4*, *Glyma.02G043700* and *GmAMT4.5*, and *Glyma.02G143600*) (Figure 2). Together with the fact that these *GmAMT2* genes encode the new GmAMT2 proteins belonging to special clades VII and VIII (Figure S1), these data suggest that soybean may have a more complex ammonium transport system.

To reveal the evolutionary history of GmAMT family genes, we analyzed the selection types of duplicate gene pairs in GmAMT family genes using the Ka/Ks ratio (the ratio of nonsynonymous to synonymous substitutions). Ka/Ks < 1 and > 1 indicate that gene pairs have experienced negative and positive selection, respectively, while Ka/Ks = 1 indicates neutral selection of the genes [38]. We found that the Ka/Ks ratios of most gene pairs were less than 1, suggesting that the majority of *GmAMT* genes have experienced negative selection. Only one gene pair, *GmAMT4.2* and *GmAMT1.1*, had a Ka/Ks ratio greater than 1 (Table S2), indicating that *GmAMT4.2* and *GmAMT1.1* may undergo positive selection and are important for the evolution of soybean.

### 2.4. Gene Structure Analysis of GmAMT Genes

**Figure 4:**
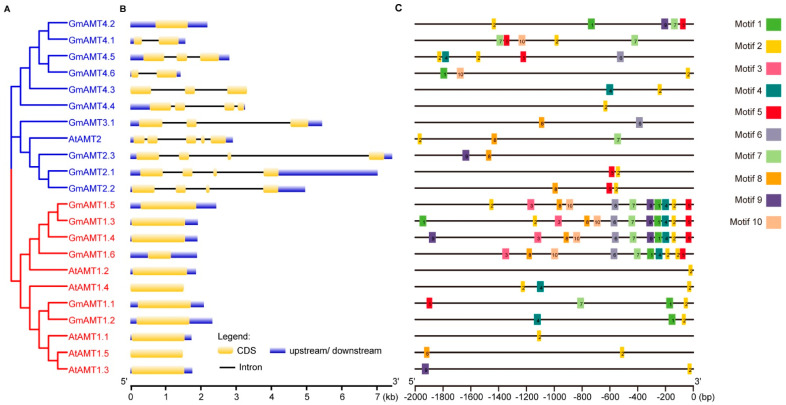
Gene structures and promoter-conserved motif analysis of the AMT family in soybean and *Arabidopsis*. (**A**) Phylogenetic tree analysis of *AMTs* in *Arabidopsis* and soybean. Maximum-likelihood trees were constructed by MEGA-X software with 1000-fold bootstrap resampling. The red and blue branches represent the AMT1 and AMT2 subfamilies, respectively. (**B**) Gene structure analysis of *AMT* genes by the GSDS 2.0 online website. The yellow boxes represent CDS regions, blue boxes represent UTR regions, and black lines represent intron regions. (**C**) The conserved motifs of *AMT* promoters in soybean and *Arabidopsis* were analyzed by the MEME website. Different colors represent different conserved motifs. The 2 kb promoters of *AMT* genes in soybean and *Arabidopsis* were obtained from the Phytozome database.

To better understand the structural changes in *GmAMT1s* and *GmAMT2s* during evolution, we reanalyzed the gene structures of these *GmAMTs*. Interestingly, the exon–intron structures of *GmAMT1.6*, *GmAMT4.1*, *GmAMT4.2*, *GmAMT4.4*, *GmAMT4.5*, and *GmAMT4.6* are different from those in a previous study [35]. The results showed that GmAMT1 and GmAMT2 subfamily genes differ greatly in their gene structures (Figure 4A,B, Supplementary Data 2 and 3). The biggest difference is that all GmAMT1 subfamily genes have no introns, which is consistent with the *AtAMT1* genes in *Arabidopsis*. In addition, all six *GmAMT1s* have untranslated regions (UTRs), which is different from some *Arabidopsis AtAMT1s* (*AtAMT1.4* and *AtAMT1.5*) that lack UTRs. In sharp contrast, among *GmAMT2* genes, all the genes contain introns, exons, and UTR regions, with the exception of *GmAMT4.2*, which lacks introns, and *GmAMT4.3*, which lacks UTRs. Notably, unlike the coding region of *AtAMT2*, which contains four introns and five exons, GmAMT2 subfamily genes have varied numbers of introns and exons ranging from zero to three. Among them, *GmAMT2.1*, *GmAMT2.2*, *GmAMT2.3*, and *GmAMT4.4* have three introns; *GmAMT3.1, GmAMT4.3*, and *GmAMT4.5* have two; *GmAMT4.1* and *GmAMT4.6* have only one; and *GmAMT4.2* has no intron. Furthermore, the lengths of UTRs were also somewhat different among these *AMT2* genes. These structural differences resulted in different lengths (657–1578 bp) of the coding sequences of these genes (Table S1). Together, these results indicate that each AMT subfamily gene has relatively conserved gene structures, and these *AMT* genes with different introns and UTRs are likely to be regulated at posttranscriptional levels.

The promoter is an essential component of one gene where transcription is initiated and regulated. Therefore, we analyzed the promoters of the *GmAMT1* and *GmAMT2* genes. We obtained the 2 kb promoters of all *AMT* genes in soybean and *Arabidopsis* and analyzed the conserved motifs of the promoters. The results showed that the promoter sequences of all these GmAMT family genes were highly different (Figure 4C and Figure S6, Supplementary Data 4). Among the 10 motifs identified from 22 *AMT* genes, motif 2 was very conserved and existed on 20 *GmAMT* promoter sequences except those of *GmAMT2.3* and *GmAMT3.1*. The results indicate that motif 2 is likely an important regulatory element for the majority of *GmAMTs* that mediate the conserved biological processes. Interestingly, the same motifs (Motifs 1–10) were found in the promoters of *GmAMT1.3*, *GmAMT1.4*, and *GmAMT1.5*, which is consistent with the close evolutionary relationship of these three genes. In addition, the motifs on the promoter of *GmAMT1.6* were almost identical to those of *GmAMT1.3*, *GmAMT.4*, and *GmAMT1.5* except for motif 9, suggesting that *GmAMT1.6* may have similar expression patterns to *GmAMT1.3*, *GmAMT1.4*, and *GmAMT1.5* and participate in similar biological processes. In contrast, different motifs were identified on other *GmAMT* promoter sequences, indicating that these genes might have different expression patterns and divergent functions.

### 2.5. Promoter Cis-Element Analysis of GmAMTs

**Figure 5:**
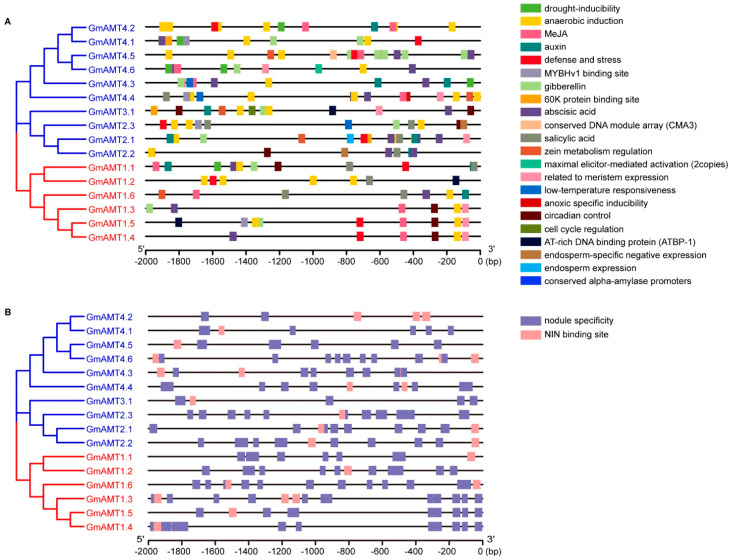
*Cis*-element analysis of *GmAMT* promoters. Sequences 2 kb upstream of the transcription initiation site were downloaded from Phytozome and used to analyze the *cis*-elements. (**A**) The *cis*-acting elements of 16 *AMT* promoters in soybean were analyzed by PlantCARE website. (**B**) *Cis*-acting element analysis of nodule specificity and NIN-binding sites in *GmAMT* promoters. *Cis*-acting elements with similar regulatory functions are indicated by the same color.

Promoters contain various short *cis*-acting regulatory elements necessary to assemble the transcriptional machinery and to regulate expression levels and functions. To explore the expression features and potential functions of *GmAMT* genes, we analyzed *cis*-acting elements in the promoters of *GmAMT* genes. The comprehensive data showed that *GmAMT* promoters have various *cis*-elements that respond to growth hormones (mainly abscisic acid, gibberellin, auxin, jasmonic acid, and salicylic acid), endogenous cues related to plant growth and development (circadian control, zeatin metabolism, conserved sequences in alpha-amylase promoters, and meristem and endosperm expression), and environmental stresses (e.g., light response elements, defense and stress, anaerobic induction, drought stress, and low temperature stress-related elements) (Figure 5A, Table S3). Notably, all *GmAMT* promoters have *cis* elements responsive to light and anaerobic conditions, suggesting an essential role of these *GmAMT* genes in plant adaptation to these conditions in soybean. In addition, we noticed that each gene promoter contains response element(s) to phytohormone(s) with varied numbers ranging from 1 to 5, indicating that these *GmAMT* genes are under the regulation of hormone(s) and are involved in hormone-mediated biological processes.

In addition, we found that 16 genes had different combinations of *cis* elements in their promoters (Figure 5A, Table S3). For example, 7 of 16 *GmAMT* genes (*GmAMT1.1*, *GmAMT1.3*, *GmAMT1.4*, *GmAMT1.5*, *GmAMT2.2*, *GmAMT2.3*, and *GmAMT3.1*) contain circadian rhythm-related *cis*-elements in their promoters, 5 of them (*GmAMT1.6*, *GmAMT2.1*, *GmAMT3.1*, *GmAMT4.4*, and *GmAMT4.5*) contain zeatin metabolism-related elements, and 7 genes (*GmAMT1.3*, *GmAMT1.4*, *GmAMT1.5*, *GmAMT2.1*. *GmAMT3.1*, *GmAMT4.4*, and *GmAMT4.6*) have *cis*-elements related to meristem expression in their promoters. Furthermore, we found that the promoters of eight genes (*GmAMT1.2*, *GmAMT1.4*, *GmAMT2.1*, *GmAMT2.2*, *GmAMT2.3*, *GmAMT4.1*, *GmAMT4.2*, and *GmAMT4.5*) have *cis*-elements related to plant defense, while eight genes share the *cis*-elements related to plant response to drought (*GmAMT1.1*, *GmAMT4.1*, *GmAMT4.2*, *GmAMT4.3*, and *GmAMT4.6*) and low-temperature stress (*GmAMT2.3*, *GmAMT4.3* and *GmAMT4.4)* in their promoters, respectively.

Since GmAMTs are mainly responsible for ammonium sensing and metabolism, we speculated that some *GmAMT* genes are involved in the nitrogen response and symbiotic nitrogen fixation. Therefore, we analyzed the *cis*-elements related to plant response to nitrogen and symbiotic nodulation. As expected, all *GmAMT* promoters contain nodule specificity elements (AAAGAT and CTCTT) [39] and NIN (Nodule Inception) binding site (NBS) *cis* elements for NINs and NLPs (NIN-like proteins) [40,41,42,43] (Figure 5B), suggesting crucial roles of these *GmAMT* genes in nodulation and nitrogen response. Taken together, these results suggest that *GmAMT* genes are likely to show different spatial and temporal expression patterns during development and in response to the environmental conditions.

### 2.6. Expression of GmAMT Family Genes during Soybean Development

**Figure 6:**
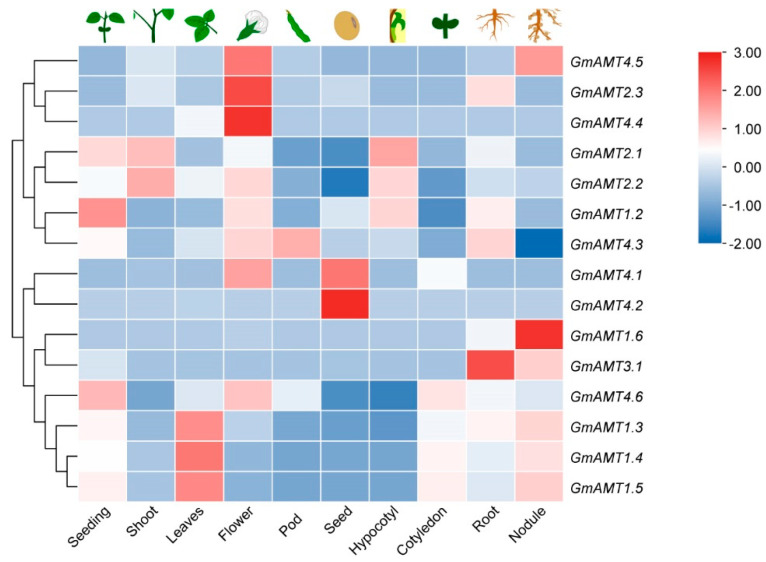
Heatmap of *GmAMT* expression in different tissues using RNA-seq data. The RNA-seq data were downloaded from the Soybean Expression Atlas website [44]. The color scale represents the normalized TPM value.

To assess the possible roles of *GmAMTs* in soybean development, we carried out spatial and temporal expression analyses during soybean growth and development. The expression of these genes was evaluated using RNA-seq data from the Soybean Expression Atlas [44]. As shown in Figure 6, tissue-specific expression patterns were observed in some of *GmAMT* genes. Interestingly, *GmAMT4.2* and *GmAMT4.4* were specifically expressed in seeds and flowers, respectively, while *GmAMT4.1* was expressed at higher levels in both flowers and seeds. Furthermore, we found that some GmAMT family genes were highly expressed in underground tissues of soybean plants. Among them, *GmAMT3.1* was mainly expressed in roots and nodules, while *GmAMT1.6* was specifically expressed in nodules. In contrast, other *GmAMT* genes were mainly expressed in aboveground tissues. For example, *GmAMT2.1* and *GmAMT2.2* were highly expressed in shoot tissues, while *GmAMT1.3*, *GmAMT1.4*, and *GmAMT1.5* were mainly expressed in leaves. The different spatial and temporal expression patterns of soybean GmAMT family genes suggest the *GmAMT* genes differentially and collaboratively mediate ammonium transport in soybean development.

### 2.7. Diurnal Changes of GmAMT Gene Expression

**Figure 7:**
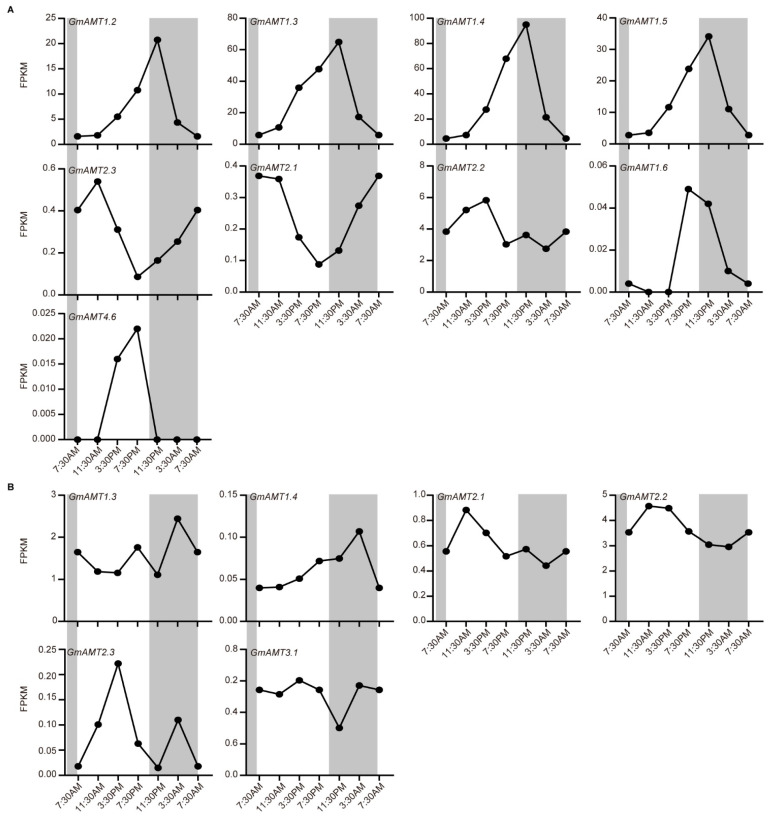
Day–night variations in gene expression of GmAMT family genes by RNA-seq data. The FPKM data of *GmAMT* genes were collected from the JGI Plant Gene Atlas [45]. The day–night expression patterns of *GmAMTs* in leaves (**A**) and nodules (**B**). Dark periods are highlighted as shaded columns.

The circadian clock generates transcriptional oscillations of many genes and regulates a wide array of metabolic processes and plant growth. As there are circadian control elements on the promoters of some *GmAMT* genes, we hypothesized that these genes are under the control of the diurnal change to regulate ammonium transport and metabolism during the day and night. To prove this possibility, we used RNA-seq data from the JGI Plant Gene Atlas [45] to analyze the day–night expression levels of GmAMT family genes in leaves and nodules (Figure 7 and Figure S7). In leaves, the expression of most GmAMT1 subfamily genes (*GmAMT1.2*, *GmAMT1.3*, *GmAMT1.4*, *GmAMT1.5*, and *GmAMT1.6*) and four genes in the GmAMT2 subfamily (*GmAMT2.1*, *GmAMT2.2*, *GmAMT2.3*, and *GmAMT4.6*) showed a typical diurnal variation. Interestingly, these *GmAMT* genes in two subfamilies showed opposite expression trends in day and night. The expression levels of *GmAMT1.2*, *GmAMT1.3*, *GmAMT1.4*, *GmAMT1.5*, and *GmAMT1.6* increased gradually in the daytime, reached the highest levels at night, and then gradually decreased back to the lowest levels before dawn. In contrast, the expression levels of *GmAMT2.1*, *GmAMT2.2*, *GmAMT2.3*, and *GmAMT4.6* decreased gradually during the daytime and increased gradually at night (Figure 7A). In nodules, the expression of the *GmAMT* genes appeared to be less regulated by the diurnal change, but *GmAMT1 and* GmAMT2 subfamily genes also showed opposite patterns during day and night. Among the *GmAMT1* genes, the transcription levels of *GmAMT1.3* and *GmAMT1.4* were lower in the daytime but higher at night, whereas the expression levels of *GmAMT2.1*, *GmAMT2.2*, *GmAMT2.3*, and *GmAMT3.1* were higher in the daytime but lower at night (Figure 7B). Diurnal control of *GmAMT* gene expression suggests that these genes might be responsible for rhythmic changes in ammonium transport and nitrogen metabolism in leaves and nodules of soybean.

### 2.8. Expression of GmAMT Genes under Different Nitrogen Treatments

**Figure 8:**
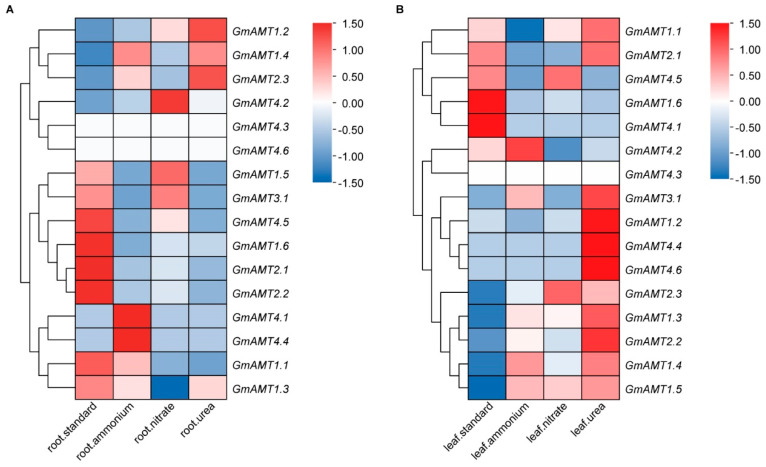
Expression patterns of *GmAMTs* in response to different forms of nitrogen treatment based on RNA-seq data. The *GmAMT* data were collected from the JGI Plant Gene Atlas [45] and shown by heatmaps with clustering created based on the normalized FPKM values of *GmAMT* genes in roots (**A**) and leaves (**B**). The white color represents NA (not available).

Soybeans can take up both ammonium and nitrate in soils to meet their nitrogen requirements. We assumed that the *GmAMT* genes are affected by both ammonium and nitrate. For this, we first analyzed the RNA-seq data from the JGI Plant Gene Atlas to determine how the GmAMT family genes respond to different types of nitrogen in roots and leaves. The GmAMT family genes showed different expression patterns in the roots and leaves of soybean plants treated with 10 mM concentrations of different nitrogen sources for 28 days (Figure 8). In roots, the expression levels of *GmAMT1.2*, *GmAMT1.4*, *GmAMT2.3*, and *GmAMT4.2* were greatly upregulated by ammonium, nitrate, and urea, whereas the expression levels of *GmAMT1.1*, *GmAMT1.3*, *GmAMT1.6*, *GmAMT2.1*, *GmAMT2.2*, and *GmAMT4.5* were downregulated by these nitrogen fertilizers. The expression levels of *GmAMT4.1* and *GmAMT4.4* were significantly upregulated under ammonium treatment (Figure 8A). In leaves, *GmAMT1.3*, *GmAMT1.4*, *GmAMT1.5*, *GmAMT2.2*, and *GmAMT2.3* were upregulated under ammonium, nitrate, and urea conditions, while the expression levels of *GmAMT1.6* and *GmAMT4.1* were downregulated by these nitrogen fertilizers. In addition, some *GmAMT* genes displayed particular expression patterns in response to different forms of nitrogen. Notably, *GmAMT1.2*, *GmAMT4.4*, and *GmAMT4.6* were specifically induced by urea, while *GmAMT4.2* was induced by ammonium and suppressed by nitrate and urea (Figure 8B). It is apparently visible that *GmAMTs* showed different gene expression responses to different forms of nitrogen in the roots and leaves. These results suggest that the *GmAMT* genes may redundantly and coordinately control the absorption and utilization of nitrogen in soybean.

### 2.9. Expression Verification of GmAMTs in Development and Response to Low Nitrogen

**Figure 9:**
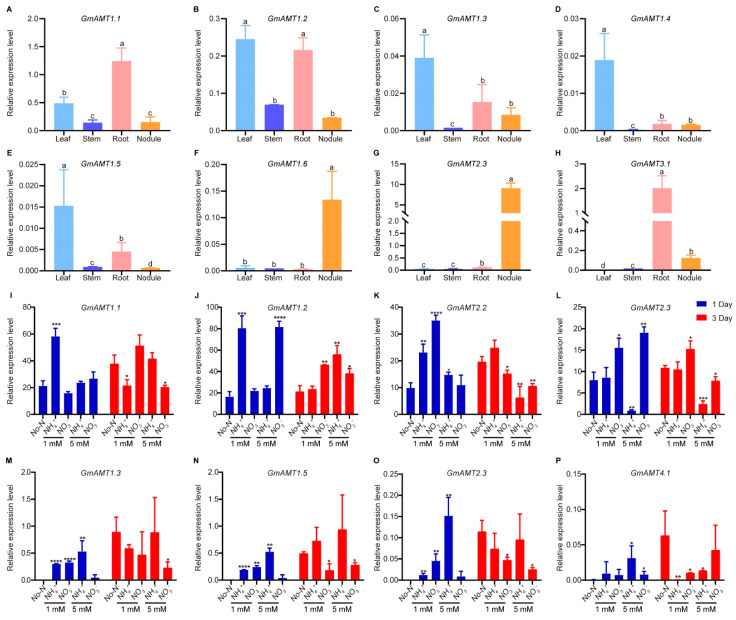
Gene expression analysis of *GmAMTs* in different tissues and in response to different nitrogen treatments by RT-qPCR. (**A**–**H**) Detection of *GmAMT* expression in leaves, stems, roots, and nodules at 24 days after rhizobial inoculation. The expression patterns of *GmAMTs* were analyzed in roots (**I**–**L**) and leaves (**M**–**P**) under different nitrogen sources (NH_4_^+^-ammonium nitrogen, NO_3_^−^-nitrate nitrogen, No-N: nitrogen free BD solution) and concentrations (1 mM and 5 mM) at 1 day (blue bars) or 3 days (red bars). *GmELF1b* was used as a reference gene. The relative gene expression levels were calculated using the method of 2^-ΔΔCq^ to express the ratio between the *GmAMTs* and reference *GmELF1B*. The data were analyzed using student’s *t*-test for significant differences (*, *p* < 0.05; **, *p* < 0.01; ***, *p* < 0.001; ****, *p* < 0.0001). Different letters represent significant differences (Tukey’s test, *p* < 0.05).

To verify the tissue expression patterns of *GmAMTs*, we performed quantitative PCR to analyze the expression patterns of GmAMT family genes in different tissues of soybean collected 24 days after rhizobial (*Bradyrhizobium diazoefficiens* USDA110) inoculation (Figure 9A–H). Indeed, these *GmAMTs* showed different tissue/organ expression patterns, consistent with the RNA-seq data. Among them, *GmAMT1.1* and *GmAMT3.1* were highly expressed in roots (Figure 9A,H). The highest transcript levels of *GmAMT1.2*, *GmAMT1.3*, *GmAMT1.4*, and *GmAMT1.5* were detected in leaves, while relatively lower expression levels of these genes were detected in roots and nodules (Figure 9B–E). Notably, *GmAMT1.6* and *GmAMT2.3* appeared to be specifically expressed in root nodules (Figure 9F,G). These results confirmed the differential expression of *GmAMT* genes during plant growth and development in soybean.

Next, we analyzed the expression patterns of eight putative nitrogen-responsive *GmAMT* genes in the roots and leaves (four each) of plants treated with 1 mM (low nitrogen) and 5 mM (moderate nitrogen) ammonium or nitrate as the nitrogen source for 1 and 3 days. We found that these selected genes were responsive to short-term treatments of low and moderate nitrogen, although the exact expression patterns were different from those in response to long-term normal nitrogen (10 mM) treatment (Figure 9I–P). In roots, compared with the No-N treatment, the expression of *GmAMT1.1* was specifically and rapidly induced by low ammonium treatment (1 mM) at 1 day after treatment, while *GmAMT1.2* were highly upregulated in roots under low ammonium and moderate nitrate conditions (Figure 9I,J). *GmAMT2.2* was highly induced in roots by both low ammonium and nitrate at 1 day after treatment, but was significantly suppressed by moderate ammonium and nitrate at 3 days after treatment (Figure 9K). The expression of *GmAMT2.3* in roots was induced by low and moderate nitrate nitrogen at 1 day after treatment, whereas its expression in roots was markedly suppressed by moderate ammonium nitrogen (Figure 9L). Furthermore, we analyzed the expression patterns of four nitrogen-responsive *GmAMTs* in leaves. Interestingly, compared with the no-nitrogen treatment, *GmAMT1.3*, *GmAMT1.5*, and *GmAMT2.3* showed similar patterns in response to nitrogen. The expression levels of these genes were rapidly and significantly upregulated by low nitrogen regardless of the nitrogen form at 1 day after treatments, but they were only induced by higher ammonium (Figure 9M–O). In comparison, *GmAMT4.1* was induced by moderate levels of both ammonium and nitrate nitrogen at 1 day but decreased with prolonged low-nitrogen treatments (Figure 9P). These results confirm that these *GmAMTs* are responsive to low nitrogen at transcription and may participate in nitrogen uptake and allocation in different tissues in response to the fluctuations in environmental nitrogen levels.

### 2.10. Expression Verification of GmAMTs under ABA Treatment

**Figure 10:**
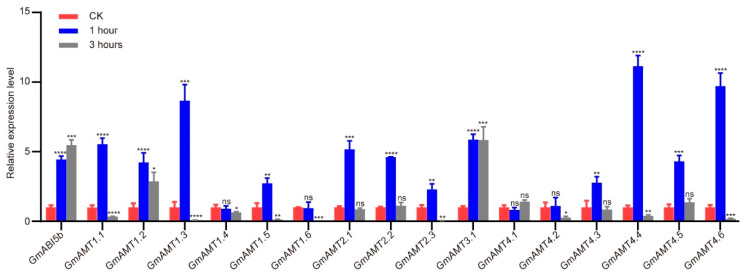
Gene expression analysis of *GmAMTs* in response to exogenous ABA treatment. RT-qPCR was used to analyze the expression of *GmAMTs* in roots treated with 50 μM ABA for 1 and 3 h. *GmABI5b* was used as a positive control. *GmELF1b* was used as a reference gene. The data were analyzed using student’s *t*-test for statistically significant differences (*, *p* < 0.05; **, *p* < 0.01; ***, *p* < 0.001; ****, *p* < 0.0001; ns, no significance).

Due to the fact that there are ABA-responsive elements in the promoters of 13 *GmAMTs* (Figure 5A, Table S3), we hypothesized that these genes may be involved in plant response to ABA. To test the hypothesis, we analyzed the expression levels of *GmAMTs* in soybean roots treated with 50 μM ABA at 1–3 h. As shown in Figure 10, the ABA-responsive gene *GmABI5b* was highly induced by ABA and reached the highest level at 3 h, suggesting that ABA and plant response were effective. As expected, most *GmAMTs* members with ABRE cis elements in their promoters were responsive to exogenous ABA treatment except *GmAMT4.1* (Figure 10). Among 16 *GmAMT* genes, the expression of *12 GmAMTs* (*GmAMT1.1*, *GmAMT1.2*, *GmAMT1.3*, *GmAMT1.5*, *GmAMT2.1*, *GmAMT2.2*, *GmAMT2.3*, *GmAMT3.1*, *GmAMT4.3*, *GmAMT4.4, GmAMT4.5*, and *GmAMT4.6*) was rapidly induced by ABA at 1 h after treatment, while *GmAMT1.4*, *GmAMT1.6*, and *GmAMT4.2* did not respond to ABA treatment. Notably, *GmAMT1.2* and *GmAMT3.1* showed a similar ABA induction expression pattern to *GmABI5b*, whereas *GmAMT1.1*, *GmAMT1.3*, *GmAMT1.4*, *GmAMT1.5*, *GmAMT1.6*, *GmAMT2.3*, *GmAMT4.2*, *GmAMT4.4*, and *GmAMT4.6* were strongly down-regulated compared with that of the control (Figure 10). These results suggest that the majority of GmAMTs family members may be regulated by ABA at transcription levels and mediate plant response to abiotic stresses.

### 2.11. Expression of GmAMTS Is Regulated by GmNINa

**Figure 11:**
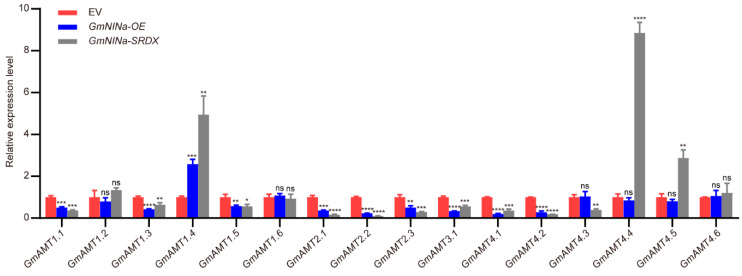
Effect of *GmNINa* on the expression levels of *GmAMT* genes. Detection of *GmAMTs* expression was done by RT-qPCR in hairy roots with empty vector (EV), *35S:GmNINa* (*GmNINa-OE*) or *35S:GmNINa-SRDX* (*GmNINa-SRDX*) at 3 days after rhizobial inoculation. The normalization was performed using *GmELF1b*. The statistically significant differences between groups were analyzed using student’s *t*-test (*, *p* < 0.05; **, *p* < 0.01; ***, *p* < 0.001; ****, *p* < 0.0001; ns, No significance).

GmNINa (soybean Nodule inception a) is the key transcription factor essential for nodulation and symbiotic nitrogen fixation in soybean [46,47,48]. Because the putative GmNINa binding sites were identified in the promoter regions of all GmAMTs genes (Figure 5B), we attempted to investigate whether GmAMTs are regulated by GmNINa during nodulation. We generated the composite plants with transgenic hairy roots overexpressing GmNINa (GmNINa-OE) and GmNINa-SRDX (GmNINa silencing) and examined the expression levels of GmAMTs during nodulation. As shown in Figure 11 and Figure S8, GmNINa showed a different influence on 16 GmAMT genes. The expression of 14 GmAMT genes was affected except GmAMT1.2 and GmAMT4.6. Among them, the expression levels of GmAMT1.1, GmAMT1.3, GmAMT1.5, GmAMT2.1, GmAMT2.2, GmAMT2.3, GmAMT3.1, GmAMT4.1, and GmAMT4.2 were significantly downregulated in both GmNINa-OE and GmNINa-SRDX transgenic roots at 3 days after rhizobial inoculation, whereas GmAMT1.4 was markedly upregulated in both GmNINa-OE and GmNINa-SRDX roots. However, GmAMT4.3, GmAMT4.4, and GmAMT4.5 exhibited different expression patterns. The expression of these three GmAMT genes remained unchanged in GmNINa-OE roots, but GmAMT4.3 and GmAMT4.4, and GmAMT4.5 were strongly down-regualted and up-regulated in the GmNINa-SDRX hairy roots, respectively. These results indicated that the majority of these GmAMTs members might function as downstream components of GmNINa in soybean nodulation.

## 3. Discussion

The *AMT* gene was first recognized as an ammonium transporter gene in *Arabidopsis* [8], and molecular genetic analyses also proved that AMTs are ammonium sensors that can sense the signal for cell–cell communication during plant growth and development of *Arabidopsis* and rice [6,49]. Several AMT homologues have also been shown to play crucial roles in ammonium transport in legumes, such as *Medicago truncatula* and *Lotus japonicus* [28,29,30]. For example, LjAMT2.1 was specifically localized on the bacterial perimembranes of nodules [28], while LjAMT2;2 transports ammonium during arbuscular mycorrhizal fungi symbiosis but not rhizobial symbiosis [29]. This implies that an ancient AMT gene family may be involved in more biological processes in legumes. Soybean is an important legume crop; however, no data are available to explain how *GmAMT* genes evolved and the potential functions of GmAMT homologues. In this study, we performed a genome-wide analysis to identify 16 GmAMT family genes that belong to two subfamilies and different clusters. The systematic analysis results of features, structures of *GmAMT* genes, and coding proteins and expression patterns point to plesiomorphic roles of soybean *AMT* genes during plant development and plant response to environmental conditions.

Soybean has the highest number of GmAMT family genes compared with nonlegume (*Arabidopsis* and rice) and legume (*Medicago truncatula* and *Lotus japonicus*) model plants (Figure S1). There are 16 GmAMT family genes, while the number of *AMT* genes in other plants vary from 6 (*Arabidopsis*) to 12 (rice). The increased number of *GmAMT* genes is likely due to two whole-genome duplications [37]. However, the numbers of genes in the GmAMT1 and GmAMT2 subfamilies of GmAMT family genes did not increase in parallel. Compared with the single copy of the *AMT2* gene in *Arabidopsis*, soybean has 10 *GmAMT2* genes. Our phylogenetic analysis results show that in addition to the conserved GmAMT2 in clade VIII, soybean evolved GmAMT3;1, which is highly homologous to rice OsAMT3 in clade VII, and importantly, soybean-specific GmAMT4.1, GmAMT4.2, and GmAMT4.4 are present in clade V and VI (Figure S1). These findings suggest that the special evolutionary pattern of soybean AMT family genes is likely to confer plants with a higher ability to sense and utilize ammonium during development and to adapt to growth environments. Furthermore, although the majority of GmAMT proteins contain conserved motifs of AMT family proteins, their physicochemical properties, structures, and transmembrane domains vary significantly. For example, four GmAMT proteins (GmAMT1.6, GmAMT4.1, GmAMT4.2, and GmAMT4.6) do not have the complete conserved Ammonium_transp domain (Figure 1), which is essential for ammonium transport activity [36], suggesting that these GmAMT proteins might have new functions. However, similar with the mechanism reported in rice [25,50], these homologous GmAMT proteins might form heteromeric complexes with other AMT proteins containing conserved Ammonium_transp domains to mediate NH_4_^+^ transport activity under normal conditions or in response to environmental stimuli, such as diurnal change, or nitrogen or ABA treatment. Moreover, GmAMT proteins have different numbers of transmembrane domains (Figure S4), and the predicted subcellular localization includes the plasma membrane and the membranes of vacuoles and chloroplasts (Table S1). This suggests that these GmAMTs may be responsible for ammonium distribution to different subcellular regions.

In addition to the changes in gene number and protein characteristics, we found that the *GmAMT* genes are also highly variable in gene structure (Figure 4A,B). First, all *GmAMT1* genes do not have introns, which is same as the *Arabidopsis AMT1.1*. However, unlike *Arabidopsis AMT1.4* and *AMT1.5*, all *GmAMT1* genes have UTRs. Second, all *GmAMT2* genes contain introns, exons, and UTRs, except *GmAMT4.2* and *GmAMT4.3*. Third, the lengths of the introns, exons, and UTRs vary among these *GmAMT2* genes. Introns are usually involved in the regulation of gene expression and/or RNA stability [51]. Mutations in critical regions in gene structure, including coding sequence site and upstream region, may alter the expression patterns and function of members of gene family under evolution events [52,53]. The lack of introns in the AMT1 subfamily genes suggests that the expression of these genes is basically regulated at the transcriptional level. Large variations in the length and number of introns in different AMT2 subfamily genes indicate that these genes may undergo more complicated regulation, such as alternative splicing, mRNA transport, or chromatin assembly, which have been reported previously [54,55]. Last, these *GmAMT* genes are different in the conserved motifs and *cis*-regulatory elements of their promoters (Figure 4C, Figure 5 and Table S3). It is well known that the promoter is the driver of a gene, and the *cis* elements in a promoter determine the spatiotemporal expression of a gene [56]. AMT proteins are in charge of ammonium transport [10,36], and it is conceivable that these *GmAMT* genes are responsive to nitrogen availability and the forms of nitrogen. Unexpectedly, we found that all *GmAMT* genes share *cis*-elements responsive to light and anaerobic conditions and contain different *cis*-elements related to endogenous and exogenous cues. The results suggest that all *GmAMT* genes are likely regulated by light and anaerobic conditions. In addition, these *GmAMT* genes are under the control of different factors during soybean development and responses to environmental conditions, thereby coordinately regulating ammonium uptake and metabolism.

Our expression analyses reveal that these *GmAMT* genes are indeed differentially expressed in different tissues/organs during soybean development and in response to various stimuli (Figure S9). For example, the majority of *GmAMT1s* (*GmAMT1.3*, *GmAMT1.4*, and *GmAMT1.5*) were mainly expressed in leaves, while most *GmAMT2s* (*GmAMT4.1* and *GmAMT4.3*, *GmAMT4.4*, *GmAMT2.3*, and *GmAMT3.1*) were highly expressed in flowers, roots, and nodules (Figure 6 and Figure 9A–H). It is likely that these *GmAMT* genes are functionally differentiated at the transcriptional level. Interestingly, we found that many *GmAMT* genes (*GmAMT1.2-1.6*, *GmAMT2.1*, *GmAMT2.2*, *GmAMT2.3*, and *GmAMT4.6*) are under diurnal control (Figure 7). Nutrient metabolism and plant growth are tightly regulated by the circadian clock, including nitrogen absorption and metabolism [2,24]. Our results suggest that transcriptional oscillations of these *GmAMT* genes in different tissues and organs are involved in the regulation of ammonium transport and metabolism during the day and night.

The key role of *AMT* genes is in the control of ammonium sensing and uptake [3,10,36]. In this study, we found that these *GmAMT* genes are differentially expressed in response to ammonium, nitrate, and urea, and this conclusion is supported by the existence of nitrogen-related *cis*-elements (Figure 5B and Figure 8). Interestingly, these *GmAMT* genes are not only responsive to ammonium; some of them are regulated by all forms of nitrogen, while some of them are responsive to only one or two forms of nitrogen (Figure 8 and Figure 9I–P). These results suggest that GmAMTs may differentially and redundantly regulate plant responses to different nitrogen forms. It is worth noting that plants often face low-nitrogen conditions that limit plant development and crop yields. Our results that show that *GmAMT* genes are differentially expressed in roots and leaves in response to low and moderate ammonium and nitrate (Figure 9I–P) implicate an important role of these *GmAMT* genes in plant adaptation to low-nitrogen conditions. As a sessile organism, soybean constantly encounters unfavorable environmental stresses, such as drought and salt stress. Soybean plants have to coordinate nitrogen metabolism with stress response, allowing better plant growth [57,58]. Our results showed that most *GmAMT* genes contain ABRE cis elements in their promoters and their expression is regulated by ABA (Figure 5A, Figure 10 and Table S3). Our results support the notion that ABA signaling may enhance the function of GmAMTs through regulating their transcription. Differential expression of *GmAMTs* in response to ABA suggests that these *GmAMT* genes may redundantly or coordinately regulate ammonium uptake, thereby obtaining sufficient nitrogen for their growth under stress conditions. In addition, we found that many *GmAMT* genes may be regulated by nodulation signaling. There are putative GmNINa binding sites in their promoter regions, and most importantly, alterations in *GmNINa* expression affect the expression of most *GmAMTs* (Figure 5B and Figure 11). Thus, we conclude that *GmAMT* genes play crucial roles in the control of absorption, assimilation, and remobilization of nitrogen in soybean under normal condition or different environmental conditions. Further genetic and molecular analyses will establish the molecular link between *GmAMTs* and ammonium uptake with stress signaling and/or nodulation signaling.

In summary, this study provides a comprehensive understanding of *AMT* genes and proteins in soybean. We also explore the potential functions of the GmAMT family genes involved in plant growth and development, the circadian rhythms of nitrogen metabolism, plant responses to nitrogen availability, and environmental stresses (Figure S9). It is apparent that *GmAMT* genes are involved in ammonium uptake and assimilation and are essential for plant development and adaptation to insufficient nitrogen in growth environments. Future studies need to focus on the functions of these *GmAMT* genes to help elucidate the genetic regulatory mechanisms integrating ammonium sensing, uptake and remobilization and assimilation into plant growth, and environmental adaptation in soybean as well as in other plants.

## 4. Materials and Methods

### 4.1. Identification of AMT Genes in Different Species

The genomic information and annotation files of *Arabidopsis thaliana* [59], *Oryza sativa* [60], *Medicago truncatula* [61], *Lotus japonicus* [62] and *Glycine max* [63] were downloaded from the Phytozome database (https://phytozome-next.jgi.doe.gov/, accessed on 5 October 2022). The hidden Markov model (HMM) of the conserved protein domain Ammonium_transp (PF00909) was downloaded from the Pfam database (http://pfam-legacy.xfam.org/ accessed on 5 October 2022) [64]. A simple HMM search of TBtools software [65] was used to obtain ammonium transporters in different species. AMTs without conserved Ammonium_transp domains were removed according to the NCBI Conserved Domain Database (NCBI-CDD) tool (https://www.ncbi.nlm.nih.gov/Structure/cdd/wrpsb.cgi accessed on 5 October 2022) [66].

### 4.2. Phylogenetic Tree Analysis of AMTs

The full-length amino acid sequences of AMTs from *Arabidopsis thaliana*, *Oryza sativa*, *Medicago truncatula*, *Lotus japonicus*, and *Glycine max* were downloaded from the Phytozome database. The amino acid sequences of AMTs were aligned by MEGA-X software [67], and a phylogenetic tree was constructed by the maximum-likelihood method (ML). Bootstrap analysis was calculated for 1000 replicates. The evolutionary tree was visualized on the web-based tool Interactive Tree Of Life (iTOL, https://itol.embl.de/ accessed on 5 October 2022) [68].

### 4.3. Physicochemical Properties and Transmembrane Structure Analysis of GmAMT Proteins

The molecular weights (kDa) and isoelectric points (pI) of GmAMT family proteins were obtained through the ExPASy website (https://web.expasy.org/protparam/ accessed on 6 October 2022) [69]. Prediction of subcellular localization of AMT family proteins was performed using the online website WOLF PSORT (https://www.genscript.com/wolf-psort.html accessed on 6 October 2022) [70]. Transmembrane structure prediction of GmAMT proteins was performed using the TMHMM website (https://services.healthtech.dtu.dk/service.php?TMHMM-2.0 accessed on 6 October 2022) [71].

### 4.4. Gene Structure, Conserved Motif and Conserved Protein Domain Analyses of AMTs

The promoter sequences, CDSs, genomic sequences, and amino acid sequences of AMTs were obtained from the Phytozome database. The gene structures of *AMTs* were analyzed using the GSDS2.0 website (Gene Structure Display Server, http://gsds.gao-lab.org/ accessed on 8 October 2022) [72]. The MEME (https://meme-suite.org/meme/tools/meme accessed on 8 October 2022) online tool [73,74] was used to identify the conserved motifs in the promoter regions of *AMT* genes and AMT amino acid sequences. The conserved domains of AMT proteins were analyzed by the NCBI CD-search tool (https://www.ncbi.nlm.nih.gov/Structure/cdd/wrpsb.cgi accessed on 5 October 2022) [75]. TBtools software was used to integrate the phylogenetic tree, gene structures, and conserved motifs identified in the promoters and AMT proteins and the conserved domains [65].

### 4.5. Chromosome Localization Analysis and Ka/Ks Analyses

The GFF file of the soybean genome was downloaded from the Phytozome database. The chromosome gene density and *GmAMT* locations on chromosomes were displayed using TBtools. The DnaSP6 tool was used to analyze the ratio of nonsynonymous substitutions (Ka) to synonymous substitutions (Ks) of gene pairs (Ka/Ks) [76]. The Ka/Ks value can be used as a molecular indicator of nucleic acid evolution to determine whether GmAMT genes have undergone selective pressure [38].

### 4.6. Synteny Analysis of AMT Genes

Collinearity analysis of AMT family genes was performed using TBtools software [65]. *Arabidopsis thaliana*, *Oryza sativa*, *Lotus japonicus*, *Medicago truncatula*, and *Glycine max* genomes were compared using One-Step MCScanX-Super Fast (default parameters e-value < 1e^−3^), and all possible collinear gene pairs between chromosomes were calculated. TBtools was used to map the collinearity graphs and meanwhile highlight the *AMT* genes on the graphs.

### 4.7. Cis-Element Analysis of GmAMT Promoter Regions

The 2 kb promoter sequences of *GmAMTs* upstream of the start codons were obtained from the Phytozome database. The *cis*-elements in the promoter regions of 16 *GmAMT* genes were analyzed using the PlantCARE online website (https://bioinformatics.psb.ugent.be/webtools/plantcare/html/ accessed on 8 October 2022) [77]. The data were visualized by TBtools [65].

### 4.8. Expression Analysis of the GmAMT Genes in Soybean

Tissue expression data of *GmAMT* genes were downloaded from the Soybean Expression Atlas (https://venanciogroup.uenf.br/cgi-bin/gmax_atlas/index.cgi accessed on 10 October 2022) [44]. *GmAMT* gene expression data in response to different nitrogen treatments or diurnal variation were obtained from the JGI Plant Gene Atlas (https://plantgeneatlas.jgi.doe.gov/ accessed on 10 October 2022) [45]. Gene expression heatmaps were constructed by TBtools software [65].

### 4.9. Plant Materials and Growth Conditions

Seeds from soybean (*Glycine max*) cultivar Williams 82 (Wm82) were sown in vermiculite soaked in nitrogen-free BD nutrient solution as described by Broughton and Dilworth [78] and cultured in a greenhouse under controlled conditions with a 16 h light/8 h dark program at 26 °C, a light intensity of 200 μmol/m^2^/s, and 60% relative humidity. Distilled water and BD nutrient solution were alternatively used to irrigate soybean plants every 5 days.

To analyze tissue-specific gene expression, 8-day-old plants were inoculated with *Bradyrhizobium diazoefficiens* USDA110 (OD_600_ = 0.08, 30 mL per plant). Different tissues, including leaves, stems, roots, and nodule tissues, were taken at 24 days after rhizobium inoculation.

For samples treated with different nitrogen conditions, 8-day-old soybean plants were treated with 1 mM and 5 mM nitrate nitrogen and ammonium nitrogen, respectively. The leaves and roots were taken at 1 and 3 days after treatment for subsequent experiments.

For samples treated with ABA, 8-day-old soybean plants were pretreated with 10 mM PBS solution (pH7.4) for 8 h (4 plants/150 mL solution), then treated with 50 μM ABA (4 plants/150 mL solution). The experimental method was improved according to Guo et al. description [79]. The roots were taken at 1 and 3 h after treatment for subsequent experiments. *GmABI5b* is a homologous gene of *Arabidopsis AtABI5*, which was used as a classic ABA-inducible gene [80].

### 4.10. Soybean Hairy Root Transformation

Soybean hairy root transformation was done using *Agrobacterium rhizogenes* K599 carrying *GmNINa*-pMDC32 (*GmNINa-OE*), *GmNINa*-SRDX-pMDC32 (*GmNINa-SRDX*) or pMDC32 (Empty vector) plasmid according to methods described by Wang et al. [81]. The composite plants were inoculated with USDA110 (OD_600_ = 0.08) at 10 days after transplantation (30 mL per plant). Three days after being inoculated with USDA110, the transgenic hairy roots were taken for analyzing gene expression. The primers of *GmNINaCDS* or *GmNINaUTR* were used for analyzing *GmNINa* expression in transgenic hairy roots having *GmNINaOE* or *GmNINa-SRDX*, respectively [82].

### 4.11. RNA Extraction and RT-qPCR

To assay the expression of *GmAMT* genes, total RNA was extracted from different samples using TRIpure reagent (Aidlab Biotechnologies). cDNA was synthesized from the RNA by a reverse transcription reagent kit (Hifair^®^ II 1st Strand cDNA Synthesis SuperMix for qPCR (gDNA digester plus), YEASEN). Real-time quantitative PCR was performed using Hieff^®^ qPCR SYBR Green Master Mix (No Rox) (YEASEN). *GmELF1b* was used as an internal control gene [83]. Gene-specific primers are listed in Table S4.

## 5. Conclusions

In this study, we gained a better understanding of ammonium transporter proteins in soybean. Except for the conserved GmAMTs, the number of GmAMT family members increased, and legume-specific AMTs were identified. The gene structures and the conserved motifs of GmAMT1 and GmAMT2 members appeared to be different based on the results of *cis*-element analysis and gene expression patterns in different tissues and in response to nitrogen treatment and ABA. GmAMTs might be involved in ammonium uptake and assimilation and may also be required for soybean plastic development under nitrogen deficiency conditions and in response to environmental stresses. Based on the result of the occurrence of *cis*-elements and gene expression data, some GmAMTs members have the potent function to participate in symbiosis downstream of GmNINa. These data not only provide important clues for further studies on the conserved and legume-specific function of GmAMTs but also provide insights into the molecular mechanisms involved in the regulation of GmAMTs in soybean.

## Data Availability

The datasets used and/or analyzed during the current study are available from the corresponding author on reasonable request. However, most of the data are shown in the Supplementary files.

## Supplementary Materials

The following supporting information can be downloaded at: https://www.mdpi.com/article/10.3390/ijms24043991/s1.
