# Supplementary material for: Genome-Wide Identification and Expression Analysis of the Ammonium Transporter Family Genes in Soybean
Source: Int J Mol Sci. 2023 Feb 16;24(4):3991. doi: 10.3390/ijms24043991 (PMC9960152; doi:10.3390/ijms24043991)
Supplement: Supplementary file 1 [file ijms-24-03991-s001.zip › ijms-2192086-Supplementary Figure.pdf]

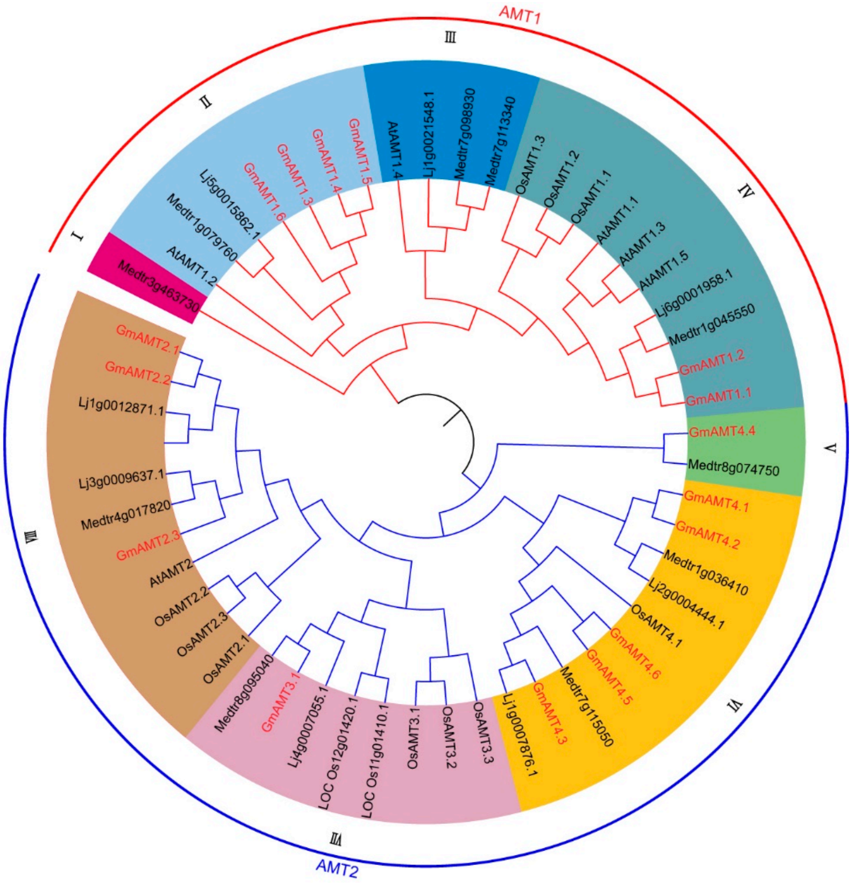

Figure S1. Phylogenetic tree analysis of AMTs from *Arabidopsis thaliana* (At), *Oryza sativa* (Os), *Medicago truncatula* (Medtr), *Lotus japonicus* (Lj) and *Glycine max* (Gm). The full-length amino acid sequences of AMTs were obtained from the Phytozome database. Phylogenetic tree was constructed by the MEGA-X software with 1000 bootstrap replicates using the neighbor-joining method. A total of 53 AMT proteins were divided into AMT1 and AMT2 subfamilies. Different colors of circles represent different clusters. AMTs of *Glycine max* are highlighted by red letters.

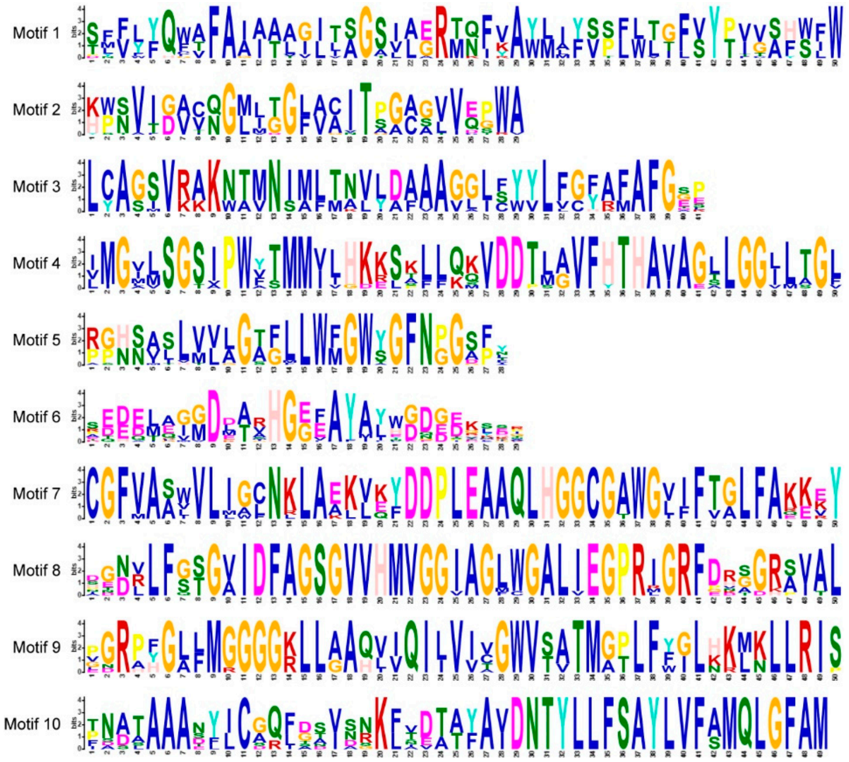

Figure S2. The conversed motif logos of GmAMT amino acid sequences. The logos of conversed motifs of GmAMT proteins were analyzed by the MEME online program. The character and size of each logo indicates the probability of an amino acid at the specific site.

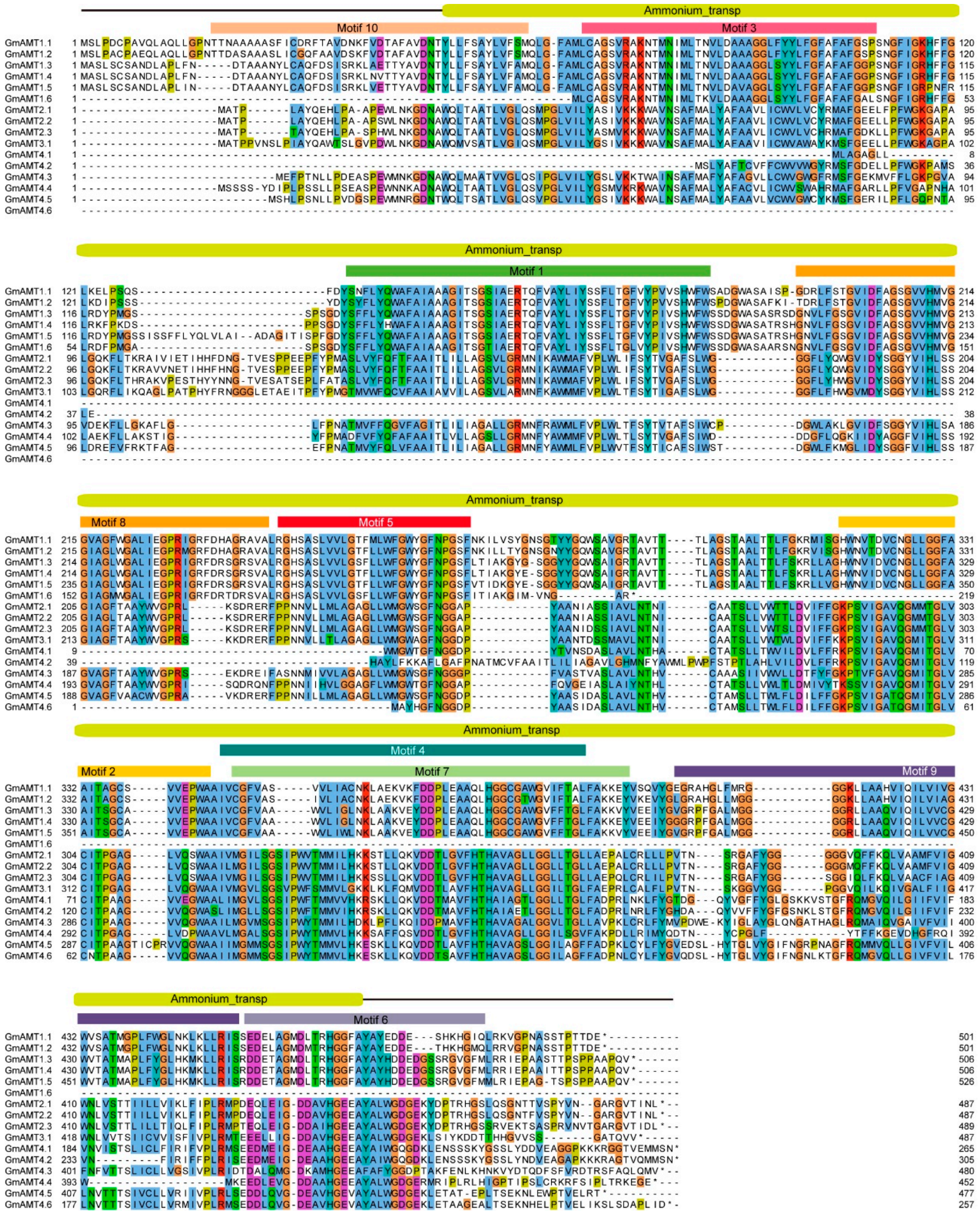

Figure S3. Alignment of the amino acid sequences of GmAMT family members. The amino acid sequences of GmAMTs were aligned by MEGA-X software. Ten conserved motifs are indicated by boxes with different colors.

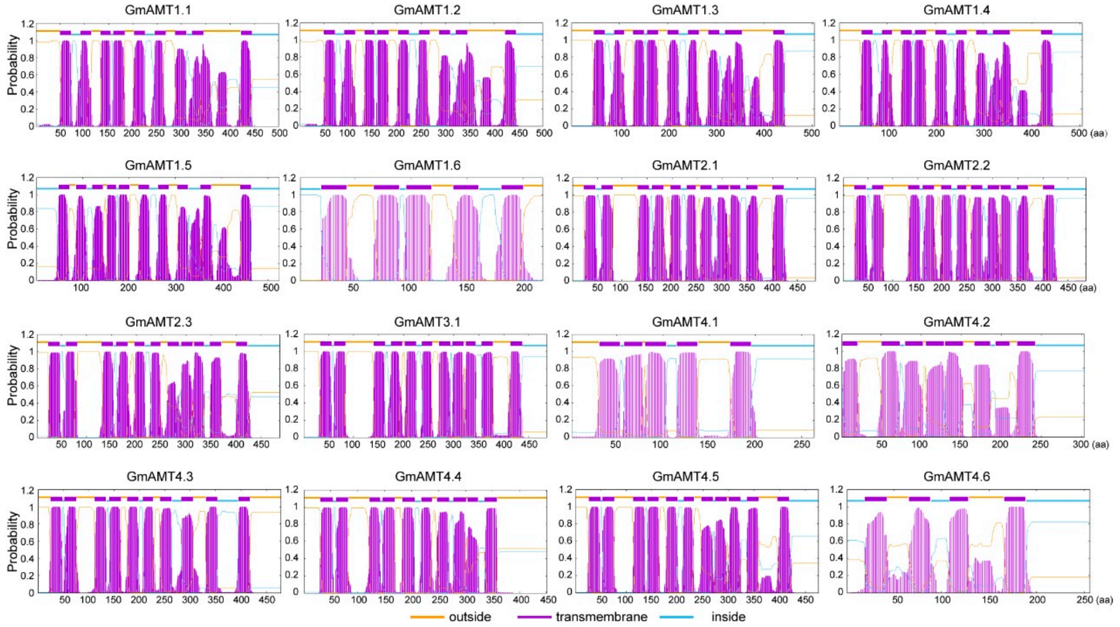

Figure S4. Transmembrane structure prediction of GmAMT proteins. The amino acid sequences of GmAMT proteins were analyzed and predicted the transmembrane structures by TMHMM Server v.2.0. Orange line represents outside, purple line indicates transmembrane, blue line represents inside.

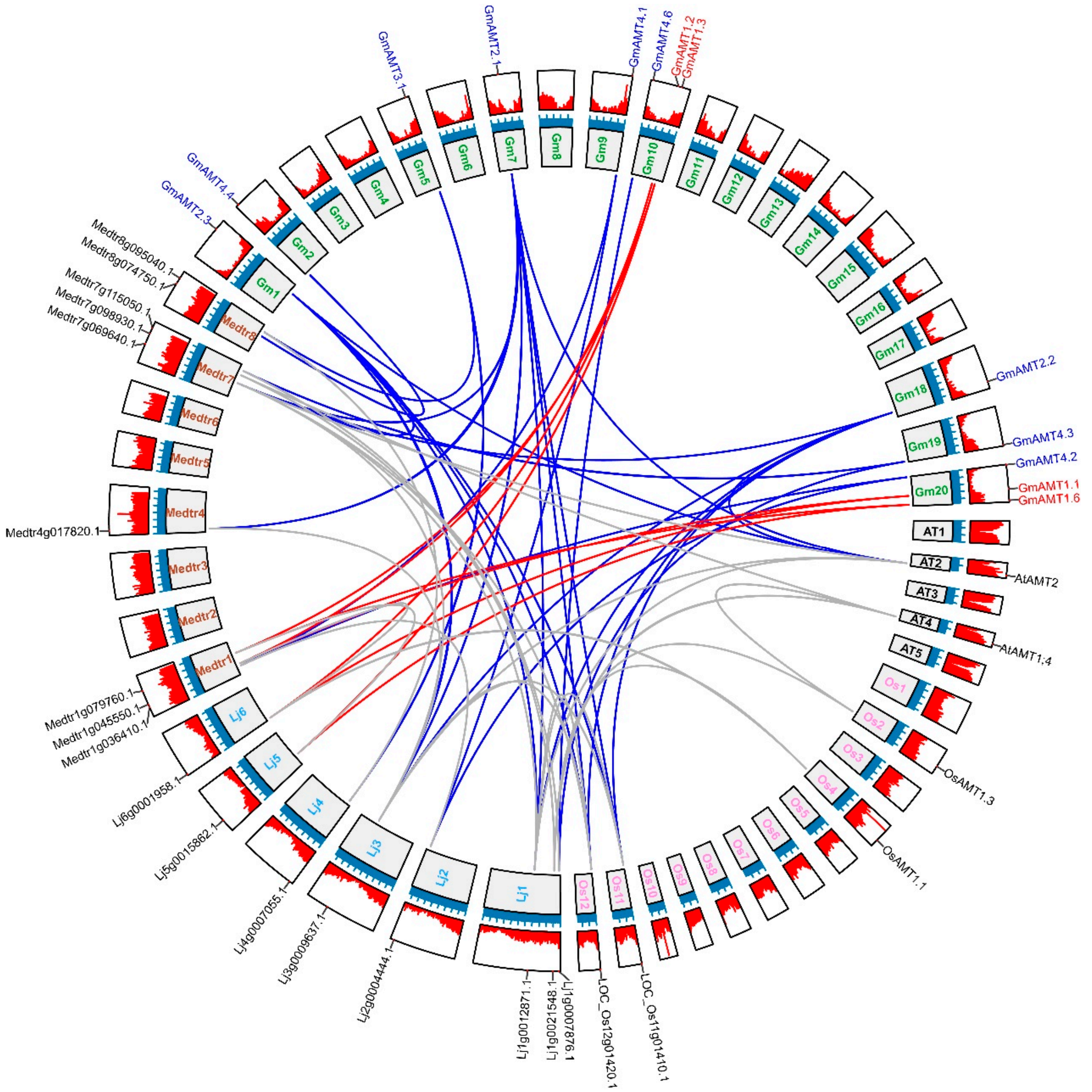

Figure S5. Synteny analysis of *AMT* genes in Arabidopsis, soybean, Lotus, Medicago and rice. The genomics sequences were aligned by One Step MCScanX-Super Fast of TBtools. The circos tracks showing chromosome length, gene density and gene location from inner to outer. Red lines represent syntenic AMT1 subfamily gene pairs in soybean, blue lines indicate syntenic AMT2 subfamily gene pairs in soybean, gray lines represent syntenic *AMTs* gene in Medicago, Lotus and rice.

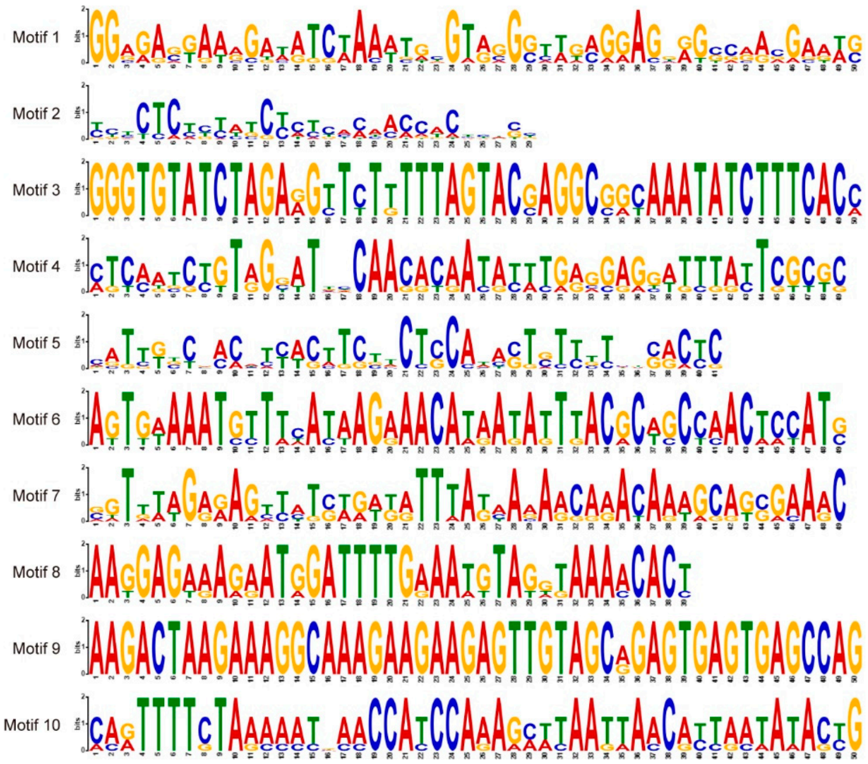

Figure S6. Sequence logos of conserved motifs on the promoter of *AMTs* in *Arabidopsis* and soybean. The logos of the conversed motifs identified from the promoter regions of *AMT* genes were generated from MEME analysis. The height of each letter represents the relative abundance of each nucleotide in the sequence.

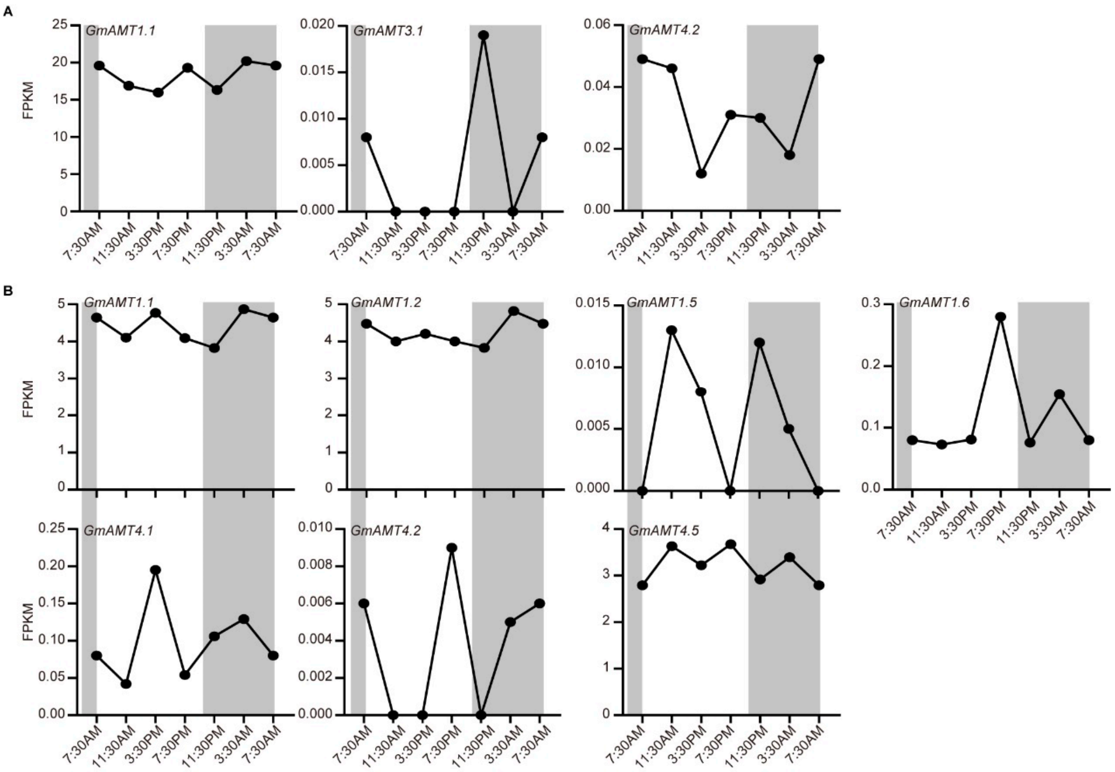

Figure S7. The day-night expression of GmAMT family genes by RNA-seq data. The FPKM data of *GmAMT* genes were collected from the JGI Plant Gene Atlas [78]. The day-night expression patterns of *GmAMTs* in leaves **(A)** and nodules **(B)**. Dark periods are highlighted as shaded columns.

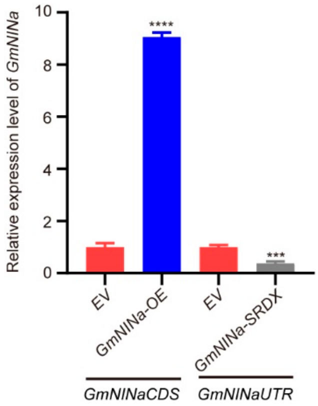

Figure S8. Expression analysis of *GmNINa*. RT-qPCR was used to detect the expression of *GmNINa* in transgenic hairy roots containing empty vector (EV), *35S:GmNINa* (*GmNINa-OE*) or *35S:GmNINa-SRDX* (*GmNINa-SRDX*) at 3 days after rhizobial inoculation. *GmELF1b* was used as a reference gene. The relative gene expression levels were calculated using the method of $2^{-\Delta\Delta Cq}$ to express the ratio between the *GmNINa* and reference *GmELF1B*. The data were analysed using student's *t*-test for statistically significant differences (***, P<0.001; ****, P<0.0001).

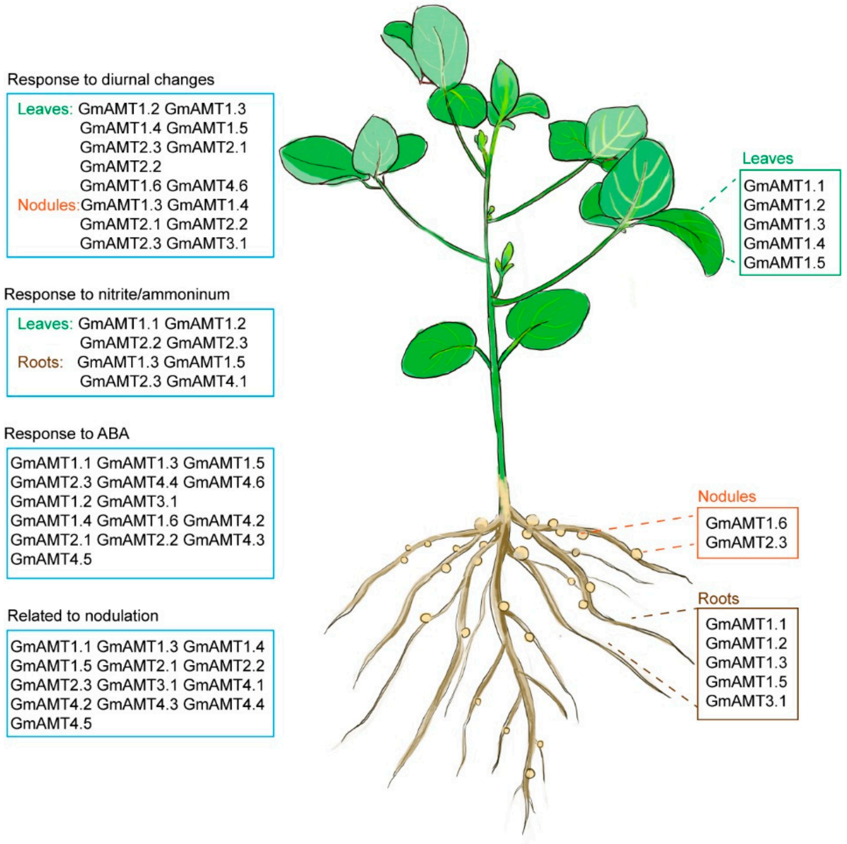

Figure S9. Function prediction of *AMTs* in soybean. The putative functions of GmAMT family members in different tissues or response to different environmental stimuli were summarized in the picture mainly based on RT-qPCR data.
