# Supplementary material for: Genome-Wide Identification and Expression Analysis of the Ammonium Transporter Family Genes in Soybean
Source: Int J Mol Sci. 2023 Feb 16;24(4):3991. doi: 10.3390/ijms24043991 (PMC9960152; doi:10.3390/ijms24043991)
Supplement: Supplementary file 1 [file ijms-24-03991-s001.zip › Supplementary Data1.pdf]

Supplementary Data 1.

The amino acid sequences of AMTs in different species.

>AtAMT2(AT2G38290)
MAGAYDPSLPEVPEWLNKGDNAWQLTAATLVGLQSMPGLVILYASIVKKKWAVNSAFMALYAF
AAVLLCWVLLCYKMAFGEELLPFWGK
GGPAFDQGYLKGQAKIPNSNVAAPYFPMATLVYFQFTFAAITTILVAGSVLGRMNIKAWMAFV
PLWLIFSYTVGAYSIWGGGFLYQWGVIDYSGGYVIHL
SSGVAGFVAAYWVGPRPKADRERFPPNNVLLMLAGAGLLWMGWSGFNGGAPYAANLTSSI
AVLNTNLSAATSLLVWTTLDVIFFGKPSVIGAIQGMVTGL
AGVTPGAGLIQTWAAIIIGVVSGTAPWASMMIIHKKSALLQKVDDTLAVFYTHAVAGLLGGIMT
GLFAHPDLCVLVLPLPATRGAFYGGNGGKQLLKQLA
GAAFIAVWNVVSTTIILLAIRVFIPLRMAEEELGIGDDAAHGEEAYALWGDGEKFDATRHVQQF
ERDQEAAHPSYVHGARGVTIVL*

>AtAMT1.2(AT1G64780)
MDTATTTCSAVDLSALLSSSSNSTSSLAAATFLCSQISNISNKLSDTTYAVDNTYLLFSAYLVFA
MQLGFAMLCAGSVRAKNTMNIMLTNVLDAAAGAIS
YYLFGFAFAFGTPSNGFIGRHHSFFALSSYPERPGSDFSFFLYQWAFAIAAAGITSGSIAERTQ
FVAYLIYSTFLTGFVYPTVSHWFWSSDGWASASRSD
NNLLFGSGAIDFAGSGVVHMVGGIAGLCGALVEGPRIGRFDRSGRSVALRGHSASLVVLGTF
LLWFGWYGFNPGSFLTILKGYDKSRPYYGQWSAVGRTA
VTTTLSGCTAALTTLFSKRLLAGHWNVIDVCNGLLGGFAAITSGCAVVEPWAAIVCGFVASWV
LIGFNLLAKKLKYDDPLEAAQLHGGCGAWGLIFTGLF
ARKEYVNEIYSGDRPYGLFMGGGGKLLAAQIVQIIVIVGWVTVTMGPLFYGLHKMNLLRISAE
DEMAGMDMTRHGGFAYAYNDEDDVSTKPWGHFAGRVE
PTSRSSTPTPTLTV*

>AtAMT1.5(AT3G24290)
MSGAITCSAADLSALLGPNATAAADYICGQLGSVNNKFTDAAYAIDNTYLLFSAYLVFAMQLGF
AMLCAGSVRAKNTMNIMLTNVLDAAAGGLFYYLFGY
AFAFGESSDGFIGRHNFGLQNFPTLTSDYSFFLYQWAFAIAAAGITSGSIAERTKFVAYLIYSSF
LTGFVYPVVSHWFWSPDGWASPFRSEDRLFGTGAI
DFAGSGVVHMVGGIAGLWGALIEGPRIGRFPDGGHAIALRGHSASLVVLGTFLLWFGWYGFN
PGSFTKILIPYNSGSNYGQWSGIGRTAVTTTLSGCTAA
LTTLFGKRLLSGHWNVTDVCNGLLGGFAAITAGCSVVDPWAAIVCGFVASLVLIGCNKLAELL
KYDDPLEAAQLHGGCGAWGLIFVGLFAKEKYINEVYG
ASPGRHYGLFMGGGGKLLGAQLVQIIVIVGWVSATMGTLFFILKKLNLLRISEQHEMRGMDLA
GHGGFAYIYHDNDDDSIGVPGSPVPRAPNPPAV*

>AtAMT1.3(AT3G24300)
MSGAITCSAADLATLLGPNATAAADYICGQLGTVNNKFTDAAFAIDNTYLLFSAYLVFAMQLGF
AMLCAGSVRAKNTMNIMLTNVLDAAAGGLFYYLFGY
AFAFGGSSEGFIGRHNFALRDFPTPTADYSFFLYQWAFAIAAAGITSGSIAERTQFVAYLIYSSF
LTGFVYPVVSHWFWSPDGWASPFRSADDRLFSTGA
IDFAGSGVVHMVGGIAGLWGALIEGPRRGRFEKGGRAIALRGHSASLVVLGTFLLWFGWYGF
NPGSFTKILVPYNSGSNYGQWSGIGRTAVNTTLSGCTA
ALTTLFGKRLLSGHWNVTDVCNGLLGGFAAITAGCSVVEPWAAIVCGFMASVVLIGCNKLAEL

VQYDDPLEAAQLHGGCGAWGLIFVGLFAKEKYLNEVY
GATPGRPYGLFMGGGGKLLGAQLVQILVIVGWVSATMGTLFFILKRLNLLRISEQHEMQGMD
MTRHGGFAYIYHDNDDESHRVDPGSPFPRSATPPRV*
>AtAMT1.1(AT4G13510)
MSCSATDLAVLLGPNATAAANYICGQLGDVNNKFIDTAFAIDNTYLLFSAYLVFSMQLGFAMLC
AGSVRAKNTMNIMLTNVLDAAAGGLFYYLFGYAFAF
GSPSNGFIGKHYFGLKDIPTASADYSNFLYQWAFAIAAAGITSGSIAERTQFVAYLIYSSFLTGF
VYPVVSHWFWSVDGWASPFRTDGDLLFSTGAIDFA
GSGVVHMVGGIAGLWGALIEGPRLGRFDNGGRAIALRGHSASLVVLGTFLLWFGWYGFNPG
SFNKILVTYETGTYNGQWSAVGRTAVTTTLAGCTAALTT
LFGKRLLSGHWNVTDVCNGLLGGFAAITGGCSVVEPWAAIICGFVAALVLLGCNKLAEKLKYD
DPLEAAQLHGGCGAWGLIFTALFAQEKYLNQIYGNKP
GRPHGLFMGGGGKLLGAQLIQIIVITGWVSATMGTLFFILKKMKLLRISSEDEMAGMDMTRHG
GFAYMYFDDDESHKAIQLRRVEPRSPSPSGANTTPTP
V*
>AtAMT1.4(AT4G28700)
MASALSCSASDLIPLLSGGANATAAAAAAEYICGRFDTVAGKFTDAAYAIDNTYLLFSAYLVFA
MQLGFAMLCAGSVRAKNTMNIMLTNVIDAAAGGLFY
YLFGFAFAFGSPSNGFIGKHFFGMYDFPQPTFDYPYFLYQWTFAIAAAGITSGSIAERTQFVAY
LIYSSFLTGLVYPIVSHWFWSSDGWASPARSENLLF
QSGVIDFAGSGVVHMVGGIAGLWGALIEGPRIGRFGVGGKPVTLRGHSATLVVLGTFLLWFG
WYGFNPGSFATIFKAYGETPGSSFYGQWSAVGRTAVTT
TLAGCTAALTTLFGKRLIDGYWNVTDVCNGLLGGFAAITSGCSVVEPWAALVCGFVAAWVLM
GCNRLAEKLQFDDPLEAAQLHGGCGAWGIIFTGLFAEK
RYIAEIFGGDPNRPFGLLMGGGGRLLAAHVVQILVITGWVSVTMGTLFFILHKLKLLRIPAEDEI
AGVDPTSHGGLAYMYTEDEIRNGIMVRRVGGDNDP
NVGV*
>Lj1g0021548.1
MAAAFTCSAADLHPLLGTGANATAAAEFICSRFGAISNKFTDTAYAVDNTYLLFSAYLVFAMQL
GFAMLCAGSVRAKNTM
NIMLTNVLDAATGGIFFYIFGFALAFGTPSNGFIGKHFFGLNEFPSPSFDYGFFLYQWAFAIAAA
GITSGSIAERTQFVA
YLIYSSFLTGLVYPIVAHWFWSSDGWGSPARTENLLFGSGVIDFAGSGVVHLVGAVAGFWGA
FIEGPRMGRFDHAGKPVP
LRGHSGTLVVLGTFLLWFGWYGFNPGSFITILKSYGESGSGNYYGQWSAIGRTAVTTTLAGC
SAALTTLFGKRLQTGHWN
VTDVCNGLLGGFAAITSGCSVVDPWAAIICGFVAAWVLIGCNVLAEKFHYDDPLEAAQLHGG
CGTWGIIFTALFAKKQYV
NEVYAGLPDRPYGLLMGGGWRLLAAHVVQILTIAGWVSVTMGAVFFVLHKLNLLRISPEEEM
AGLDMTSHGGLAYEYHED
VGSVQKALEIRV*
>Lj1g0007876.1
MALPSNLLPDEASPEWLNKGDNAWQLTAATLVGLQSIPGLVILYGSLVKKTWAVNSAFMAFYA
FAMVLLCWVAWGFCMSF

GEKLVFFLGKPGVALDENFLLGKAFLGMFPNATMVLFQGVFAGITLILIAGALLGRMNIRAWM
MFVPLWVSLSYSVTAFS
IWCPDGWLAKLGVIDFAGGYVIHLSAGVAGFTAAYWVGPRPEKEREMFPANNMILVLAGAGL
LWMGWSGFNGGAPFAAST
VASLAILNTHVCTSASIVVWVLLDTFYFGKPTVFGAVQGMITGLVCITPAAGVVQGWAAILMGL
ISGSIPWYTMMILHNK
VPFLKQIDDPMAVFHTHAVAGALGGILTGFFAVPKLCRLFYMVPDWEKYIGLAYGLQNGATQA
GLKQMGIQVVAIVFVIC
FNFVSTTLICLLVRLMVPLRLDSDELQGDKAMHGEEAFNLHGGGGDHHAVKFEKVKHNKVY
DTPEFSFINESTSSVFVNL
QMV*
>Lj1g0012871.1
MSLPTAYQEHLPAAPYWLNKGDNAWQMTASTLVGLQSMPGLVILYASIVKKKWAVNSAFMAL
YAFAAVLICWVLVGYRMA
FGEELLPFWGKGAPALGQKFLIRQAKVPESTHFYKNGTLETSTEEPFFPMASLVYFQFTFAAI
TMILLAGSVLGRMNIKA
WMAFVPLWLIFSYTVGAFSLWGGGFLYHWGVIDYSGGYVIHLSSGIAGFTAAYWVGPRVKSD
RERFPPNNILLMLAGAGL
LWMGWSGFNGGAPYAANRAASLAVLNTNVSAATSLLVWTCLDVIFFGRPSVIGAVQGMMTG
LVCITPGAGLVQSWAAIVM
GIAAGSIPWFTMMILHKKSSLLQKVDDTLGVFHTHAVAGLLGGLLTGLLAEPALCRLLLPVTNS
RGAFYGGDGGVQFVKQ
LVAALFVIGWNLVSTTIILLVIQLFIPLRMPDEQLEIGDDAVHGEEAYALWGDGEKFDPTRHGSIT
TGHGVSPYVNGARG
VTINL*
>Lj3g0009637.1
MATPTAYQENLPASPEWLNKGDNAWQMTAATLVGLQSMPGLVILYASIVKKKWAVNSAFMAL
YAFAAVLLCWVLLCYRMA
FGDKLFPFWGKGAPALGQKFLINQASVPESTHYFNNGSIETQTQRPFYPMATLVYFQFNFAAI
TMILLAGSVLGRMNIKA
WMAFVPLWLIFSYTVGAFSIWGGGFLYHWGVIDFSGGYVIHLSSGIAGFTAAYWVGPRLKSD
RERFPPNNVLLMLAGAGL
LWMGWSGFNGGAPYAANIDASIAVLNTNICAATSLLVWTSLDVAFFGKPSVIGAVQGMMTGLV
CITPGAGLVQSWAAIVM
GILSGSIPWVSMMILHKKSSLLQKVDDTLGVFHTHAVAGLLGGLLTGLLAEPELCRLVLPVSNS
RGAFYGGTGGAQFLKQ
LVAALFVIGWNLVSTTLILLAIQLFIPLRMPDYQLEIGDDAVHGEEAYALWGDGEKYDPTKHGS
TIVDETLASAYSVGAR
GVTVNL*
>Lj5g0015862.1
MASLSCSATDLAPLLTATTNATATAAATYLCNQLDTISRKLSDTTYAVDNTYLLFSAYLVFAMQL
GFAMLCAGSVRAKNT
MNIMLTNVLDAAAGGLSYYLFGFAFAFGAPSNGFIGRHFFGLKHYPSPTYDYSFFLYQWAFAI
AAAGITSGSIAERTQFV

AYLIYSSFLTGFVYPVVSHWLWSSDGWASPTRTTGSLLFGSGAIDFAGSGVVHMVGGIAGLW
GAFIEGPRIGRFDRTGRS
VALRGHSASLVVLGSFLLWFGWYGFNPGSFLTIAKAYGNNGENGNYYGQWSAIGRTAVTTTL
AGCTAALTTLFSKRLLEG
HWNVLDVCNGLLGGFAAITSGCSVVEPWPAIVCGFVAAWVLIGLNLVAAKMKYDDPLEAAQL
HGGCGAWGVLFTGLFAKG
EYVAEVYGSAGRPYGLLMGGGGKLLAAQVIEILVVCGWVTATMGPLFYGLHKMKLLRISEDD
ETAGMDLTRHGGFAYAYH
DEDDVSTKRGVMMSRIGPGSSSPSTMNTPAASAAANDC*
>Lj2g0004444.1
MSTVVPLPSNLAFSDASPEWMNKADNAWQLTAATLVGLMSIPGLMIMYGGAVKKKWAVNSA
FMALYAFACVIFCWVFWGY
RMSFGDKLFPFLGKPAVALDEKYLFKPAFFGMFPNVTMVYFQGVFAAITEILLAGAVLGRMNF
YAWMLFVPLWLTFSYTF
VAFSIWCPAGFLSKMGLIDYSGGYVIHLSSGVAGFTAAYWVGPRLNKDRERFPPNNILLMLAG
AGLLWMGWTGFNGGDPY
TVSMDASLAVLNTHACTATSLLTWVILDVIFFRKPSVIGAVQGMITGLVCITPAAGVVQGWAALI
MGVFSGSIPWFTMMV
IHKRSKLLQKVDDTMAVLHTHAIAGSLGGILTGLFAEPRLNYLFFGTYNKYVGLLYGIQMNMFG
TGLRQLGVQLFGILFV
VVVNVISTSLICLLVQLFVPLRMSEEDMEIGDEAAHGEEAYAIWGQGDKLENSSSKYGSSLYE
DLEAPGATKNKRGGGQL
EMTSN*
>Lj6g0001958.1
MALPECSAANLAQLIGPNATDAAAVAGFICDQFTAVGQRFSDTAFAVDSTYLLFSAYLVFSMQL
GFAMLCAGSVRAKNTM
NIMLTNVLDAAAGGLFYYLFGFAFAFGAPSNGFIGRHFFGLKDVPTVAFDYSYFLYQWAFAIAA
AGITSGSIAERTQFVA
YLIYSSFLTGFVYPVVSHWFWSGDGWASATNTGNLLFGTGVIDFAGSGVVHMVGGVAGLWG
ALIEGPRIGRFDHAGRAVS
LRGHSASLVVLGTFMLWFGWYGFNPGSFTKILSAYDSGNYYGQWSAIGRTAVTTTLAGCTAA
LTTLFGKRIISGHWNVTD
VCNGLLGGFAAITAGCSVVEPWAAIVCGFVAAVVLIACNMLAEKVRYDDPLEAAQLHGGCGA
WGIIFTALFAKEEYVNQV
YPGKPGRPYGLFMGGGGKLLGAHVIQILVIIGWVSATMGPLFFILNKMKLLRISTEDELAGMDL
TRHGGFAYAYEDDESH
KPGIQLRKIEPNSSSTPSAES*
>Lj4g0007055.1
MASPPLPVAYQAWTSLAVPDWLNKGDNAWQMIAATLVGIQSMPGLVILYGSIVKKKWAVNSA
FMALYAFAAVIICWVSWA
YKMSFGEKLLPFWGKAGPALGQRFLLKQAALPATPHYYNDGRLETAEIEPFYPMATMVWFQ
CVFAAIALVILAGSVLARM
SFKAWMMFVPLWLTFSYTIGAFSLWGGGFLFQWGVMDYSGGYVIHLSSGIAGFTAAYWVGP
RSKKDRERFPPNNVLLTLA

GAGLLWLGWAGFNGGDPYSANTDSSMAVLNTNICAATSLLVWTCLDVIFFKKPSVIGAVQGM
ITGLVCITPGAGLVQGWA
AIVMGVLSGSVPWYTMMVLGKKLKIFQMVDDTLAVFHTHAVAGILGGVLTGLFAEPRLSTLFL
PVTNSKGGVYGGSGGVQ
ILKQIVGALFIIGWNIVATSIICFVISFIVPLRMTEEELLIGDDAVHGEEAYALWGDGEKLSLYKDD
TTHHNVVMASSGA
TQDV*
>Medtr8g095040
MAAPPVNTLPVAYQAWTSLAVPDWLNKGDNSWQMISATLVGIQSMPGLVILYGSIVKKKW
AVNSAFMALYAFAAVIICWVTWAYKMSFGEKLLPFWGKAGPALGQKFLIRQAGLPATVHY
HHDGSLETAEIEPFYPMATMVWFQCVFAAISLVILAGSVLARMNFKAWMMFVPLWLTFSY
TIGAFSLWGGGFLFQWGVMDYSGGYVIHLSSGIAGFTAAYWVGPRSKKDRERFPPNNVLL
TLAGAGLLWMGWAGFNGGDPYSANIDSSMAVLNTNICAATSLLVWTWLDVIFFKKPSVIG
AVQGMITGLVCITPGAGLVQGWAAIVMGVLSGSVPWYTMMVLGKKISIFQKVDDTLAVFH
THAVAGLLGGVLTGLFAEPQLSTLFLPVTNSKGGVYGGSGGVQILKQIVGALFIVGWNIV
ATSIICLVISFIVPLRMTEEELLIGDDAVHGEEAYALWGDGEKLSLYKDDTTQHGMASSG
VTQVV
>Medtr8g074750
MNFNSSKYISHLPESLLPNDASPEWNNKADNAWQLTAATLVGLQTVPGLVILYGSMVKKK
WAVNSAFMALYAFAAVLVCWVLWAHHMAFGTKLLPFVGKPNFALSQKFLLSKASTNYYLP
MADFVFYQFAFAAITLVLLGGSLLGRMNFYAWMLFVPLWLTLSYTVGAFTIWGNGFLEGK
IIDYAGGFVIHLSSGVAGFTAAYWVGPRTSNDRQNFPPNNIIHMLGGAGFLWMGWTGFNG
GAPFQVGEITSLAIFNTHLCTATSILVWISLDMAVYKKGSLIGSVQGMMTGLVCITPGAG
LVDPWAAILMGALSGSIPWYTMMVLHKKSPFFQSVDDTLGVFHTHAVAGILGGILSGVFA
KPKLLRILYGPYGSGLLYSYFDDNIGQGIKQMWYQLLGAVFITIWNVVITSLICILLNRF
VNLRMQEEDLEVGDDAAHGEEAYVLWGDGERMRLPLRRDISPIIPYISHQRHSFPINKID
E
>Medtr1g036410
MSGVPFPSNLLPSPSSPEWLSKADNAWQLMAATLVGMQSVPGLIILYGGAVKKKWAVNSA
FMSLYAFACVFFCWVFWAYRMSFGDTLFPFWGKPALALDEKYLFKQAFLGAFPNATMIYF
QCVFAAITLILIAGAVLGRMNFYAWMMFVPLWLTFSYTFTAFSIWSTNGFLAKMGIIDYS
GGYVIHLSSGVAGFTAAYWVGPRLNKDRERFPPNNLLLMLAGAGLLWMGWTGFNGGDPYS
VGLDASLAVLNTHACTATSLLTWVFLDVIFFRKPSVIGAVQGMITGLVCITPAAGVVQGW
AALIMGLFSGSIPWFTMMVIHKRSKLLQKVDDTMAVLHTHAIAGSLGGILTGLFAEPKLN
SLFYGVYNKYVGLFYGIQMNMASTGRRQIGIQLLGILFVIFVNVVSTSLICLFIRLFVPL
RMSEEDMEIGDEAAHGEEAYAIWGQGDKHENSNSKYGSSLYEDVEVAATTKNKRGSQIEM
M
>Medtr1g045550
MSLPECSVSQLAQFIGPNATNAEAAANFICDQLTAVSNRFVDTQFAVDNTYLLFSAYLVF
SMQLGFAMLCAGSVRAKNTMNIMLTNVLDAAAGGVFYYLFGFAFAFGTPSNGFIGKHFFG
LKDVPTANFDYSYFLYQWAFAIAAAGITSGSIAERTQFVAYLIYSSFLTGFVYPVVSHWF
WSGDGWASATNTGNLLFGTGVIDFAGSGVVHMVGGIAGLWGALIEGPRIGRFDHSGRAVA
LRGHSASLVVLGTFMLWFGWYGFNPGSFTKILSPYNSGTYYGQWSAVGRTAVTTTLAGCT
AALTTLFGKRILSGHWNVTDVCNGLLGGFAAITAGCSVVEPWAAIVCGFIAAVVLIACNK

LAEKMRYDDPLEAAQLHGGCGAWGIIFTALFAKEEYVNQVYPGKLGRPYGLFMGGGGKLL
GAHVIQILVIIGWVSATMGPLFFVLNKLKLLRISTEDELAGMDLTRHGGFAYAYEDEGSH
RNGTQLRKIDHNSSSTPTPTTDL
>Medtr1g079760
MASLSCSATDFAPLLTTAANATAVADYLCTQFNTISNKLSDTTYAIDNTYLLFSAYLVFA
MQLGFAMLCAGSVRAKNTMNIMLTNVLDAAAGGLSYYLFGFAFAFGAPSNGFIGRHFFGL
RDYPSPSGDYSFFLYQWAFAIAAAGITSGSIAERTQFVAYLIYSSFLTGFVYPIVSHWVW
SADGWASPTRTDGDVLFGSGVIDFAGSGVVHMVGGIAGLWGAFIEGPRIGRFDRTGRSVA
LKGHSASLVVLGSFLLWFGWYGFNPGSFLTIVKSYGNMSNKRNYYGQWSAIGRTGVTTTL
AGCTAALTTLFSKRLLVGHWNVTDVCNGLLGGFAAITAGCSVVEPWAAIVCGFVAAWVLI
GLNKLAIKVKYDDPLEAAQLHGGCGAWGVLFTGLFAKGEYVDEIYGVGRPYGLLMGGGGR
LFAAQVVEVLVICGWVTVTMVPLFYGLNKMKLLRISRDDETAGMDLTRHGGFAYAYHDEE
DDESIRKLGFMMGRIEPATSVSPSAIGHDTPVATSAPPV
>Medtr3g463730
MEVTWEQSVTYSINTIYLLFSTYLVFVMQLGFAMICAGSVRAKNAMNIMLTNVVDAVVGS
ISFYLFGFAFAYGNSNPFIGTNLFALANIPNENYDYGFFLYQWAFAIAVAGITSGSIAER
TQFSAYLVFSFFLTGFVYPIVAHWVWSSSGWLSPNSSDLFLGSGAIDFAGSGVVHLVGAI
AGLWGSVIEGPRVGRFDAYGKPIPFRGHNATLVVLGTFLLWFGWFGFNPGSFDRILVAYP
GTSNEGHWTSVGRTAVTTTLAGSTAGIVTLFGRRLLIGHWNATDVCNGLLGGFVAITSGC
SVVEPWAAILCGFVAAWVLIGLNILALKLNYDDPLEAAQLHGGCGLWGLLFTGLFAKEQF
VIQTYNAGVAVKRPYGLLLGGGWGLIGAQVIEILVIFVFVSITMGPMFYGLHKLRLLRIP
VDDELAGLDISCHGGYAYAPQEENHPRSYGDYLRMQDDHS
>Medtr4g017820
MSLPTAYQEHLPSSPQWLNKGDNSWQMTASTLVGLQSMPGLVILYASMVKKKWAMNSAFM
ALYAYASVLICWVLLCYRMAFGDELFPFWGKGAPSLGQKFLTHQAKVPESTHFFKNGTEE
ASTTQPLFPMASLVYFQFNFAAITVILLAGSVLGRMNIRAWMAFVPLWLIFSYTVGAFSI
WGGGFLYHWGVIDFSGGYVIHLSSGIGGFTAAYWVGPRLKSDRERFPPNNVLLMLAGAGL
LWMGWSGFNGGAPYAANIDSSIAVLNTNICAATSLLVWTTLDVAFFGKPSVIGAVQGMMT
GLVCITPGAGLVQSWAAIVMGILSGSIPWWTMMVLHKKSTLLQKVDDTLGVFHTHAVAGL
LGGLLTGLLAEPDLCRLILPVTNSRGAFYGGGGGIQFLKQLVAALFITGWNLVSTTLILL
TIQLFIPLRMSNDQLETGDDAVHGEEAYALWGDGEKYDPTKHGSSSKADEIEVSPYVTGA
RGVTINL
>Medtr7g069640
MAASPAYQAHLPTSPEWLNKGDNAWQMTAATLVGLQSMPGLVILYASIVKKKWAVNSAFM
ALYAFAAVLICWVLVAYRMAFGDELLPFWGKGAPALGQKFLVARAKVPASTHYYKNGVDI
ETPMEEPFFPMASLVYFQFTFAAITMILLAGSVLGRMNIKAWMAFVPLWLIFSYTVGAFS
LWGGGFLYHWGVIDYSGGYVIHLSSGIAGFTAAYWVGPRIKSDRERFPPNNVLLMLAGAG
LLWMGWSGFNGGAPYAANVASSIAVLNTNICAATSLLVWTSLDVVFFGKPSVIGAVQGMM
TGLVCITPGAGLVQSWAAIVMGILSGSIPWVSMMILHKKSSLLQKVDDTLGVFHTHAVAG
LLGGLLTGLFAEPALCRLLLPVTNSRGAFYGGSGGMQLLKQLVAAMFVIGWNIVSTTIIL
LVIQLFIPLRMPDEQLEIGDDAAHGEEAYALWGDGEKYDPTRHGSLNTGNTGVSPYVNGA
RGVTINL
>Medtr7g113340
MATFTCSASDLHALLGGSANSTAAAEYICGGFNAVANKFIDTSYAVDNTYLLFSAYLVFA

MQLGFAMLCAGSVRAKNTMNIMLTNVLDAAIGGIFFYVFGFALAFGTPSNGFIGKHFFGL
TDFPSQSFDYGFFLFQWAFAIAAAGITSGSIAERTQFVSYLIYSSFLTGLVYPIVAHWFW
SADGWGSPVRSENLLFGSGVIDFAGCGVVHLVGAVAGFWGAFIEGPRIGRFDHEGKSSPI
RGHSGTLVVMGTFLLWFGWYGFNPGSFLNIVKAYGESGHYYGQWSAIGRTAVTTTLAGCT
AALTTLFGKRMQTGHWSVSDVCNGLLGGFAAITAGCSVVDPWAAIICGFVAAWVLIGCNL
LAEKFHYDDPLEAAQLHGGCGTWGIIFTALFAKKQYVNEVYSGSPDRTYGLLLGGGGRLL
AAHVVQILAIVAWVSITMGTMFFLLHKLNLLRSSPEEEMEGLDLTSHGGLAYEYHQEAEE
HAKRRAFEV
>Medtr7g115050
MELPSNLLPDEASPEWMNKGDNAWQLTAATMVGLQSIPGLVILYGSLVKKTWAINSAFMA
FYAFASVLLCWVSWAYQMSFGEKMVFFLGKPNVALDEKFLLGKAFLGNFPNATMVFYQGV
FAGLTLILIAGALLGRMNIRAWMLFVPLWVTFSYTVVAFSIWCPDGWLAKRGVIDFAGGY
VIHLSAGVAGFTAAYWVGPRADKDRETFPAATNNMIMVLAGAGLLWMGWSGFNGGAPFVA
STIASLAILNTHVCTAASITVWVMLDTFYFGKPTVFGAVQGMITGLVCITPAAGVVQGWA
AILMGFISGSIPWYTMMVLHNKVNFLKKIDDPMAVFHTHAIAGALGGILTGFFAVPKLCR
LFYMVPDWEKYIGLAYGLQNKGATQAGLKQMVIQIEAIVFVICYNVLMTSLICLIVRVIV
PLRLNGDALQMGDKAIHGEDAFALHSEATKFVNIKRNQVYDTQDFSSIPESRSLGELQMV
>Medtr7g098930
MASFTCSASDLNTLLGSGANSTAAAEYICNGFNAVANKFIDTTYAVDNTYLLFSSYLVFA
MQLGFAMLCAGSVRAKNTMNIMLTNVLDAATGGIFFYIFGFAFAFGTPSNGFIGKHFFGL
SDFPSQSFDYGYFLYQWAFAIASAGITSGSIAERTQFVSYLIYSSFLTGLVYPIVAHWFW
SADGWGSPVRSENLLFGSGVIDFAGCGVVHLVGAVAGFWGALIEGPRIGRFDHEGKGVSM
RGHSGTLVVMGTFLLWFGWYGFNPGSFLNILKIYGESGNYYGQWSAIGRTAVTTTLAGCT
AALTTLFGKRLQTGHWNVTDVCNGLLGGFAAITAGCSVVDPWAAIICGFIAAWVLIGCNM
LAEKFQYDDPLEAAQLHGGCGTWGIIFTALFAKKQYVKEVYGGSPDRPYGLLLGGGGRLL
AAHLVQILAIVVWVSVTMGTLFFILHKLNLLRSSHEEEMSGLDLTSHGGLAYEYHEELEE
RRPKKRGIEI
>OsAMT1.1(LOC_Os04g43070)
MATCAADLAPLLGPVAANATDYLCNRFADTTSAVDATYLLFSAYLVFAMQLGFAMLCAGSVRA
KNTMNIMLTNVLDAAAG
ALFYYLFGFAFAFGTPSNGFIGKQFFGLKHMPQTGFDYDFFLFQWAFAIAAAGITSGSIAERT
QFVAYLIYSAFLTGFVY
PVVSHWIWSADGWASASRTSGPLLFGSGVIDFAGSGVVHMVGGVAGLWGALIEGPRIGRFD
HAGRSVALKGHSASLVVLG
TFLLWFGWYGFNPGSFTTILKTYGPAGGINGQWSGVGRTAVTTTLAGSVAALTTLFGKRLQT
GHWNVVDVCNGLLGGFAA
ITAGCSVVDPWAAIICGFVSAWVLIGLNALAARLKFDDPLEAAQLHGGCGAWGILFTALFARQ
KYVEEIYGAGRPYGLFM
GGGGKLLAAHVIQILVIFGWVSCTMGPLFYGLKKLGLLRISAEDETSGMDLTRHGGFAYVYHD
EDEHDKSGVGGFMLRSA
QTRVEPAAAAASNSNNQV*
>OsAMT2.2(LOC_Os01g61550)
MHLRMASPPQPGPYMPDLPAVPAWLNKGDTAWQLVAATFVGIQSMPGLVVIYGSIVKKKWAV
NSAFMALYAYASTLIVWV

LVGFRMAFGDRLLPFWAKAGPALTQDFLVQRAVFPATAHYGSDGTLETPRTEPFYAEAALVLF
EFEFAAITLVLLAGSLL
GRMNIKAWMAFTPLWLLFSYTVGAFSLWGGGFLYQWGVIDYSGGYVIHLSSGVAGFTAAYW
VGPRLKSDRERFSPNNILL
MIAGGGLLWLGWAGFNGGAPYAPNVTATVAVLNTNVSAATSLLTWTCLDVIFFGKPSVIGAVQ
GMMTGLVCITPGAGLVH
TWSAMLMGMFAGSVPWFTMMILHKKSTFLMKVDDTLAVFHTHAVAGILGGVLTGLLATPELC
ALDCPIPNMRGVFYGSGI
GQLGKQLGGALFVTVWNLIVTSAILLCIGLFIPLRMSDDQLMIGDDAAHGEEAYALWGDGEKF
DVTRPETTRTGGAGGAG
REDTMEQRLTNMGARGVTIQL*
>OsAMT3.1(LOC_Os01g65000)
MSGDAFNMSVAYQPSGMAVPEWLNKGDNAWQMISATLVGMQSVPGLVILYGSIVKKKWAVN
SAFMALYAFAAVWLCWVTW
GYNMSFGHKLLPFWGKARPALGQSFLLAQAVLPQTTQFYKGGGGADAVVETPWVNPLYPM
ATMVYFQCVFAAITLILLAG
SLLGRMNIKAWMLFVPLWLTFSYTVGAFSLWGGGFLFHWGVMDYSGGYVIHLSSGVAGFTA
AYWVGPRSTKDRERFPPNN
VLLMLTGAGILWMGWAGFNGGDPYSANIDSSLAVLNTNICAATSLLVWTCLDVIFFKKPSVIGA
VQGMITGLVCITPGAG
LVQGWAAIVMGILSGSIPWFTMMVVHKRSRLLQQVDDTLGVFHTHAVAGFLGGATTGLFAEP
VLCSLFLPVTNSRGAFYP
GRGGGLQFVRQVAGALFIICWNVVVTSLVCLAVRAVVPLRMPEEELAIGDDAVHGEEAYALW
GDGEKYDSTKHGWYSDNN
DTHHNNNKAAPSGVTQNV*
>OsAMT2.3(LOC_Os01g61510)
MASPTRPGPYMPRPPAVPEWLNTGDNGWQLAAATFVGLQSMPGLVVLYGSIVKKKWAVNS
AFMALYAYASTLIVWVLVGF
RMAFGDRLLPFWGKAGAALTEGFLVARASVPATAHYGKDGALESPRTEPFYPEASMVLFQFE
LAAITLVLLAGSLLGRMN
IKAWMAFTPLWLLFSYTVCAFSLWGGGFLYQWGVIDYSGGYVIHLSSGIAGFTAAYWVGPRL
KSDRERFSPNNILLMIAG
GGLLWLGWAGFNGGAPYAPNITASIAVLNTNVSAAASLLTWTCLDVIFFGKPSVIGAVQGMMT
GLVCITPGAGLVHTWAA
ILMGICGGSLPWFSMMILHKRSALLQKVDDTLAVFHTHAVAGLLGGFLTGLFALPDLTAVHTHI
PGARGAFYGGGIAQVG
KQIAGALFVVVWNVVATTVILLGVGLVVPLRMPDEQLKIGDDAAHGEEAYALWGDGERFDVT
RHEGARGGAWGAAVVDEA
MDHRLAGMGARGVTIQL*
>OsAMT3.2(LOC_Os03g62200)
MSSSATVVPLAYQGNTSASVADWLNKGDNAWQLVAATVVGLQSVPGLVVLYGGVVKKKWAV
NSAFMALYAFAAVWICWVT
WAYNMSFGEKLLPIWGKARPALDQGLLVGRAALPATVHYRADGSVETAAVEPLYPMATVVYF
QCVFAAITLILVAGSLLG

RMSFLAWMIFVPLWLTFSYTVGAFSLWGGGFLFHWGVIDYCGGYVIHVSAGIAGFTAAYWVG
PRAQKDRERFPPNNILFT
LTGAGLLWMGWAGFNGGGPYAANSVASMAVLNTNICTAMSLIVWTCLDVIFFKKPSVVGAVQ
GMITGLVCITPAAGVVQG
WAALVMGVLAGSIPWYTMMILHKRSKILQRVDDTLGVFHTHGVAGLLGGLLTGLFAEPTLCNL
FLPVADSRGAFYGGAGG
AQFGKQIAGGLFVVAWNVAVTSLICLAINLLVPLRMPDDKLEVGDDAVHGEEAYALWGDGEM
YDVTKHGSDAAVAPVVV*
>OsAMT4.1(LOC_Os03g53780)
MAAEAAPEWVEKGDNAWPLAAATLVGLQSVPRLVILYGDCGAVGPRTEKDREAFPPNNVLLT
LAGAGLLLWMGWTGFNGG
APYAANVDASVTVVNTHLCTATSLLVWLLLDSFVFGRLSVISAVQGMITGLVCVTPAARLVLHK
RSRLLARVDDTLAVLH
THGVAGSLSGVLTGLLLLAEPRFARLFFGDDPRYVGLAYAVRDGRAGSGLRQVGVQLAGIAF
VVALNVAVTSAVCLAVRV
AVPQLAGGGDAIHGEDAYAVWGDGETYEQYSVHGGGSNHGGFPMTANPVASKADEMIWI*
>OsAMT3.3(LOC_Os02g34580)
MAAGAIPMAYQTTPSSPDWLNKGDNAWQMTSATLVGLQSMPGLVILYGSIVKKKWAINSAFM
ALYAFAAVWICWVVWAYN
MSFGDRLLPFWGKARPALGQSFLVAQSELTATAIRYHNGSAEAPMLKPLYPVATMVYFQCMF
ASITIIILAGSLLGRMNI
KAWMAFVPLWITFSYTVCAFSLWGGGFLFQWGVIDYSGGYVIHLSSGIAGLTAAYWVGPRSA
SDRERFPPNNILLVLAGA
GLLWLGWTGFNGGDPYSANIDSSMAVLNTHICASTSLLVWTILDVFFFGKPSVIGAVQGMITG
LVCITPGAGLVQGWAAI
VMGILSGSIPWYTMMVLHKKWSFMQRIDDTLGVFHTHAVAGFLGGATTGLFAEPILCSLFLSIP
DSKGAFYGGPGGSQFG
KQIAGALFVTAWNIVITSIICVIISLILPLRIADQELLIGDDAVHGEEAYAIWAEGELNDMTHHNES
THSGVSVGVTQNV
*
>OsAMT1.3(LOC_Os02g40710)
MATCLDSLGPLLGGAANSTDAANYICNRFTDTSSAVDATYLLFSAYLVFAMQLGFAMLCAGSV
RAKNSMNIMLTNVLDAA
AGALFYYLFGFAFAFGTPSKGFIGKQFFGLKHMPQTGYDYDFFLFQWAFAIAAAGITSGSIAE
RTRFSAYLIYSAFLTGF
VYPVVSHWFWSTDGWASAGRLTGPLLFKSGVIDFAGSGVVHLVGGIAGLWGAFIEGPRIGRF
DAAGRTVAMKGHSASLVV
LGTFLLWFGWFGFNPGSFTTISKIYGESGTIDGQWSAVGRTAVTTSLAGSVAALTTLYGKRWL
TGHWNVTDVCNGLLGGF
AAITAGCSVVDPWASVICGFVSAWVLIGCNKLSLILKFDDPLEATQLHAGCGAWGIIFTALFAR
REYVELIYGVPGRPYG
LFMGGGGRLLAAHIVQILVIVGWVSATMGTLFYVLHRFGLLRVSPATEMEGMDPTCHGGFGY
VDEDEGERRVRAKSAAET
ARVEPRKSPEQAAAGQFV*

>OsAMT1.2(LOC_Os02g40730)
MATCADTLGPLLGTAAANATDYLCNQFADTTSAVDSTYLLFSAYLVFAMQLGFAMLCAGSVRA
KNTMNIMLTNVLDAAAG
ALFYYLFGFAFAFGAPSNGFIGKHFFGLKQVPQVGFDYSFFLFQWAFAIAAAGITSGSIAERTQ
FVAYLIYSAFLTGFVY
PVVSHWIWSADGWASASRTSGSLLFGSGVIDFAGSGVVHMVGGVAGLWGALIEGPRIGRFD
HAGRSVALRGHSASLVVLG
SFLLWFGWYGFNPGSFLTILKSYGPPGSIHGQWSAVGRTAVTTTLAGSTAALTTLFGKRLQTG
HWNVIDVCNGLLGGFAA
ITAGCSVVDPWAAIICGFVSAWVLIGLNALAARLKFDDPLEAAQLHGGCGAWGVIFTALFARK
EYVDQIFGQPGRPYGLF
MGGGGRLLGAHIVVILVIAAWVSFTMAPLFLVLNKLGLLRISAEDEMAGMDQTRHGGFAYAYH
DDDASGKPDRSVGGFML
KSAHGTQVAAEMGGHV*
>LOC_Os12g01420
MASVAVPEWLNKGDNAWQMLSATLVALQGFPGLALFYAGAVTRKCALTSAFMALYAMAATM
PCWALWAHNMAFGHRLLPF
VGRPAPALAQHYMLTQALLPFTLHLHSNGEVETAAVAPLYPSASMVFFQWASAGVTVGLVAG
AVLGRMSVKAWMAFVPLW
TTLSYTVGAYSIWGGGFLFHWGVMDYSGGYVVHLAAGVSGYTAAYWVGPRRKEEEEMTMA
GGGNLVAMVAGAGILWMGWT
GFNGGDPFSANTDSSVAVLNTHICTTTSILAWVCCDIAVRGRPSVVGAVQGMITGLVCITPAA
GLVQGWAALLMGVASGT
LPCYTMNAAMSFKVDDTLGILHTHAVSGVLGGVLTGVFAHPTLCDMFLPVTGSRGLVYGVRA
GGVQVLKQVAAALFVAAW
NVAATSIILVVVRAFVPLRMTEDELLAGDIAVHGEQAYYFSSGTNCSLSHETIEVGNS*
>OsAMT2.1(LOC_Os05g39240)
MAAAGAYSASLPAVPDWLNKGDNAWQLTASTLVGIQSMPGLVVLYGSIVKKKWAVNSAFMAL
YAYASSLLVWVLVGFRMA
FGDQLLPFWGKAGVALTQSYLVGRATLPATAHGAIPRTEPFYPEATLVLFQFEFAAITLVLLAGS
VLGRMNIKAWMAFTP
LWLLLSYTVGAFSLWGGGFLYRWGVIDYSGGYVIHLSSGIAGFTAAYWVGPRLKSDRERFSP
NNILLMIAGGGLLWMGWA
GFNGGAPYAANIAASVAVLNTNVCAATSLLMWTCLDVIFFRKPSVIGAVQGMMTGLVCITPGA
GLVQTWAAVVMGIFAGS
VPWFTMMILHKKSALLMKVDDTLAVFHTHAVAGLLGGILTGLLATPELFSLESTVPGLRGAFYG
GGIKQIGKQLGGAAFV
IAWNLVVTTAILLGIGLFIPLRMPDEQLMIGDDAAHGEEAYALWGDGEKFDATRHDLSRGGGG
GDRDGPAGERLSALGAR
GVTIQL*
>LOC_Os11g01410
MASAAVPEWLNKGDNAWQMLSATLVALQGFPGLALFYVGAVPRKWALTSAFMALYAMAATM
PCWALWAHNMAFGRRLLPF
VGRPAPALAQDYMLSQALLPSTLHLRSNGEVETAAVAPLYPSASMVFFQWAFAGVTVGLVAG

AVLGRMSVKAWMAFVPLW
TTLSYTVGAYSIWGGGFLFHWGVMDYSGGYVVLLAAGVSGYTAAYWVGPRRKEEDEEEMA
TASGGNLVVMVAGAGILWMG
WTGFNGGDPFSANTDSSVAVLNTHICATTSIVAWVCCDVAVRGRPSVVGAVQGMITGLVCITP
RSNIKYSFLLVVISDEM
PVPDLS*
>GmAMT4.2(Glyma.20G004100)
MSLYAFTCVFFCWVVWGYRMSFGDELLPFWGKPAMSLEHAYLFKKAFLGAFPNATMCVFA
AITLILIAGAVLGHMNFYAWMLPWPFSTPTLAHLVILDVLFFRKPSVIGAVQGMITGLVC
ITPAAGVVQGWASLIMGLLSGSIPWFTMMVIHKRSKLLQKVDDTMAVFHTHAIAETLGGL
LTGLFADPRLNRLFYGHDAQYVVFFYGFGSNKLSTGFRQMGVQILGIIFVIFVNFIRIFF
PLRISEEDMEIGDEAAHGEEAYAIWGQGDKLENSSSKYGSSLYNDVEAGAPKKKRAGTVQ
MMSN*
>GmAMT1.1(Glyma.20G082450)
MSLPDCPAVQLAQLLGPNTTNAAAAASFICDRFTAVDNKFVDTAFAVDNTYLLFSAYLVF
SMQLGFAMLCAGSVRAKNTMNIMLTNVLDAAAGGLFYYLFGFAFAFGSPSNGFIGKHFFG
LKELPSQSFDYSNFLYQWAFAIAAAGITSGSIAERTQFVAYLIYSSFLTGFVYPVVSHWF
WSADGWASAISPGDRLFSTGVIDFAGSGVVHMVGGVAGFWGALIEGPRIGRFDHAGRAVA
LRGHSASLVVLGTFMLWFGWYGFNPGSFNKILVSYGNSGTYYGQWSAVGRTAVTTTLAGS
TAALTTLFGKRMISGHWNVTDVCNGLLGGFAAITAGCSVVEPWAAIVCGFVASVVLIACN
KLAEKVKFDDPLEAAQLHGGCGAWGVIFTALFAKKEYVSQVYGEGRAHGLFMRGGGKLLA
AHVIQILVIVGWVSATMGPLFWGLNKLKLLRISSEDELAGMDLTRHGGFAYAYEDDESHK
HGIQLRKVGPNASSTPTTDE*
>GmAMT1.6(Glyma.20G221401)
MLCAGSVRAKNTMNIMLTKVLDAAAGGLSYYLFGFAFAFGALSNGFIGRHFFGLRDFPMG
ASPSGDYSFFLYQWAFAIAAAGITSGTIAERTQFVAYLIYSSFLTGLVYPIVSHWFWSSD
GWASAARSNGNVLFGSGVIDFAGSGVVHMVGGIAGMWGALIEGPRIGRFDRTDRSVALRG
HSASLVVLGSFLLWFGWYGFNPGSFITIAKGIMVNGAR*
>GmAMT4.4(Glyma.02G043700)
MSSSSYDIPLPSSLLPSEASPEWNNKADNAWQLTAATLVGLQSVPGLVILYGSMVKRKWA
VNSAFMALYAFACVLICWVSWAHRMAFGARLLPFVGAPNHALAEKFLLAKSTIGYFPMAD
FVFYQFAFAAITLVLLAGSLLGRMNFYAWMMFVPLWLTLSYTVGAFSIWDDDGFLQGKII
DYAGGFVIHLSSGVAGFTAAYWVGPRISQDRQNFPPNNIIHVLGGAGFLWMGWTGFNGGA
PFQVGEIASLAIYNTHLCTATSLLVWLTLDMIVYTKSSVIGAVQGMITGLVCITPGAGLV
DPWAAVLMGALSGSIPWYTMMVLHKKSAFFQSVDDTLGVFHTHAVAGLLGGILSGVFAKP
DLLRIMYQDTNYCPGLFYTFFKGEVDHGFRQIWMKEEDLEVGDDAAHGEEAYALWGDGER
MRIPLRLHIGPTIPSLCRKRFSIPLTRKEGE*
>GmAMT4.5(Glyma.02G143600)
MSHLPSNLLPVDGSPEWMNRGDNTWQLTSATLVGLQSVPGLVILYGSIVKKKWALNSAFM
ALYAFAAVLVCWVGWCYKMSFGERILPFLGQPNTALDREFVFRKTFAGEFPNATMVYFQL
VFAAITLILIAGALLGRMNFYAWMLFVPLWVTFSYTICAFSIWSTDGWLFKMGLIDYSGG
FVIHLSSGVAGFVAACWVGPRAVKDRERFPPNNILLMLAGAGLLWMGWSGFNGGDPYAAS
IDASLAVLNTHVCTAMSLLTWLFLDILFFGKPSVIGATQGMITGLVCITPAAGTICPRVV
QGWAAIIMGMMSGSIPWYTMMVLHKESKLLKQVDDTLAVFHTHAVAGSLGGILAGFFADP

KLCYLFYGVEDSLHYTGLVYGIFNGRPNAGFRQMMVQLLGIVFVILLNVTTTSIVCLLVR
IIVPLRLSEDDLQVGDEAVHGEVAYALWGDGEKLETATEPLTSEKNLEWPTVELRT*
>GmAMT1.2(Glyma.10G132300)
MSLPACPAEQLAQLLGPNTTDASAAASLICGQFAAVDSKFVDTAFAVDNTYLLFSAYLVF
SMQLGFAMLCAGSVRAKNTMNIMLTNVLDAAAGGLFYYLFGFAFAFGSPSNGFIGKHFFG
LKDIPSSSYDYSYFLYQWAFAIAAAGITSGSIAERTQFVAYLIYSSFLTGFVYPVVSHWF
WSPDGWASAFKITDRLFSTGVIDFAGSGVVHMVGGIAGLWGALIEGPRMGRFDHAGRAVA
LRGHSASLVVLGTFLLWFGWYGFNPGSFNKILLTYGNSGNYYGQWSAVGRTAVTTTLAGS
TAALTTLFGKRVISGHWNVTDVCNGLLGGFAAITAGCSVVEPWAAIVCGFVASIVLIACN
KLAEKVKFDDPLEAAQLHGGCGTWGVIFTALFAKKEYVKEVYGLGRAHGLLMGGGGKLLA
AHVIQILVIAGWVSATMGPLFWGLNKLKLLRISSEDELAGMDMTRHGGFAYAYEDDETHK
HGMQLRRVGPNASSTPTTDE*
>GmAMT1.5(Glyma.10G168200)
MASLSCSANDLAPLINDTAAANYLCAQFDSISRKLVETTYAVDNTYLLFSAYLVFAMQLG
FAMLCAGSVRAKNTMNIMLTNVLDAVAGGLSYYLFGFAFAFGGPSNGFIGRPNFRLRDYP
MGSSISSFFLYQLVLAIADAGITISPFGDYSFFLYQWAFAIAAAGITSGSIAERTQFVAY
LIYSSFLTGFVYPIVSHWFWSSDGWASATRSHGNVLFGSGVIDFAGSGVVHMVGGIAGLW
GALIEGPRIGRFDRSGRSVALRGHSASLVVLGTFLLWFGWYGFNPGSFVTIDKGYESGGY
YGQWSAIGRTAVTTTLAGSTAALTTLFSKRLLVGHWNVIDVCNGLLGGFAAITSGCAVVE
PWAAIVCGFVAAWVLIWLNKLAAKVEYDDPLEAAQLHGGCGAWGVFFTGLFAKKVYVEEI
YGVGRPFGALMGGGGRLLAAQVIQILVVCGWVTATMAPLFYGLHKMKLLRISRDDETAGM
DLTRHGGFAYAYHDDEDGSSRGVGFMMLRIEPAGTSPSPPAAPQV*
>GmAMT1.3(Glyma.10G167800)
MASLSCSANDLAPLFNDTAAANYLCAQFDSISRKLAETTYAVDNTYLLFSAYLVFAMQLG
FAMLCAGSVRAKNTMNIMLTNVLDAAAGGLSYYLFGFAFAFGGPSNGFIGRHFFGLRDYP
MGSSPSGDYSFFLYQWAFAIAAAGITSGSIAERTQFVAYLIYSSFLTGFVYPIVSHWFWS
SDGWASASRSDGNVLFGSGVIDFAGSGVVHMVGGIAGLWGALIEGPRIGRFDRSGRSVAL
RGHSASLVVLGSFLLWFGWYGFNPGSFLTIAKGYGSGGYYGQWSAIGRTAVTTTLAGSTA
ALTTLFSKRLLAGHWNVIDVCNGLLGGFAAITSGCAVVEPWAAIVCGFVAAWVLIGLNKL
AAKVEYDDPLEAAQLHGGCGAWGVFFTGLFAKKVYVEEIYGVGRPFGALMGGGGRLLAAQ
VIQILVVCGWVTATMAPLFYGLHKMKLLRISRDDETAGMDLTRHGGFAYAYHDDEDGSSR
GVGFMLRRIEPAASTTPSPPAAPQV*
>GmAMT1.4(Glyma.10G168100)
MASLSCSANDLAPLFNDTAAANYLCAQFDSISRKLNVTTYAVDNTYLLFSAYLVFAMQLG
FAMLCAGSVRAKNTMNIMLTNVLDAAAGGLSYYLFGFAFAFGGPSNGFIGRHFFGLRKFP
KDSPPSGDYSFFLYHWAFAIAAAGITSGSIAERTQFVAYLIYSSFLTGFVYPIVSHWFWS
SDGWASATRSHGNVLFGSGVIDFAGSGVVHMVGGIAGLWGALIEGPRIGRFDRSGRSVAL
RGHSASLVVLGTFLLWFGWYGFNPGSFLTIAKGYESGGYYGQWSAIGRTAVTTTLAGSTA
ALTTLFSKRLLAGHWNVIDVCNGLLGGFAAITSGCAVVEPWAAIVCGFVAAWVLIGLNKL
AAKVEYDDPLEAAQLHGGCGAWGVFFTGLFAKKEYVEEIYGGGRPFGALMGGGGRLLAAQ
VIQILVVCGWVTATMAPLFYGLHKMKLLRISRDDETAGMDLTRHGGFAYAYHDDEDGSSR
GVGFMLRRIEPAAITTPSPPAAPQV*
>GmAMT4.6(Glyma.10G030800)
MAYHGFNGGDPYAASIDASLAVLNTHVCTAMSLLTWLFLDILFFGKPSVIGATQGMITGL

VCNTPAAGVVQGWAAIIMGMMSGSIPWYTMMVLHKESKLLKQVDDTSAVFHTHAVAGSLG
GILAGFFADPNLCYLFYGVQDSLHYTGLVYGIFNGNLKTGFRQMGVQLLGIVFVILLNVT
TTSIVCLLVRMIVPLRMSEDDLQVGDEAVHGEVAYALWGDGEKLETAAGEALTSEKNHEL
PTVELIKSLSDAPLID*
>GmAMT2.3(Glyma.01G123400)
MATPTAYQEHLPASPHWLNKGDNAWQLTAATLVGLQSMPGLVILYASMVKKKWAVNSAFM
ALYAFAAVLICWVLVCHRMAFGDKLLPFWGKGAPALGQKFLTHRAKVPESTHYYNNGTVE
SATSEPLFATASLVYFQFTFAAITLILLAGSVLGRMNIKAWMAFVPLWLIFSYTVGAFSL
WGGGFLYHWGVIDYSGGYVIHLSSGIAGFTAAYWVGPRLKSDRERFPPNNVLLMLAGAGL
LWMGWSGFNGGAPYAANIDSSIAVLNTNICAATSLLVWTSLDVIFFGKPSVIGAVQGMMT
GLVCITPGAGLVQSWAAIVMGILSGSIPWVTMMILHKKSSLLQKVDDTLGVFHTHAVAGL
LGGLLTGLLAEPQLCRLILPVTNSRGAFYGGSGGIQLFKQLVAACFIAGWNLVSTTLILL
TIQLFIPLRMPTEQLEIGDDAIHGEEAYALWGDGEKYDPTRHGSSRVEKTSASPRVNVTG
ARGVTIDL*
>GmAMT2.1(Glyma.07G153800)
MATPLAYQEHLPAAPEWLNKGDNAWQLTAATLVGLQSMPGLVILYASIVKKKWAVNSAFM
ALYAFAAVLICWVLVCYRMAFGEELFPFWGKGAPALGQKFLTKRAIVIETIHHFDNGTVE
SPPEEPFYPMASLVYFQFTFAAITLILLAGSVLGRMNIKAWMAFVPLWLIFSYTVGAFSL
WGGGFLYQWGVIDYSGGYVIHLSSGIAGFTAAYWVGPRLKSDRERFPPNNVLLMLAGAGL
LWMGWSGFNGGAPYAANIASSIAVLNTNICAATSLLVWTTLDVIFFGKPSVIGAVQGMMT
GLVCITPGAGLVQSWAAIVMGILSGSIPWVTMMILHKKSTLLQKVDDTLGVFHTHAVAGL
LGGLLTGLLAEPALCRLLLPVTNSRGAFYGGGGGVQFFKQLVAAMFVIGWNLVSTTIILL
VIKLFIPLRMPDEQLEIGDDAVHGEEAYALWGDGEKYDPTRHGSLQSGNTTVSPYVNGAR
GVTINL*
>GmAMT4.1(Glyma.09G281600)
MLAGAGLLWMGWTGFNGGDPYTVNSDASLAVLNTHACTATSLLTWVILDVLFFRKPSVIG
AVQGMITGLVCITPAAGVVEGWAALIMGVLSGSIPWFTMMVVHKRSKLLQKVDDTMAVFH
THAIAGTLGGLLTGLFADPRLNKLFYGTDGQYVGFFYGLGSKKVSTGFRQMGVQILGIIF
VIFVNVISTSLICLFIRIFVPLRMSEEDMEIGDEAAHGEEAYAIWGQGDKLENSSSKYGS
SLYDDVEAGGPKKKRGGTVEMMSN*
>GmAMT3.1(Glyma.05G196500)
MATPPVNSLPIAYQAWTSLGVPDWLNKGDNAWQMVSATLVGIQSMPGLVILYGSIVKKKW
AVNSAFMALYAFAAVIICWVAWAYKMSFGEELLPFWGKAGPALGQRFLIKQAGLPATPHY
FRNGGGLETAEITPFYPMGTMVWFQCVFAAIAVVILAGSVLARMNFKAWMMFVPLWLTFS
YTIGAFSLWGGGFLFHWGVMDYSGGYVIHLSSGIAGFTAAYWVGPRSKKDRERFPPNNVL
LTLAGAGLLWMGWAGFNGGDPYAANTDSSMAVLNTNICAATSLLVWTWLDVIFFKKPSVI
GAVQGMITGLVCITPGAGLVQGWAAIVMGVLSGSVPWFSMMVLGKKLKLFQMVDDTLAVF
HTHAVAGLLGGILTGLFAEPRLCALFLPVTNSKGGVYGGPGGVQILKQIVGALFIIGWNL
VVTSIICVVISFIVPLRMTEEELLIGDDAVHGEEAYALWGDGEKLSIYKDDTTHHGVVSS
GATQVV*
>GmAMT4.3(Glyma.19G244400)
MEFPTNLLPDEASPEWMNKGDNAWQLMAATVVGLQSIPGLVILYGSLVKKTWAINSAFMA
FYAFAGVLLCWVGWGFRMSFGEKMVFFLGKPGVAVDEKFLLGKAFLGLFPNATMVFFQGV
FAGITLILIAGALLGRMNFRAWMLFVPLWLTFSYTVTAFSIWCPDGWLAKLGVIDFSGGY

VIHLSAGVAGFTAAYWVGPRSEKDREIFASNNMIVVLAGAGLLWMGWSGFNGGGPFVAST
VASLAVLNTHVCAAASIIVWVLLDTFYFGKPTVFGAVQGMITGLVCITPAAGVVQGWAAI
LMGVMSGSIPWYTMMILHDKLPFLKQIDDPMAVFHTHAVAGALGGVLTGLLAVPKLCRLF
YMVPDWEKYIGLAYGLQNGATHAGLRQMAIQVGAIVFVIIFNFVTTSLICLLVGSIVPLR
IDTDALQMGDKAMHGEEAFAFYGGDPTAKFENLKHNKVYDTQDFSFVRDTRSFAQLQMV*
>GmAMT2.2(Glyma.18G204800)
MATPLAYQEHLPAAPSWLNKGDNAWQLTAATLVGLQSMPGLVILYASIVKKKWAVNSAFM
ALYAFAAVLICWVLVCYRMAFGEELLPFWGKGAPALGQKFLTKRAVVNETIHHFHNGTVE
SPPEEPFYPMASLVYFQFTFAAITLILLAGSVLGRMNIKAWMAFVPLWLIFSYTVGAFSL
WGGGFLYQWGVIDYSGGYVIHLSSGIAGLTAAYWVGPRLKSDRERFPPNNVLLMLAGAGL
LWMGWSGFNGGAPYAANIASSIAVLNTNICAATSLLVWTTLDVIFFGKPSVIGAVQGMMT
GLVCITPGAGLVQSWAAILMGILSGSIPWVTMMILHKKSTLLQKVDDTLGVFHTHAVAGL
LGGLLTGLLAEPALCRLLLPVTNSRGAFYGGGGGMQFFKQLVAAMFVIGWNLVSTTIILL
VIKLFIPLRMPDEQLEIGDDAVHGEEAYALWGDGEKYDPTRHGSLQSGNTFVSPYVNGAR
GVTINL*
