# Supplementary material for: Genome-Wide Identification and Expression Analysis of the Ammonium Transporter Family Genes in Soybean
Source: Int J Mol Sci. 2023 Feb 16;24(4):3991. doi: 10.3390/ijms24043991 (PMC9960152; doi:10.3390/ijms24043991)
Supplement: Supplementary file 1 [file ijms-24-03991-s001.zip › Supplementary Data2.pdf]

Supplementary Data 2.

The CDS sequences of *AMTs* in Arabidopsis and soybean.

>AtAMT2
ATGGCCGGAGCTTACGATCCAAGCTTGCCGGAGGTTCCTGAATGGCTCAACAAAGGAGA
C
AACGCGTGGCAGCTCACGGCAGCGACTCTGGTTGGTCTACAGAGTATGCCAGGTCTTGT
T
ATCCTCTATGCCTCCATCGTCAAGAAGAAATGGGCTGTGAATTCAGCTTTTATGGCTCTT
TACGCTTTCGCCGCCGTTCTTCTCTGTTGGGTTCTCCTCTGTTACAAAATGGCTTTTGGA
GAAGAGCTTTTGCCGTTTTGGGGCAAAGGTGGTCCAGCTTTCGACCAAGGATACCTTAA
G
GGACAAGCAAAGATCCCAAATAGTAATGTGGCGGCGCCGTATTTTCCGATGGCGACGTTG
GTGTATTTTCAGTTCACATTCGCGGCGATAACGACGATACTTGTGGCGGGATCTGTGTTG
GGGAGGATGAATATTAAAGCATGGATGGCTTTTGTGCCATTGTGGTTGATCTTTAGCTAC
ACAGTTGGAGCTTATAGTATATGGGGAGGTGGGTTTCTGTATCAGTGGGGAGTTATTGAT
TATTCCGGCGGTTATGTTATTCATCTCTCCTCCGGTGTTGCCGGTTTCGTCGCTGCTTAC
TGGGTAGGACCAAGGCCTAAGGCTGACAGAGAGAGATTCCCACCGAACAATGTTCTTCTA
ATGCTTGCTGGAGCTGGACTTTTATGGATGGGATGGTCCGGTTTTAACGGTGGTGCTCCT
TACGCGGCCAACTTAACCTCCTCTATCGCCGTGTTAAACACCAACCTCTCGGCCGCCACA
AGCCTCCTTGTATGGACTACACTTGATGTCATCTTCTTTGGCAAACCTTCTGTCATCGGA
GCAATTCAAGGCATGGTTACTGGCTTAGCCGGCGTCACTCCCGGAGCAGGTTTGATCCA
A
ACATGGGCAGCTATAATAATTGGAGTAGTCTCAGGAACAGCTCCATGGGCCTCTATGATG
ATCATTCACAAGAAATCCGCTCTCCTTCAAAAGGTGGATGATACATTAGCGGTGTTTTAC
ACACACGCCGTGGCTGGTTTACTTGGTGGAATAATGACAGGGTTGTTTGCACACCCTGAT
CTCTGCGTTTTGGTACTTCCTCTCCCAGCGACCAGAGGAGCTTTCTACGGTGGCAATGG
C
GGCAAACAGCTTTTGAAACAGTTGGCTGGAGCTGCCTTCATTGCCGTCTGGAATGTGGT
G
TCGACTACTATCATTCTACTCGCTATTAGGGTGTTCATACCATTGAGAATGGCTGAGGAA
GAGCTCGGGATTGGAGACGACGCAGCACATGGGGAAGAAGCTTATGCTCTTTGGGGAG
AT
GGAGAGAAGTTTGATGCTACAAGGCATGTGCAACAGTTTGAGAGAGATCAAGAAGCTGC
T
CATCCTTCTTATGTTCATGGTGCTAGAGGTGTCACCATTGTTCTATGA
>AtAMT1.2
ATGGACACCGCAACCACCACATGCTCTGCCGTAGATCTATCTGCCCTCCTATCCTCTTCT
TCTAACTCAACATCTTCCCTCGCCGCGGCAACCTTTTTATGTTCCCAAATTTCAAACATC
TCCAACAAACTCTCCGACACAACTTATGCCGTCGACAACACGTATCTCCTCTTCTCCGCC
TACCTTGTCTTTGCCATGCAGCTCGGTTTCGCTATGCTTTGTGCTGGATCAGTCCGAGCC
AAGAACACTATGAACATCATGCTTACCAATGTCCTTGATGCTGCCGCTGGAGCCATCTCT
TACTACCTCTTCGGATTCGCATTCGCCTTTGGTACACCTTCCAACGGATTCATCGGTCGC
CACCATAGCTTCTTCGCTTTAAGCTCTTACCCTGAACGCCCCGGCTCCGACTTCAGCTTT
TTCCTCTACCAATGGGCTTTTGCCATAGCCGCGGCCGGAATCACTAGCGGTTCCATCGCC

GAGCGAACGCAATTCGTTGCTTACCTTATCTACTCTACTTTCTTGACCGGTTTTGTTTAC
CCGACAGTCTCGCACTGGTTCTGGTCAAGTGATGGATGGGCTAGCGCGTCCCGGTCTGA
C
AACAATCTCTTGTTTGGCTCAGGTGCTATTGATTTCGCAGGTTCAGGAGTTGTTCACATG
GTAGGTGGAATTGCCGGTTTATGTGGAGCGTTAGTTGAAGGACCAAGAATAGGTAGATTT
GACCGGTCAGGCCGGTCCGTGGCTTTACGTGGTCACAGTGCATCCCTTGTCGTGCTTGG
T
ACCTTCTTGTTGTGGTTTGGATGGTATGGGTTTAACCCTGGTTCCTTTTTAACCATTCTT
AAAGGCTACGACAAGTCTCGGCCATATTATGGTCAATGGAGCGCTGTAGGTCGCACCGC
G
GTCACCACAACGCTTTCTGGCTGCACCGCTGCGTTGACTACTCTATTCAGTAAACGGCTT
TTAGCAGGTCATTGGAACGTTATTGACGTATGCAACGGACTTCTAGGCGGCTTTGCAGCT
ATAACCTCCGGATGTGCCGTGGTGGAGCCGTGGGCTGCTATAGTATGTGGCTTTGTGGC
A
TCATGGGTTTTAATCGGATTTAACTTGCTTGCCAAGAAACTTAAATATGATGACCCACTC
GAGGCTGCTCAGCTCCACGGTGGATGTGGAGCATGGGGATTAATCTTTACCGGGCTGTT
C
GCAAGGAAAGAATACGTTAACGAGATTTACTCCGGTGATAGGCCTTACGGACTGTTCATG
GGCGGGGGAGGAAAACTGCTCGCCGCGCAGATCGTTCAGATTATTGTGATCGTTGGGTG
G
GTGACGGTAACTATGGGACCGTTGTTTTATGGGTTACATAAGATGAATCTTTTGAGGATA
TCAGCAGAAGATGAGATGGCAGGAATGGACATGACACGTCATGGAGGATTTGCTTACGCA
TACAATGACGAAGACGACGTGTCGACTAAACCATGGGGTCATTTCGCTGGAAGAGTGGA
G
CCTACAAGCCGGAGCTCGACTCCTACACCGACCTTGACTGTTTGA
>AtAMT1.5
ATGTCAGGAGCTATTACTTGCTCTGCGGCTGATCTCTCAGCCCTACTCGGCCCAAATGCC
ACGGCAGCGGCTGACTACATTTGCGGCCAGTTGGGTTCCGTTAACAACAAGTTTACCGAT
GCAGCCTACGCTATAGACAACACGTACCTCCTCTTCTCTGCCTATCTTGTCTTTGCGATG
CAGCTCGGCTTCGCTATGCTTTGTGCTGGCTCCGTTAGAGCTAAGAACACGATGAACATC
ATGCTCACTAATGTCCTTGATGCTGCAGCCGGAGGACTCTTCTACTACCTCTTTGGTTAT
GCATTTGCCTTTGGTGAATCCTCCGATGGATTCATTGGAAGACACAACTTTGGTCTTCAA
AACTTTCCGACTCTCACCTCGGATTACTCCTTCTTCCTCTACCAATGGGCGTTTGCAATC
GCAGCCGCTGGAATCACCAGCGGCTCCATTGCCGAGAGGACTAAGTTCGTGGCGTATTT
G
ATATACTCTTCTTTTTTGACCGGGTTTGTTTACCCAGTTGTCTCTCACTGGTTCTGGTCT
CCGGATGGATGGGCTAGTCCCTTCCGTTCAGAAGACCGTTTGTTTGGCACTGGAGCCAT
C
GACTTTGCTGGGTCAGGTGTTGTTCACATGGTTGGTGGTATCGCAGGATTATGGGGTGCC
CTTATTGAAGGCCCTCGGATTGGTCGGTTTCCTGATGGGGGTCATGCTATTGCTCTGCGA
GGCCACTCTGCCTCACTCGTCGTCTTAGGGACCTTCCTTCTCTGGTTTGGTTGGTACGG
G
TTCAACCCTGGTTCCTTCACCAAGATACTCATTCCCTACAATTCTGGTTCCAACTATGGC
CAATGGAGTGGAATAGGCCGCACCGCGGTTACAACTACACTCTCGGGATGCACAGCGGC

T
CTAACCACACTCTTCGGAAAACGTCTCCTATCAGGCCACTGGAACGTAACTGACGTTTGC
AACGGGTTACTCGGAGGGTTTGCGGCCATAACGGCAGGTTGCTCTGTGGTTGATCCATG
G
GCAGCGATCGTATGTGGCTTCGTGGCTTCCCTCGTCCTTATCGGATGCAACAAGCTCGCA
GAGCTCTTAAAATATGACGATCCACTTGAGGCCGCACAACTACACGGAGGGTGTGGTGCT
TGGGGTTTGATATTTGTAGGACTGTTTGCAAAAGAGAAGTATATAAATGAGGTTTACGGC
GCGAGCCCAGGAAGGCACTACGGGCTATTTATGGGCGGAGGAGGGAAGCTATTGGGAG
CA
CAACTGGTTCAAATAATTGTGATTGTTGGATGGGTTAGTGCCACAATGGGAACACTCTTC
TTCATCCTCAAAAAGCTCAATTTGCTTAGGATCTCGGAGCAGCATGAAATGCGAGGAATG
GATTTAGCAGGTCATGGTGGTTTTGCTTATATCTACCATGATAATGATGATGATTCCATT
GGAGTGCCTGGATCTCCAGTACCTCGTGCGCCTAACCCTCCAGCCGTTTGA
>AtAMT1.3
ATGTCAGGAGCAATAACATGCTCTGCGGCCGATCTCGCCACCCTACTTGGCCCCAACGC
C
ACGGCGGCGGCCGACTACATTTGCGGCCAATTAGGCACCGTTAACAACAAGTTCACCGA
T
GCAGCCTTCGCCATAGACAACACCTACCTCCTCTTCTCTGCCTACCTTGTCTTCGCCATG
CAGCTCGGCTTCGCTATGCTTTGTGCTGGTTCTGTTAGAGCCAAGAATACGATGAACATC
ATGCTTACCAATGTCCTTGACGCTGCAGCCGGAGGACTCTTCTACTATCTCTTTGGTTAC
GCCTTTGCCTTTGGAGGATCCTCCGAAGGGTTCATTGGAAGACACAACTTTGCTCTTAGA
GACTTTCCGACTCCCACAGCTGATTACTCTTTCTTCCTCTACCAATGGGCGTTCGCAATC
GCGGCCGCTGGAATCACAAGTGGTTCGATCGCAGAGAGGACTCAGTTCGTGGCTTACTT
G
ATATACTCTTCTTTCTTAACCGGATTTGTTTACCCGGTTGTCTCTCACTGGTTTTGGTCC
CCGGATGGATGGGCCAGTCCCTTTCGTTCAGCGGATGATCGTTTGTTTAGCACCGGAGC
C
ATTGACTTTGCTGGCTCCGGTGTTGTTCACATGGTTGGTGGCATAGCAGGTTTATGGGGT
GCTCTTATTGAAGGTCCTCGTCGTGGTCGGTTCGAGAAAGGTGGTCGCGCTATTGCTCT
G
CGCGGCCACTCTGCCTCGCTAGTAGTCTTAGGAACCTTCCTCCTATGGTTTGGATGGTAT
GGTTTCAACCCCGGTTCCTTCACTAAGATACTCGTTCCGTATAATTCTGGTTCCAACTAC
GGCCAATGGAGCGGAATCGGCCGTACAGCGGTTAACACCACACTCTCAGGATGCACAGC
A
GCTCTAACCACACTCTTTGGTAAACGTCTCCTATCAGGCCACTGGAACGTAACGGACGTT
TGCAACGGGTTACTCGGTGGGTTTGCGGCCATAACCGCAGGTTGCTCCGTCGTAGAGCC
A
TGGGCAGCGATTGTGTGCGGCTTCATGGCTTCTGTCGTCCTTATCGGATGCAACAAGCTC
GCGGAGCTTGTACAATATGATGATCCACTCGAGGCAGCCCAACTACATGGAGGGTGTGG
C
GCGTGGGGGTTGATATTCGTAGGATTGTTTGCCAAAGAGAAGTATCTAAACGAGGTTTAT
GGCGCCACCCCGGGAAGGCCATATGGACTATTTATGGGCGGAGGAGGGAAGCTGTTGG
GA

GCACAATTGGTTCAAATACTTGTGATTGTAGGATGGGTTAGTGCCACAATGGGAACACTC
TTCTTCATCCTCAAAAGGCTCAATCTGCTTAGGATCTCGGAGCAGCATGAAATGCAAGGG
ATGGATATGACACGTCACGGTGGCTTTGCTTATATCTACCATGATAATGATGATGAGTCT
CATAGAGTGGATCCTGGATCTCCTTTCCCTCGATCAGCTACTCCTCCTCGCGTTTAA
>AtAMT1.1
ATGTCTTGCTCGGCCACCGATCTCGCTGTCCTGTTGGGTCCTAATGCCACGGCGGCGGC
C
AACTACATCTGTGGCCAGTTAGGCGACGTCAACAACAAATTTATCGACACCGCTTTCGCT
ATAGACAACACTTACCTTCTCTTCTCCGCCTACCTTGTCTTCTCTATGCAGCTTGGCTTC
GCTATGCTCTGTGCCGGTTCCGTGAGAGCCAAGAATACTATGAACATCATGCTTACCAAC
GTCCTTGACGCTGCAGCCGGTGGTCTCTTCTATTATCTGTTTGGCTACGCCTTTGCCTTT
GGATCTCCGTCCAATGGTTTCATCGGTAAACACTACTTTGGTCTCAAAGACATCCCCACG
GCCTCTGCTGACTACTCCAACTTTCTCTACCAATGGGCCTTTGCAATCGCTGCGGCTGGA
ATCACAAGTGGCTCGATCGCTGAACGGACACAGTTCGTGGCTTACCTAATCTATTCCTCT
TTCTTAACCGGGTTTGTTTACCCGGTCGTCTCTCACTGGTTCTGGTCAGTTGATGGATGG
GCCAGCCCGTTCCGTACCGATGGAGATTTGCTTTTCAGCACCGGAGCGATAGATTTCGCT
GGGTCCGGTGTTGTTCATATGGTCGGAGGTATCGCTGGACTCTGGGGTGCGCTCATCGA
A
GGTCCACGACTTGGCCGGTTCGATAACGGAGGCCGTGCCATCGCTCTTCGTGGCCACTC
G
GCGTCACTTGTTGTCCTTGGAACATTCCTCCTCTGGTTTGGATGGTACGGATTTAACCCC
GGTTCCTTCAACAAGATCCTAGTCACGTACGAGACAGGCACATACAACGGCCAGTGGAG
C
GCGGTCGGACGGACAGCTGTCACAACAACGTTAGCTGGCTGCACCGCGGCGCTGACAA
CC
CTATTTGGGAAACGTCTACTCTCGGGACATTGGAACGTCACTGATGTATGCAACGGCCTC
CTCGGAGGGTTTGCAGCCATAACTGGTGGCTGCTCTGTCGTTGAGCCATGGGCTGCGAT
C
ATCTGCGGGTTCGTGGCGGCCCTAGTCCTCCTCGGATGCAACAAGCTCGCTGAGAAGCT
C
AAATACGACGACCCTCTTGAGGCAGCACAACTACACGGTGGTTGCGGTGCGTGGGGACT
A
ATATTCACGGCTCTCTTCGCTCAAGAAAAGTACTTGAACCAGATTTACGGCAACAAACCC
GGAAGGCCACACGGTTTGTTTATGGGCGGTGGAGGAAAACTACTTGGAGCTCAGCTGAT
T
CAGATCATTGTGATCACGGGTTGGGTAAGTGCGACCATGGGGACACTTTTCTTCATCCTC
AAGAAAATGAAATTGTTGCGGATATCGTCCGAGGATGAGATGGCCGGTATGGATATGACC
AGGCACGGTGGTTTTGCTTATATGTACTTTGATGATGATGAGTCTCACAAAGCCATTCAG
CTTAGGAGAGTTGAGCCACGATCTCCTTCTCCTTCTGGTGCTAATACTACACCTACTCCG
GTTTGA
>AtAMT1.4
ATGGCGTCGGCTCTCTCTTGCTCTGCCTCTGATCTGATTCCATTACTATCAGGTGGAGCC
AACGCCACCGCAGCAGCAGCCGCCGCTGAATACATCTGCGGGAGATTCGACACAGTCG
CC

GGGAAATTCACTGATGCGGCTTACGCAATCGACAACACTTACCTTCTCTTCTCTGCTTAC
CTCGTTTTCGCGATGCAGCTCGGTTTCGCCATGCTCTGTGCCGGATCCGTACGTGCAAA
A
AACACGATGAACATTATGCTCACGAACGTCATCGACGCTGCAGCCGGAGGTCTCTTCTAT
TATCTCTTCGGTTTCGCTTTTGCTTTTGGATCTCCTTCTAATGGATTCATCGGAAAACAT
TTCTTTGGAATGTATGATTTTCCTCAACCTACGTTTGATTATCCTTATTTTCTATATCAA
TGGACTTTCGCTATCGCCGCCGCTGGAATCACGAGTGGTTCGATAGCGGAGAGGACTCA
G
TTCGTTGCGTATTTGATCTATTCTTCTTTCTTGACGGGTCTTGTTTACCCGATTGTGTCG
CATTGGTTTTGGTCTTCTGATGGTTGGGCGTCTCCGGCTAGATCTGAGAACCTTCTGTTT
CAATCAGGTGTGATTGATTTCGCTGGCTCTGGTGTTGTTCATATGGTTGGTGGTATTGCT
GGTTTATGGGGAGCTTTAATTGAAGGACCTAGGATTGGTCGGTTTGGAGTTGGGGGTAAA
CCGGTTACGTTGCGTGGTCATAGTGCTACGTTGGTTGTTCTTGGAACGTTTTTGTTATGG
TTCGGATGGTACGGGTTTAACCCGGGCTCGTTTGCAACTATTTTTAAGGCGTATGGGGAG
ACTCCAGGGAGCTCGTTTTACGGACAATGGAGCGCAGTTGGGAGAACCGCGGTAACAAC
T
ACGTTAGCTGGTTGCACGGCGGCGTTAACGACTCTGTTTGGGAAAAGACTTATTGATGGG
TATTGGAATGTAACTGATGTTTGCAATGGTTTGTTAGGCGGGTTTGCGGCTATAACTAGC
GGATGTTCGGTTGTGGAACCGTGGGCTGCGCTTGTATGTGGGTTTGTAGCCGCATGGGT
G
CTGATGGGATGCAATAGACTAGCGGAAAAGCTCCAATTTGATGATCCGTTGGAAGCGGCT
CAGCTTCACGGTGGTTGTGGTGCGTGGGGGATTATTTTCACCGGGTTGTTCGCGGAGAA
A
AGATACATTGCCGAGATCTTTGGAGGCGACCCGAATAGGCCTTTCGGATTGCTAATGGGA
GGAGGAGGTAGGTTGCTTGCGGCGCACGTCGTTCAGATTTTGGTGATTACGGGTTGGGT
T
AGTGTGACAATGGGGACTCTGTTTTTTATTTTGCATAAGCTGAAACTGTTGAGGATACCG
GCGGAGGATGAGATAGCTGGGGTGGATCCGACGAGTCACGGAGGGTTGGCTTATATGTA
C
ACAGAAGATGAGATTAGGAATGGGATCATGGTTAGGAGAGTGGGTGGTGATAATGATCCC
AATGTAGGTGTTTGA
>GmAMT4.2
ATGTCACTCTATGCTTTTACATGTGTGTTCTTCTGTTGGGTTGTGTGGGGATACAGAATGT
CTTTTGGTGATGAGCTTCTTCCTTTCTGGGGAAAGCCTGCTATGTCACTTGAGCATGCATA
TCTTTTCAAGAAGGCATTTTTGGGGGCCTTCCCAAATGCCACAATGTGTGTGTTTGCTGC
CATCACTTTGATTCTCATTGCTGGGGCCGTGTTGGGGCACATGAACTTCTATGCTTGGAT
GCTTCCCTGGCCGTTCTCAACACCCACGCTTGCTCACTTGGTCATCCTTGATGTCCTCTT
TTTCCGAAAGCCTTCTGTAATTGGAGCTGTTCAAGGCATGATCACTGGTTTGGTTTGCATA
ACCCCTGCTGCAGGTGTTGTTCAGGGATGGGCATCACTTATAATGGGACTTCTCTCAGGC
TCAATTCCTTGGTTCACCATGATGGTCATCCATAAGAGATCAAAGCTACTGCAGAAGGTTG
ATGATACTATGGCAGTGTTCCATACTCATGCCATTGCAGAAACCCTAGGAGGACTCCTCAC
TGGGCTCTTTGCTGATCCAAGACTCAACCGGTTGTTCTATGGACATGATGCTCAATACGTT
GTTTTCTTTTATGGTTTCGGTTCCAACAAGCTCAGCACCGGCTTCAGGCAAATGGGAGTC
CAAATCCTTGGGATTATCTTTGTTATCTTTGTCAACTTCATTCGGATCTTTTTTCCATTGAGA

ATTTCTGAAGAGGATATGGAGATAGGTGATGAGGCTGCTCATGGTGAAGAAGCTTATGCA
ATCTGGGGTCAGGGTGATAAGCTTGAAAACTCAAGTTCAAAGTATGGAAGTTCATTATATA
ATGATGTTGAAGCTGGTGCCCCCAAGAAGAAGCGTGCTGGCACTGTTCAGATGATGTCC
AACTAA
>GmAMT1.1
ATGTCGCTGCCAGATTGTCCCGCCGTCCAACTTGCCCAACTCCTGGGCCCAAATACCAC
AAACGCTGCCGCCGCCGCCTCCTTCATCTGCGACCGGTTCACCGCCGTGGACAACAAG
TTCGTCGACACGGCCTTCGCGGTCGACAACACTTACCTCCTCTTCTCCGCCTACCTCGTC
TTCTCGATGCAGCTCGGCTTCGCCATGCTCTGCGCCGGCTCCGTCCGCGCCAAGAACAC
CATGAACATCATGCTCACCAACGTCCTCGACGCCGCCGCCGGCGGCCTCTTCTACTACC
TCTTCGGCTTCGCCTTCGCCTTCGGCTCCCCCTCCAACGGCTTCATTGGCAAACACTTCT
TCGGCCTCAAGGAACTCCCCTCCCAAAGCTTCGACTACAGCAACTTTCTCTATCAATGGG
CCTTCGCCATCGCCGCCGCCGGCATCACCAGCGGCTCCATCGCCGAACGCACACAGTT
CGTGGCCTATCTCATCTACTCCTCCTTCCTCACCGGCTTCGTCTACCCCGTCGTCTCCCA
CTGGTTCTGGTCCGCAGACGGCTGGGCTTCTGCCATTTCCCCCGGAGACCGGCTATTTT
CCACCGGCGTGATAGACTTCGCCGGCTCCGGCGTAGTCCACATGGTTGGTGGAGTAGCC
GGCTTCTGGGGCGCACTGATAGAAGGCCCGAGAATCGGACGCTTCGACCACGCGGGAC
GCGCCGTTGCCCTCAGAGGCCACAGCGCGTCCTTAGTAGTCCTGGGGACCTTCATGCTT
TGGTTCGGTTGGTACGGATTTAACCCTGGTTCATTTAATAAAATCCTAGTTTCCTACGGTAA
CTCAGGAACTTACTACGGTCAATGGAGCGCGGTTGGGAGAACCGCGGTCACCACTACCC
TTGCAGGGTCAACTGCTGCGTTGACCACTCTCTTCGGGAAACGGATGATATCCGGTCATT
GGAACGTGACCGATGTCTGCAACGGGCTGTTAGGCGGCTTCGCCGCCATAACAGCCGG
CTGCTCCGTCGTTGAGCCATGGGCAGCCATCGTGTGTGGTTTTGTCGCTTCAGTGGTTC
TGATAGCGTGCAACAAATTAGCAGAGAAGGTTAAGTTCGATGATCCTTTGGAAGCGGCGC
AGTTACACGGTGGGTGTGGCGCGTGGGGGGTGATATTCACGGCGCTGTTCGCGAAAAA
GGAGTATGTGAGCCAGGTTTATGGGGAGGGGAGGGCGCACGGGTTGTTCATGAGGGGT
GGAGGGAAGTTGCTGGCGGCGCACGTGATTCAGATTTTGGTTATTGTTGGGTGGGTGAG
TGCGACCATGGGACCCTTGTTTTGGGGGTTGAATAAATTGAAATTGTTGAGGATTTCTTCC
GAGGATGAGCTTGCGGGGATGGATCTTACCCGTCATGGAGGATTTGCTTATGCTTATGAG
GATGATGAGTCGCACAAGCATGGGATTCAGCTGAGGAAGGTTGGGCCCAACGCGTCGTC
CACACCCACCACTGATGAATGA
>GmAMT1.6
ATGCTCTGCGCCGGCTCCGTCAGAGCCAAGAACACCATGAACATCATGCTCACCAAAGT
CCTCGACGCCGCCGCCGGCGGCCTCTCTTACTACCTCTTCGGCTTCGCATTCGCCTTCG
GCGCCCTCTCCAACGGCTTCATCGGCCGCCACTTCTTCGGCCTCCGAGATTTCCCGATG
GGTGCCTCTCCCTCCGGCGACTACAGCTTCTTCCTCTACCAGTGGGCCTTCGCCATCGC
CGCCGCCGGAATCACCAGCGGCACCATCGCCGAGAGAACACAGTTCGTGGCTTACCTC
ATCTACTCTTCTTTCTTAACCGGTTTGGTTTACCCCATCGTTTCGCATTGGTTCTGGTCCTC
AGACGGTTGGGCCAGCGCGGCTCGTAGCAACGGAAATGTTTTGTTCGGGTCTGGAGTCA
TCGACTTTGCGGGCTCAGGCGTTGTTCACATGGTTGGCGGGATAGCGGGCATGTGGGG
GGCTTTAATTGAAGGCCCGAGAATCGGCCGGTTCGACCGGACGGACCGGTCCGTGGCT
TTGCGTGGCCACAGCGCGTCTTTAGTTGTGCTTGGTTCGTTTTTGTTGTGGTTCGGGTG
GTACGGCTTCAACCCTGGTTCGTTTATCACTATAGCGAAGGGTATTATGGTCAATGGAGCG
CGATAG

>GmAMT4.4
ATGAGTTCTAGTTCTTATGATATCCCTTTGCCATCGTCCCTTTTGCCAAGCGAGGCATCGC
CGGAGTGGAACAACAAAGCAGACAACGCGTGGCAGCTCACGGCGGCAACCCTAGTGGG
CCTCCAGAGCGTTCCCGGGCTCGTGATCCTATATGGCAGCATGGTAAAGCGCAAATGGG
CCGTGAACTCGGCGTTCATGGCCCTGTACGCCTTCGCGTGTGTGCTCATTTGTTGGGTTT
CATGGGCACACCGCATGGCGTTCGGGGCCAGGCTTTTACCCTTTGTGGGAGCGCCCAA
CCATGCCTTGGCCGAGAAGTTTCTCCTCGCAAAGTCAACAATTGGGTATTTTCCAATGGC
TGACTTTGTGTTCTACCAGTTTGCTTTTGCCGCAATTACTCTTGTGTTGCTTGCAGGGTCC
TTGCTTGGGAGAATGAACTTCTATGCGTGGATGATGTTTGTCCCTTTGTGGCTTACGTTGT
CTTATACCGTTGGTGCCTTCAGCATATGGGACGATGATGGGTTCCTTCAGGGAAAAATAAT
TGACTATGCTGGTGGGTTTGTCATTCATTTGTCTTCTGGCGTTGCGGGTTTCACAGCTGC
TTATTGGGTTGGTCCAAGAATTTCACAGGACAGGCAAAACTTTCCACCAAACAACATAATT
CACGTGCTTGGAGGTGCAGGGTTTTTGTGGATGGGTTGGACAGGTTTCAATGGTGGAGC
TCCATTTCAAGTGGGAGAAATCGCATCCTTGGCCATTTACAATACCCATCTTTGCACTGCC
ACAAGCCTACTCGTTTGGCTTACGCTTGACATGATTGTATATACCAAGAGCTCAGTCATAG
GTGCTGTCCAGGGAATGATCACTGGCCTCGTCTGCATCACACCAGGTGCAGGCTTGGTG
GATCCATGGGCAGCAGTATTGATGGGAGCATTGTCTGGTTCCATTCCATGGTACACAATG
ATGGTGCTACACAAAAAATCCGCATTCTTTCAAAGTGTAGATGACACATTAGGAGTCTTTC
ACACTCATGCTGTGGCTGGTCTTCTTGGGGGTATCCTTTCTGGCGTGTTTGCCAAACCCG
ACCTTCTAAGAATAATGTATCAAGATACAAATTATTGCCCCGGCTTATTCTACACCTTTTTA
AGGGGGAAGTGGACCATGGCTTTAGGCAAATTTGGATGAAAGAAGAGGATCTTGAGGTT
GGTGATGATGCTGCCCATGGGGAAGAAGCATATGCATTGTGGGGTGATGGAGAGAGAAT
GAGGATCCCTCTTCGTCTTCATATAGGTCCAACAATTCCTTCCTTGTGCCGAAAAAGGTTT
TCAATTCCTCTCACTAGGAAAGAAGGAGAATAG
>GmAMT4.5
ATGAGTCACCTACCATCAAACTTGTTGCCCGTTGACGGAAGCCCAGAGTGGATGAACAG
GGGTGACAACACGTGGCAGCTGACGTCAGCTACTCTGGTGGGCCTGCAAAGCGTTCCG
GGCCTGGTCATCCTCTACGGCAGCATAGTGAAGAAGAAATGGGCTTTGAACTCTGCCTTC
ATGGCCCTCTACGCTTTTGCGGCGGTTCTTGTTTGTTGGGTTGGGTGGTGCTACAAAATG
TCATTCGGCGAGAGGATCCTTCCATTTCTGGGACAGCCAAACACGGCCTTGGACCGGGA
ATTCGTTTTTCGCAAAACTTTTGCTGGGGAATTTCCCAACGCCACCATGGTGTACTTTCAG
CTGGTTTTTGCTGCCATCACGTTGATTCTCATAGCGGGGGCGTTGCTTGGGAGAATGAAC
TTTTATGCATGGATGTTGTTTGTTCCATTATGGGTTACGTTCTCTTACACCATTTGTGCCTT
CAGTATATGGAGCACCGATGGATGGTTGTTCAAGATGGGCCTCATTGATTATTCTGGTGGC
TTTGTTATTCACCTTTCTTCTGGGGTTGCTGGTTTCGTCGCAGCATGTTGGGTAGGACCA
AGGGCAGTGAAGGACAGAGAGAGGTTCCCTCCAAACAACATACTTCTGATGCTGGCTGG
GGCTGGCTTACTATGGATGGGTTGGTCTGGATTCAACGGTGGAGATCCATACGCAGCCA
GCATAGATGCATCTCTGGCCGTGCTTAACACCCACGTGTGCACAGCAATGAGCTTGTTGA
CTTGGCTTTTCCTTGACATCCTCTTCTTTGGAAAACCTTCCGTCATTGGTGCCACTCAAGG
CATGATCACTGGCCTTGTTTGCATCACCCCTGCTGCCGGTACCATATGCCCACGTGTGGT
GCAAGGTTGGGCAGCAATCATAATGGGAATGATGTCCGGAAGCATCCCATGGTACACAAT
GATGGTCCTGCACAAAGAAAGTAAGCTCCTCAAACAAGTTGATGATACTCTAGCAGTTTTC
CACACCCATGCCGTGGCTGGTAGCCTAGGTGGCATTTTGGCTGGTTTTTTTGCTGACCCT
AAGCTTTGTTATCTCTTCTATGGAGTAGAAGATTCCCTACATTATACGGGTCTTGTTTATGG

CATATTCAATGGGAGGCCAAATGCAGGGTTCAGGCAAATGATGGTTCAGCTTCTTGGCAT
TGTGTTTGTGATACTTCTCAATGTCACCACAACTAGCATTGTGTGCTTGTTGGTTAGGATC
ATAGTTCCACTTAGGCTGAGTGAAGATGACTTGCAAGTTGGGGATGAAGCAGTCCATGGC
GAAGTTGCATATGCATTGTGGGGTGATGGAGAGAAGCTTGAGACTGCTACTGAGCCTTTG
ACATCTGAAAAGAATCTTGAATGGCCAACTGTGGAACTAAGAACTTAA
>GmAMT1.2
ATGTCGCTGCCTGCTTGTCCCGCCGAACAACTGGCCCAACTTCTCGGCCCAAACACCAC
AGACGCCTCCGCCGCCGCCTCCCTTATCTGCGGCCAGTTCGCCGCCGTGGACAGCAAG
TTCGTCGACACGGCCTTCGCCGTCGACAACACCTACCTCCTCTTTTCCGCCTACCTCGTT
TTTTCTATGCAGCTCGGCTTCGCCATGCTCTGCGCCGGCTCCGTCCGCGCCAAGAACAC
CATGAACATCATGCTCACCAACGTCCTGGACGCTGCCGCCGGCGGCCTCTTCTACTACC
TCTTCGGCTTCGCCTTCGCTTTCGGCTCCCCCTCCAACGGCTTCATCGGTAAACATTTCT
TCGGCCTCAAGGACATCCCTTCATCCTCCTACGACTACAGCTACTTCCTCTACCAATGGG
CCTTCGCCATCGCCGCCGCCGGCATCACCAGCGGAAGCATCGCCGAACGCACACAGTT
CGTGGCCTATCTCATCTACTCCTCCTTCCTCACCGGCTTCGTCTATCCGGTGGTCTCCCA
CTGGTTCTGGTCCCCAGACGGCTGGGCCTCTGCCTTTAAGATCACCGACCGGCTATTTT
CCACCGGCGTAATAGACTTCGCCGGTTCCGGCGTAGTCCACATGGTCGGCGGAATAGCC
GGCCTATGGGGAGCGCTGATCGAAGGCCCAAGAATGGGACGTTTCGATCATGCAGGACG
AGCTGTGGCCTTGCGAGGCCACAGCGCGTCCTTAGTAGTCCTGGGAACCTTCTTGCTTT
GGTTCGGTTGGTACGGATTTAACCCCGGTTCATTTAACAAAATCCTACTTACTTACGGTAA
CTCAGGAAATTACTACGGTCAATGGAGCGCGGTTGGCAGAACCGCGGTCACCACTACCC
TAGCGGGGTCAACAGCTGCCTTGACCACGCTATTCGGTAAACGGGTGATATCCGGTCACT
GGAACGTGACCGATGTCTGCAACGGGCTGTTAGGCGGTTTCGCGGCGATAACAGCCGG
TTGCTCCGTGGTTGAGCCATGGGCAGCCATCGTATGCGGTTTTGTTGCTTCTATAGTATTA
ATAGCTTGCAACAAATTAGCAGAGAAGGTTAAGTTCGACGATCCTCTGGAGGCGGCGCA
GTTGCACGGTGGGTGTGGCACGTGGGGGGTGATATTCACGGCGTTGTTCGCAAAAAAG
GAGTATGTGAAGGAGGTTTACGGGTTGGGGAGGGCGCACGGGTTGCTCATGGGGGGTG
GTGGGAAGTTGCTGGCGGCGCACGTGATTCAGATTCTGGTGATTGCTGGGTGGGTTAGT
GCGACCATGGGACCCTTGTTTTGGGGGTTGAATAAACTGAAGCTGTTGAGGATTTCTTCA
GAGGATGAGCTTGCGGGGATGGACATGACTCGCCATGGAGGCTTTGCTTATGCTTATGAG
GATGATGAGACGCACAAGCATGGGATGCAGTTGAGGAGGGTTGGGCCCAACGCGTCTT
CCACACCCACCACTGATGAATGA
>GmAMT1.5
ATGGCTTCTCTCTCTTGCTCCGCCAACGACCTTGCCCCACTCATCAACGACACCGCCGC
CGCCAACTACCTCTGCGCACAATTCGATTCCATTTCTAGAAAGCTCGTCGAAACAACCTAC
GCCGTCGACAACACCTACCTTCTGTTTTCAGCGTATCTTGTCTTCGCCATGCAGCTCGGC
TTCGCCATGCTCTGCGCCGGCTCCGTCAGAGCCAAAAACACCATGAACATCATGCTCAC
CAACGTCCTCGACGCCGTCGCCGGCGGTCTCTCCTACTACCTATTCGGCTTTGCATTCGC
CTTCGGCGGCCCCTCCAACGGCTTCATCGGCCGCCCAAACTTCCGCCTACGAGATTACC
CGATGGGCTCCTCTATCTCCAGCTTCTTCCTCTACCAGTTGGTCTTAGCCATCGCCGACG
CAGGAATCACCATCTCTCCCTTCGGCGACTACAGCTTCTTCCTCTACCAGTGGGCCTTCG
CCATCGCCGCCGCAGGAATCACCAGCGGCTCCATCGCCGAGAGAACACAGTTCGTGGC
TTACCTTATCTACTCTTCTTTCTTAACCGGTTTCGTTTACCCCATCGTTTCGCATTGGTTCT
GGTCCTCAGACGGTTGGGCCAGCGCGACTCGTAGCCACGGAAATGTTTTATTCGGGTCT

GGAGTCATCGACTTCGCGGGCTCAGGCGTTGTTCACATGGTTGGCGGGATAGCGGGCC
TGTGGGGGGCTTTAATTGAAGGCCCGAGAATCGGCCGGTTCGACCGTTCGGGCCGGTC
GGTTGCTTTACGTGGCCACAGCGCGTCTTTAGTTGTGCTTGGTACGTTTTTGTTATGGTTC
GGCTGGTACGGCTTCAACCCTGGTTCGTTTGTGACAATAGACAAGGGGTATGAAAGTGG
AGGGTATTATGGTCAATGGAGCGCTATAGGGAGGACAGCTGTCACGACGACATTGGCTG
GGAGCACTGCGGCTCTGACGACGTTGTTCAGCAAGCGGTTATTGGTTGGCCACTGGAAC
GTGATTGACGTGTGTAACGGCCTGCTTGGCGGGTTCGCTGCCATTACATCGGGCTGTGC
CGTTGTGGAACCGTGGGCCGCGATTGTCTGCGGGTTTGTGGCGGCGTGGGTTTTGATTT
GGCTTAATAAGCTTGCCGCGAAGGTAGAGTACGATGATCCGTTGGAGGCGGCGCAGCTT
CACGGCGGGTGCGGAGCTTGGGGGGTTTTCTTCACGGGATTGTTTGCGAAGAAAGTGTA
CGTGGAGGAGATTTACGGTGTTGGAAGGCCGTTCGGGGCTTTGATGGGTGGCGGAGGG
AGGCTGCTGGCGGCGCAGGTGATTCAGATATTGGTGGTGTGCGGGTGGGTTACGGCGA
CCATGGCGCCGTTGTTCTATGGGCTTCATAAGATGAAACTGTTGAGAATTTCGAGGGATG
ATGAGACTGCGGGGATGGATTTGACGAGGCATGGTGGGTTTGCTTATGCATACCATGATG
ATGAAGATGGTTCAAGCAGGGGAGTAGGATTCATGATGCTTAGAATTGAGCCTGCTGGTA
CCTCTCCCTCTCCCCCCGCTGCACCACAAGTTTAA
>GmAMT1.3
ATGGCTTCTCTCTCTTGCTCCGCCAACGACCTTGCCCCACTCTTCAACGACACCGCCGC
CGCCAACTACCTCTGCGCCCAATTCGATTCCATTTCTAGAAAGCTCGCCGAAACAACCTA
CGCCGTCGACAACACCTACCTTCTGTTTTCAGCGTATCTTGTCTTCGCCATGCAGCTCGG
CTTCGCCATGCTCTGCGCCGGCTCCGTCAGAGCCAAAAACACCATGAACATCATGCTCA
CCAACGTCCTCGACGCCGCCGCCGGCGGTCTCTCCTACTACCTATTCGGCTTTGCATTC
GCCTTCGGCGGCCCCTCCAACGGCTTCATCGGCCGCCACTTCTTCGGCCTACGAGATTA
CCCGATGGGCTCCTCTCCCTCCGGCGACTACAGCTTCTTCCTCTACCAGTGGGCCTTCG
CCATCGCCGCCGCAGGAATCACCAGCGGCTCCATCGCCGAGAGAACACAGTTCGTGGC
TTACCTTATCTACTCTTCTTTCTTAACCGGTTTCGTTTACCCCATCGTTTCGCATTGGTTCT
GGTCCTCGGACGGTTGGGCCAGCGCGAGTCGTAGCGACGGAAATGTTTTATTCGGGTCT
GGAGTCATCGACTTCGCGGGCTCAGGCGTTGTTCACATGGTTGGCGGGATAGCGGGCC
TGTGGGGGGCTTTAATTGAAGGCCCGAGAATCGGCCGGTTCGACCGTTCGGGCCGGTC
GGTTGCTTTACGTGGCCACAGCGCGTCTTTAGTTGTGCTTGGTTCGTTTTTGTTATGGTTC
GGCTGGTACGGCTTCAACCCTGGTTCGTTTCTGACAATAGCCAAGGGGTATGGAAGTGG
AGGGTATTATGGTCAATGGAGCGCTATAGGGAGGACAGCTGTCACGACGACATTGGCTG
GGAGCACTGCGGCTCTGACGACGTTGTTCAGCAAGCGGTTATTGGCTGGCCACTGGAAC
GTGATTGACGTGTGTAACGGCCTGCTTGGCGGGTTCGCTGCCATTACATCGGGCTGTGC
CGTTGTGGAACCGTGGGCCGCGATTGTGTGTGGGTTTGTGGCGGCGTGGGTTTTGATTG
GGCTTAATAAGCTTGCCGCGAAGGTAGAGTACGATGATCCGTTGGAGGCGGCGCAGCTT
CACGGCGGGTGCGGCGCGTGGGGTGTTTTCTTCACGGGATTGTTTGCGAAGAAAGTGTA
CGTGGAGGAGATTTACGGTGTTGGAAGGCCGTTCGGGGCTTTGATGGGTGGCGGAGGG
AGGCTGCTGGCGGCGCAGGTGATTCAGATATTGGTGGTGTGCGGGTGGGTTACGGCGA
CCATGGCGCCGTTGTTCTATGGGCTTCATAAGATGAAACTGTTGAGAATTTCGAGGGATG
ATGAGACTGCGGGGATGGATTTGACGAGGCATGGTGGGTTTGCTTATGCATACCATGATG
ATGAAGATGGTTCAAGCAGGGGAGTAGGGTTCATGCTGCGTAGAATTGAGCCTGCTGCTA
GTACCACTCCCTCTCCCCCCGCTGCACCACAAGTTTAA
>GmAMT1.4

ATGGCTTCTCTCTCTTGCTCCGCCAACGACCTTGCCCCACTCTTCAACGACACCGCCGC
CGCCAACTACCTCTGCGCCCAATTCGATTCCATTTCTAGAAAGCTCAACGTAACAACCTAC
GCCGTCGACAACACCTACCTTCTGTTTTCAGCGTATCTTGTCTTCGCCATGCAGCTCGGC
TTCGCCATGCTCTGCGCCGGCTCCGTCAGAGCCAAAAACACCATGAACATCATGCTCAC
CAACGTCCTCGACGCCGCCGCCGGCGGTCTCTCCTACTACCTATTCGGCTTTGCATTCG
CCTTCGGCGGCCCCTCCAACGGCTTCATCGGCCGCCACTTCTTCGGCCTACGAAAATTC
CCGAAGGACTCCCCTCCCTCCGGCGACTACAGCTTCTTCCTCTACCACTGGGCCTTCGC
CATCGCCGCCGCAGGAATCACCAGCGGCTCCATCGCCGAGAGAACACAGTTCGTGGCT
TACCTTATCTACTCTTCTTTCTTAACCGGTTTCGTTTACCCCATCGTTTCGCATTGGTTCTG
GTCCTCAGACGGTTGGGCCAGCGCGACTCGTAGCCACGGAAATGTTTTATTCGGGTCTG
GAGTCATCGACTTCGCGGGCTCAGGCGTTGTTCACATGGTTGGCGGGATAGCGGGCCT
GTGGGGGGCTTTAATTGAAGGCCCGAGAATCGGCCGGTTCGACCGTTCGGGCCGGTCG
GTTGCTTTACGTGGCCACAGCGCGTCTTTAGTTGTGCTTGGTACGTTTTTGTTATGGTTCG
GCTGGTACGGCTTCAACCCTGGTTCGTTTCTGACAATAGCCAAGGGGTATGAAAGTGGA
GGGTATTATGGTCAATGGAGCGCTATAGGGAGGACAGCTGTCACGACGACATTGGCTGG
GAGCACTGCGGCTCTGACGACGTTGTTCAGCAAGCGGTTATTGGCTGGCCACTGGAACG
TGATTGACGTGTGTAACGGCCTGCTTGGCGGGTTCGCTGCCATTACATCGGGCTGTGCC
GTTGTGGAACCGTGGGCCGCGATTGTGTGTGGGTTTGTGGCGGCGTGGGTTTTGATTG
GGCTTAATAAGCTTGCCGCGAAGGTAGAGTACGATGATCCGTTGGAGGCGGCGCAGCTT
CACGGCGGGTGCGGAGCGTGGGGGGTTTTCTTCACGGGATTGTTTGCGAAGAAAGAGT
ACGTGGAGGAGATTTACGGTGGTGGAAGGCCGTTCGGGGCTTTGATGGGTGGCGGAGG
GAGGCTGCTGGCGGCGCAGGTGATTCAGATATTGGTGGTGTGCGGGTGGGTTACGGCG
ACCATGGCGCCGTTGTTCTATGGGCTTCATAAGATGAAACTGTTGAGAATTTCGAGGGAT
GATGAGACTGCGGGGATGGATTTGACGAGGCATGGTGGGTTTGCTTATGCATACCATGAT
GATGAAGATGGTTCAAGCAGGGGAGTAGGGTTCATGCTGCGTAGAATTGAGCCTGCAGC
TATTACCACTCCCTCTCCCCCCGCTGCACCACAAGTTTAA
>GmAMT4.6
ATGGCTTACCATGGATTCAACGGTGGAGATCCATACGCAGCCAGCATAGATGCATCTCTG
GCCGTCCTTAACACCCACGTCTGCACGGCAATGAGCTTGTTGACTTGGCTTTTCCTTGAC
ATACTCTTCTTTGGAAAACCTTCCGTCATTGGTGCCACTCAAGGCATGATCACTGGCCTG
GTTTGCAACACCCCTGCTGCCGGTGTGGTGCAAGGTTGGGCAGCAATCATAATGGGAAT
GATGTCTGGAAGCATCCCATGGTACACAATGATGGTCCTGCACAAAGAAAGTAAGCTACT
CAAACAAGTAGATGATACTTCAGCAGTTTTCCACACCCATGCCGTGGCTGGTAGCTTAGG
TGGCATTTTGGCTGGTTTCTTTGCTGACCCTAACCTTTGTTATCTCTTCTATGGAGTACAA
GATTCCTTGCACTATACGGGTCTTGTTTATGGCATATTCAATGGGAATCTCAAGACAGGGT
TCAGGCAAATGGGGGTTCAGCTTCTTGGCATTGTTTTTGTAATACTTCTGAATGTCACCAC
AACTAGCATTGTGTGTTTGTTGGTTAGGATGATAGTTCCACTTAGGATGAGTGAAGATGAC
TTGCAAGTTGGGGATGAAGCAGTTCATGGCGAGGTTGCATATGCATTGTGGGGTGATGG
AGAGAAGCTTGAGACTGCTGCTGGTGAGGCTTTAACTTCTGAAAAGAATCATGAATTGCC
AACTGTGGAACTTATAAAAAGTTTGAGTGATGCTCCATTAATCGATTGA
>GmAMT2.3
ATGGCCACTCCCACAGCATACCAAGAACACCTCCCTGCATCCCCCCACTGGCTAAACAAA
GGGGACAACGCATGGCAGCTGACAGCAGCCACTCTCGTAGGTCTCCAAAGCATGCCGG
GTCTGGTGATCCTCTACGCCAGCATGGTGAAGAAATGGGCCGTGAACTCTGCATTCA

TGGCCCTCTACGCCTTTGCAGCAGTCCTAATATGCTGGGTGCTCGTTTGTCACCGAATGG
CCTTCGGTGACAAACTCCTTCCCTTCTGGGGGAAGGGCGCCCCAGCACTAGGCCAGAA
GTTTTTAACACACCGCGCCAAAGTCCCCGAAAGCACGCACTATTATAACAATGGTACGGT
CGAAAGCGCGACTTCGGAACCGTTGTTTGCCACGGCTTCTCTTGTGTATTTTCAATTCAC
GTTTGCGGCTATCACGCTTATCTTGTTGGCGGGATCGGTGCTAGGAAGAATGAACATCAA
GGCTTGGATGGCTTTTGTGCCTCTTTGGCTGATTTTTTCGTACACTGTTGGGGCGTTTAG
TCTTTGGGGTGGTGGCTTTCTCTACCATTGGGGGGTTATTGATTACTCTGGTGGATATGTT
ATTCATCTTTCCTCTGGAATTGCTGGTTTCACTGCTGCTTACTGGGTTGGGCCAAGGTTG
AAGAGTGACAGGGAGAGGTTCCCACCGAACAACGTATTGCTGATGCTTGCGGGTGCAGG
GTTACTATGGATGGGGTGGTCAGGGTTCAATGGTGGAGCACCATATGCAGCCAACATTGA
CTCTTCCATTGCTGTGCTCAACACTAACATTTGTGCTGCCACTAGCCTCCTTGTATGGACT
TCTCTTGATGTAATCTTCTTCGGCAAGCCTTCGGTGATTGGTGCTGTTCAGGGCATGATG
ACTGGACTCGTTTGCATCACCCCAGGAGCAGGGCTTGTGCAATCTTGGGCGGCTATAGT
GATGGGAATTCTATCAGGGAGCATTCCATGGGTGACCATGATGATCCTCCACAAGAAGTC
AAGCCTTCTACAGAAGGTAGATGATACCTTAGGAGTGTTCCACACACACGCTGTGGCTGG
CCTATTGGGTGGCCTCCTCACAGGTCTCTTAGCAGAACCTCAGCTTTGTAGGCTCATATT
GCCAGTGACCAACTCAAGGGGTGCCTTCTATGGTGGCAGTGGTGGCATTCAACTTTTTAA
GCAATTAGTGGCAGCATGTTTTATAGCTGGATGGAACTTGGTCTCGACCACACTGATTCTT
CTGACCATACAGTTGTTCATACCCTTGAGGATGCCAACCGAGCAGCTCGAGATCGGCGA
CGATGCCATCCACGGCGAAGAAGCATATGCTCTTTGGGGTGATGGTGAAAAATATGACCC
AACCAGGCATGGATCCTCAAGAGTGGAAAAAACTTCAGCTTCGCCCCGTGTTAATGTAAC
TGGAGCAAGGGGTGTGACCATAGATTTATGA
>GmAMT2.1
ATGGCTACACCCTTGGCCTACCAAGAGCACCTTCCGGCGGCACCCGAATGGCTGAACAA
AGGTGACAACGCATGGCAGCTAACAGCAGCCACCCTCGTCGGTCTTCAAAGCATGCCGG
GTCTCGTGATCCTCTACGCCAGCATAGTGAAGAAAAAATGGGCAGTGAACTCAGCTTTCA
TGGCTCTCTACGCCTTTGCGGCGGTTCTAATATGTTGGGTGCTTGTGTGTTACCGAATGG
CCTTTGGAGAAGAACTTTTCCCCTTCTGGGGAAAGGGTGCTCCAGCACTAGGCCAGAAG
TTCCTCACGAAAAGAGCCATAGTCATTGAAACCATCCACCACTTTGATAATGGCACTGTTG
AATCACCTCCTGAGGAACCCTTTTACCCTATGGCCTCGCTTGTGTATTTCCAATTCACTTTT
GCTGCTATTACTCTTATTTTGTTGGCTGGCTCTGTCCTTGGCCGAATGAACATCAAGGCTT
GGATGGCTTTTGTGCCTCTTTGGTTGATCTTTTCCTACACAGTCGGGGCTTTTAGTCTTTG
GGGTGGTGGCTTTCTCTACCAATGGGGCGTTATTGATTATTCTGGCGGCTATGTCATACAC
CTTTCTTCTGGAATCGCTGGCTTCACTGCTGCTTACTGGGTTGGACCAAGGTTGAAGAGT
GATAGGGAGAGGTTCCCACCAAACAATGTGCTTCTCATGCTTGCTGGTGCTGGGTTGTTG
TGGATGGGGTGGTCAGGGTTCAACGGTGGAGCACCATATGCTGCAAACATTGCATCTTCA
ATTGCGGTGTTGAACACAAACATTTGTGCAGCCACTAGCCTCCTTGTGTGGACAACTTTG
GATGTCATTTTTTTTGGGAAACCTTCGGTGATTGGAGCTGTGCAGGGCATGATGACTGGA
CTTGTATGCATCACCCCAGGGGCAGGGCTTGTGCAATCATGGGCTGCTATAGTGATGGGA
ATATTATCTGGGAGCATTCCATGGGTGACTATGATGATTTTGCATAAAAAGTCAACTTTGCT
ACAGAAGGTAGATGACACCCTTGGTGTGTTTCACACACATGCTGTGGCTGGCCTTTTGG
GTGGTCTCCTCACAGGTCTATTAGCAGAACCAGCCCTTTGTAGACTTCTATTGCCAGTAAC
AAATTCAAGGGGTGCATTCTATGGTGGAGGTGGTGGTGTGCAGTTCTTCAAGCAATTGGT
GGCGGCCATGTTTGTTATTGGATGGAACTTGGTGTCCACCACCATTATTCTCCTTGTCATA

AAATTGTTCATACCCTTGAGGATGCCGGACGAGCAGCTGGAAATCGGTGACGACGCCGT
CCACGGTGAGGAAGCTTATGCCCTTTGGGGTGATGGAGAAAAATATGACCCAACTAGGCA
TGGTTCCTTGCAAAGTGGCAACACTACTGTCTCACCTTATGTTAATGGTGCAAGAGGGGT
GACTATAAACTTATGA

>GmAMT4.1

ATGCTGGCAGGGGCAGGGCTGCTGTGGATGGGGTGGACGGGCTTCAACGGAGGAGAT
CCATACACAGTAAACTCGGATGCTTCCCTTGCCGTCCTCAACACCCATGCTTGCACTGCT
ACCAGCTTGCTCACTTGGGTCATCCTTGATGTCCTCTTTTTCCGAAAGCCTTCTGTCATTG
GAGCTGTTCAAGGCATGATCACTGGCTTGGTTTGCATAACCCCTGCTGCAGGTGTTGTG
GAGGGATGGGCAGCACTTATAATGGGAGTGTTATCTGGCTCAATTCCATGGTTCACCATG
ATGGTGGTCCATAAGAGATCAAAGCTACTGCAGAAGGTTGATGATACAATGGCAGTGTTC
CACACTCATGCCATTGCAGGAACCCTAGGAGGACTCCTCACTGGACTCTTTGCTGATCCA
AGACTTAACAAATTGTTCTATGGAACTGATGGTCAATACGTTGGTTTCTTTTATGGTTTAGG
TTCGAAGAAGGTCAGTACCGGGTTCAGGCAAATGGGGGTCCAAATCCTTGGGATTATCTT
TGTTATCTTTGTCAATGTTATCAGCACAAGTTTGATTTGCCTCTTCATTAGAATTTTTGTTCC
GTTGAGAATGTCTGAAGAGGATATGGAGATAGGTGATGAGGCTGCTCATGGGGAAGAAG
CTTATGCAATCTGGGGTCAGGGTGATAAGCTTGAAAACTCAAGTTCTAAGTATGGAAGTTC
ATTATATGATGATGTTGAAGCTGGTGGCCCCAAGAAGAAGCGTGGCGGCACGGTTGAGAT
GATGTCGAACTAA

>GmAMT3.1

ATGGCTACTCCTCCGGTGAATTCGTTGCCAATAGCATACCAAGCATGGACAAGCCTGGGA
GTCCCAGATTGGTTGAACAAAGGAGACAACGCATGGCAAATGGTGTCAGCCACCTTGGT
TGGGATTCAAAGCATGCCGGGCCTCGTCATCCTCTACGGAAGCATCGTCAAAAAGAAATG
GGCCGTCAACTCTGCCTTCATGGCCCTCTACGCTTTTGCGGCGGTTATCATCTGTTGGGT
GGCGTGGGCCTACAAGATGTCGTTTGGGGAGGAGCTGCTGCCGTTTTGGGGCAAAGCC
GGGCCCGCGTTGGGCCAGAGGTTTCTGATAAAGCAGGCGGGCCTGCCCGCCACGCCG
CATTATTTTAGAAACGGTGGTGGTTTGGAGACGGCGGAGATAACGCCGTTTTATCCGATG
GGTACTATGGTATGGTTTCAGTGTGTTTTTGCGGCTATTGCGGTTGTTATATTGGCTGGGT
CGGTGCTGGCTAGGATGAACTTCAAGGCGTGGATGATGTTTGTGCCGCTTTGGCTTACTT
TCTCTTACACCATTGGGGCGTTTAGCTTGTGGGGTGGAGGGTTCTTGTTCCACTGGGGC
GTTATGGACTACTCTGGTGGTTATGTTATTCATCTCTCTTCCGGGATTGCTGGCTTCACTG
CTGCTTATTGGGTGGGGCCAAGATCGAAGAAGGACAGAGAGAGATTCCCGCCAAACAAT
GTGTTGCTAACGTTGGCGGGGGCAGGGCTACTATGGATGGGATGGGCAGGTTTCAACGG
CGGAGATCCGTACGCGGCAAACACGGACTCGTCGATGGCGGTGCTTAACACCAACATTT
GCGCCGCCACCAGCCTCCTCGTATGGACGTGGTTGGACGTTATTTTCTTCAAGAAACCCT
CAGTTATTGGAGCCGTTCAGGGCATGATAACTGGCCTTGTTTGCATCACTCCCGGAGCTG
GTCTGGTTCAAGGATGGGCTGCCATAGTGATGGGAGTTCTTTCAGGCAGTGTCCCATGG
TTCAGCATGATGGTATTAGGGAAAAAGCTGAAATTGTTTCAAATGGTTGATGACACCCTTG
CTGTGTTCCACACTCATGCTGTGGCTGGCCTTCTTGGAGGCATACTCACTGGCCTATTTG
CCGAACCTCGTCTGTGTGCACTCTTTCTACCTGTCACCAACTCCAAAGGAGGAGTCTATG
GAGGCCCTGGTGGAGTCCAAATCCTTAAACAAATCGTGGGAGCTTTGTTCATCATTGGGT
GGAACCTTGTGGTCACTTCAATTATTTGTGTGGTTATTAGTTTCATAGTTCCACTTAGAATG
ACAGAGGAAGAGCTTCTCATTGGAGATGATGCGGTTCATGGGGAAGAGGCTTATGCTCT
GTGGGGTGATGGAGAGAAACTTAGCATCTACAAAGATGATACCACTCACCATGGAGTTGT

GTCTAGTGGTGCCACTCAAGTGGTCTAG
>GmAMT4.3
ATGGAGTTTCCTACGAACCTTTTGCCAGACGAAGCGAGCCCAGAATGGATGAACAAAGG
AGACAATGCATGGCAGCTTATGGCAGCAACTGTGGTGGGTCTCCAAAGCATTCCGGGGC
TGGTGATTCTCTACGGAAGCCTAGTGAAAAAAACATGGGCCATTAACTCTGCTTTCATGGC
CTTCTACGCCTTCGCGGGGGTACTTCTATGCTGGGTGGGGTGGGGATTCCGCATGTCAT
TCGGCGAGAAAATGGTGTTCTTCTTGGGGAAACCCGGGGTGGCGGTGGACGAGAAGTT
TCTTCTGGGGAAAGCTTTTCTGGGGCTCTTTCCCAATGCTACGATGGTGTTTTTTCAGGG
GGTGTTTGCGGGGATCACCTTGATCTTGATAGCTGGGGCGTTGCTCGGGAGGATGAACT
TTCGTGCGTGGATGCTGTTTGTGCCTCTTTGGCTTACTTTTTCGTACACCGTCACTGCCTT
TAGCATTTGGTGCCCCGATGGCTGGTTGGCAAAGCTTGGAGTTATTGATTTCTCTGGCGG
ATATGTTATTCACCTCTCCGCCGGAGTTGCTGGTTTTACTGCAGCTTATTGGGTGGGTCCA
CGATCTGAAAAGGACAGAGAAATATTTGCATCAAACAACATGATTGTGGTGCTTGCAGGC
GCAGGCCTGCTTTGGATGGGTTGGAGTGGGTTCAACGGTGGAGGTCCGTTCGTGGCGA
GCACGGTGGCATCTCTGGCCGTCCTTAACACACACGTGTGTGCTGCTGCTAGCATCATC
GTCTGGGTCCTACTCGACACTTTCTACTTTGGTAAGCCCACTGTGTTTGGCGCCGTACAG
GGCATGATCACTGGTCTCGTTTGCATCACACCTGCCGCAGGAGTTGTGCAAGGGTGGGC
AGCGATCTTGATGGGTGTGATGTCAGGAAGCATTCCCTGGTACACAATGATGATCCTCCA
TGACAAGCTCCCATTCCTAAAGCAAATCGACGACCCCATGGCGGTGTTCCACACCCATGC
CGTCGCCGGCGCTCTCGGCGGCGTCCTCACGGGGCTCTTGGCGGTGCCTAAACTGTGC
CGTCTCTTCTACATGGTCCCCGATTGGGAAAAATACATTGGACTCGCCTATGGCCTCCAG
AACGGTGCAACTCATGCGGGTCTAAGGCAAATGGCCATCCAAGTTGGGGCCATAGTTTTC
GTTATTATCTTCAACTTTGTTACGACCAGCTTGATTTGCTTGCTCGTGGGGTCCATAGTAC
CACTCAGGATTGACACCGATGCTTTGCAAATGGGTGACAAGGCAATGCATGGAGAAGAG
GCTTTCGCTTTCTATGGAGGAGACCCTACAGCAAAGTTTGAGAACCTCAAGCACAATAAA
GTTTATGATACTCAAGACTTTTCGTTTGTTAGAGACACAAGGTCATTTGCCCAGCTTCAAAT
GGTATAA
>GmAMT2.2
ATGGCCACACCCTTGGCCTACCAAGAGCACCTTCCGGCGGCACCCAGTTGGCTGAACAA
AGGTGACAACGCATGGCAGTTAACAGCAGCCACCCTCGTTGGTCTTCAAAGCATGCCGG
GTCTCGTGATCCTCTACGCAAGCATAGTGAAGAAGAAATGGGCAGTGAATTCAGCTTTCA
TGGCTCTCTATGCCTTTGCAGCAGTTCTAATATGTTGGGTGCTTGTGTGTTACCGCATGGC
CTTTGGAGAAGAACTTTTACCCTTCTGGGGTAAGGGTGCTCCAGCACTAGGCCAGAAGTT
CCTCACAAAACGAGCCGTAGTCAATGAAACCATCCACCACTTTCATAATGGCACTGTTGAA
TCCCCTCCAGAAGAACCCTTTTACCCTATGGCCTCACTTGTGTATTTCCAATTCACTTTTG
CTGCTATTACCCTTATTTTGTTGGCTGGCTCTGTCCTCGGCCGAATGAACATCAAGGCTTG
GATGGCTTTTGTGCCTCTTTGGTTGATCTTTTCCTACACTGTTGGGGCTTTTAGTCTTTGG
GGTGGTGGCTTTCTCTACCAATGGGGGGTTATTGACTATTCTGGCGGCTATGTCATCCAC
CTTTCTTCTGGAATCGCTGGTTTAACTGCTGCTTACTGGGTTGGACCAAGGTTGAAGAGT
GACAGGGAGAGGTTTCCACCAAACAACGTGCTACTTATGCTTGCTGGTGCTGGGTTGTT
GTGGATGGGGTGGTCAGGGTTCAACGGTGGAGCACCATATGCTGCAAACATTGCATCTT
CAATTGCGGTGTTGAACACAAACATTTGTGCAGCCACTAGCCTCCTTGTGTGGACAACTT
TGGATGTCATTTTCTTTGGGAAACCTTCGGTGATTGGAGCTGTGCAGGGCATGATGACTG
GACTTGTATGCATCACCCCAGGAGCAGGGCTTGTGCAATCGTGGGCTGCTATACTGATGG

GAATATTATCTGGGAGCATTCCATGGGTGACCATGATGATTTTGCATAAAAAGTCAACCTTG
CTACAAAAGGTGGATGACACCCTTGGTGTGTTTCACACACATGCTGTGGCTGGCCTTTTG
GGTGGTCTCCTCACAGGTCTATTAGCAGAACCAGCCCTTTGTAGACTACTATTGCCAGTTA
CCAACTCAAGGGGTGCATTCTATGGTGGTGGTGGTGGTATGCAGTTCTTCAAGCAATTGG
TGGCGGCCATGTTTGTCATTGGATGGAACTTGGTGTCCACCACCATCATTCTCCTTGTCAT
AAAATTGTTCATACCCTTGAGGATGCCGGATGAGCAGCTGGAAATCGGCGACGACGCCG
TCCACGGCGAGGAAGCTTATGCCCTCTGGGGTGATGGAGAAAAATATGACCCAACTAGG
CATGGTTCCTTGCAAAGTGGCAACACTTTTGTGTCACCTTATGTTAATGGTGCAAGAGGG
GTGACCATAAACTTATGA
