# Supplementary material for: Genome-Wide Identification and Expression Analysis of the Ammonium Transporter Family Genes in Soybean
Source: Int J Mol Sci. 2023 Feb 16;24(4):3991. doi: 10.3390/ijms24043991 (PMC9960152; doi:10.3390/ijms24043991)
Supplement: Supplementary file 1 [file ijms-24-03991-s001.zip › Supplementary Data3.pdf]

Supplementary Data 3.

The genomic sequences of *AMTs* in *Arabidopsis* and soybean.

>AtAMT2
CACACCTACACAAAAAAAATTCGTACCATTGACTATAAACATCACAAACCCAACAAAAAT
TTCATCAACTCCGGGAAAGATAGAATAACAAAATGGCCGGAGCTTACGATCCAAGCTTGC
CGGAGGTTCCTGAATGGCTCAACAAAGGAGACAACGCGTGGCAGCTCACGGCAGCGAC
TC
TGGTTGGTCTACAGAGTATGCCAGGTCTTGTTATCCTCTATGCCTCCATCGTCAAGAAGA
AATGGGCTGTGAATTCAGCTTTTATGGCTCTTTACGCTTTCGCCGCCGTTCTTCTCTGTT
GGGTTCTCCTCTGTTACAAAATGGCTTTTGGAGAAGAGCTTTTGCCGTTTTGGGGCAAAG
GTGGTCCAGCTTTCGACCAAGGATACCTTAAGGGACAAGCAAAGGTCTTGAATTTGGTTA
TGCACACAAAGTGTTCGATAAAATGCCTGAATGAGAATTTGATTTGATATTTTTTTCTTA
CAGATCCCAAATAGTAATGTGGCGGCGCCGTATTTTCCGATGGCGACGTTGGTGTATTTT
CAGTTCACATTCGCGGCGATAACGACGATACTTGTGGCGGGATCTGTGTTGGGGAGGAT
G
AATATTAAAGCATGGATGGCTTTTGTGCCATTGTGGTTGATCTTTAGCTACACAGTTGGA
GCTTATAGTATATGGGGAGGTGGGTTTCTGTATCAGTGGGGAGTTATTGATTATTCCGGC
GGTTATGTTATTCATCTCTCCTCCGGTGTTGCCGGTTTCGTCGCTGCTTACTGGGTACGT
TTATTACATAAAATATTCAAGTTCAGAGTTTCTTTAGTTTGTTAGAGGTTAGTTTATTTA
GTTAGGGAAACTATTAAATTCTCAGGCTATTGTTTGATATTTGGGTATTATTACTAGAAA
TATAATGAAAGATTGAAGAAGATGTTTGTTTGTTGAAAATATTGGAATTAATTAATGTAC
GGTAGTCGGTAGATGACATAGTCTCAAAAAAAATTCTTAAGAAATAATTCGATGTAGACT
GGTCTTTAATTGCCAAGTTTTGTTTTGCTTTCTCTTTCGGTATGATTACGTATTTAAGGT
CCATAGTTTATAGATAAGTAGTAAGTTCTATGATTGTTTGGTCCACTAACAAAAGGTTAC
TAATTTCCAAGTACTAAGATTAATTAAATCAAATCAATTTTGGATTTGACAACACGATCT
TGATCTATATTGTATTAGCAAATTAGATAATTTAGTTAGAGGAGAGTAATCGAATAAAGT
AACGCTCCAATATTGATCACACTCAACTGAAATATCTATTTTTGCCTAATAGTAAATTCA
CAAAATAATTGAGGTTGATTCCATTTTTGCATTTTATATCTTATAGATATGTATACACTA
ATTAATTTGTTTTTCTTGTATTCTGCATTTTGTCGCACATAAGCAAAATGGTAAAAGTCA
AGTCAATTCCACTGACGACGACATAATATTCCACATAGTAAATATATAAAATCATGCAAA
AGAAAATGAAATTAAATGGTTAGTTAAAAAGCAAACACTAGCTAGTTGAGAAATACGATT
TTCTGACATGATGTTATTATAGGTAGGACCAAGGCCTAAGGCTGACAGAGAGAGATTCCC
ACCGAACAATGTTCTTCTAATGCTTGCTGGAGCTGGACTTTTATGGATGGGATGGTCCGG
TTTTAACGGTGGTGCTCCTTACGCGGCCAACTTAACCTCCTCTATCGCCGTGTTAAACAC
CAACCTCTCGGCCGCCACAAGCCTCCTTGTATGGACTACACTTGATGTCATCTTCTTTGG
CAAACCTTCTGTCATCGGAGCAATTCAAGGCATGGTTACTGGCTTAGCCGGCGTCACTCC
CGGAGCAGGTTTCAGTCATCTTCTTTTCAAGTTTTTTGTTTACTCTAAGTCTCTAAGTTT
ATTTATGATTAAGGTTGAATTATGTGGAAACTTATTAATATTGCATTGTTAATTTATTAT
ACTGACAAATAAGATATCTATTGCAGGTTTGATCCAAACATGGGCAGCTATAATAATTGG
AGTAGTCTCAGGAACAGCTCCATGGGCCTCTATGATGATCATTCACAAGAAATCCGCTCT
CCTTCAAAAGGTAATTTAGATCATAAATCCCATTTTAATTTCATAACCAACAAAAAAAAA
AAAAAAATGTTTTACCTTATTAGAAAAACGAAAATGTTTCAATTGTAGAATTAAGGTATT
AGTAATAACAAACGAATGATTTGAACTGTAATATTTTGATTGGGTTGAAATAAACAGGTG
GATGATACATTAGCGGTGTTTTACACACACGCCGTGGCTGGTTTACTTGGTGGAATAATG

ACAGGGTTGTTTGCACACCCTGATCTCTGCGTTTTGGTACTTCCTCTCCCAGCGACCAGA
GGAGCTTTCTACGGTGGCAATGGCGGCAAACAGCTTTTGAAACAGTTGGCTGGAGCTGC
C
TTCATTGCCGTCTGGAATGTGGTGTCGACTACTATCATTCTACTCGCTATTAGGGTGTTC
ATACCATTGAGAATGGCTGAGGAAGAGCTCGGGATTGGAGACGACGCAGCACATGGGGA
A
GAAGCTTATGCTCTTTGGGGAGATGGAGAGAAGTTTGATGCTACAAGGCATGTGCAACAG
TTTGAGAGAGATCAAGAAGCTGCTCATCCTTCTTATGTTCATGGTGCTAGAGGTGTCACC
ATTGTTCTATGATTTTTCTTCTTCTTTTTCTTATTTATTTTTTGTGTATTGATTTCCTTA
TGACATTAGAGATGTGAATGAGTTATTTGTGTTCTGTATAGGTGGTAAAACTTCACTTTT
CATATTTTTTGGTGTGTATTTGGAGTTAACAACCATTTGTGATTTTGTTGAAATGAAAAC
ATAGAGTTCGTGAACAAATGTACCTTGTA
>AtAMT1.2
ACTTAAGCAAACACGTTCCACAATCAAGTACCCTCTCTCTATCTCTCCCTCCCTCCCTCT
CCACCATGGACACCGCAACCACCACATGCTCTGCCGTAGATCTATCTGCCCTCCTATCCT
CTTCTTCTAACTCAACATCTTCCCTCGCCGCGGCAACCTTTTTATGTTCCCAAATTTCAA
ACATCTCCAACAAACTCTCCGACACAACTTATGCCGTCGACAACACGTATCTCCTCTTCT
CCGCCTACCTTGTCTTTGCCATGCAGCTCGGTTTCGCTATGCTTTGTGCTGGATCAGTCC
GAGCCAAGAACACTATGAACATCATGCTTACCAATGTCCTTGATGCTGCCGCTGGAGCCA
TCTCTTACTACCTCTTCGGATTCGCATTCGCCTTTGGTACACCTTCCAACGGATTCATCG
GTCGCCACCATAGCTTCTTCGCTTTAAGCTCTTACCCTGAACGCCCCGGCTCCGACTTCA
GCTTTTTCCTCTACCAATGGGCTTTTGCCATAGCCGCGGCCGGAATCACTAGCGGTTCCA
TCGCCGAGCGAACGCAATTCGTTGCTTACCTTATCTACTCTACTTTCTTGACCGGTTTTG
TTTACCCGACAGTCTCGCACTGGTTCTGGTCAAGTGATGGATGGGCTAGCGCGTCCCGG
T
CTGACAACAATCTCTTGTTTGGCTCAGGTGCTATTGATTTCGCAGGTTCAGGAGTTGTTC
ACATGGTAGGTGGAATTGCCGGTTTATGTGGAGCGTTAGTTGAAGGACCAAGAATAGGTA
GATTTGACCGGTCAGGCCGGTCCGTGGCTTTACGTGGTCACAGTGCATCCCTTGTCGTG
C
TTGGTACCTTCTTGTTGTGGTTTGGATGGTATGGGTTTAACCCTGGTTCCTTTTTAACCA
TTCTTAAAGGCTACGACAAGTCTCGGCCATATTATGGTCAATGGAGCGCTGTAGGTCGCA
CCGCGGTCACCACAACGCTTTCTGGCTGCACCGCTGCGTTGACTACTCTATTCAGTAAAC
GGCTTTTAGCAGGTCATTGGAACGTTATTGACGTATGCAACGGACTTCTAGGCGGCTTTG
CAGCTATAACCTCCGGATGTGCCGTGGTGGAGCCGTGGGCTGCTATAGTATGTGGCTTTG
TGGCATCATGGGTTTTAATCGGATTTAACTTGCTTGCCAAGAAACTTAAATATGATGACC
CACTCGAGGCTGCTCAGCTCCACGGTGGATGTGGAGCATGGGGATTAATCTTTACCGGG
C
TGTTCGCAAGGAAAGAATACGTTAACGAGATTTACTCCGGTGATAGGCCTTACGGACTGT
TCATGGGCGGGGGAGGAAAACTGCTCGCCGCGCAGATCGTTCAGATTATTGTGATCGTT
G
GGTGGGTGACGGTAACTATGGGACCGTTGTTTTATGGGTTACATAAGATGAATCTTTTGA
GGATATCAGCAGAAGATGAGATGGCAGGAATGGACATGACACGTCATGGAGGATTTGCTT
ACGCATACAATGACGAAGACGACGTGTCGACTAAACCATGGGGTCATTTCGCTGGAAGA
G

TGGAGCCTACAAGCCGGAGCTCGACTCCTACACCGACCTTGACTGTTTGATACTTTGATT
GGAGAATTGAGTGGTCCCAAACGAGTCAGTTTTAATGTGGTGAAGACAAGAGTTCGGGC
A
CCAAACATGTTGGACGCATCTTTGTGTATTATTGGTCTTCTTCTTCTTCTTTTTTTTTCT
CTTGGTTATCGCTCTGTTGTGGACAGATAGTGTGGAACTGTTAACAATAACATGATCAGT
ATGTCTTTTTAATTAAAGTGAACGTTTGGTATCAAAATTAAACATTGGAATTTGAGCGGT
C
>AtAMT1.5
ATGTCAGGAGCTATTACTTGCTCTGCGGCTGATCTCTCAGCCCTACTCGGCCCAAATGCC
ACGGCAGCGGCTGACTACATTTGCGGCCAGTTGGGTTCCGTTAACAACAAGTTTACCGAT
GCAGCCTACGCTATAGACAACACGTACCTCCTCTTCTCTGCCTATCTTGTCTTTGCGATG
CAGCTCGGCTTCGCTATGCTTTGTGCTGGCTCCGTTAGAGCTAAGAACACGATGAACATC
ATGCTCACTAATGTCCTTGATGCTGCAGCCGGAGGACTCTTCTACTACCTCTTTGGTTAT
GCATTTGCCTTTGGTGAATCCTCCGATGGATTCATTGGAAGACACAACTTTGGTCTTCAA
AACTTTCCGACTCTCACCTCGGATTACTCCTTCTTCCTCTACCAATGGGCGTTTGCAATC
GCAGCCGCTGGAATCACCAGCGGCTCCATTGCCGAGAGGACTAAGTTCGTGGCGTATTT
G
ATATACTCTTCTTTTTTGACCGGGTTTGTTTACCCAGTTGTCTCTCACTGGTTCTGGTCT
CCGGATGGATGGGCTAGTCCCTTCCGTTCAGAAGACCGTTTGTTTGGCACTGGAGCCAT
C
GACTTTGCTGGGTCAGGTGTTGTTCACATGGTTGGTGGTATCGCAGGATTATGGGGTGCC
CTTATTGAAGGCCCTCGGATTGGTCGGTTTCCTGATGGGGGTCATGCTATTGCTCTGCGA
GGCCACTCTGCCTCACTCGTCGTCTTAGGGACCTTCCTTCTCTGGTTTGGTTGGTACGG
G
TTCAACCCTGGTTCCTTCACCAAGATACTCATTCCCTACAATTCTGGTTCCAACTATGGC
CAATGGAGTGGAATAGGCCGCACCGCGGTTACAACTACACTCTCGGGATGCACAGCGGC
T
CTAACCACACTCTTCGGAAAACGTCTCCTATCAGGCCACTGGAACGTAACTGACGTTTGC
AACGGGTTACTCGGAGGGTTTGCGGCCATAACGGCAGGTTGCTCTGTGGTTGATCCATG
G
GCAGCGATCGTATGTGGCTTCGTGGCTTCCCTCGTCCTTATCGGATGCAACAAGCTCGCA
GAGCTCTTAAAATATGACGATCCACTTGAGGCCGCACAACTACACGGAGGGTGTGGTGCT
TGGGGTTTGATATTTGTAGGACTGTTTGCAAAAGAGAAGTATATAAATGAGGTTTACGGC
GCGAGCCCAGGAAGGCACTACGGGCTATTTATGGGCGGAGGAGGGAAGCTATTGGGAG
CA
CAACTGGTTCAAATAATTGTGATTGTTGGATGGGTTAGTGCCACAATGGGAACACTCTTC
TTCATCCTCAAAAAGCTCAATTTGCTTAGGATCTCGGAGCAGCATGAAATGCGAGGAATG
GATTTAGCAGGTCATGGTGGTTTTGCTTATATCTACCATGATAATGATGATGATTCCATT
GGAGTGCCTGGATCTCCAGTACCTCGTGCGCCTAACCCTCCAGCCGTTTGA
>AtAMT1.3
GTATCTCTCTTTCTCTCTCTCAGCTCTCTCAAACATGTCAGGAGCAATAACATGCTCTGC
GGCCGATCTCGCCACCCTACTTGGCCCCAACGCCACGGCGGCGGCCGACTACATTTGC
GG
CCAATTAGGCACCGTTAACAACAAGTTCACCGATGCAGCCTTCGCCATAGACAACACCTA

CCTCCTCTTCTCTGCCTACCTTGTCTTCGCCATGCAGCTCGGCTTCGCTATGCTTTGTGC
TGGTTCTGTTAGAGCCAAGAATACGATGAACATCATGCTTACCAATGTCCTTGACGCTGC
AGCCGGAGGACTCTTCTACTATCTCTTTGGTTACGCCTTTGCCTTTGGAGGATCCTCCGA
AGGGTTCATTGGAAGACACAACTTTGCTCTTAGAGACTTTCCGACTCCCACAGCTGATTA
CTCTTTCTTCCTCTACCAATGGGCGTTCGCAATCGCGGCCGCTGGAATCACAAGTGGTTC
GATCGCAGAGAGGACTCAGTTCGTGGCTTACTTGATATACTCTTCTTTCTTAACCGGATT
TGTTTACCCGGTTGTCTCTCACTGGTTTTGGTCCCCGGATGGATGGGCCAGTCCCTTTCG
TTCAGCGGATGATCGTTTGTTTAGCACCGGAGCCATTGACTTTGCTGGCTCCGGTGTTGT
TCACATGGTTGGTGGCATAGCAGGTTTATGGGGTGCTCTTATTGAAGGTCCTCGTCGTGG
TCGGTTCGAGAAAGGTGGTCGCGCTATTGCTCTGCGCGGCCACTCTGCCTCGCTAGTAG
T
CTTAGGAACCTTCCTCCTATGGTTTGGATGGTATGGTTTCAACCCCGGTTCCTTCACTAA
GATACTCGTTCCGTATAATTCTGGTTCCAACTACGGCCAATGGAGCGGAATCGGCCGTAC
AGCGGTTAACACCACACTCTCAGGATGCACAGCAGCTCTAACCACACTCTTTGGTAAACG
TCTCCTATCAGGCCACTGGAACGTAACGGACGTTTGCAACGGGTTACTCGGTGGGTTTG
C
GGCCATAACCGCAGGTTGCTCCGTCGTAGAGCCATGGGCAGCGATTGTGTGCGGCTTCA
T
GGCTTCTGTCGTCCTTATCGGATGCAACAAGCTCGCGGAGCTTGTACAATATGATGATCC
ACTCGAGGCAGCCCAACTACATGGAGGGTGTGGCGCGTGGGGGTTGATATTCGTAGGAT
T
GTTTGCCAAAGAGAAGTATCTAAACGAGGTTTATGGCGCCACCCCGGGAAGGCCATATGG
ACTATTTATGGGCGGAGGAGGGAAGCTGTTGGGAGCACAATTGGTTCAAATACTTGTGAT
TGTAGGATGGGTTAGTGCCACAATGGGAACACTCTTCTTCATCCTCAAAAGGCTCAATCT
GCTTAGGATCTCGGAGCAGCATGAAATGCAAGGGATGGATATGACACGTCACGGTGGCTT
TGCTTATATCTACCATGATAATGATGATGAGTCTCATAGAGTGGATCCTGGATCTCCTTT
CCCTCGATCAGCTACTCCTCCTCGCGTTTAATTTTCAACTTTTTGGTAATTTATTACCGT
TTAAGTATTGTTTGGGTTTTGGTTTTGAAATATAAATATTTGGATGTTTTGGTTTGTTTT
AAGTGACCTATCGTCTTTTTGTGTTTATAAGTGTTTTAGTTTATGTTTTTTTTTTTTTTC
TTGAATTTTAATTTTACATGCCTCGGCTAATGTTTATGCTATTTCTTAGAAATTTATATA
TACAACTTTTGGTGATCC
>AtAMT1.1
AGCCTCTCTGTTTCATCTTCTTCTCTAAACTCTCAACATGTCTTGCTCGGCCACCGATCT
CGCTGTCCTGTTGGGTCCTAATGCCACGGCGGCGGCCAACTACATCTGTGGCCAGTTAG
G
CGACGTCAACAACAAATTTATCGACACCGCTTTCGCTATAGACAACACTTACCTTCTCTT
CTCCGCCTACCTTGTCTTCTCTATGCAGCTTGGCTTCGCTATGCTCTGTGCCGGTTCCGT
GAGAGCCAAGAATACTATGAACATCATGCTTACCAACGTCCTTGACGCTGCAGCCGGTGG
TCTCTTCTATTATCTGTTTGGCTACGCCTTTGCCTTTGGATCTCCGTCCAATGGTTTCAT
CGGTAAACACTACTTTGGTCTCAAAGACATCCCCACGGCCTCTGCTGACTACTCCAACTT
TCTCTACCAATGGGCCTTTGCAATCGCTGCGGCTGGAATCACAAGTGGCTCGATCGCTGA
ACGGACACAGTTCGTGGCTTACCTAATCTATTCCTCTTTCTTAACCGGGTTTGTTTACCC
GGTCGTCTCTCACTGGTTCTGGTCAGTTGATGGATGGGCCAGCCCGTTCCGTACCGATG
G

AGATTTGCTTTTCAGCACCGGAGCGATAGATTTCGCTGGGTCCGGTGTTGTTCATATGGT
CGGAGGTATCGCTGGACTCTGGGGTGCGCTCATCGAAGGTCCACGACTTGGCCGGTTC
GA
TAACGGAGGCCGTGCCATCGCTCTTCGTGGCCACTCGGCGTCACTTGTTGTCCTTGGAA
C
ATTCCTCCTCTGGTTTGGATGGTACGGATTTAACCCCGGTTCCTTCAACAAGATCCTAGT
CACGTACGAGACAGGCACATACAACGGCCAGTGGAGCGCGGTCGGACGGACAGCTGTC
AC
AACAACGTTAGCTGGCTGCACCGCGGCGCTGACAACCCTATTTGGGAAACGTCTACTCT
C
GGGACATTGGAACGTCACTGATGTATGCAACGGCCTCCTCGGAGGGTTTGCAGCCATAA
C
TGGTGGCTGCTCTGTCGTTGAGCCATGGGCTGCGATCATCTGCGGGTTCGTGGCGGCC
CT
AGTCCTCCTCGGATGCAACAAGCTCGCTGAGAAGCTCAAATACGACGACCCTCTTGAGG
C
AGCACAACTACACGGTGGTTGCGGTGCGTGGGGACTAATATTCACGGCTCTCTTCGCTC
A
AGAAAAGTACTTGAACCAGATTTACGGCAACAAACCCGGAAGGCCACACGGTTTGTTTAT
GGGCGGTGGAGGAAAACTACTTGGAGCTCAGCTGATTCAGATCATTGTGATCACGGGTT
G
GGTAAGTGCGACCATGGGGACACTTTTCTTCATCCTCAAGAAAATGAAATTGTTGCGGAT
ATCGTCCGAGGATGAGATGGCCGGTATGGATATGACCAGGCACGGTGGTTTTGCTTATAT
GTACTTTGATGATGATGAGTCTCACAAAGCCATTCAGCTTAGGAGAGTTGAGCCACGATC
TCCTTCTCCTTCTGGTGCTAATACTACACCTACTCCGGTTTGATTTGGATTTTTACTTTT
ATTCTCTATTTTCTAGAGTATTATTTTAAATGATGTTTTGTGATACTTAAATATTGTTTT
GGATATTTTTTTGCATTTCAGTAATGTTTTAGATGTACAGTTTCATGGGGTTGTGATGAT
AATATCTATGTGGTCATTTGTGTTCTCTTTGGAGTTTTTTCTATAACGCTTTTTTC
>AtAMT1.4
ATGGCGTCGGCTCTCTCTTGCTCTGCCTCTGATCTGATTCCATTACTATCAGGTGGAGCC
AACGCCACCGCAGCAGCAGCCGCCGCTGAATACATCTGCGGGAGATTCGACACAGTCG
CC
GGGAAATTCACTGATGCGGCTTACGCAATCGACAACACTTACCTTCTCTTCTCTGCTTAC
CTCGTTTTCGCGATGCAGCTCGGTTTCGCCATGCTCTGTGCCGGATCCGTACGTGCAAA
A
AACACGATGAACATTATGCTCACGAACGTCATCGACGCTGCAGCCGGAGGTCTCTTCTAT
TATCTCTTCGGTTTCGCTTTTGCTTTTGGATCTCCTTCTAATGGATTCATCGGAAAACAT
TTCTTTGGAATGTATGATTTTCCTCAACCTACGTTTGATTATCCTTATTTTCTATATCAA
TGGACTTTCGCTATCGCCGCCGCTGGAATCACGAGTGGTTCGATAGCGGAGAGGACTCA
G
TTCGTTGCGTATTTGATCTATTCTTCTTTCTTGACGGGTCTTGTTTACCCGATTGTGTCG
CATTGGTTTTGGTCTTCTGATGGTTGGGCGTCTCCGGCTAGATCTGAGAACCTTCTGTTT
CAATCAGGTGTGATTGATTTCGCTGGCTCTGGTGTTGTTCATATGGTTGGTGGTATTGCT
GGTTTATGGGGAGCTTTAATTGAAGGACCTAGGATTGGTCGGTTTGGAGTTGGGGGTAAA

CCGGTTACGTTGCGTGGTCATAGTGCTACGTTGGTTGTTCTTGGAACGTTTTTGTTATGG
TTCGGATGGTACGGGTTTAACCCGGGCTCGTTTGCAACTATTTTTAAGGCGTATGGGGAG
ACTCCAGGGAGCTCGTTTTACGGACAATGGAGCGCAGTTGGGAGAACCGCGGTAACAAC
T
ACGTTAGCTGGTTGCACGGCGGCGTTAACGACTCTGTTTGGGAAAAGACTTATTGATGGG
TATTGGAATGTAACTGATGTTTGCAATGGTTTGTTAGGCGGGTTTGCGGCTATAACTAGC
GGATGTTCGGTTGTGGAACCGTGGGCTGCGCTTGTATGTGGGTTTGTAGCCGCATGGGT
G
CTGATGGGATGCAATAGACTAGCGGAAAAGCTCCAATTTGATGATCCGTTGGAAGCGGCT
CAGCTTCACGGTGGTTGTGGTGCGTGGGGGATTATTTTCACCGGGTTGTTCGCGGAGAA
A
AGATACATTGCCGAGATCTTTGGAGGCGACCCGAATAGGCCTTTCGGATTGCTAATGGGA
GGAGGAGGTAGGTTGCTTGCGGCGCACGTCGTTCAGATTTTGGTGATTACGGGTTGGGT
T
AGTGTGACAATGGGGACTCTGTTTTTTATTTTGCATAAGCTGAAACTGTTGAGGATACCG
GCGGAGGATGAGATAGCTGGGGTGGATCCGACGAGTCACGGAGGGTTGGCTTATATGTA
C
ACAGAAGATGAGATTAGGAATGGGATCATGGTTAGGAGAGTGGGTGGTGATAATGATCCC
AATGTAGGTGTTTGA
>GmAMT4.2
GAAGAAGTAAACGGGAGAGATTTTGATAGGGACTAAACATTTGTTTTTTTTTTTTTCCTCAG
GGACTAAAACATGTGTTTAAGTGTAGGAAATAAAACATAAAAATTGTGTCGTTGCGTCGTG
TTGACTTAGCATCACTATTTTGCCTCATACACAACATATTAGAGGATATATAACTCTACTATAG
GTACCATTTTCTTTTTCTGACTGTTATTTGTTTGACGACAATGGTTTTGCATACATGAATATA
TAAGCATTTACTGATCATTTACTATCACGAAAAGCATACTATATATACTAATTACTAATTAATAG
GTCTCGTGCTTGTAGATGGAAGGCTTTGAAGGGCTCCGATGAAGGTGTTCACGACGAGG
TAAATGCTAGATATGTTGTGGCCATAAAAGGTCATTCATATGCCCTATCTAGGTGTTACACAT
AGAAGAGTAAACCTCCCATGACACTTAGTTCTCCTACTTAGCTAGGTCACTCAAACAAAAC
AAAAAAACAAAGAGTGGGAAAAAAATGAATGTGCCATTACCCTCAAACCTAGCGGTTGTT
GATGGAAGCCCTGAATGGTTTAGTAGAGCAGACAATGCATGGAAGCTAATAGCTGCAACC
CTAGTTGGTAACCCTAGTTGGTCTGCAAAGTGTTCTAGGGCTCATAATCTTGTAAGGTGGA
GCAGTGAAGAAGAAATGGGTAGTGAACTCAGCATTCATGTCACTCTATGCTTTTACATGTG
TGTTCTTCTGTTGGGTTGTGTGGGGATACAGAATGTCTTTTGGTGATGAGCTTCTTCCTTT
CTGGGGAAAGCCTGCTATGTCACTTGAGCATGCATATCTTTTCAAGAAGGCATTTTTGGG
GGCCTTCCCAAATGCCACAATGTGTGTGTTTGCTGCCATCACTTTGATTCTCATTGCTGG
GGCCGTGTTGGGGCACATGAACTTCTATGCTTGGATGCTTCCCTGGCCGTTCTCAACAC
CCACGCTTGCTCACTTGGTCATCCTTGATGTCCTCTTTTTCCGAAAGCCTTCTGTAATTGG
AGCTGTTCAAGGCATGATCACTGGTTTGGTTTGCATAACCCCTGCTGCAGGTGTTGTTCA
GGGATGGGCATCACTTATAATGGGACTTCTCTCAGGCTCAATTCCTTGGTTCACCATGATG
GTCATCCATAAGAGATCAAAGCTACTGCAGAAGGTTGATGATACTATGGCAGTGTTCCATA
CTCATGCCATTGCAGAAACCCTAGGAGGACTCCTCACTGGGCTCTTTGCTGATCCAAGAC
TCAACCGGTTGTTCTATGGACATGATGCTCAATACGTTGTTTTCTTTTATGGTTTCGGTTCC
AACAAGCTCAGCACCGGCTTCAGGCAAATGGGAGTCCAAATCCTTGGGATTATCTTTGTT
ATCTTTGTCAACTTCATTCGGATCTTTTTTCCATTGAGAATTTCTGAAGAGGATATGGAGAT

AGGTGATGAGGCTGCTCATGGTGAAGAAGCTTATGCAATCTGGGGTCAGGGTGATAAGC
TTGAAAACTCAAGTTCAAAGTATGGAAGTTCATTATATAATGATGTTGAAGCTGGTGCCCC
CAAGAAGAAGCGTGCTGGCACTGTTCAGATGATGTCCAACTAAATACTATATAGTATGATAT
GTCCTTGTCTTGCTCATGCTCTTGTTTTCAGAGACACGTATTATTAAAGTAAAATAAAAGAT
CGTGTCTCATTGTTGTTTGAGTCCTAGGATACAACAACTTTGTTTATTTTACTTTCCCTATGT
TCGTTCTTCATAACAAATGAATAAGACTAGTAATTAACCTTCAATGCAAATGAAATTTCAGTA
TTAAGTAACCGTCCTATCCTTGTTGTACCCTTACCCTCCCAATCAGAAACAGAACCTTCAAT
GCAAATTAACATCAGATAAATTTATGTGCAACATAGCCATTGAAACCAGATTGTTGAAGAAA
GAGCAGGAAAACTTGCTCTAGTGAAGTATCAGCAAGTGCTTTGGCAGGGAAGGGTATTGA
CTATTGAACTCTCAATTCATTGATGAATTGAATTGAAGACTTGAGAAATCAGGGATGTTGCC
CACTGACTCCTGAAGAGATTGGATTACAAGGCGTGTTCAAAGCTATATACAACTAACTAGT
TCAATGTCTTTATTAGTGCAGCTAGTCTTAAATGTCAATAGT
>GmAMT1.1
CCACTTTCTTTCTCTCTCTCTCCTCCCCCACTTCGATAGCGCACACAATTTGACTTCCATA
GTCCCCACTTTCTCAGTTTCTCTATATATACACACTTCACAGTTCACACTACCCTAATTAAAC
ACCGCGCCCAACCCTTTTCCTCTCCCTTCTCCCACCTCAAACGCCGTCGTTTCGACCAC
CTTCTTCGGTCGCGGCACAACCAATAACCATGTCGCTGCCAGATTGTCCCGCCGTCCAA
CTTGCCCAACTCCTGGGCCCAAATACCACAAACGCTGCCGCCGCCGCCTCCTTCATCTG
CGACCGGTTCACCGCCGTGGACAACAAGTTCGTCGACACGGCCTTCGCGGTCGACAAC
ACTTACCTCCTCTTCTCCGCCTACCTCGTCTTCTCGATGCAGCTCGGCTTCGCCATGCTC
TGCGCCGGCTCCGTCCGCGCCAAGAACACCATGAACATCATGCTCACCAACGTCCTCGA
CGCCGCCGCCGGCGGCCTCTTCTACTACCTCTTCGGCTTCGCCTTCGCCTTCGGCTCCC
CCTCCAACGGCTTCATTGGCAAACACTTCTTCGGCCTCAAGGAACTCCCCTCCCAAAGC
TTCGACTACAGCAACTTTCTCTATCAATGGGCCTTCGCCATCGCCGCCGCCGGCATCACC
AGCGGCTCCATCGCCGAACGCACACAGTTCGTGGCCTATCTCATCTACTCCTCCTTCCTC
ACCGGCTTCGTCTACCCCGTCGTCTCCCACTGGTTCTGGTCCGCAGACGGCTGGGCTTC
TGCCATTTCCCCCGGAGACCGGCTATTTTCCACCGGCGTGATAGACTTCGCCGGCTCCG
GCGTAGTCCACATGGTTGGTGGAGTAGCCGGCTTCTGGGGCGCACTGATAGAAGGCCC
GAGAATCGGACGCTTCGACCACGCGGGACGCGCCGTTGCCCTCAGAGGCCACAGCGC
GTCCTTAGTAGTCCTGGGGACCTTCATGCTTTGGTTCGGTTGGTACGGATTTAACCCTGG
TTCATTTAATAAAATCCTAGTTTCCTACGGTAACTCAGGAACTTACTACGGTCAATGGAGCG
CGGTTGGGAGAACCGCGGTCACCACTACCCTTGCAGGGTCAACTGCTGCGTTGACCAC
TCTCTTCGGGAAACGGATGATATCCGGTCATTGGAACGTGACCGATGTCTGCAACGGGCT
GTTAGGCGGCTTCGCCGCCATAACAGCCGGCTGCTCCGTCGTTGAGCCATGGGCAGCC
ATCGTGTGTGGTTTTGTCGCTTCAGTGGTTCTGATAGCGTGCAACAAATTAGCAGAGAAG
GTTAAGTTCGATGATCCTTTGGAAGCGGCGCAGTTACACGGTGGGTGTGGCGCGTGGG
GGGTGATATTCACGGCGCTGTTCGCGAAAAAGGAGTATGTGAGCCAGGTTTATGGGGAG
GGGAGGGCGCACGGGTTGTTCATGAGGGGTGGAGGGAAGTTGCTGGCGGCGCACGTG
ATTCAGATTTTGGTTATTGTTGGGTGGGTGAGTGCGACCATGGGACCCTTGTTTTGGGGG
TTGAATAAATTGAAATTGTTGAGGATTTCTTCCGAGGATGAGCTTGCGGGGATGGATCTTA
CCCGTCATGGAGGATTTGCTTATGCTTATGAGGATGATGAGTCGCACAAGCATGGGATTC
AGCTGAGGAAGGTTGGGCCCAACGCGTCGTCCACACCCACCACTGATGAATGATTACGA
TCACGATTAATTCGGCCCCGACAGTATTATCTTCAATTGAAATTACGTGTGACTTAGAAGAA
GAAAAAAAGATGATGATGATTTTGTTTGTAATTTATTTTATTTGTTTTGGGTTTTTTTTTTAAT

TTTGTAGATTTTTCTTTTTATGATGGGTAAGTAGGGATTTTAATTTGTAATTGTTATTGGCCG
TATATTGGTAGATGCTGGAAATTGAAGATTCTGCTGGAAGATGCGAACGTTTCTGAAAATG
ATAGATGGCTGTGGAAAATGAAAATATTTTATTTGTGGGATTTAATTTTCGTAGTTTTCGCCA
AAAAAGAAGGAAGAGTCGTATATAGTAGAAATTTTAATTTGAGACTTTGATGT
>GmAMT1.6
CACCAAATTGTTTAAACATAGGTTTGAGGTTTATGAGAGAAAAAGAAGGTTTTTTTGCTCTC
TCTCTTTCAATTTAGAGAGTTATCTGATATTTATAAAAACAAACAAAACAGCAAAACATTAAA
TTTATACAAATAGGGGAGTGACTGAGCTAGAAAAAAAAAAGGAGAGGAAACGTGTCTAAAT
ACGTAGGGCTGAGGAGAAGCCAACGAAAGAATCCCAAACTCAGCAGTGTGTAGTATATCA
ACACAATATTTGAGGAGGATCTATTCGCGCATATGATTTAAATTCGAAGGTTCCCTCTAAAG
CTCTCAAAACCACTAACCTAACAGCATGTGGCGCAATGACTTCCAATTCCCATCATACTCT
TCTCTCTCTTGCTCCGCCAACCACCTTGCACCCCTCTTCAACGACACCGCCGCCGCAAA
CTACCTCTGCGCCCAATTCGATTCCATTTCCAAGGGCCTCGCCGACACCACCTACTTTCT
GTTTTCGCCATGCTCTGCGCCGGCTCCGTCAGAGCCAAGAACACCATGAACATCATGCT
CACCAAAGTCCTCGACGCCGCCGCCGGCGGCCTCTCTTACTACCTCTTCGGCTTCGCAT
TCGCCTTCGGCGCCCTCTCCAACGGCTTCATCGGCCGCCACTTCTTCGGCCTCCGAGAT
TTCCCGATGGGTGCCTCTCCCTCCGGCGACTACAGCTTCTTCCTCTACCAGTGGGCCTT
CGCCATCGCCGCCGCCGGAATCACCAGCGGCACCATCGCCGAGAGAACACAGTTCGTG
GCTTACCTCATCTACTCTTCTTTCTTAACCGGTTTGGTTTACCCCATCGTTTCGCATTGGTT
CTGGTCCTCAGACGGTTGGGCCAGCGCGGCTCGTAGCAACGGAAATGTTTTGTTCGGGT
CTGGAGTCATCGACTTTGCGGGCTCAGGCGTTGTTCACATGGTTGGCGGGATAGCGGGC
ATGTGGGGGGCTTTAATTGAAGGCCCGAGAATCGGCCGGTTCGACCGGACGGACCGGT
CCGTGGCTTTGCGTGGCCACAGCGCGTCTTTAGTTGTGCTTGGTTCGTTTTTGTTGTGGT
TCGGGTGGTACGGCTTCAACCCTGGTTCGTTTATCACTATAGCGAAGGGTATTATGGTCAA
TGGAGCGCGATAGGGAGGACAGCTGTCACGACGGCATTGGCTGGGAGCACTGCGGTTA
TTGGTGGGCCATTGGAACGCGATTGACGTGTGTAACGGCCTGCTTGACGGGTTCGCTGC
GATTACATCGGGCTGTGCCGTAGTAGAACCGTGGGCCGCGATTGTGTGCGGGTTTGAGG
CGGCGTGGGTTTTGATTGGGCTTAATAAGCTTGCCGCGAAGGTGGAGTACGATGATCCA
CTGGAGGCGGCGCAGCTTCACGGCGGGTGTGGCGCGTGGGGAGTGTTCTTCACGAGA
TTGTTTGCGAAGAGAGTGTACATGGAGGAGATTTGCGGCGTTGGAAGGCCGTTCGGGGC
GTTGATGGGCGGCGCAGGTGATTCAGATATTGGTGGTGTGCGGGTGGGTCACGGCGAC
CATGGCGGCGTTGTTCAAAAGATGAATCTGTTGAGAATTTCGAGGGATGGTGGGTTTGCA
TATGCATATGATAGCTGAAGCAGGGGAGTAGGATTGATGATGCTAATACCACTGCACCACA
AGTCTGATACTTGCTAGCTACTTCATCAAGGAATATGTTTATGCATGCTTTTCTAGCTCTCTA
GTTTCCTATCTATATATTTTATGATGGCAATATCTATTTTTCTATAAGAATAAATTATTTTTTATT
ATGTGAGTGGAGAACTATAATTTAATATTATTGTACATGAAAATTTTACAGCCTAGCTATGGA
AACTTCATAAAAAATATATTTGTTTTGCG
>GmAMT4.4
CCTCCAGAAAACTAGATAAAATTCGAACACGAAGAGTTGAAAGTTGAAACGAAATCATACA
TGAAAGCGATTAGAACTAAGCCCTTAACGTCACAAGTGAATTGCTTATATCCCCCGTCTCA
ATTGCAAATTATTAAATATAAAGAGAAATGCTGGGAATATTTTTCAGATTTAGTTTTTTAATAC
ACTTTTTTATTCGTTGAAATTCATTAAAATTTTATATATAGATCTTACATTATATTAAAAAAGTG
TGTCTAAAAATATGTACGCAATATGAAGTAAAACTTTAACCTGAAACTTCTTTGAAGAAGTG
GCTTAAGATTCTCGAGTTTAGCTTCTATATGACAGAATTTTCAAATGAATCTAATTGCGTCG

ACAGCATAGAATGTCGAATCACCGCAACAAGAAACCACACCTTAGCGATAATTCCAAAGAT
TCCTTAACAAAATGTCCAAAAATGTGTCACTCTTTAAATTCCAAACTCCACCACACCATCCA
GAAAATTTTCGAAATTCTCGAGTGACATTAATTTGGTTTCATTATTATTACTATGAGTTCTAG
TTCTTATGATATCCCTTTGCCATCGTCCCTTTTGCCAAGCGAGGCATCGCCGGAGTGGAA
CAACAAAGCAGACAACGCGTGGCAGCTCACGGCGGCAACCCTAGTGGGCCTCCAGAGC
GTTCCCGGGCTCGTGATCCTATATGGCAGCATGGTAAAGCGCAAATGGGCCGTGAACTC
GGCGTTCATGGCCCTGTACGCCTTCGCGTGTGTGCTCATTTGTTGGGTTTCATGGGCAC
ACCGCATGGCGTTCGGGGCCAGGCTTTTACCCTTTGTGGGAGCGCCCAACCATGCCTTG
GCCGAGAAGTTTCTCCTCGCAAAGTCAACAATTGGGTATTTTCCAATGGCTGACTTTGTG
TTCTACCAGTTTGCTTTTGCCGCAATTACTCTTGTGTTGCTTGCAGGGTCCTTGCTTGGGA
GAATGAACTTCTATGCGTGGATGATGTTTGTCCCTTTGTGGCTTACGTTGTCTTATACCGTT
GGTGCCTTCAGCATATGGGACGATGATGGGTTCCTTCAGGGAAAAATAATTGACTATGCT
GGTGGGTTTGTCATTCATTTGTCTTCTGGCGTTGCGGGTTTCACAGCTGCTTATTGGGTA
CATATATAACATTGAAATTGTAACCTTTAATTTGATTCATTCAGTATTTGTCCAACACCATGG
CTTGAAGAGTCATCATCAAACATCGATCTAATATGGCATGCAGGTTGGTCCAAGAATTTCA
CAGGACAGGCAAAACTTTCCACCAAACAACATAATTCACGTGCTTGGAGGTGCAGGGTTT
TTGTGGATGGGTTGGACAGGTTTCAATGGTGGAGCTCCATTTCAAGTGGGAGAAATCGC
ATCCTTGGCCATTTACAATACCCATCTTTGCACTGCCACAAGCCTACTCGTTTGGCTTACG
CTTGACATGATTGTATATACCAAGAGCTCAGTCATAGGTGCTGTCCAGGGAATGATCACTG
GCCTCGTCTGCATCACACCAGGTGCAGGTAATTAATTAAGCTCACATTGCACACCCCCTC
CTTAAAAAACAAAACTTATTTTAAGAACAAATTTTAGCATATATAATTGTATTAATAAATTAAAA
TATTCTAACAGATTTATAAAATATTAGACCTTACATATGTAAAGTTTAATTTATATATATCATCAA
TGTAAAGTAATTTTACAATTGCATAAAACGTAATTTACCTTTACATTACATCATATTAAAAAATA
AGATAATGTGATAGATGATCAAAACTTATTGGGTGTCTTTATAAAAGTGTTTTACTCTGTATA
AATTCATTAAAAAAAGGGGAAAAGGGAATTCTTTTATCCTCTCGAATTCTATCTTTTCATCAT
ACAAATAATACGGGGTGATTTTAATTTTCCTACACATTTTATCAATATTAAAAAATTTAGACTT
ACAAGATCCAAATAATGTATGGTGCATAACAATTTATAATTAAATATAAAAACTTATATTATAAA
TAAATTCTAATTAAATTTATATAACATTTATTATTGAATTAATAAAATTTATGTAATTCATATTATA
GTATTTAATAGGCAAAATTGCATTTTTGATTCCCCAGTTTATCTCCAGTTTTGGATTTGGTCT
CTCACTAATTTAATTCACGAATTTGATCCTCCTATTTTATAAAATCGTGCAATGTTGGTTCCT
CAAACCACAATTGGATGTTGACAATCAGAACATGATACGTGATAGTCATCATCCAATAAGAA
CGTGACACATAGCAGTCAACATCATTTGTTAACCGTCAACTTTCAAGTGTGGTCTGAGGAA
TCAACATTGCACGATTTTACAAAATAGGAAAATCAAATTGGTGAATTAAATTATTGGTGGAC
CAAATCCGAAACTCGAGATAAGCTAGAGGATCAAATTTACAATTTTGTCTATTTAATAATAAA
TTAATGTTGTACATGCATTTAGTATGATAAACATCAATTAGTACCAATAAGAAAACAATTAGTA
AAAAAATTGTGTTACGCAGTGTTTCAAACATTAATTATACATTTACAAAGGCTTTCCACTTTT
CACTTTTCGAATTGAAAGACATATGTTGTTGGCATTCTAACCTTTTACCTATGTATGTGAAAA
GGCTTGGTGGATCCATGGGCAGCAGTATTGATGGGAGCATTGTCTGGTTCCATTCCATGG
TACACAATGATGGTGCTACACAAAAAATCCGCATTCTTTCAAAGTGTAGATGACACATTAG
GAGTCTTTCACACTCATGCTGTGGCTGGTCTTCTTGGGGGTATCCTTTCTGGCGTGTTTG
CCAAACCCGACCTTCTAAGAATAATGTATCAAGATACAAATTATTGCCCCGGCTTATTCTAC
ACCTTTTTTAAGGGGGAAGTGGACCATGGCTTTAGGCAAATTTGGTACCAATTACTTGGAG
CAGGTTTTATTATTGTTTGGAATGTTGTTATTACTAGCCTCATTTGTATTCTCATAAGCCGCA
TTGTGGATCTTAGGATGAAAGAAGAGGATCTTGAGGTTGGTGATGATGCTGCCCATGGGG

AAGAAGCATATGCATTGTGGGGTGATGGAGAGAGAATGAGGATCCCTCTTCGTCTTCATAT
AGGTCCAACAATTCCTTCCTTGTGCCGAAAAAGGTTTTCAATTCCTCTCACTAGGAAAGAA
GGAGAATAGATGTTGCATACTTGCATTTGCATTTGCCTT
>GmAMT4.5
GAAACTCCTTGTTTAAATTTAAATATTCACAATACAAGAAGCAAGTACAACTCATGATCAGAT
TATTATAGTTTAAATGTTACAACCAATGGCTTGGTTAAAAGATTAATTCACATTACAAGAATC
GACGGGCTTTGTTAGGCCTTGGATTCTCCGTTCTCAAATACGCCAACCTTGTCAATCTCAA
ATTCCAATCATATAATATAGAATATAGAGCTAGCGACACAAATACATATTAATACACTATTGTT
TGCCAAATACTATATAATCGCTCTCTGTTGTTTATTCTGGATCCTTAAACCTCCACTTGAAG
CTACGTGGTTATATATTCTGGGAATACATGCATATATAGAACAGTATATATATCAGAAATGAGT
CACCTACCATCAAACTTGTTGCCCGTTGACGGAAGCCCAGAGTGGATGAACAGGGGTGA
CAACACGTGGCAGCTGACGTCAGCTACTCTGGTGGGCCTGCAAAGCGTTCCGGGCCTG
GTCATCCTCTACGGCAGCATAGTGAAGAAGAAATGGGCTTTGAACTCTGCCTTCATGGCC
CTCTACGCTTTTGCGGCGGTTCTTGTTTGTTGGGTTGGGTGGTGCTACAAAATGTCATTC
GGCGAGAGGATCCTTCCATTTCTGGGACAGCCAAACACGGCCTTGGACCGGGAATTCGT
TTTTCGCAAAACTTTTGCTGGGGAATTTCCCAACGCCACCATGGTGTACTTTCAGCTGGT
TTTTGCTGCCATCACGTTGATTCTCATAGCGGGGGCGTTGCTTGGGAGAATGAACTTTTAT
GCATGGATGTTGTTTGTTCCATTATGGGTTACGTTCTCTTACACCATTTGTGCCTTCAGTAT
ATGGAGCACCGATGGATGGTTGTTCAAGATGGGCCTCATTGATTATTCTGGTGGCTTTGTT
ATTCACCTTTCTTCTGGGGTTGCTGGTTTCGTCGCAGCATGTTGGGTATATAATTTCTAATT
GTTTTGCATCATCTTTCATGCATGATCTCCCTAAATAACTACTACTTCCCTTTCTTATTATAAA
AAATAAGTTATAGTGTAAATAAAAAGGAATAGTATAATTTACTTTTGATTTTTAGGAATACGAG
TATAATTAATGAAGGGTTGTTTGAAAATTATTAAGTGATGCGGAATTATTTGAGGTTAATCAA
TATCCTGAGTTGTAGCTACCAAATTTATATGATGTTTGTCTTCAAAATATTGATTTTAGGTTAC
TTTAGTAATTTTGTGACAAATAAGTTTATTGATGATAACAGATGGTGAATGAAGTGAGGGAT
ATGATTTTGTATGCAGGTAGGACCAAGGGCAGTGAAGGACAGAGAGAGGTTCCCTCCAA
ACAACATACTTCTGATGCTGGCTGGGGCTGGCTTACTATGGATGGGTTGGTCTGGATTCA
ACGGTGGAGATCCATACGCAGCCAGCATAGATGCATCTCTGGCCGTGCTTAACACCCACG
TGTGCACAGCAATGAGCTTGTTGACTTGGCTTTTCCTTGACATCCTCTTCTTTGGAAAACC
TTCCGTCATTGGTGCCACTCAAGGCATGATCACTGGCCTTGTTTGCATCACCCCTGCTGC
CGGTACCATATGCCCACGTAACGTTATGTCACATATTTTACCAAGCATTAACAGCGTTAATA
GTATCTTATATGGTTAACATATAACTTTCCCTCTTAAACATATGGGTGAGAGATTTGATTTAAA
AGTTAATATATATATATAGATAAATATTTATTGAGAGAGATCAACTCTTTAAATAAATGAATTTC
AGTAGTGCCAAGAACTAAAATTTAAGGTTCAGAAAATTGGTGAGTTAAACAAACAAAAATG
ATCCATTTTCCAAGTACTAATAAAAACTACAATTAACCAAGCATCATATTATATATGCTTCTAG
TGTTGTGACTAAAATGAATTTCTTAGGAAATCTGAAACTTGGTTTATTTATTTACTTGAGATT
AGGTGTGGTGCAAGGTTGGGCAGCAATCATAATGGGAATGATGTCCGGAAGCATCCCAT
GGTACACAATGATGGTCCTGCACAAAGAAAGTAAGCTCCTCAAACAAGTTGATGATACTCT
AGCAGTTTTCCACACCCATGCCGTGGCTGGTAGCCTAGGTGGCATTTTGGCTGGTTTTTT
TGCTGACCCTAAGCTTTGTTATCTCTTCTATGGAGTAGAAGATTCCCTACATTATACGGGTC
TTGTTTATGGCATATTCAATGGGAGGCCAAATGCAGGGTTCAGGCAAATGATGGTTCAGCT
TCTTGGCATTGTGTTTGTGATACTTCTCAATGTCACCACAACTAGCATTGTGTGCTTGTTG
GTTAGGATCATAGTTCCACTTAGGCTGAGTGAAGATGACTTGCAAGTTGGGGATGAAGCA
GTCCATGGCGAAGTTGCATATGCATTGTGGGGTGATGGAGAGAAGCTTGAGACTGCTACT

GAGCCTTTGACATCTGAAAAGAATCTTGAATGGCCAACTGTGGAACTAAGAACTTAATTCG
AGTGATGCTCAATTAATGGAATGAACTCAATGAAGGATAAAATATTCTAGCTCTTTTGTTGG
TTTTGTGATTGTTTGAGAGGGAGATATTAAAATAAGGATCAGTAATCTAGTATTACTAGTAAG
AAGAGAATTTGGTTCCTCTGTGATTGTTGTTGCAATGCCAGGATGTCAATTGCAAGATGAC
CCGTTGTAGATTTGCTTAATGAAATAATGATGTTTATTTAAGTAAGCTTGTTTTTGTTAACTTA
TAGTATGCCTTGAACAAACAATGACAAGAAAGTTGCACG
>GmAMT1.2
CTCTTCCCCCAGTCCGATAGCGCACACAATTTGATTTCCATAGTCCCCACTTTCTCTGTTT
CTCTATATATACACACACTTCACAGTTCACACTTCCCTAATTAGCCACCACTCCCAACCCCC
ACCCGTAGTTTCTACCACCTTCAGTCACGGCGTAATACACTAACCAACCCACCATGTCGC
TGCCTGCTTGTCCCGCCGAACAACTGGCCCAACTTCTCGGCCCAAACACCACAGACGCC
TCCGCCGCCGCCTCCCTTATCTGCGGCCAGTTCGCCGCCGTGGACAGCAAGTTCGTCG
ACACGGCCTTCGCCGTCGACAACACCTACCTCCTCTTTTCCGCCTACCTCGTTTTTTCTAT
GCAGCTCGGCTTCGCCATGCTCTGCGCCGGCTCCGTCCGCGCCAAGAACACCATGAAC
ATCATGCTCACCAACGTCCTGGACGCTGCCGCCGGCGGCCTCTTCTACTACCTCTTCGG
CTTCGCCTTCGCTTTCGGCTCCCCCTCCAACGGCTTCATCGGTAAACATTTCTTCGGCCT
CAAGGACATCCCTTCATCCTCCTACGACTACAGCTACTTCCTCTACCAATGGGCCTTCGC
CATCGCCGCCGCCGGCATCACCAGCGGAAGCATCGCCGAACGCACACAGTTCGTGGCC
TATCTCATCTACTCCTCCTTCCTCACCGGCTTCGTCTATCCGGTGGTCTCCCACTGGTTCT
GGTCCCCAGACGGCTGGGCCTCTGCCTTTAAGATCACCGACCGGCTATTTTCCACCGGC
GTAATAGACTTCGCCGGTTCCGGCGTAGTCCACATGGTCGGCGGAATAGCCGGCCTATG
GGGAGCGCTGATCGAAGGCCCAAGAATGGGACGTTTCGATCATGCAGGACGAGCTGTG
GCCTTGCGAGGCCACAGCGCGTCCTTAGTAGTCCTGGGAACCTTCTTGCTTTGGTTCGG
TTGGTACGGATTTAACCCCGGTTCATTTAACAAAATCCTACTTACTTACGGTAACTCAGGAA
ATTACTACGGTCAATGGAGCGCGGTTGGCAGAACCGCGGTCACCACTACCCTAGCGGGG
TCAACAGCTGCCTTGACCACGCTATTCGGTAAACGGGTGATATCCGGTCACTGGAACGTG
ACCGATGTCTGCAACGGGCTGTTAGGCGGTTTCGCGGCGATAACAGCCGGTTGCTCCGT
GGTTGAGCCATGGGCAGCCATCGTATGCGGTTTTGTTGCTTCTATAGTATTAATAGCTTGC
AACAAATTAGCAGAGAAGGTTAAGTTCGACGATCCTCTGGAGGCGGCGCAGTTGCACGG
TGGGTGTGGCACGTGGGGGGTGATATTCACGGCGTTGTTCGCAAAAAAGGAGTATGTGA
AGGAGGTTTACGGGTTGGGGAGGGCGCACGGGTTGCTCATGGGGGGTGGTGGGAAGT
TGCTGGCGGCGCACGTGATTCAGATTCTGGTGATTGCTGGGTGGGTTAGTGCGACCATG
GGACCCTTGTTTTGGGGGTTGAATAAACTGAAGCTGTTGAGGATTTCTTCAGAGGATGAG
CTTGCGGGGATGGACATGACTCGCCATGGAGGCTTTGCTTATGCTTATGAGGATGATGAG
ACGCACAAGCATGGGATGCAGTTGAGGAGGGTTGGGCCCAACGCGTCTTCCACACCCA
CCACTGATGAATGATCTTTTTTTCCCATATGCATGTCTCATTAGTCAAACATTAAATTTGGAT
ACATATTCCTTGTAAGGATTCAAACCTTGGTTACTTGTTACTTCTGTTAGATCCAACTCCGG
TTGATACTCATGACTTTTTACTTCTTTTTTTTTTTATTTGTCTTGGGTCTTCTTTTTTCGTAGA
TTTTTCTTTTTATGATGATGGGCAATTAGGGATTTTGATTTGTAATTGTCATTGGTCGTGCAT
TGGTGGATGCTGGAAGTTAAAGATTCTGGTGGAAGATGCGTACGTTTCTGTGGGGGGTG
GTTGTTGACTAAGGCATGTTGGTCCTGGAAATGACAGATGGCTGTGGAAAATGGAAATTT
GTGGGATTTATTTTTGTAGTTTTCACCAAAAAAGAAGGAAGAAGATTGGTATATAGTAGAAA
TACTACTGTTTGGCCGTGAGGCATATAGTTTTTTTTTCTTTTCCTTAATTTGAGACTTTTATG
TTAAACTTTTTCATTATGTCTAATGTAAATATATGGAAGTAGTTTTTATATTTTACTGCCTGAAT

GTTTGTTTTTTGTGTTATATGTTTTTGTTTATATGGAATTGAAATCGATTGTAATATGTTACGT
GGAAGTAATGTAAGTTAAAAGATGATGTAGGTAGT

>GmAMT1.5

GAGCCAGAAAAAAGGAGAGGAAAGATATCTAAATGCGTAGGGTTGAGGAGGAGCCAACG
AATCCCGAACTCAATCTGTAGGATTCCAACACAATATTTGAGGAGGATTTATTCGCGCATG
TGATTTATAATTGAAAGATTCCCTCTGCAGCTCTCAAAACCACTAACCCTAATAGCTGAACT
CCAAAGAGATAAAATAACAGCAAGTGGCCCTCATCAATTCTAAGTATATATTAAATCATTGC
CGCCATCACTTCTTCTCCATACTCTTGTCTCACTCTAACAGCCAAAGCATGGCTTCTCTCT
CTTGCTCCGCCAACGACCTTGCCCCACTCATCAACGACACCGCCGCCGCCAACTACCTC
TGCGCACAATTCGATTCCATTTCTAGAAAGCTCGTCGAAACAACCTACGCCGTCGACAAC
ACCTACCTTCTGTTTTCAGCGTATCTTGTCTTCGCCATGCAGCTCGGCTTCGCCATGCTCT
GCGCCGGCTCCGTCAGAGCCAAAAACACCATGAACATCATGCTCACCAACGTCCTCGAC
GCCGTCGCCGGCGGTCTCTCCTACTACCTATTCGGCTTTGCATTCGCCTTCGGCGGCCC
CTCCAACGGCTTCATCGGCCGCCCAAACTTCCGCCTACGAGATTACCCGATGGGCTCCT
CTATCTCCAGCTTCTTCCTCTACCAGTTGGTCTTAGCCATCGCCGACGCAGGAATCACCA
TCTCTCCCTTCGGCGACTACAGCTTCTTCCTCTACCAGTGGGCCTTCGCCATCGCCGCC
GCAGGAATCACCAGCGGCTCCATCGCCGAGAGAACACAGTTCGTGGCTTACCTTATCTAC
TCTTCTTTCTTAACCGGTTTCGTTTACCCCATCGTTTCGCATTGGTTCTGGTCCTCAGACG
GTTGGGCCAGCGCGACTCGTAGCCACGGAAATGTTTTATTCGGGTCTGGAGTCATCGAC
TTCGCGGGCTCAGGCGTTGTTCACATGGTTGGCGGGATAGCGGGCCTGTGGGGGGCTT
TAATTGAAGGCCCGAGAATCGGCCGGTTCGACCGTTCGGGCCGGTCGGTTGCTTTACGT
GGCCACAGCGCGTCTTTAGTTGTGCTTGGTACGTTTTTGTTATGGTTCGGCTGGTACGGC
TTCAACCCTGGTTCGTTTGTGACAATAGACAAGGGGTATGAAAGTGGAGGGTATTATGGT
CAATGGAGCGCTATAGGGAGGACAGCTGTCACGACGACATTGGCTGGGAGCACTGCGG
CTCTGACGACGTTGTTCAGCAAGCGGTTATTGGTTGGCCACTGGAACGTGATTGACGTG
TGTAACGGCCTGCTTGGCGGGTTCGCTGCCATTACATCGGGCTGTGCCGTTGTGGAACC
GTGGGCCGCGATTGTCTGCGGGTTTGTGGCGGCGTGGGTTTTGATTTGGCTTAATAAGC
TTGCCGCGAAGGTAGAGTACGATGATCCGTTGGAGGCGGCGCAGCTTCACGGCGGGTG
CGGAGCTTGGGGGGTTTTCTTCACGGGATTGTTTGCGAAGAAAGTGTACGTGGAGGAGA
TTTACGGTGTTGGAAGGCCGTTCGGGGCTTTGATGGGTGGCGGAGGGAGGCTGCTGGC
GGCGCAGGTGATTCAGATATTGGTGGTGTGCGGGTGGGTTACGGCGACCATGGCGCCG
TTGTTCTATGGGCTTCATAAGATGAAACTGTTGAGAATTTCGAGGGATGATGAGACTGCGG
GGATGGATTTGACGAGGCATGGTGGGTTTGCTTATGCATACCATGATGATGAAGATGGTTC
AAGCAGGGGAGTAGGATTCATGATGCTTAGAATTGAGCCTGCTGGTACCTCTCCCTCTCC
CCCCGCTGCACCACAAGTTTAATCAAAATGTGGTTTATGATTTTCAAGCGTTTTTTAGTTTC
GTACCTGCACATAGCTATGGGCAAAGCTAGCCTAGTCAAAACCATATACAAGCAAGACACG
AGGGATGCATATATGTAGTATAAAAATTAATGCGTGGGGGTCAACATTTAGGAAATGTCTTC
TAGAGTTACTGTACATTTTAAAATGTTTGTTGGCTTGGTTTATTATTTTCATCTTTGAATTCCA
AGACTAGTTTGGTCGACTGTTGTCACGTTAGTTTGTATCCTGCTGCAGAATAACTTGCTTG
TAATTGTATACTGATTAGTTGGTATATAGTGATATATTATATATACTAAATGCATTAATGCAGGT
TATGATTTTGCTAATCTTGTGATATTAGTGTTTCAACATACATTTTTTTCTTTTGATAAAATAAA
TATCATAGCTGAAGGTCAAGTACCATCCATCTCAGCTTGGTATCTTGAAATATGTTCTCAGA
GAATAAGATACTTCATCATAACAGAAGACCGACAGTGTAGGAGCATCAAAAGCAAATAAAT
ATAATTTAATATTTGAATAGTATTG

>GmAMT1.3
CTCCATACTCTTGTCTCACTCTAACAGCCAAAGCATGGCTTCTCTCTCTTGCTCCGCCAAC
GACCTTGCCCCACTCTTCAACGACACCGCCGCCGCCAACTACCTCTGCGCCCAATTCGA
TTCCATTTCTAGAAAGCTCGCCGAAACAACCTACGCCGTCGACAACACCTACCTTCTGTT
TTCAGCGTATCTTGTCTTCGCCATGCAGCTCGGCTTCGCCATGCTCTGCGCCGGCTCCG
TCAGAGCCAAAAACACCATGAACATCATGCTCACCAACGTCCTCGACGCCGCCGCCGGC
GGTCTCTCCTACTACCTATTCGGCTTTGCATTCGCCTTCGGCGGCCCCTCCAACGGCTTC
ATCGGCCGCCACTTCTTCGGCCTACGAGATTACCCGATGGGCTCCTCTCCCTCCGGCGA
CTACAGCTTCTTCCTCTACCAGTGGGCCTTCGCCATCGCCGCCGCAGGAATCACCAGCG
GCTCCATCGCCGAGAGAACACAGTTCGTGGCTTACCTTATCTACTCTTCTTTCTTAACCGG
TTTCGTTTACCCCATCGTTTCGCATTGGTTCTGGTCCTCGGACGGTTGGGCCAGCGCGA
GTCGTAGCGACGGAAATGTTTTATTCGGGTCTGGAGTCATCGACTTCGCGGGCTCAGGC
GTTGTTCACATGGTTGGCGGGATAGCGGGCCTGTGGGGGGCTTTAATTGAAGGCCCGAG
AATCGGCCGGTTCGACCGTTCGGGCCGGTCGGTTGCTTTACGTGGCCACAGCGCGTCT
TTAGTTGTGCTTGGTTCGTTTTTGTTATGGTTCGGCTGGTACGGCTTCAACCCTGGTTCGT
TTCTGACAATAGCCAAGGGGTATGGAAGTGGAGGGTATTATGGTCAATGGAGCGCTATAG
GGAGGACAGCTGTCACGACGACATTGGCTGGGAGCACTGCGGCTCTGACGACGTTGTT
CAGCAAGCGGTTATTGGCTGGCCACTGGAACGTGATTGACGTGTGTAACGGCCTGCTTG
GCGGGTTCGCTGCCATTACATCGGGCTGTGCCGTTGTGGAACCGTGGGCCGCGATTGT
GTGTGGGTTTGTGGCGGCGTGGGTTTTGATTGGGCTTAATAAGCTTGCCGCGAAGGTAG
AGTACGATGATCCGTTGGAGGCGGCGCAGCTTCACGGCGGGTGCGGCGCGTGGGGTG
TTTTCTTCACGGGATTGTTTGCGAAGAAAGTGTACGTGGAGGAGATTTACGGTGTTGGAA
GGCCGTTCGGGGCTTTGATGGGTGGCGGAGGGAGGCTGCTGGCGGCGCAGGTGATTC
AGATATTGGTGGTGTGCGGGTGGGTTACGGCGACCATGGCGCCGTTGTTCTATGGGCTT
CATAAGATGAAACTGTTGAGAATTTCGAGGGATGATGAGACTGCGGGGATGGATTTGACG
AGGCATGGTGGGTTTGCTTATGCATACCATGATGATGAAGATGGTTCAAGCAGGGGAGTA
GGGTTCATGCTGCGTAGAATTGAGCCTGCTGCTAGTACCACTCCCTCTCCCCCCGCTGC
ACCACAAGTTTAATCAAAATGTGGTTTATGATTTTCAAGCGTTTTTTAGTTTCGTACCTGCA
CATAGCTATGGGCAAAGCTAGCCTTGTCAAAACCATATACAAGCAAGACACGAGGGATGC
ATATATGAAGTATAAAAATTAATGCGTGGGGGTCAACATTTAGGAAATGTCTTCTAGAGTTA
CTGTACATTTTAAAATGTTTGTTGGCTTGGTTTATTATTTTCATCTTTGAATTCCAAGACTAG
TTTGGTCGACTGTTGTCACGTTAGTTTGTATCCTGCTGCAGAATAACTTGCTTGTAATTGTA
TACTGATTAGTTGGTATATAGTGATATATTATATATACTAAATGCATTAATGCAGGTTATGATTT
TG
>GmAMT1.4
CTCCATACTCTTGTCTCACTCTAACAGCCAAAGCATGGCTTCTCTCTCTTGCTCCGCCAAC
GACCTTGCCCCACTCTTCAACGACACCGCCGCCGCCAACTACCTCTGCGCCCAATTCGA
TTCCATTTCTAGAAAGCTCAACGTAACAACCTACGCCGTCGACAACACCTACCTTCTGTTT
TCAGCGTATCTTGTCTTCGCCATGCAGCTCGGCTTCGCCATGCTCTGCGCCGGCTCCGT
CAGAGCCAAAAACACCATGAACATCATGCTCACCAACGTCCTCGACGCCGCCGCCGGCG
GTCTCTCCTACTACCTATTCGGCTTTGCATTCGCCTTCGGCGGCCCCTCCAACGGCTTCA
TCGGCCGCCACTTCTTCGGCCTACGAAAATTCCCGAAGGACTCCCCTCCCTCCGGCGAC
TACAGCTTCTTCCTCTACCACTGGGCCTTCGCCATCGCCGCCGCAGGAATCACCAGCGG
CTCCATCGCCGAGAGAACACAGTTCGTGGCTTACCTTATCTACTCTTCTTTCTTAACCGGT

TTCGTTTACCCCATCGTTTCGCATTGGTTCTGGTCCTCAGACGGTTGGGCCAGCGCGACT
CGTAGCCACGGAAATGTTTTATTCGGGTCTGGAGTCATCGACTTCGCGGGCTCAGGCGT
TGTTCACATGGTTGGCGGGATAGCGGGCCTGTGGGGGGCTTTAATTGAAGGCCCGAGAA
TCGGCCGGTTCGACCGTTCGGGCCGGTCGGTTGCTTTACGTGGCCACAGCGCGTCTTTA
GTTGTGCTTGGTACGTTTTTGTTATGGTTCGGCTGGTACGGCTTCAACCCTGGTTCGTTT
CTGACAATAGCCAAGGGGTATGAAAGTGGAGGGTATTATGGTCAATGGAGCGCTATAGGG
AGGACAGCTGTCACGACGACATTGGCTGGGAGCACTGCGGCTCTGACGACGTTGTTCA
GCAAGCGGTTATTGGCTGGCCACTGGAACGTGATTGACGTGTGTAACGGCCTGCTTGGC
GGGTTCGCTGCCATTACATCGGGCTGTGCCGTTGTGGAACCGTGGGCCGCGATTGTGTG
TGGGTTTGTGGCGGCGTGGGTTTTGATTGGGCTTAATAAGCTTGCCGCGAAGGTAGAGT
ACGATGATCCGTTGGAGGCGGCGCAGCTTCACGGCGGGTGCGGAGCGTGGGGGGTTTT
CTTCACGGGATTGTTTGCGAAGAAAGAGTACGTGGAGGAGATTTACGGTGGTGGAAGGC
CGTTCGGGGCTTTGATGGGTGGCGGAGGGAGGCTGCTGGCGGCGCAGGTGATTCAGAT
ATTGGTGGTGTGCGGGTGGGTTACGGCGACCATGGCGCCGTTGTTCTATGGGCTTCATA
AGATGAAACTGTTGAGAATTTCGAGGGATGATGAGACTGCGGGGATGGATTTGACGAGG
CATGGTGGGTTTGCTTATGCATACCATGATGATGAAGATGGTTCAAGCAGGGGAGTAGGG
TTCATGCTGCGTAGAATTGAGCCTGCAGCTATTACCACTCCCTCTCCCCCCGCTGCACCA
CAAGTTTAATCAAAATGTGGTTTATGATTTTCAAGCGTTTTTTAGTTTCGTACCTGCACATA
GCTCTGGGCAAAGCTAGCCTAGTCAAAACCATATACAAGGGATGCATATATGAAGTATAAA
AATTAATGCGTGGGGGTCAACATTTAGGAAATGTCTTCTAGAGTTACTGTACATTTTAAAAT
GTTTGTTGGCTTGGTTTATTATTTTCATCTTTGAATTCCAAGACTAGTTTGGTCGACTGTTG
TCACGTTAGTTTGTATCCTGCTGCAGAATAACTTGCTTGTAATTGTATACTGATTAGTTGATA
TATAGTGATATATTATATGTACTAAATGCATTAATGCAGGTTATGATTTTG
>GmAMT4.6
AGGTTCCCTCCAAACAACATACTTCTGATGGCTTACCATGGATTCAACGGTGGAGATCCAT
ACGCAGCCAGCATAGATGCATCTCTGGCCGTCCTTAACACCCACGTCTGCACGGCAATG
AGCTTGTTGACTTGGCTTTTCCTTGACATACTCTTCTTTGGAAAACCTTCCGTCATTGGTG
CCACTCAAGGCATGATCACTGGCCTGGTTTGCAACACCCCTGCTGCCGGTACCATATGCC
TTCATAACCATATCTCACATATTTTATGAAACATTAATAGTTTCTATTAGTACACAAAGATTATG
TGATATGATTATGAGATAACTTTTTCTTTTAAATATTTGATAAGAAATTTGATTATAAAGATAAT
ACATACATAAAAAATATAATATTTGTCTAGAGAAATTAACTTCTTAAATAAATGAATTTTATTAG
CAGAAGAACTAAAACTTAAAATTTTTAAAATTGGGGACTAAAACAAAAGATGACCCATTTTC
ATATATCAAAACTATAATTTCAAACATTATATTATATATTCTTATGTAAGATTAATTGAATGGTTA
AGAAAGAACAGAACAAGAGGTTGAATCCATTCTACGTGACAAAAAATGACAGACTAATAAT
TAACCTTTATCCTTTATTTGATAAAAAAATTATTATATATGTTTTTGGTGTCATGAGTCATGAC
TAAAATGAATTTGTTAGGAAATCTGAAACTTGATTTATTTGTTTGTTTGAGTTTAGGTGTGGT
GCAAGGTTGGGCAGCAATCATAATGGGAATGATGTCTGGAAGCATCCCATGGTACACAAT
GATGGTCCTGCACAAAGAAAGTAAGCTACTCAAACAAGTAGATGATACTTCAGCAGTTTTC
CACACCCATGCCGTGGCTGGTAGCTTAGGTGGCATTTTGGCTGGTTTCTTTGCTGACCCT
AACCTTTGTTATCTCTTCTATGGAGTACAAGATTCCTTGCACTATACGGGTCTTGTTTATGG
CATATTCAATGGGAATCTCAAGACAGGGTTCAGGCAAATGGGGGTTCAGCTTCTTGGCAT
TGTTTTTGTAATACTTCTGAATGTCACCACAACTAGCATTGTGTGTTTGTTGGTTAGGATGA
TAGTTCCACTTAGGATGAGTGAAGATGACTTGCAAGTTGGGGATGAAGCAGTTCATGGCG
AGGTTGCATATGCATTGTGGGGTGATGGAGAGAAGCTTGAGACTGCTGCTGGTGAGGCT

TTAACTTCTGAAAAGAATCATGAATTGCCAACTGTGGAACTTATAAAAAGTTTGAGTGATGC
TCCATTAATCGATTGAATTGAAGGGTAAGAGATTCTAGCTCTTTTGTTGGTTTTGTTATTGT
TTGAGAGGGAAATACTAAAATAATAAGGATCAGTACTCATTAGTCTAGTAAGAAGAGAATTT
GGTTC
>GmAMT2.3
CTAAAGACAAAGACCGGAAGACATGCAAGTGTATACCAACCATCAAAGCAAAAGATATCAA
GACACAAAGATTCACAAACTCCAGTTTCATAAAATGATCATATATATACACTAGTGAGTCCA
AAGCTTGATTACAAAACTAAGATCTTCCAATTAAGAACTTGTGCTACCCATGGCCACTCCC
ACAGCATACCAAGAACACCTCCCTGCATCCCCCCACTGGCTAAACAAAGGGGACAACGC
ATGGCAGCTGACAGCAGCCACTCTCGTAGGTCTCCAAAGCATGCCGGGTCTGGTGATCC
TCTACGCCAGCATGGTGAAGAAGAAATGGGCCGTGAACTCTGCATTCATGGCCCTCTACG
CCTTTGCAGCAGTCCTAATATGCTGGGTGCTCGTTTGTCACCGAATGGCCTTCGGTGACA
AACTCCTTCCCTTCTGGGGGAAGGGCGCCCCAGCACTAGGCCAGAAGTTTTTAACACAC
CGCGCCAAAGTCCCCGAAAGCACGCACTATTATAACAATGGTACGGTCGAAAGCGCGAC
TTCGGAACCGTTGTTTGCCACGGCTTCTCTTGTGTATTTTCAATTCACGTTTGCGGCTATC
ACGCTTATCTTGTTGGCGGGATCGGTGCTAGGAAGAATGAACATCAAGGCTTGGATGGCT
TTTGTGCCTCTTTGGCTGATTTTTTCGTACACTGTTGGGGCGTTTAGTCTTTGGGGTGGT
GGCTTTCTCTACCATTGGGGGGTTATTGATTACTCTGGTGGATATGTTATTCATCTTTCCTC
TGGAATTGCTGGTTTCACTGCTGCTTACTGGGTACACAACCATCCTTCACTCTGTTCACAA
TCATTTATTAAACTTAATTGTTAAAAAAAAAAAAAAAAGAAGTTAGCAATGATGGTACAGAGAT
GTATTAAAATGCTGTTTTGTTGATTTGGTCAAGTGATATTTGGCATTATTGGTTGATTTGATT
TTGCTGCGCTTGACATTATTGAAAATATTAATTTGGTACTTTTTAATACCAAGTTACTTGAGG
AGAAACAATCTTACTTAAATTAAATAGTACTATTATGAGTTTTATACATTTCTTCATCTAAGTAT
TTAATCAACCGAATTTGAAATTGCATTTAACAAGTAAAATCAATTTAGCACTTCAAAATTTCA
TTTCATTAATTTTTGGCATCCCTAAAAAGTAAACTTGATTTCATGCTGAGAAAAAAAAAATTC
TTTTCATAACTACTACTAGTAGTTTTTTTTACTATTAAAAACTAAAATATTCGATAGTGTCATCT
TAAAGATATGAAAAGATGCTTAATTTATGTATATTTATGCATAAGACTGAGAATTTACTTGAAA
AATCATAAAACTAAGGATGGTTTTAGGTTGGGCCAAGGTTGAAGAGTGACAGGGAGAGGT
TCCCACCGAACAACGTATTGCTGATGCTTGCGGGTGCAGGGTTACTATGGATGGGGTGG
TCAGGGTTCAATGGTGGAGCACCATATGCAGCCAACATTGACTCTTCCATTGCTGTGCTC
AACACTAACATTTGTGCTGCCACTAGCCTCCTTGTATGGACTTCTCTTGATGTAATCTTCTT
CGGCAAGCCTTCGGTGATTGGTGCTGTTCAGGGCATGATGACTGGACTCGTTTGCATCA
CCCCAGGAGCAGGTATTTATATGTGATTTCTTAACATCTGTTTCATCCTTATGAGTTGTAAC
AAAAGTTCATTATATTCATATGATGACAGTAATAACAAGATGAATAAAGAAAATTAAAACCTA
GGGAATTTAAAAACAAGGAGTACTCTTTTCCAGCAAAAAAAAGAAGAGTATTAGTAATAATG
TTACAGTACTCCTAAAATATTGTTTGTTATTAGTTAAAATTTATTAAAAGTTATAAAAACATCAA
TACAACTTATTAAATAAAATAAGATGTGAGATTTACAAAAATTTATAATTTCTTATAAATTGATA
TTAAGGTAATAACAAAAAATGTGTTAAAAAAGTGTGTATCTAATATTCTTTTTTCTAATTATTTA
GCTTAAGAAAATATTTTTATTTTCATTGTTACCTTTACTTTAAAGTGTCACTTGTTTTGATTTT
ATACTTTGCTCTTTTTTTAAAAAAAACTTCAAAAATTGAAAAATGTTTTTATAATATTTTATTTT
TTATTTTCTGCTTGTGCTAATTTAATTGCTATAAAAATTGGAAAAAATATAAATATAAATATAAA
AAACATTTTCTTAAACTAAACAAGCCATAAATAATATTATCTAAGTTTTAAAAGTGAATATAGA
TAAAATTTGATCTTTATTTTATGCATATAAAAAAACTGATCATTTTTGGCATATGTCAGAATTT
TATTTGTAACTAAAGTATATTTTTAGTTTACTTGAATGTAATTCCGTTTTATTGTAATCATTTAA

AAATTACTAGTTTTTTATTTCGTAAATAATTTCTATTTAATAATTATGTGACTTACCAAAGCATA
TCATGTCGTGACATTTTTTTTCAACTAATTTTGATATGATATTAATAAATATATGTCATGACATA
AATATGAACAAAGTACGAAAGTCTAAACATTGACATAATCAATATAAGATAAAAGATTTTGAA
TGATTAAAAAAATTAATGATAGATGGACTAAAATATTTGAAGCAGGTATATAATTACCATCACT
TCTGATGTTTAGTTGTACCGTAGTTAATTGCCAGTTGTTGACAATTAATACAAAAGGGTGAT
GCGGATGCAAATTGCAGGGCTTGTGCAATCTTGGGCGGCTATAGTGATGGGAATTCTATC
AGGGAGCATTCCATGGGTGACCATGATGATCCTCCACAAGAAGTCAAGCCTTCTACAGAA
GGTATGGTTTTGTTTTCGTCAAAGCACCGTACTTTTCAACTTCCACTGTACACTTAACAAG
ACTCTTAATTTTTTTTCCATTTAGGAGAATACAAAACATTCAATTCATTTTAGGTGGATTGGA
GTGGAAAGAAAAGATACGAAGAAAAAAGAAAAAAATAAAGAAAATAGTAAATCTGATAAATA
ATATAATGAAAACAAAAGAGAGATAAGAAGAAAATAAAATGAAAAGGGAAAAAAAGTGTTTA
AACACTCAAAAGAAATTGACCCGTCAAGAGAGATAAAAAAAAAAAAAAAAAGATGATGATA
GATAATAAAATATGATAAAAAGAGAGAGATAGAGAGAAAGTAGGAGTGTTAATAAGGTGTTG
AAATATATATAAAAACTCAAGTGTAAAGTTAAGTTTCAACCCTTAATTAATATTTTATTTGATAA
TATAAAATATATTTGAATTTAATTTCTAGTGTTTACTCTTGTTATTTTCATAGTTGATATGTACA
GACAATTTATGATCTATCAAATTACAATATAAAATCTGTTTATATGATATATATACATTATTTAAT
TACTCATTTATTTAAAACGACCTTAAAAAGTGTTTTGTAAAATTGGAAAAATTTATGAGTGGG
TTAATTTTAAAAATAAAGATTACATGAATGGATAGATTTTGAAAATATTACGTGCTAGAGATAA
GTTGCGTTATAAAATGTGATTTCTTTATAATAAATCATGTTTTAAAACATAACTTTAGAACATC
ACAATTTATATATATATATATATATATAGAGAGTAGTGTTTTATTATGAGAGGTGAGAGGATTAA
ATAATAATCTTTAGATCAAAATAGAAGGTTTAGAATTAATTGTTAATTAAACAAGATGAGAGA
AAAATTATATGTGCACTTAATATTGACTCTCTCACGTGTAATATTTTTTTTTCGCTCATCTCAT
TTAGTTAGCAATTTGATCTAAGAACTATTATTTAATTCTCTCACCTCTCATAATAAAACACTCC
TCTCATCTGGTATGTCTCTCATATATATATATATATATATATATATATATATATATATATATATA
TATATATATATATATATATATAATATGCTTGAAGCCACTTTAGTATTATATTTTAAAAAAAATCAAA
TAATTACTTTTGTCTTACATGTGTAATCCCTAACAAATGCGTTCTTGAAAGATAAAAATAAAA
AATTTATTTTCCAAAAGTGTAAAAAGTACAGCAAATATATTTGACCGTTAACTCTCGTCTGTC
ACCAATAATAAAATAGCATATGTGACATAAAGGGACAAATTTGTCGTCACCAACTTGGCCAT
ATCGAATTGCCAACATGGGGATATATTTGTTATATAATATTTTGTTGATTTTTCCACTCTGCTA
ACAAATGCATTCCTAAAAGATAAAAATATAAAATTTAGTCTCCGAAAGTGTAAAAAGTGTGAT
AAATATATTCAGGCGTTAATAGTAGATTGTGACTAATTATTATAATTTGGAAAAATTTATACTAA
TTACAACAAATTTTTATGACAAACTCATAAATTAAATTGAGTCTTAAAGAAAAAAGAATATTAT
TTTTATAAACTCGTAAATATCTTTTTTATACTCTCATGTTAGATGAAAGGTTTAAGTAAAAAAA
AATACTTATTGTAAAATAATATAGTTAAATTAGATCCACGATTAATTTTTAGGAAAAATTACAAT
TGCAATAACATTTTTAGTTTGTAAAAAACACTCATCTCCTTGGAATGATTTTTTTTAAGATTAT
GATTTTTTAAATGACCAATCAATAAAAAATAATTGTGTATGATATAAAAAATTAATTTAAAAATC
AACAAACTTTTATTATAATTTGTAATTTTGATGTCACTACATATACTAATAAATATTCATATTTTA
TTATGAAAAATTTATAAAAAAAAATTAAATAAATAGTGTTTGATTAAACATAAACAATGATTTTCA
TATCGTTTTACAGTAACATTTCTTTTTTTTCCTTGTGTTGATAGCATTTATTCAAGAGTTAACA
ACCAAAAGATTGATTTTTTTTTTTAGTTGAAAAAAAGTAAGAAGAATTCACCAAAACAATAAA
AATTCACTTTTATTGATAATATTTTGTTGCAATTATTATAAAATTTTCAAAATTATAATAATCAGT
CACATTCTACTATTAACGCTCAAATATATTTGTCTTGTATACTTTCGGAGATTAAATTTTGTAC
TTTCATATTTCAGGGACATATTTGTCAGTGAAATGGAAGACTAAAAGTAAAAAAAAAATTATA
TGACAAATATGTCCCTATGCTCACAATTAGAAATAGTCATATTTACCATCATTTCAGTGACAA

ATTCGTCTCTCTGTATCACGTAAGTTATTTTATTAACGGTGATGGATGCAAGTTAACGACCG
GATATATTTGTCGCATTTTTTACACCTTCGGAGACTAAATTTTGTATTTTTATTTTTTAGGGAT
ATATTTGTCAGCGAATTACACATTCAATGATAAAAATGACTATTTATCCTAAAGAAATTACTAC
ATTCTAAAATCATATTTTAAGACACAACTTATCCATAGCCTATAATATTTTCAAAATCTATCCAT
TCTTGTAATTTAAATAAATAAATAACCCACTCCTATAAATTAGCTGCAAAATTGTGATATTCTT
GTATCAGTTTAGGTTTGGGTCGTTTTTGCCAAGCTTGTTTCATTAACTCTCTTTCGATGATA
TATTCCTGTATAAGTTTAGGTCTGGGTTGCTTTTGCTTCGCTGGTTTCATTAATTCTCATCA
ATAATATTAGAAAGTTAGCTAAGAAATGGACAAATTCACATGTAATCTGTTGATTTACACACT
TTCCCCCCGCGAAAAGGTAAGAGCTCGGTTGGATTTGATATAAAAGATAAACATAGAAGAT
CGATGAATATATTAATTCTTTATCTTCAATAAAACATCTTCAAATAGGTATTATTTTAACGCATG
TTTGATTTCCAGTTGAAATTATTATGATACTCACTTCAATGCAGATTAAATGAATAAAAAAAAT
AAAAATAGAAATAAAAGTCCTGATTTTTTTGCAGAATGATTTTTGCCTCAAAAGTCAGATTTT
CAAACATGTCCTTAATTATTTAATAATAATTTCCTTCCGTTTTCCATATTGGTCAGATGGCATT
TTAGTCTCCAACGATATTATTAATCCAATGCAAGTTACATAATTAATCCTACGATATCGCTGT
CATATCGTGAAATGAATTCTCAACCACACTTCTTAATCATATCTATTGCATCTCCATCAAACA
AATTACATGAGTTGCTACATATAAAATTTTATAATTTTTTTACACTTCTCAAATATACATGACA
GTTAACTCACTCATCTGTATTTTCTTATTTATTTATTTTAATTTCAATTTAATATATTTATATGTTA
TTTGTATAAATAATTAATACATATATAATGATTTTCAATTACCTATATTTCCACTTCAAAGAAAA
AGCCTGAACCTTTTTAAATAAATATACAATAATTTTCATTAAATATTTTATAATACATGAAGAAA
TACTATGTAATAATAATTTAAATAAATAAAACAAATTTTACATCAATATTTTTGAAAATATACGAA
CACTTACAAAGTAACCTATTATTAAAATACAACCACTAAGTGCATGCATCAAGTCAAATAGA
CATATAATGGGAGGCCAAGATGATGGGTTCAACAATAATTACTAGGATAAAATCAAATACAT
ATTATAATTTTGGGATCATACCTACTCAACTTCACAAAAATAGGATAATGCCCCATTTGAATA
TTCTGTTTTTACCGTATCACTAATAGTTACATTGAATTTATACTATGTTCTTACCATTTTGTGA
ATATACAATTCACAGGTAGATGATACCTTAGGAGTGTTCCACACACACGCTGTGGCTGGCC
TATTGGGTGGCCTCCTCACAGGTCTCTTAGCAGAACCTCAGCTTTGTAGGCTCATATTGC
CAGTGACCAACTCAAGGGGTGCCTTCTATGGTGGCAGTGGTGGCATTCAACTTTTTAAGC
AATTAGTGGCAGCATGTTTTATAGCTGGATGGAACTTGGTCTCGACCACACTGATTCTTCT
GACCATACAGTTGTTCATACCCTTGAGGATGCCAACCGAGCAGCTCGAGATCGGCGACG
ATGCCATCCACGGCGAAGAAGCATATGCTCTTTGGGGTGATGGTGAAAAATATGACCCAA
CCAGGCATGGATCCTCAAGAGTGGAAAAAACTTCAGCTTCGCCCCGTGTTAATGTAACTG
GAGCAAGGGGTGTGACCATAGATTTATGATTCATGTAGATATAGCTCTCTATAGTAGGAGTG
TTATGGGCAAAATATGAATGAATGCTCGTTCCTGTGTACGTTTGCTATTATAATAGGCAATG
TAAATTGTACTGGCAATCAATTCACTCATGTCCAAAGCTGGGAGATGTAAGTGGATTGGAT
GGTCAAAGAAGGAAAGAGGGGGAAAAAAATCACGGATCCGATCTTCTTTGCTAACCAAAA
ATTAACATTT
>GmAMT2.1
AGTAAAATTGCCACAAACAATTCATCAGCTCATACACGTAATTTCTTTTCCTCTTTTCCTCTT
ATCCAATTCTAATCACGATCAGACATTAAATGTAAACACTTCTCTATCAAAAATTTGAACTTA
GTTCGCCTCACACTTTTGTTTTGTCACCTTGTGAGAGACTAATTCCCTCTAATAAACGCAA
CGTTGTTCATCAGTGGCACATACATATACAGCATCACAATTCTTTGAAGGGTGAAAAAGCT
TGATCAAGAATTGAAGCATATTGATCTTCAGCCATGGCTACACCCTTGGCCTACCAAGAGC
ACCTTCCGGCGGCACCCGAATGGCTGAACAAAGGTGACAACGCATGGCAGCTAACAGCA
GCCACCCTCGTCGGTCTTCAAAGCATGCCGGGTCTCGTGATCCTCTACGCCAGCATAGT

GAAGAAAAAATGGGCAGTGAACTCAGCTTTCATGGCTCTCTACGCCTTTGCGGCGGTTCT
AATATGTTGGGTGCTTGTGTGTTACCGAATGGCCTTTGGAGAAGAACTTTTCCCCTTCTG
GGGAAAGGGTGCTCCAGCACTAGGCCAGAAGTTCCTCACGAAAAGAGCCATAGTCATTG
AAACCATCCACCACTTTGATAATGGCACTGTTGAATCACCTCCTGAGGAACCCTTTTACCC
TATGGCCTCGCTTGTGTATTTCCAATTCACTTTTGCTGCTATTACTCTTATTTTGTTGGCTG
GCTCTGTCCTTGGCCGAATGAACATCAAGGCTTGGATGGCTTTTGTGCCTCTTTGGTTGA
TCTTTTCCTACACAGTCGGGGCTTTTAGTCTTTGGGGTGGTGGCTTTCTCTACCAATGGG
GCGTTATTGATTATTCTGGCGGCTATGTCATACACCTTTCTTCTGGAATCGCTGGCTTCAC
TGCTGCTTACTGGGTACGTATATTTTACTTTCATTTTCTTCATAAACACAAACTCTAGTCAAA
TTTGCTTCAAAAGTCTAAAGGCTAGCTCCCTAGCCAAAGTTTTTAATATTGTGATAAATTATG
GAAAGAAGATGCGGCTGTGGTTTTGTTACAATCTTTCATATTGTGAGAAATTGCAAAACAA
AGTCGTCCATGTATCCGCAATTGGGATCCCATGTGGGGTTATGGAGAGGCCAAAAACTTT
GATGTTAAGGCCATATTTAAACTGTCTTTGGTTGTTGGTTTTTGGTTTTCAGCACAAATACT
AAATTCAGTTATTTTCATTTTAGAAGAAATAACCTTTTTTTTCTTCTTTTTATCAATTTTGATAA
TAGTTTTTTTTTATATAAAATTAATGTTATTTTCCTAAAGTAACAATTTCCAAACATGTTAATGT
TGGTATAAAAATCAACCTATTAATATTTGATCATTAATTTTGGGCACCAAAGGAAACCCACAT
GCTGATTATTCATTTATTGAATGCCAATTTGGTTTTTGATTGTGTGTGCATTAAATATGTTGT
GCAGGTTGGACCAAGGTTGAAGAGTGATAGGGAGAGGTTCCCACCAAACAATGTGCTTC
TCATGCTTGCTGGTGCTGGGTTGTTGTGGATGGGGTGGTCAGGGTTCAACGGTGGAGCA
CCATATGCTGCAAACATTGCATCTTCAATTGCGGTGTTGAACACAAACATTTGTGCAGCCA
CTAGCCTCCTTGTGTGGACAACTTTGGATGTCATTTTTTTTGGGAAACCTTCGGTGATTGG
AGCTGTGCAGGGCATGATGACTGGACTTGTATGCATCACCCCAGGGGCAGGTATATATAG
AGAGAGAATTGTCGTTCTTAATCAATGATCACATTTTATATGACTATGCAATGAGATAAAATA
CAAGTTACTCTTAGCATTAATGCTTAATTTGAAAAGAAAAATATTTTTTTTTTTGGATTAAATA
ATACTTATTTTGTTTTTGACTTTGCAACCCGGGTTATATATTGAATAAATTTTCCTTGGATTAA
GTAGTATTGTGAAATGTTTTAGAATTAGTATACCAGAGTTTAAATTCGTTGGCAAGATCACAT
GATATAGCGTGTAGTCCCAGAAAAATAATACTACTAAAAACTACGAGGGAATAAAATAGAAG
CTACACCAGGTAAGACAATTAAAAAATTAAAATTCGGCATAAAACTAAAAATCCTATCCTTC
CTTTCCACAGAGAGGCCAAATTTTAACATGTGCATCAGTCACCTGATTACAATTATGTATAT
AAACTAAACTCTTAATTTTATCCAAAAAAGTATTATAAATAATGAAAATGACTAAATCTATTCAT
TGATGATCGAGCATGTGTGGATGCAGTTTTTGTTGACAATTAATTAATGCAAGGGGTGTGA
CTATAACGTGCAGGGCTTGTGCAATCATGGGCTGCTATAGTGATGGGAATATTATCTGGGA
GCATTCCATGGGTGACTATGATGATTTTGCATAAAAAGTCAACTTTGCTACAGAAGGTCAG
GGTTTCTTTACTCAAGTCACACAAATTTTTCCACCCAAAATAACTTATAGTACTCATTTTATC
TTTCTCTTTCATTTCACTTTCATTTTATCTTTTTTTTTCATCTCTTTTTTTTTCATTTTTATCATA
TCATTTGTTAAATCTATCCTACGTTTATCATTTAATGTAAGTATAGAATTTAGGTGTAGCTTCA
CTTTTTCTGTGCACGTTTTTGTGAATTTTCATTTAATATTAGGGAAAAAAAGACTAAAACTCC
AGGTCATCCGTTGATTCCAACAACTTTCATTTCTTTTTGCCCACTTACGTGTTATTTATTAAT
TTCTTATATATATAAAAAAAAAAAGATGATGCAAGATGAAAGTCATCCTCTTTTTCTTGGAAA
GTAGATGGGAGTGCCTGATGTCACCTAATTTTCTTAAAAAATTTAATTCATCATTTTTAAAAA
AAAAATATTTTTTAATTAAATTAATATTTAAAGAGCAGAAGTAATTGACCTAATAAAATGACGA
TAAACACTACCCATTTTATCTTGTTCTTCGATTTTTCTCTTCATTGCTTAATAGATTCCTTGAA
TCCTTCTGTTTACCATAATGGCGTAATGACATTTTCGTCTAAGCATTATCTGCGCTTGCCTA
ATTACTCCTCAAAGATCACTATCATACCACCAAACTTCAACTACACATTAATAATAACATTTT

GAATCTTATTTTTCGTTCCAAAAAACACATTTCGTGGATCAATCATGCATATTTAGCTAGTTC
GGTCACCTTCCATTTGTCTATTTATCCACCTTTAGTTATATAAATTTGATCGTATTTCATTAAC
CGACTTGAATTTTTAGATGAAAATTAATTTGAAATGTGCATAACTGATCACATACGGAATGTA
ACAAGAATTAATACTTATTGGTGTAAAAAAAATTATACTATCAAATAATAACAAGATATCATCG
TATATAATTTTCTTGTCGACTTTTATGATAATTAAAAGTCTAACCTGACTTAATGATTAGTTGA
TTATATAAAATTATTAATATATATATATATATTTAAATTCTTTTTTTAGCAAAAAATATGTACATTTA
GAATTTTTTTATCAGCTATACTTAGACTTTAAAGAAATTTTTATATTATCACTTTATTTAATTTT
GTGATGTGTAGTAAATTTATTGATTTTTATAATAATTAAAAATCATATTAGTCATATTTTTTTATT
AGTTGACCGTTAGACTGAAATGTGTATATGTATTAAACTCTTGATTTTTTACTTAGTATAATTT
GTACTTACCATAATTTTGTGAAAAATATTATGTTCAGGTAGATGACACCCTTGGTGTGTTTC
ACACACATGCTGTGGCTGGCCTTTTGGGTGGTCTCCTCACAGGTCTATTAGCAGAACCAG
CCCTTTGTAGACTTCTATTGCCAGTAACAAATTCAAGGGGTGCATTCTATGGTGGAGGTGG
TGGTGTGCAGTTCTTCAAGCAATTGGTGGCGGCCATGTTTGTTATTGGATGGAACTTGGT
GTCCACCACCATTATTCTCCTTGTCATAAAATTGTTCATACCCTTGAGGATGCCGGACGAG
CAGCTGGAAATCGGTGACGACGCCGTCCACGGTGAGGAAGCTTATGCCCTTTGGGGTG
ATGGAGAAAAATATGACCCAACTAGGCATGGTTCCTTGCAAAGTGGCAACACTACTGTCT
CACCTTATGTTAATGGTGCAAGAGGGGTGACTATAAACTTATGAGTCAAGAAATTAGGCTG
TGCCTTGCTCACACATGCATGTGTATAAATTTATATGATTAACAAATGTGATGAATCCGTGA
GTGGTATAAGTAGATATTTGATTTTGTCATGAAAGAAAATTTCCAAATTTTGAGATCTGATGT
TCCTCTGGTCATCTTGCATTCGAAGACCTGGTCATATATTTCTGGCACAGAATGTCTTGGC
ATGTGTATAAAATTTAGATTTGTCAAATTTTAAAGGAACTTATGATTAGTTTTTTTCACTTAGA
AGAAGATCAAATTTTCTTTTTATGAAATGCTTAATACGTATTTGTTAAAAAATTTGTCCAAATG
TTATAAGTACGAAATATTTATTAGTTTTATTAACGAGTAATTTATACTAAAATGTGTTTGGATAG
AGAATTTCAATTGAGAAAAGTAATTTATTAGAGAATTTAAATTTTTTTAATTTAAAATTCATTGT
TTGTATGTTTTTTGTGAAAAATTTAAATTTTTGGAATTATAAAACAGAATTTTAAATAACCAAA
AATGTGAAATTTTATTTTTTTTCTAAAAGGTGAGAAATTGAAATTCTTTTCTTGATAGAAGAA
CTTTCCAAAACGTTTGTGTGTTTTTTTTATCACCTTCATCCTCTTTCCCATCTGAAAGTTCT
CAAAATCATGTTCTCAAACTCGCGATTCTTCCCTCATGTCGATGATACTCTTATTCGAGAAG
TACCGCCCAAGCTCGCGGAGCGTCGGGTGCAGGCGTAGGGGGACATGTAGAATTGCAC
CCGTCAAACAAGGGAGTAGTCGTCGCCGTCCATCATGCGACGGAGGTGCTCGCCGGCA
CAGTTGTGGTCGGATGTGGTGGTGTACATTACCCTATCCTAGTTCCCTGCGAACTCCACA
TATCGCATAGTATTATTCTCTTTAGAAGTAAACTAATCATATCTTCTTTTTTTCTTTGCATTCATT
TTCTTTCATCCTCACAATTTTAGTTTTTTTTTATTCAAACATAATTTTAAAAATAAAAGAATTTCA
ATTGAAGTATTTGAAATTCTTAGAATTTAAAATTTCTCATAATTTTAAGTTTTCTTATCCAAAC
ACACTCTTAGGAGATTTATGTTTAAAAGAATGATTTCAACTCCAAAATATTCACCTAGTTCTT
CTCAAACAGATTTTATTTTGACTCATGTTGTTTTTACTGCATTATTTTGGCTCACTTAAAGGG
TAAAAGTTTGTACATTATTTTTAGTTATGCATTTCTGAACGAAGTTTGGTTCGAAATACTTTC
AGAGCAGGTCAAATATAAATTCTTCAAAAGAAAAAATCTAGATTATTTGACAGAAAAATAGAT
AAGTTTCTCATAAACTGAGCACAAGTTTTCCAAATTGATGGTACGACAGATGTACTTCTGT
CCAAAGCTATTGACTCTGGAATGGAGCGACTTCGAGTGTAAATTATAATGTGGAATGGAGC
GACTTCGAGTATAAATTATAATGTGGAATGGAGAGATTTCAATGTTTGCGGCCAAAGTATGC
TAAATTGGTGTTTTAATATAAAAAAAAATGTTTTTATTTTTTGACGATAAAATTGGTCTTTTTC
ACAATTTATACGTATAGAGGAACCTTCTATGAAAAATTTAAGTAATCCATGTGGAAAAGTGA
CAATTGGTAGGTTAGAAGAATCATAGGATAGGATGAAAATTAAGAAGTTGGAAAAATCCATC

ACGTTAAGTGGTTTTGTTTTATACGGAAATTGAATGAGGATGCAATTTCATTTGTTCGCTCG
TGAGACAACACCCGTCCTCACTTACCTTTTGAGTTGGAATGTGGCCATTTGTCGTTTCACT
TATTGATAATTAGTAGCTCTTTTATTTATTTATTAGTCATGTTTATTTTGAAAAGTCTTCCATTA
ATTGGAGCAGTCTCTTTGGACCTGGATGTTAGAGTTTTTCTTTATGATTATCATGGTAGAAG
CAAGAAAGCAATCATATCTGATATATTTCTTATGAGATTACTTACCTTTTAATTACATCATCTAT
CATAAATGTATGTAGGATCATATAGTGCAAAAGTTTGCAGTGTCTTCATCTTGAGTATCTTGT
TCTTGGATTAATTTGACAAGAAAATATTAAAATAAACTCTAAGTCTTTTACCATATATCCAGC
CTTAAATTCAAGAGTGAATGAAATGGACTAAGTTCTGACTTTTATCAAACAATGGTTGTAGT
GTACTTGCTATCAACAATCTTTTCAGTGCATATCAGAACTGACAATGGATACACTCTCCAAA
TTTCATGGAAGGATTTATGTAAAGGAATAACCAGATTATGCTCCTCCATTTTCTTCTTCATTC
CAAATTCTAACTTCAAAGTATGCCCCCTAAGGCAGTCAAATGGTTTACAGATATTTTGTCTT
CAGCAAACTACAAAAAATTCAACCTTGATTGAGGAATCAAGGTCTAAAAATATGGTGTTCTA
TATGCTTTGTCGTTTTATGTTACATAGAAATTTCTTAGCTAATCAAGCCGCTCCCAATCTTGA
CTTACATTTCTAGAAGATGACATTGAGGTAGGGGCATTCAAGCTTCCCATTCCAGAGTCAA
AGGTCTCCCATTGGGTTGCTACAGAGACAGCTTTACCAATGGGGGTAAAAGGATTGGAGT
TCTTTGCCTTCAGAGAATCTAGTGTATCTACAAAGTTCTGCACTCGACGCACCTGTTGACG
GGTGAAAAAGCTGATGTTCAGGCCTTCAAAGATAATTTGAAAAGATAATACAACTATGAATG
ATTATCAAAATGGTATGTTCATCACTCACCACCATGCACAAAATT
>GmAMT4.1
AAGAATAACGATGAGTGCATACACATGTAAATGATTTGACAGGTTGGGCCACGGCTAAACA
AGGACAGGGAAAGGTTCCCTCCAAACAACATTCTTCTGATGCTGGCAGGGGCAGGGCTG
CTGTGGATGGGGTGGACGGGCTTCAACGGAGGAGATCCATACACAGTAAACTCGGATGC
TTCCCTTGCCGTCCTCAACACCCATGCTTGCACTGCTACCAGCTTGCTCACTTGGGTCAT
CCTTGATGTCCTCTTTTTCCGAAAGCCTTCTGTCATTGGAGCTGTTCAAGGCATGATCACT
GGCTTGGTTTGCATAACCCCTGCTGCAGGTTTCTAAACTTCAATCTCCATATTTATTTATATA
ATTTTTTTGAGTAAATTATACCGATATTCTTTGAAATTAATTTGTCAAATTTTCTCAATATCCCT
TCTTTTTCAACTCTTACAATAATCTCTTAATTGTTTGAAAAATATTACACTAATATCTTATATAT
GTTAAGGTTAATTTATTAGTTTTTATTAATGAGAGGATTTAAACCTACCATCTCTCTCCTCTTT
CTTCCTAAACCACTACTATATTAATCTTATATTTATTATTAAATGATCTCCTTGAAAACATTGAA
CCATGTAGTCTTATAAGAGAGATTGTTATAAACGTTTTAAAAAACCAGTAGGATGGCATGCA
ACCTCAAGAAATCGCAAAGGTGTCAGTGTAACTTACTTTCTTTTTTTTGGGTGTACTATAGT
GAGTAGGAACTAATTAGAATGTTTCATGGTATAATTGTTCCTTCCTTGTCTTGTTCAGGTGT
TGTGGAGGGATGGGCAGCACTTATAATGGGAGTGTTATCTGGCTCAATTCCATGGTTCAC
CATGATGGTGGTCCATAAGAGATCAAAGCTACTGCAGAAGGTTGATGATACAATGGCAGT
GTTCCACACTCATGCCATTGCAGGAACCCTAGGAGGACTCCTCACTGGACTCTTTGCTGA
TCCAAGACTTAACAAATTGTTCTATGGAACTGATGGTCAATACGTTGGTTTCTTTTATGGTT
TAGGTTCGAAGAAGGTCAGTACCGGGTTCAGGCAAATGGGGGTCCAAATCCTTGGGATT
ATCTTTGTTATCTTTGTCAATGTTATCAGCACAAGTTTGATTTGCCTCTTCATTAGAATTTTT
GTTCCGTTGAGAATGTCTGAAGAGGATATGGAGATAGGTGATGAGGCTGCTCATGGGGAA
GAAGCTTATGCAATCTGGGGTCAGGGTGATAAGCTTGAAAACTCAAGTTCTAAGTATGGAA
GTTCATTATATGATGATGTTGAAGCTGGTGGCCCCAAGAAGAAGCGTGGCGGCACGGTT
GAGATGATGTCGAACTAAATATATAGTATGATATGTCGTTGTATTGGTCTTGGTCTTGTTTTC
AGAAACATATATTAATGTGAAATAAAAGGTCGTGAATGTCTCATTGTTCTTTGGGTCCTAGG
ATACATCTACTTTGTTTATTTTACTTTCCCTATGTTCATTCTTCAGAACAAATGAATAAGATCA

CTAACGGAAATTTCGATT

>GmAMT3.1

TAATTAAGGAAAATAAAGAAAGAATATAAATGTGCAAGAATCCTGGGATGTCAACGTGGAG
GATCACCTTTTACTTAGACAAGTAGATGGAATCAGTAGGGAGATGCATCTTCTCTTATATCT
TAAAAAATACGTAATTATTATTAGGAGCCAGGCGTACACTTGAAGTATGAATAAAAAGACAC
ACCAAAGATACCATTGTTAAAAGTTTGGGCAGCCCTAGGCACAAAAGAAAGTGGTGATTG
ATGGCTACTCCTCCGGTGAATTCGTTGCCAATAGCATACCAAGCATGGACAAGCCTGGGA
GTCCCAGATTGGTTGAACAAAGGAGACAACGCATGGCAAATGGTGTCAGCCACCTTGGT
TGGGATTCAAAGCATGCCGGGCCTCGTCATCCTCTACGGAAGCATCGTCAAAAAGAAATG
GGCCGTCAACTCTGCCTTCATGGCCCTCTACGCTTTTGCGGCGGTTATCATCTGTTGGGT
GGCGTGGGCCTACAAGATGTCGTTTGGGGAGGAGCTGCTGCCGTTTTGGGGCAAAGCC
GGGCCCGCGTTGGGCCAGAGGTTTCTGATAAAGCAGGCGGGCCTGCCCGCCACGCCG
CATTATTTTAGAAACGGTGGTGGTTTGGAGACGGCGGAGATAACGCCGTTTTATCCGATG
GGTACTATGGTATGGTTTCAGTGTGTTTTTGCGGCTATTGCGGTTGTTATATTGGCTGGGT
CGGTGCTGGCTAGGATGAACTTCAAGGCGTGGATGATGTTTGTGCCGCTTTGGCTTACTT
TCTCTTACACCATTGGGGCGTTTAGCTTGTGGGGTGGAGGGTTCTTGTTCCACTGGGGC
GTTATGGACTACTCTGGTGGTTATGTTATTCATCTCTCTTCCGGGATTGCTGGCTTCACTG
CTGCTTATTGGGTTTGTTACTTCACTTCTTACACTTCCACCCTAAGGTCATCATTTTTAATTT
TCTATATGGCCGCAACCATGCAACCTAATTTCGTTTCTTGATGAGAGTCTAGGATTAAAAAG
ACAAAGTAAATTATTCAACATACATACTAAAAATGAACAAGGATGTGTATACACGATATGCAT
GCACGCTTTCACTGTTTAGTATTTTCTGAAATGCATCAAAATTATATTAACTTTAATTTAATTA
ATGTGTGTTTTTTGCTTTGCCTTGTTCCACATGACCTTATTTCCTTCTATAATTAAAAATTAA
AAGATAAAAGTCTAGTTAAGAAACATAGTTTAAATGAAAACCGGAACCGTTTGATAGTGTTT
GTATTTTATATATCATTAATCTCTCACTATATATCTAATAATTTTTGTTAACATAATGACGCATCA
ACAATCACATATATATTTTAATAGGGTTAGCTAAGCTTACACGTTAAATTAAAATGTAAAGCTT
AATTTTCTTGGTGATGCAACCACAATATTTAAATAAAAGCGGGAAATTAATCAAACAGAATTA
ATAGAGTAATTATCATCATCGATCGTTATCATGGAATTTGAAAGCAACGAAATTAATCTGAAG
ACAAAGAACAAGGTGGACCGATGGAGAATGGTGAAATGATAAAATTTTGAAAATGAAACA
GGTGGGGCCAAGATCGAAGAAGGACAGAGAGAGATTCCCGCCAAACAATGTGTTGCTAA
CGTTGGCGGGGGCAGGGCTACTATGGATGGGATGGGCAGGTTTCAACGGCGGAGATCC
GTACGCGGCAAACACGGACTCGTCGATGGCGGTGCTTAACACCAACATTTGCGCCGCCA
CCAGCCTCCTCGTATGGACGTGGTTGGACGTTATTTTCTTCAAGAAACCCTCAGTTATTGG
AGCCGTTCAGGGCATGATAACTGGCCTTGTTTGCATCACTCCCGGAGCTGGTAAGTCCCT
AATTATAACAAATCCTATCAAGTGGTTTTTATTAGGATATGTAAAATTATATTGTTTATATTTTTT
TTATGTGTTTTTATAATTTTTACATTAATTTTTTAATTAACATCTTAAGAATCAGATTCATACTTA
GATTATGTGTTAATGGATACTTACCAATAAAAATAAAAGTGAACATTAAATTTAGGAGCTAGG
AAGTTGAACGCTCAACCAAGTTAATTAGTTCGATCCGTTGATCACGATATAAATCCTTCTTA
ATAATCAGGAATTATCCTTTTTTAATTTTTAAATTTTTTGGGTACGAAACTAGCTGGGAGATT
CCACTCTTAACTTTGGGATCCCAATAGAGTTGAATGCTTAATTAATAAACTAATCAATAAACT
TTAAAAAATGGGAAGATAAAATTAAAGCATGAAACATTCAACATTAGCGGAAACAAACTGCA
ACTGGTTTAATTATATTATCAAAGACCGAATACTTAATTATTTGAGGTCATGCTAACGAAGGG
CATAGATTAATAAATATAGTATTTTGAAAACTATTAAAAAAAAGTAATTTTTAAAAGAGTTTAAT
GTTTATACATGATAATTTAAAATAATTTTACATTGTTATTTAATCACAAATCATCATTTAATTATT
TTAAAATAATTATTTTAAAAATCAATAAATTTATCATATGATAAGTTATGATTAGATTATTGTGTA

AATTTTTTTACACTATAAAGGCATAACTCATTTTCTGTTTTTGAAAAGTAACTATTAAATAATT
TTATGTTTTTAATATTATAAGTGTTTTTTTTTTTTTTACTTTCATCGTAACGGGATCATATAACAT
TTGCTAATTAATTTAATATGTCAGAATATGATATTGCAAATTTTATCTATTTCAACATACAATTT
GTACTCAATGATAAAAAAAAAAGTTAAATTAATTTTTGTTCACTAATTTATATTTTAGATTCAATT
TGACTCTTTAATTTTTTTTGGGATTGATTTTTGAATTTTTTTAAAACGATTCAATTTGATCATATC
ATCAAAATTGAGTCAATATCCTTAAAAATCATAATTTATAACGATCTTAAATCGCATAAAATATA
TATTTTCTAAACGAAAGGATGAAATTAAATCAATTTTAAAAATTATAAGATTAAATTGTGCCAA
AAAAATAAAAGATTTAATCGAACCTAAATAATAATTTTGAGAAAAAATAATAATTTAACCTTAA
AAAAAACATACAATTTGTACTCAGATTCATTCCAATGACTTCATCACTTTATACATACAATGA
CTATTCAAAATTCATTATATATACCCAATAATTTGTACAATTAATGCGTCTAGATTTAAGGGTAT
ACAATTTTATTTTATTTTTTTGCTGAAATTAAGGGTTTACGTACATTTCTAATCTAGAATATATA
CGTGTGATGATTCTTTTGCGTCCTATTTACGAAAAAAGGAAAAGTCAATGCCTGCATGTGT
GATTCATTTTAAACTATGTTTTGTTTTGAAAAGAACTACGTATTGATATGATATAATCATAACC
AGTCATGGTATACAGTATACACAAAAAAAATTATTATTTCACGAAAAGCCTTAATTACCAACC
GATTATTTCAGCTAACAAATGATAATTAAAAACGAAATAGGAAAACAAACTCTTAACATAAAT
TAATCGGCCATAGTAGCACTTTCGGGTATTTTGTTTCCTATCAAGTCTAGCTTTTTTTTTAAC
GCAATCAAAGTCTAATATTATATATATTTACATTTTTCAAATAATATATGCAAATGGATCAAGTC
TTGATTTATTGTTATTAATTAATCGTATATTTGTCTAATAATATGTATGGATCAAGTCTTGATCT
GTTGTCAATCGTATTAATTAATCATACCTTTTTATTATTCAATTAGCTGTTTTATAAAAGTTATT
ATTGTAATGAAACTCTTCATATTGACGTTGATCATATCAAAATTAAATTCATATAAAATATTAAG
ATAAATTCTTGCCGGCGGTCAATCAAAACTTAAAATTCGCTATATCACAGACTCCCGGTGAT
GTTGTCTACCCTTTAAAAATATTGTTTACCCATCAAAATTTAAAAGCTTATCTTTTGTGTGTC
TATCCCTTACAAAATAGGGTACGTAGAAATTAATTATCTAGCTCTCATCATTAATTTTATAGAG
TTTTACTTATTTAATTTCTATCCAGACAGATAAATTTTAAGCACGAGTTTATTATTATGTAAAA
GTTTTTTTTTACTGCATTATGTAAAAGGTTAAACTGTTCATTTCGATTCTTGTTCTTATCTTAA
GTTTTAATTCTTATACAAGAAAATTATAAGTTTTAGTTTCTAGCTAGACAATTTTTTTTAAGAT
TTATTACCAAGGATTTTTTTTTTTTATAGTAATTTTATATCATCAGATAATTTTCTCTAGTATTAAT
GCCAAGCATCAAAAGTTATCTTTTATCTAAAACTTACAATTTTCTAGAATAAGAGTAAAAAGT
TTAAAATATAGACTATAGAAATCAAATGAATAGTTTAACCTCACGTAAATTTAAACATAGGGTA
GGATTTAATTTTCCCTTAATACAACCAACATATTCAGTCAATTAATCTTTGTGCCAAATGTTG
GCATTGGATCTTAATTTGTGAATAGGTCTGGTTCAAGGATGGGCTGCCATAGTGATGGGA
GTTCTTTCAGGCAGTGTCCCATGGTTCAGCATGATGGTATTAGGGAAAAAGCTGAAATTGT
TTCAAATGGTTGATGACACCCTTGCTGTGTTCCACACTCATGCTGTGGCTGGCCTTCTTG
GAGGCATACTCACTGGCCTATTTGCCGAACCTCGTCTGTGTGCACTCTTTCTACCTGTCA
CCAACTCCAAAGGAGGAGTCTATGGAGGCCCTGGTGGAGTCCAAATCCTTAAACAAATCG
TGGGAGCTTTGTTCATCATTGGGTGGAACCTTGTGGTCACTTCAATTATTTGTGTGGTTAT
TAGTTTCATAGTTCCACTTAGAATGACAGAGGAAGAGCTTCTCATTGGAGATGATGCGGTT
CATGGGGAAGAGGCTTATGCTCTGTGGGGTGATGGAGAGAAACTTAGCATCTACAAAGAT
GATACCACTCACCATGGAGTTGTGTCTAGTGGTGCCACTCAAGTGGTCTAGATGATCGAG
GAGTTGTACTTGAATTGAATCAATTGATTCTTTGTTGCTAATTTGTGTCATTCCATAATGTTT
CTTTAGGATATAGCTGTTGAAGAAAAAGAAATCTGATTTGTTCTGTCATCTGTGTTTGTGTT
AATTAATGCCTGCAATGTTGATTTTATTAGTATCATCTTAGCGCTTTGGTCTCAGACTCTCT
GTTTAATGATTTCTTCACAGATATAAATGTACCAATGGGCAGTCTTGGTAATTATGTATTTGA
GAATTGAGCTTATGAGCTTGGAAGTTGCATTTACGAAATTTCGGAAATGAGAGTAATTTTAA

TAGTTTGGAATATTTCGCCGTTTGGGGCTAGCTCTATTCTTTTCTTTTTTATATTCAAACTAT
TAAATGTG
>GmAMT4.3
ATGGAGTTTCCTACGAACCTTTTGCCAGACGAAGCGAGCCCAGAATGGATGAACAAAGG
AGACAATGCATGGCAGCTTATGGCAGCAACTGTGGTGGGTCTCCAAAGCATTCCGGGGC
TGGTGATTCTCTACGGAAGCCTAGTGAAAAAAACATGGGCCATTAACTCTGCTTTCATGGC
CTTCTACGCCTTCGCGGGGGTACTTCTATGCTGGGTGGGGTGGGGATTCCGCATGTCAT
TCGGCGAGAAAATGGTGTTCTTCTTGGGGAAACCCGGGGTGGCGGTGGACGAGAAGTT
TCTTCTGGGGAAAGCTTTTCTGGGGCTCTTTCCCAATGCTACGATGGTGTTTTTTCAGGG
GGTGTTTGCGGGGATCACCTTGATCTTGATAGCTGGGGCGTTGCTCGGGAGGATGAACT
TTCGTGCGTGGATGCTGTTTGTGCCTCTTTGGCTTACTTTTTCGTACACCGTCACTGCCTT
TAGCATTTGGTGCCCCGATGGCTGGTTGGCAAAGCTTGGAGTTATTGATTTCTCTGGCGG
ATATGTTATTCACCTCTCCGCCGGAGTTGCTGGTTTTACTGCAGCTTATTGGGTCATTCAC
TTTTCTCTTATTTATCACTTTTACCCAAAAAATTCATTTAAATAACTAGTCGTATGTCTTAGTA
ACTCATTTATAATCGAAATTATGAATAAATACTTAAATCATTTTTTTTAAAGCAAACTCTTACTA
TAAAAATTCATAATTGTGTTTTAAGTTAACAAATAAATTTTGTGATATATTTTTTATGTTGATCC
AAATGAAATTGGGATCGATTTTCTTATATAGAATCGTATTATGAATGAATAAAATGAATTAATA
ATAAAGACTCTATAATAATAGGTTCTTTGTCTAATTAATTGTATCCAATTATTAATGTTAATTAA
GTAAGTTTTTTGAAAAAAATTAATCACTAATAAAGATTAATTATTGATAAAATTAGTAAATACTT
TATTGTATCAATAACTTAAAAAAATTATATAAGGTACTATTTTACAATTTAAAGAATTTCTTTAA
TATTATAATTTTTAAACATGTTTTTTCTTTAAAAATATTGTTGCTAAAATAAAACTCAAACAAAC
GGCCCTGTATATAATACCTGAAAAGAAAATGCAGATCTTTTTTTTTAGGGGAAATGCAGATC
TTGTTAACTATCTGCACTATAAATATCATAATTGGAAAGTATATAATAATTATTTTGAGTTATAC
ATATACAACACCTAATTAAAATATCATATATGATGCGCGAATATATTTTTCATGTTTCAACTAAA
ATATAAATATGGGTGATATTGTTTTTAAAAAAATTGAATGATTATGGTACACATAATATTACCAA
ACAACTTTTGAAGTAAAATTTAATTCCACTCTTAATTTTATTTATATGTACAAATATACAATGAA
TATTCAAGAGAAAGGAATAGAACATTTTTTGCGTGAATAATTTGAAGTAGTTAATTTTCTTTA
TAAAAAAAATGCTGACATTTGCATTGCATGTGATGGTGGTTGAATAGGTGGGTCCACGATC
TGAAAAGGACAGAGAAATATTTGCATCAAACAACATGATTGTGGTGCTTGCAGGCGCAGG
CCTGCTTTGGATGGGTTGGAGTGGGTTCAACGGTGGAGGTCCGTTCGTGGCGAGCACG
GTGGCATCTCTGGCCGTCCTTAACACACACGTGTGTGCTGCTGCTAGCATCATCGTCTGG
GTCCTACTCGACACTTTCTACTTTGGTAAGCCCACTGTGTTTGGCGCCGTACAGGGCATG
ATCACTGGTCTCGTTTGCATCACACCTGCCGCAGGTACAAACACACCTTTTTCCTTCAAA
CTTGGACCTGAATAATAATTTTAAAAATCTAGATAATGATATGATTTTTATTTCATTAAGAATAA
AATATGTTTTTTTGTTTTTAGTTTTTTAATTCAATATTTAAAGTTTTTTTTTCCATTTTTAAAGA
AAACACAGGACAATGTTTTAAGCTACCATTTCTTATATTTCTTTTTTGTCTTTAATATTTTTAA
CCTTGTATTTAAAGAGAAACATTTTATGAAATTTTCATTTTTAAAGAGAAAATAAAAAATAACT
TTATAAAACTTTTAATTTCCATTTTCAATTGAAATTCTACTTTTTCTCATTTTTATTTTATTTATT
TTTCTCTTATTGAAATAAACAAAATAAATTTTTGTTTTTTACTGATCATTTATCGCATTTTTTTT
TTCTTGAGTTATATGTATAGATAGTATGTGCGTCCAATCATTCGTCCTTTACAATGATGTCGC
CTGCTTTTGCCTAACCTCTCCCTTTTCATCATTTTCTATACACCAATTAATACATTTCACAAA
ACATGAAAGACAAGAGTAAAACCAAATTACCTTATAGGAGTTTTTCTTAAAGGCTAAAGTGA
AATATTATTTATAGCAACTAGCTAGTTTTAGCACAAGTGGTGCCAACCTTATAGGAGTATCC
ATTGTAAACGGATTTCTTTTAATCCGACATGATTTTTTTTAATGTTTATATATGGTGATTATAG

GAGTATCCAATGTAAACGAATTATTCTTAAATAATAAAATGCTATGAGTATAATGTATATAGAA
AATAATCAAACAATTAAAAAGAATTAGTATAGAAGTAAAAATAAATGTAGGAGCATGACAGAA
GAAGAAATTTGATGAATCCTGAGAGAATTGTAAAAATAAAGATTCCATTCCGTGAACAGGA
GTTGTGCAAGGGTGGGCAGCGATCTTGATGGGTGTGATGTCAGGAAGCATTCCCTGGTA
CACAATGATGATCCTCCATGACAAGCTCCCATTCCTAAAGCAAATCGACGACCCCATGGC
GGTGTTCCACACCCATGCCGTCGCCGGCGCTCTCGGCGGCGTCCTCACGGGGCTCTTG
GCGGTGCCTAAACTGTGCCGTCTCTTCTACATGGTCCCCGATTGGGAAAAATACATTGGA
CTCGCCTATGGCCTCCAGAACGGTGCAACTCATGCGGGTCTAAGGCAAATGGCCATCCA
AGTTGGGGCCATAGTTTTCGTTATTATCTTCAACTTTGTTACGACCAGCTTGATTTGCTTGC
TCGTGGGGTCCATAGTACCACTCAGGATTGACACCGATGCTTTGCAAATGGGTGACAAGG
CAATGCATGGAGAAGAGGCTTTCGCTTTCTATGGAGGAGACCCTACAGCAAAGTTTGAGA
ACCTCAAGCACAATAAAGTTTATGATACTCAAGACTTTTCGTTTGTTAGAGACACAAGGTC
ATTTGCCCAGCTTCAAATGGTATAA
>GmAMT2.2
GAAGGGTGAAAAACCTTGATCAAGAATTGAAGCATTAATCTTCAACCATGGCCACACCCTT
GGCCTACCAAGAGCACCTTCCGGCGGCACCCAGTTGGCTGAACAAAGGTGACAACGCA
TGGCAGTTAACAGCAGCCACCCTCGTTGGTCTTCAAAGCATGCCGGGTCTCGTGATCCT
CTACGCAAGCATAGTGAAGAAGAAATGGGCAGTGAATTCAGCTTTCATGGCTCTCTATGC
CTTTGCAGCAGTTCTAATATGTTGGGTGCTTGTGTGTTACCGCATGGCCTTTGGAGAAGA
ACTTTTACCCTTCTGGGGTAAGGGTGCTCCAGCACTAGGCCAGAAGTTCCTCACAAAACG
AGCCGTAGTCAATGAAACCATCCACCACTTTCATAATGGCACTGTTGAATCCCCTCCAGAA
GAACCCTTTTACCCTATGGCCTCACTTGTGTATTTCCAATTCACTTTTGCTGCTATTACCCT
TATTTTGTTGGCTGGCTCTGTCCTCGGCCGAATGAACATCAAGGCTTGGATGGCTTTTGT
GCCTCTTTGGTTGATCTTTTCCTACACTGTTGGGGCTTTTAGTCTTTGGGGTGGTGGCTTT
CTCTACCAATGGGGGGTTATTGACTATTCTGGCGGCTATGTCATCCACCTTTCTTCTGGAA
TCGCTGGTTTAACTGCTGCTTACTGGGTACATAAATTTTACTCTTATCATATTTGCTTCAAAT
ATCTAAAAGCTAGCTAGCTCCCTTAGCCAAAGTTTTAAATTGCGGTGGCGGTTGTAGTTTT
GGTCACGATTTTTAATATCGTGATAAATTGTGGAAAGAAGATGTGGGCATGTGACTCTGGT
TTTATTACGATCTTTGATACTGTGAGAAATTGCAAATCAAACATCTCTTTAAAAGCATGCTCT
TACCTAATGGGAGTTGCTATGGTACCTAAGGAAAAAATTATCTTTGGTTGTTGGTTGTTAGT
TTTCAACAGAGATACTAAAATTGTTAGTTGTTGGTTGTTGGTTGCTAGTTATTTTCATTTTGG
TAGAAATAACCGTTTTTTATTATTAATATTTTAAAAAAAGATATATTCTTTTTACCAATTTTGATA
GTTATTAAGAAAAATAATGTTATTTTCCAAAAGCAACAATTTCCAAACTTGTTAATGTTAATC
AACCAATTAATGTTTGGTCATTAATTTTGGGCACTAAAGGAAACCCTCATGCTGATTCATTC
ATTTATGTATGCCAACTTGGTTTTTGATTGTGGGTGCATTAAATATGTTGTACATGTGCAGG
TTGGACCAAGGTTGAAGAGTGACAGGGAGAGGTTTCCACCAAACAACGTGCTACTTATG
CTTGCTGGTGCTGGGTTGTTGTGGATGGGGTGGTCAGGGTTCAACGGTGGAGCACCATA
TGCTGCAAACATTGCATCTTCAATTGCGGTGTTGAACACAAACATTTGTGCAGCCACTAGC
CTCCTTGTGTGGACAACTTTGGATGTCATTTTCTTTGGGAAACCTTCGGTGATTGGAGCT
GTGCAGGGCATGATGACTGGACTTGTATGCATCACCCCAGGAGCAGGTATAAAGAATTGT
CCTTGCTTCTTAATATAATAATGGTCACATTTATGACTACTCAATGAGATAAAATACAAAACT
CTTGCCCATTAATGTTTTGAAAAGTAAATAATACTTTTTTTATTAAATAATACTTATTTTGCTTT
TATTTTGCAACTTGGGTTATATTGAATGAATCTCTCTCATTGGATTAAGTACTATTGTCAAAT
GTTTTACAATTAGTATACTAGAGTTTAGTTAAAAATTTAAAGAATTAGCTCAATTGATTAAGAA

GAGTATGAGAATTACGTAGAGTATGAGGATTATTGTAAACTTTCTTTAATTCTTGCAAATAAA
AGAAAATGAAAGTTTAAATTCGCTGGCAAAACATTATGTTTACATAAACATTGAATGAATAC
GGAATTTAATTACTTTATTATCACGTTACATTTATAAATATGATGGCGTATAAAAATATTTTACT
ATGTATATGAATTAAACTCTTTTAATTTTATCCAACAATATTAAAAACAATGAAAACGATTCCA
TTGATTCTGTTTTTGACAAATAATATGAAATGTGACTACAACTTGCAGGGCTTGTGCAATCG
TGGGCTGCTATACTGATGGGAATATTATCTGGGAGCATTCCATGGGTGACCATGATGATTT
TGCATAAAAAGTCAACCTTGCTACAAAAGGTCAGGGTTTCTTAACTCACATCACAAATTTTT
CCCCACAAAATACTTATAGTACTCATTTTATCCTTCACTTTCATTTTGTCTTTTTTTTTTCATC
TCTTTTTCCATTTTTATCATATCATTTGTTACATCTATTACTCACTCTTTCTCTATATTTTTATTT
ATTTATCTTTCCATCCATAGACTCTTACATTTATAATTTAAGCATAGACTTTAGGTGTAGCTTC
ACTTTTGCAGTGCACGTGTTTGTGAATATTCATTTAATATTAGAAAAAAATAATGGCCTAAAC
TTCAGGTCATCCGTTGATTCCAACCACTTTCATTTTATTTTGCCCACTTAGGTGTCATTTATT
ATTATTTTTTATATAAAAAAATAGATGATGTAAAGATGAAAGTCATCCTCGTTTTCTCGGGGA
GTAAGGAGTGGCTGATGTCACCTCATTTTTAAAATTTTAATTGATTTTTATTTTTAAAAAAAT
CTTTTTTTAAGTAAATTAATATTTAAGGACCAGAAGTAACTAACCTGTTAAAATGACGATAAA
CAGTACTACGCATTTACCTTGTTCTTCGATTTTTCTCTTCATTGCTTTAATAGATTCCTTGAA
TACTTTTGTTTACCATAATGGCGTGATGACATTTTCGTCTAAGCATAATCTAGCCTTGCCTA
ATTACTCCACAAAGATCACTATCATACCACCAAACTTCAACTACTCATTATTAATTACATTTT
GAATCTTATTTTTCATTACTAGAAAAGCACTTCGTGGATCATGCATATTTAGTTAGTTAGGTC
GCCTTCCATTTCTCTATTTATATCCACCTTTAGTTATATAAATTTTATCGTATTTCATTAACCAA
CTTGAATTTTTAGATGAAAATTAATTTGGAATCTACATAACTAATCACATACGGAATGTAATAA
GAATTAGTACTTTTATAAAAAAAAATTACACTATTAAGTAACAAGATATTGTTATATATTATTTTT
CAGCAGTGCTATATAGCTATATAGTACGATAACTATATGCACGAACCTAGTAGGAAAACATG
TGCCTAAATATTATTGGTTAATATAAAAAATAAAAAATAAAATCATTGACGGAAATCAAGGATA
AAGGTAATTAATTATAAGACTCTTGGTGCATATACACAAATTCGTGCATAATAGTATTTTTTAT
AATTTTTTTCTTAATTTTATAATAACTAACTTAAACCTATTATAGCATGACTTATGATTAGTTCA
CTGCATAAAATTATTTTAAACTCTTTTAGCCAATTATATCTACATTTATAAAAAAAATATTATCA
ACTATATAAATGTAAAACATTTTTATATTGTTACTTAATTATTGTAATGTGTAATAAATTTATTGA
TTTTTATAATAATTATTTTTAAAATTATATTTGATGTATTTTTTATTGGTTAACCATACCCTGACT
ATATATATTAAACTCTTGGTATTTTGCTTAGTATTATACTTTGTCCTTAGCATAATTTTGTAAAA
TGTATTTACATTCAGGTGGATGACACCCTTGGTGTGTTTCACACACATGCTGTGGCTGGC
CTTTTGGGTGGTCTCCTCACAGGTCTATTAGCAGAACCAGCCCTTTGTAGACTACTATTGC
CAGTTACCAACTCAAGGGGTGCATTCTATGGTGGTGGTGGTGGTATGCAGTTCTTCAAGC
AATTGGTGGCGGCCATGTTTGTCATTGGATGGAACTTGGTGTCCACCACCATCATTCTCC
TTGTCATAAAATTGTTCATACCCTTGAGGATGCCGGATGAGCAGCTGGAAATCGGCGACG
ACGCCGTCCACGGCGAGGAAGCTTATGCCCTCTGGGGTGATGGAGAAAAATATGACCCA
ACTAGGCATGGTTCCTTGCAAAGTGGCAACACTTTTGTGTCACCTTATGTTAATGGTGCAA
GAGGGGTGACCATAAACTTATGAGTCAAGAAATTCGGCTGTGCTTTGCTCACACATATGTA
TAAAGTTATGTGATGAATCCGTGAGTGGTGTAAGTAGAAATTTGATTTTGTCATGAAAGAAA
ATTCAAGTTTTGAGATCTGATGTTCCTCTGGCCATCCAGCATTCGAAGACCTGATCATATAT
TTCTGGCACAGATTGTGTTGACATGTTTATAAAATTTAGATTTGTCAATTTTTGAAGGAGCT
TGTGATTAGTTTTCTTTTCCACTTATATGTTTTAATTACTAGAAGAATATCAAATTTTCTTTTTA
CGAAATGCTTAGTACATAATTGTTAAAAAAAATCATCATGTAATGGGTACGAAATATTTATCA
ATTCTATGAATGAGTAATTTATACTTAGATAACTTCAGTGACCACTTTTAGAAAATTTATCCTA

TGTATAAATTTTAAAAGAATGGTTTTAACTCCAAAATTTTCACCTAGTCCTTGTCAAACAAAT
TTTATTTTGGCTCACTTAAAGGTAAAATTATTTAGTTATGCATTTCAGAATGAAGTTTGGTTC
GAAATATTTTGACAGTGTGTCAAATATAAATTCTTCAAAAGAAAAAGCCAAGACTACTTTAC
AACAAAATAGATAAGTTTCTCATAAACTGAGCACAAGTTTTCCAAATTGATGGTACTACAAA
TGTACTTCCTTCCAAAGCCATTGACTTTGGAATGGAGCGACTTCAAGTATATACTATAATGT
GGAATTGAGAAAGTTCAAAGTTAGCGACGAAAG
