# Supplementary material for: Genome-Wide Identification and Expression Analysis of the Ammonium Transporter Family Genes in Soybean
Source: Int J Mol Sci. 2023 Feb 16;24(4):3991. doi: 10.3390/ijms24043991 (PMC9960152; doi:10.3390/ijms24043991)
Supplement: Supplementary file 1 [file ijms-24-03991-s001.zip › Supplementary Data4.pdf]

Supplementary Data 4.

The promoter sequences of *AMTs* in Arabidopsis and soybean.

>AtAMT1.1

CTGGTTGAGATATCACAGAAATTATCAATTAATATCTTATAAATGAATTCGAAATATGCTAAAT
GCAAACATAGTTTTGTAAGTACGTAATTTATAATATATTAATACTCAAATCTAAGACTTACTTTT
GAAAACAAAAGAATATCTTGTCAAATTGACACTTTTTAATGTTCAAAAGAGATAGTAAGAAA
TTTCGCCTAACCAAGAAAACAATAAAAACAAGGAATTTCAGTCTCAATACTATATATATTTTT
CTGTTATTCACTTATATATTTGGATGTGATAGAATACTTTTTTAAGAACACACATATCTTATCT
TCACCGATATATTAGCTGATACATTTTTTACATTCCTTTTTATTATTATTATTCTTATTTATTTTC
TATTCACATGTACTTACAAACAAAAACTTAAAGACTTTGCAATTTGCGTTACGAATATTTTTC
GTCAGGCTGTAATTTGAAAAGTCTTCTAGATGATTTCAAGTCTCTGCTTAATGTGCCAACTA
GGCTCTATGTCACTACTTTGTGTTGTCTCTAATTCTCATTCTTCTTGACAAGACCAAACATA
TCACTAATGATGAAGAGATGACTAATATTTGTTTATACATTTGCTTGTAATGAAGACATGTAG
TATAGCAAAGCACCATATAATATAGTGATAGCATTCTATTAATTTGTTCTTTTTTACAGCCGAA
GAAGAAGAGTATAAGAAACATGTTTAAGATTCAGCCTTAAACAAGTGAAGAAAAAACAAAA
AGAAAGGCTCAACGAATGGGAACTTTGATCCAACCATTGGCAAAAGCAATGGCTAAGATG
ACGAATGGAGTTGACATACCGAATATGATAATGTAAGGAAGAGGCGTCTTCATCGGATTCT
CTCCTTCTCTTTCTCCAACGCTTTGCCAATACCTACAAAATTCCAATGCAATCCTCATCCAT
AACCATTTCTTCAATCACAATTGCATGTTTATGAAAAATGAACATGCAAGAATTTAAGTTCTT
TTTCTATTTTAAAAGAGTTTAGTTCTTTTGACTAAACTACAACCCAAATATTTTTATTCTTTTG
CTAACACGGATTTGAGTCTGGATATTGATACAATACTAACACAATGTCACAAACCACCAGTC
TACATGTCATTGACACATACAAAAAAAAAAACTACTTAAATTCTGCAATGGAGACTAAAATTT
TTGTATTAAATTGGTAACCTACATATCGTTTTGTAATTTTCCATGTTTTCATCAATTTCCTAGG
TTTGTTTTTCTGTGATTTTGCAAAATTACATTACCATTCTATGAGTTTCTGTTATTTTCTACAA
TCTCATGATGAAAATTGAGAATTAAGATTAACTTACTGTTCAGGTTCTTCCTCTTTGGTTATA
AACTTTTTCTTATCTTTAGGCTTCACTTCTCCATCTCCTCCACCTTATGCAATAACCAAAGAT
TAACAAAATTTACATCTACAAACAAATAAAAGATATAAGAGTACCTAATTTGGCATGAAGCTT
GAATTCTGGGAATCCTAATGGTGATGGTGGACAAAAGAATTGAGGTAAAGCAAGCAATGA
TGAATATGAAGAAGATGATGAAGTTGAGGAATCACTAGCCCTGTGGCTAAACGAAGAAGA
AGAAATTCGAAATTTCGCTGCCATTTCCAACAACTATATGGATGTGATAGAATATTTTTACAT
TTTATTTTTCTGTTATTCACTTATACATTTGGATGTGATAGAATATTTTTTTCTGAACACACAG
ATCTTATCTTCACCGATATATTAGCTGAAGCCTGATACATTTACATTCCTTATTTATTTTTTATT
CACATGTATGTACTAATAAACAAAACTTAAAGACTTTGTATTATGCGTTACGAATATTTTTCG
TCAGGATTGAAAAGTCAACCCTAGGCCTTTAGCTTCCTTCTTTTATATAAACAGAGAAACAA
AACACTTTCTTTCTCAGCCTCTCTGTTTCATCTTCTTCTCTAAACTCTCAAC

>AtAMT1.2

CTGGAGACAAAACCCTATGTTAATCGTATAAACGTTTGGTAAGTTTGAAGGATGAAAAGAA
ACGATTATCTTCCAGGAGTAGAAAAGAAAATTTTTGGAAGCTACGAGGACTAGATCTTCTT
GTGTAGAATGACATAAACTATGCTAGTGAGCAACACGTCCTTCAGCATTTGAATGACCGTC
AAAGTTATATTGTTTACCAGAAAAGTGTATTTAATCACGTGGTTAGCGGTTCACTTTTGCGG
CAGTTTTTACCATATTTTGACTCTAGCAAATTTAGTGGCGGCAAAAAACCGGAACCGATTA
CTCAAACTGGAACCGACCCGAAAAATTAGATCCGAATTCGAAGATTTATCTTATTTGTCTTA
AATTTCTCTACCAAAAAACCGAATTCGAATTCGACCCGAATCCGAAAATCCGGGTATTTATT
CGAAAATTTGGGTATCGATTCCAAAAAAGGCCTGAAAATCTATATTCGAACCCGATTGGAC

CCGAACCAAAATCAAACCGATATTTTAGAATTACTCTATTGGATCCTAAACTTCTCTATCCAA
AAAAACCTGTACCCGATCGGATTTTATCAGAACCTGACTCGAATGCCCATCTCGCATTATC
CTGCATAGTAACAATTGGTTTTGGATCGGATCTTGCTGATAACACTCTAATTTTACCCATTTT
TTCATAAATCGCATTTATTTTTACCCATTTTTTCATAAATCGCATTTTAAAAACATTTAACGTA
ATATAAAAACATTTTCACTGTTCATATGTCCATTCATTTTGTTTTTTTCTTTGTTGCGTGATTT
CCTACTATCACTGTGATTCCGTGAAAACCTAATACGATTTTACTATTGAATCTGTTCATCTTT
CCTTTGTTGCATATATATTTTTCCTGTAATCACAAAATTGAACCCTATATATAGTTTGTAGCTT
TAACAGTTTATTCTCTAATACGTAGTGCAATATAAATTCTATGTGAAATCGGTACCTCAAATTA
CAACATATTTGTTTGATTAACTAAAAATACAAAAATAAATGGCGCTAAAAATCACACAGCATC
AAATAATGCCTATTATTCAAGTCGAATTACGTATTCAGTTGTGAAGTAGTTGTAGTCAAGTT
GAAAATAATTTTTATATTTAATCTAGCGTGATAATTTTATAACTATTTGAGTGATCGTCCTTGC
CCCCTATATCACATTCATATAAATGATACATCATTCATGATCTATCAACTATTGACTTCATCGA
TACAAACGAAACGAAAAATTACATTTTTCAAATTATTTTAATTCCAAAAAAGAAATCAAATTAT
TTCTCCTTTGAATCGTGCCGTCAAAAAAAATAAATTATTTCTCCTTTGTAAGAACATTAACTA
GTTCATATAAAATATTGAGTTGGAAAAATAGACATACAAATTCATTTAAAAGAATCATATCGTC
ATATGCTTGAACTTGTTCTTTAATGTCGTACTTCTATGTAGCGATGTTTAATTAATTGTTTCTC
ATAAACAAATGCTGACCCGATTCCTTGTCATACTAAAATAAACGATTCAAACACTAAACGAA
ATATTATAAGAATTTTCTTTTTTCTTTTTTTATTCGTATAACCAAAATTAATTGCACACAAAAAC
TGATTAAATATCTTATTAAAATAAAAAAATCTTCTTTAAAAAATTAACTTTAAATATGTTCAAAA
CTGAATAGAAACTAAAACAAAACTATACCAAAACAAATTTATTAATTTTATTTTAGTTTGATTA
CTACTGTTTAAAAAAACTTAAACCAGAGACCTAGAATTTTAAAATTCAGAAAACTTCTTTTAA
AACCTCAACTAGAAGAAGAGTAAGTAGTACCGCAACTTTTTGTGATGTAACGAAATAAATAT
GCTTTTTTCCACTTTAAATACACCAAAGACACTCTCACCACAAGTACTCACTTAAGCAAAC
ACGTTCCACAATCAAGTACCCTCTCTCTATCTCTCCCTCCCTCCCTCTCCACC
>AtAMT1.3
TTAAAATAATCTTGCTGTGAATGTCTACTCGTTGAACTTTGAACCAAAAAAAAAAAAAAAAT
CTAAGAAGTATAATAATTAGAGACTAATAGAGTCAATAACTCATTCTTTAAAATATATAGAACT
AGCTGGAAACAATAGATTTGAATGGTACTACTTAGAATCCAGCAGGGTTCTTTAAAGTCAT
CATGAAGCAATGCAAAGTCTACCACTCTTGGCGTAATTGTTTTTTGGCACAAAAGTTAATG
CAAAAGTCTTGATTATCGCAATTTTTCTTATGATGCCTTTGGTTTCTAACATCAGAGGATAAA
AAAAGAATTTCACGAACAAAAATTGTGAATTTTGATATGCATAGAGGCGAGCTATAGACTCC
CTAACGTGCTATTGCGTCGAGCCGTGGTGCTTACCTAGTTACACAGTATAACAAGCCTGCT
TTAGTTTTATTTCATAAACCAATATAATGTAAATCACTTACTATGGCTTTTCGTATTTAGTAAA
CAATTTTACATTTATAATTTTCAGTTGACCTAAATTAAAAAATATGTACTACAATTAATCAATAG
TTACTACGTATTACTAAAGAATCAATATTTGTTATGTGGTAATCAATGATGTCACGTCAATTCT
TGACATTATTTTGTACGATTTAGAAAGCTACCGTCTACTAAAATATGTGCATAATTTAACGTT
CATTATTTTAGAAAACAGTTTGCTTTGTCTATTTTTCTGAGTGTCAACTCTCATTTACAACTC
ATCAGTAAATATATAGATAACAAGTCACAAGTGTCTCCCATAATGATTAAGTTAGGTTGACTT
AAAACATGATTAAGGTCAGACACACACACACATACATATATAGAAAGCTTTCAGTTTTTAGA
ACTAGTTAGTGACAATAAAAAGCCTATTGGTGAAACATCAGCACTTGTAACAACTTTTAAGT
ATATTTATTCAAATACATCATAATAGATTTTTGATCAAAAGAGATCATCATGTTCCTAAAAGTA
ATACTTGACTTGAATTTTCATGGCTTGGCACAAACTGAGAGATAAATGTCTTTTTTCAATCA
TTAAATATCTTAAAATATATATATATATATATATATATATATATCTTAAACATGAATGTTTTTGTTTT
GGTATAAACGTGAATTATTCATGAACTGTTAATAGGTTTCAATGTCAATTGGATCGAATTATT

GTATATATCTCTTGCTTGAAACTATATGTCGTGTGTCTTCATTTTTTTGTTTTCCTAATTGAAT
ACTTTATACATGCATGTAGTATTATTATACAAAACCAAGATAATTTTTTTTAATGCTTCTGTTG
ATGATCTGAGTAATTTTGGACATATCTAAGAGTTAAGGGTGTAAAGTGCAATTTTCAGCAAA
TAAAACGTGGTTCCCGCTAACCCATGTTATCATTTTTGGTTTCAACCTTCAGCAAACTTAAT
TAATATTTGGTTATGAACTTTATTAAAATTTTGCAAGATTATCATAAGATTGTCGTATAAAGAG
TGCTGAATCTTGACTTAATTTCATAAAACTGCTGATCTAAAATCGACATCTATGTAGTAAGTT
CTGATCGGTTTGCTTGAACATATACGGTTGAGCTAGGACAATTATTCTTTGATTTATTACTTT
AAATACTAGTATCAATTTTATTCACAAATTTATTTTTGTAATTTTTTTATACAAGCCTTTAATTTA
CCTTTTTTTTATTCACACATTACATGGCAATCACTTAATCACCAAAAACCGTATAGGATTAGG
ATCGTAATTTGCAAGTTATTAAGAAGATTTGGCAGATTACATCTCTAATTCAATAATGTTGTT
ACGTCACATAACTTCTTAAATAATTGATAAACTCTCTTAAATACGAACTTAATTGGTCTAGCC
GGAATCTAAAGTCAACCCTAAACTCCTCCTTCTTCCTTTTAAGAGACACACAAGAGACAAC
CTTTCTTCTCCGTATCTCTCTTTCTCTCTCTCAGCTCTCTCAAAC
>AtAMT1.4
AGCTTGTAGGGATTTTCCGCATCCGCGACATGCTTGTGCTAAGTACCCGTTCAAATCCAC
ATTACACCAGACGTTTTGTGAGATGGTAAGAATGTGAAGTTATTTGTTTGTTCTTTTGTTTT
CTAAGAATCTTTTGAGACAGTTTAAGTGATGTTTCTTTTCTTGCTAATGACGTTTTTTGTTTT
GCTTTTTGTTCTTTACAGTGTCACTGTTATGTTTGTGACACCCGTGCTCCGTGTACTTACT
GGTTCAGTGGCGGGATCTCTAACACTGATCACTGTCATGCCAATGATAAAGAGAAGACAT
GGAATGATCAACGGGAATGCATCAGAACTGGAAATATGTTACCTCGACCTGTTTCGAAAC
CTGCTTCGGCCAAATTACAATTTAAACGTGCTCCACGGCGTCCATCTGTATATGTACCTGT
ACCGTTGCTCCAAAACTCGTCTGGAACCAACTTAGGAATCCGGGCTTGCTCCAAATCTAC
TAAGGTTGCAATTCACCCAAAAACATACACTAGACCCGGTCATAGAACCGAGCAATCAAGA
ACCGTCCAACAAAATCCTGGGTCACAACCTCACGCTATGCAGTCATTACCAAATCACAAAA
GTGGCTCTAATAATGGTAATCCAAGTCCTCAAGTTGTCCCTTCCAATCCATATGTATTGACT
CGACGGCCAAGTAATGGCGTTTATCCTCCGGAAAACAGTGTACAGAATATTTCTCAAGGC
TTACAACCTACCTATTATGCTCCTCTCATGGCTTCACAAGGCTCACAACCTACCCGCTTTG
CTCCTCCCGTGGCTTCACAAGGCTCACAGCCTACCTGCTGTGCTCCTCCCGTTGCTTCA
CAAGGCTCGCAGCCTACCCGATATGCTCCTCCCGTGGCTTCACAAGGCAATGCACAGCG
GATTGTTACTGGTTCTATCTCAACTGTGCCGGTATCTCAGAGCAAAGAGGCAAAACAGTTT
AGCCGGAATATGTATTCTGCTAATGTTCAGACGAATGCAGTCCCAACTATGACTCCTAATC
CTCCGGCGGTCCAGCAGCAACAACAACAACAATCAGGAAGAAGTAACGATAGAGTATTGT
CCGAGTTTGAAGACTGGCTCTTGGACGACTCAACCTTGCCTTGTCCCTCGTCTGTTCAA
GACAATACATTGAAAATCGATTTCGAAACCTTCTTGAATGATTGAAACATTACGTTAGGATA
GTGTAGATATCCTAACATAATTGTAGACTTTTAGCTGTACAGTATTAATAGTGTAGAGTTTTT
GGTTTAGGAGATTTAAGTCTACGATAACATTTGTGATACCCATCTAAAATTCATTCTTTTAAA
AATATTCTTCAATGGATGATTTTTTTTTGTTTATGGTTCCTGCTTTATGGTAATTATAGAATGAG
ATAATCTAGTGAGTCACACAATTTAGTTTAATTCCCCCTCGTTTAAAGTTAATGATTTATATTT
GTCAATAATTCTTTTGGATTTTCTAATTTATTTTACCAACCCAAATTATAAAATTTGTCATAATC
TAATCCAGTGGTATTTACTACTTACCAATCAGAATTTATCAATTGAAAGAATGTAAATTAACC
TTTTTGTAGTCAAAGATATTTGTTCCCAAAAACAAAAACTAATAAAAATTAGTCCACACATTT
TTTAATGTTAGGACAATTATAGTCCACACATTACAAAACGTATTTTTCGGAAAATATAAAAATT
TAATTACTTTGGAAATGAGCAGTAAGTACGTTTAAGGAGGAGAAGTCAAAGTTAACACCAA
CAATAGTGGAATAAAACTGTTACAGAATCTTAAAATTTAGGGTTATAGTAAATACAATTATTAC

TATAGTGAAAATGAGAGAATAGAATCTGAAAGATTCAAATTCTCTAATAATGATTCAACAGAA
TGTCGTGAATCTCTCTACCTTCCTCCTCTATAAACTCTACTCTCTGACTCTTCTTCATCTCT
CACAAAATCTCTCTTAATCTTTGCAACA
>AtAMT1.5
GTAAGAAAAAATATTAAAATAATTACGAACCAGTTAAATATTCTTGCCCGTATAGTTTAGACT
TAGAGTTTTATTTTATTTTAATTATCTATCGGTAATCCGACATTTTACCACCCAACTAATTTCT
TAGAGTGATTATAGTCGTCAAGTACTAGTGTATTCACCAAATTTTCTAATAATAAAGAAAAAA
AATATAACACTATTTCGACTAGGTAAATAACAATTGATGTAGATAATTCAATGTTTTGTAATGA
ATCTTTTCTTGTTCTAGACCGACAAGGCAACGCTTCCTAGAATGGGAATCGCTAAACCACT
ACCACTTGATTTATATATTTGTTTACTACTTAGAATCTAAGATGGTTCTTTAAGATCCTCTAGC
ATTGCAAATCAACCAGTATTGAGTTAAATTGTTTTGGCAAATAGCCAAATATGTTAATGCAAT
TATTATGATTATCTATAGAGTTTTTTTTATGATGTTCTTGGTTATTAAACACTCAATCACTATTA
TAGAACATAAAAAGTAATTTCACGAACAAATATTTTATAGTCTGATATGCATAGAGGATAGCT
AGAGACGCCGCAATGCGCCGTTGCATAATTTCATAAAACAGAAAACGTCCCAATGTGGCT
TTTCGTATTTAGTAAATTTTTAGTTCTTATAGTTAATTGTCAGTTGACCTAAACTAAAATATAAT
ATGCACTATAATTAATCAGTAAATTAAACGTATTATCAAATAATTAATTTGTGTTATGTGGTAAT
CAATGATGTCAGGTTACTCTGTATGATTTTAAAAGCTCCCGTTGCAATTAAAATATTTATCGT
TCGTCGACGACATTAGATTAAAAAAAAAGTTTGGTTTTGTCTATTTTCCTGAGTGTCAAATC
CCAATATTAATGTATTCTAAATCGTATACTTCTTCGTTTTCCATCAGCTAATTTGTAATCTAGT
AAACTTATTTATTCTCCTTCGTACGTATATATTCACTAGTGAATTTACAAGTATGTCTTATAAT
GATTAAGTTTACATATAGGCATAGTTGGAACATTACTTATTGCTACAATTTTATCTATAAACTA
TACTCATTCTTATTCGACCATTGTGGTTGCTAATATATAGAATCACATTAAACGCCATTGATT
TGTTGAATTTTAAGATCGCAAGTGAAATTTTCAACTAATAAAACGTGGTTCCCGCTAAACCA
TGTTATCATTTGCAGTTTCGTCAGCAATTATATTAATACTTTACAAGTTCATTATATTTAAGATT
TTCGTATTAAGAATGCTGAATTTTGACCTGATTCCCAAAAAAAGAGTTGCTGATCTACAACT
AGCAAGGTGCCACAAATTGATTTGCTAGAAACCCTAGAAGTACTAATTTTGTTTATATAATT
CTATTAAAATAGAAATTAGAAGATACTCAGGTACATGTAAACGTTTTTTCCCCGAAAGTAAA
AGCACGAATGAGCTTCCCTTTCGTGTGAGGTGGTGTGACAGTGTGAGTCGAAAACGGCG
ACCAACTCTCTACGGGAATCTTCTTAGATTCCTTGGTCAAATATGTTTTGCTTACATATGCT
ACAATTGAATAGATCATATTTTTTATTTTTAATATCTTTTTGCAATATTATAATGTCGTCATTCAT
ATTTTGGGTTAAATTTGCTTCCATCAGCTTGCTTAGATTGCTAAAAGAAGCATGTGATAAGG
GAATTTAAACTCCCTCTCTCTCTTTTTTTATAATAAGAAACTATTGCGTTTGATACCAAATAA
AAAGTACATGCCAATCACTAAATTTCCAAAACCGTATAAGATTCTCGTATATTGCAAGTAGTT
TATAAGATTTGTTAGATTCCATCTCTAATAATTTAATAATGAATATCTCTCCACTTAACTACGA
ATTTGATTGGTCAAGCCAGAATCAGAAACTCCTCCTCTTTTTCTTTTTATAGCTGCACAAGA
GACAATCTTTCTTATTCGTGTGGTTCCTCTCTCAAGTCTCTAAAAC
>AtAMT2
TTGTCCTTGTCGCTATGTCCAGGGTCGTCTTCGTGGAAAAAGAGCCGACTGATGAAAGAT
GAAGAGGATCGGACGGTTAAGATTGGTCAGGAGAGTACTCTGGATCTGACTCTATGAATT
ATATTTAAGAATGAGACAAATTCTAAGTGAGATCTTGAGGTACTTACCGATATCTTAATATTC
AGTGTCGTTGTTGTTTATCATTAATCATGTATTTTCTTTATTATTAGAAAGGAAACAAAAATGT
TTGATTATATTAACGGTCTTGAGTTTGTTTGATACCGCCAATTTGTATCATCACATATATAAAC
AAATATTACTAGCCACATCATAATGATTCGATCTTTTGTCTTCTCATAGCAATATACTATATGA
AAATTACATATACATATAACAACAAATTTACATCGATTAGAGTATCTCTGCTGGTGAGCTTCA

TTCATTTGTAGATGAGACTTTTACGTAAATAGTACAAAAATTTTGGTTTCGTATTTATCCGAA
CAGTACATGGACAATTTTGTAGTTCCAAAACATGTTATAGTTAATAGTAGTGTTTTCAATAAT
TTCCAAAATCCATCTTCACTCATTTTTCCCTTTTCAGAGACATAGTGAAACAATTTTACACTA
TGCAAATCCGAAGCCAATTATTTTTGAGGTTTTTACTATATGTGAACAAGATTAATACTTAAA
CAACAAGACACACCTCGTTCTTTGTAAATATTAAAGAGGTTAAAGATCATAATACAAAAAAA
AACATAGAAAAAGTTCACAAATCATCAAATCTTTTGAGTTCATTATTCTATATAAAATTTTATC
TCGTGAAATGTTTATTGTATAAGGATGTGATTAGTTGCAAACCAAAAATTAATGTTTAATAGT
ATTTGATAGGGAACTTATTTCAACTGTGTAAAATTTTCCCGGTAAAATTTTCGTTCAATCAGT
CGATTCTAAGAACATGAATCTTATTGAATCTCTAATAATTTATTGTATAATATGGTGGCACCTA
TACGTCAATACGATAATGACGTCGTTAATAGTAACGTTGAGAGTTGAGACTGTTGTATAATT
TTGATTCGACGTGCCAAATACTACGTTCGACCAAGTATCAATTTTGATTTTCCAAAAAGAAA
AAACAAGAAACTTAGGATCTCCGTTAGTTTGATTGGATACACTGATACTGTTTCGATATTTA
GACAAACAAATGGAATCTTTGCTTCTTAAACATTCGATGCGTGTGTGTACCTGGTCAACTT
CTTCAGACTTCATTATCAAACATGGCCGCACGATCAGTTTTATTTTGCTACTGGAGAATTAA
CTTATCCACCTCAGAATCTAAACTAATTTCTGAAACTATATAAGTGTTTTTTGGGGCTAAAAA
TAAGATGCATGAAGTGAAAGTATCCTAGTGGTTAAAAAGGGGTGTAAACATAACGTTTAAT
GTTGTATCCCTTTATATGTACCTCTAATGTCCCTCTTTATTTACTTTTAATGATTTTTAAGACT
AAATATCTTCAGTTGAAGTAGGTAATCTTTTTGCCAAATTAGTCTTCGCAATACACGGATCG
ATTTCCTTATAAGATATTAAATTTAACTCATTCAAAAAATATTAAATTTGCAATGACTTATAAGA
TAAATAAAAACCAAGGTCCATATCTTATCCCAATTTGTTTTTTTTTTTTGTATACCAAATTTGT
CTCCTATATTGTTTTTATATGTTTATATTCTTTCTCCTAGTAATTTACACAAAGAATTTAAGAAG
CTTCATTGAAATATCAAGACTAGAATTGAAGAATGAGAAACATATCTATCAAACCTCGAAGA
CTTTTCTCCATCAAAATGTGCAAACAAAACACCAAACCCAACTTGTGTCTTGTCTATTCTAC
ACACATTATTATAATTAAAGGCTTTCACACCTACACAAAAAAAATTCGTACCATTGACTATAA
ACATCACAAACCCAACAAAAATTTCATCAACTCCGGGAAAGATAGAATAACAAA
>GmAMT4.2
TTTTTTTTAGTTTGGTTCATTTTTTGGATCTTGTAAAAACTGAACCCAATCAAATTGAACCG
AACATTATATATGGATTTTTACTTAATATATGTTTAGCATTATGTACTATAATTTTAAACTTTTGA
TGGTTTATTATGTATTTCTTTTTTGTAAGATTTGTAATATGTGGTTTTTGTTGTGGTATTAGTA
ATATACTATAATTTTAAACTTTTAATGGTTACAAACTTGCAATGTGATATATAATGTATTTCTTC
TTTTAAGATTTGTAATATGTTGTTTTTGCTGTGGTATTAATAATATGTAATTAAGTATTATTAAC
TCGTTATAGTTGAGATGTACGATTTACTTTTATGTTACAAAAAGATATGTCAATATTAATATGT
TCATTTTTTTAGTAATAATAGACATGCAATGGAACGAGTCAAAAATTAGTTTGGTTTTCTTTC
TACTAAACCATTGGTTTTAGTTCCAATACAAGTTTTTTTTTATATGTAAATATAAATGTTTTAGT
CCTTATTAACATCTTTTCCTTCTTCTTCCGTTTCTTTCTTTTCTTTTTCCTCACCAACGTCTC
CCTTCTCCTTTTATTTTGTGGGTTCCGTTTGTGTTTAAATCTTCTTACATAAACTAATCTTAA
ATATAGTCTTTCGAGGTTGCAAAGTTCAAACATGCATCTTGGACAAGACGCGGTAACTTGT
AAGCAACAATTTGTGTTGGCACTCTTTGCAATGACAGGACTTACCGCATGGTTTTAATCTT
CATGCATAAAACTTGTAAACATGAAAGTTGTCATTTGATTCATTTTCTGGATCACTTAATGCA
TCAGTCACGCACAACTGCCAAAAAAACAAAACACTAGAGCTAACATGGTAGAAGCATTTTT
TTTTTTCAATTTTTTTGCCAATTTAGCAATCGTTATTTTTGTGTGTGCTAGAGAACCTAGAGC
TAACATAGTTACAAGACCCAAAATACATTGTTTGACGCAACAAGATTAAAATGTTAAGAATG
ATGCAACAAGGTTTGATGCATTGTATATCTCCAAGCATTTAGTCATTCAACATCGTATACTG
AAGAGATTTATGCATCAAGATTAAAACAATGCAGTAAGTTACTGCTGTTGAAAAGAATGCCA

AACACAAATCATTACTCACGGTCTACTTCATCTTGTCTAAGAAGTGTACTTGACACAAACA
GAACCCGCAAAATGAAAGGAGGAAGGAGGTGTTGATATAAGGAAAAGGAAAAGAAGGAA
ATGAAAAGGAGAAAGGAAAAGAAGGGGGAGGAAGAAGTAAACGGGAGAGATTTTGATAG
GGACTAAACATTTGTTTTTTTTTTTTTCCTCAGGGACTAAAACATGTGTTTAAGTGTAGGAA
ATAAAACATAAAAATTGTGTCGTTGCGTCGTGTTGACTTAGCATCACTATTTTGCCTCATAC
ACAACATATTAGAGGATATATAACTCTACTATAGGTACCATTTTCTTTTTCTGACTGTTATTTG
TTTGACGACAATGGTTTTGCATACATGAATATATAAGCATTTACTGATCATTTACTATCACGA
AAAGCATACTATATATACTAATTACTAATTAATAGGTCTCGTGCTTGTAGATGGAAGGCTTTG
AAGGGCTCCGATGAAGGTGTTCACGACGAGGTAAATGCTAGATATGTTGTGGCCATAAAA
GGTCATTCATATGCCCTATCTAGGTGTTACACATAGAAGAGTAAACCTCCCATGACACTTAG
TTCTCCTACTTAGCTAGGTCACTCAAACAAAACAAAAAAACAAAGAGTGGGAAAAAAATGA
ATGTGCCATTACCCTCAAACCTAGCGGTTGTTGATGGAAGCCCTGAATGGTTTAGTAGAG
CAGACAATGCATGGAAGCTAATAGCTGCAACCCTAGTTGGTAACCCTAGTTGGTCTGCAA
AGTGTTCTAGGGCTCATAATCTTGTAAGGTGGAGCAGTGAAGAAGAAATGGGTAGTGAAC
TCAGCATTC
>GmAMT1.1
ACCCTCATCCCCTTCTTAATTATTTCATGGTCACTTGATCCAACAGCAACAATGGTGATGGT
GACGATGACAGTGGTGTCGTGACAGTGGAAGCAAAGTGCAATGGTGGTGGTGGAAGTAA
TGGTTAAATAGTAACGACGATGATGGTCGTGACAATAGTGTGATAATGGTAGTGGTAGAGG
TGGCAACAATAAGGTGTGGTGGTGATATAATTAGAATGTATTTTTTAAAACTAAAAACAAATT
AAAAAAATTTTGATTTTAATAATAGATGTAAAACTATTTTGAAATTGAATTAAAATTAATACAAC
TTCTTCAGCATGTTTTATTTTAAAAATTATATTCGAATCTACGCTTCAAAGGTTTCATGAAAC
TTTTTCCTGTTTTGTTTAAGTTTTGATTTTAGACATCAATAATCAATAATTTGTTACAACTGTC
ACATAAAAAAAAATATTAAGTTATCATAATAAATTAATAATTTTCAATAGAACGAGATCAAGAT
GTCATATTTGCATCAGGTTAGCAGTGCCCGTACGGCCATAGCTCCAAAACAAAAGATTCAA
ACCAGAAAATAATATTATAAAATTACGTCTCCCATCCCAAATCTCTTCATTGTTTTATTAGATT
GAAAACACAAATACAAAAGAGCAACAGAATGGTACTTAGCTTATATAATTTATTATTTTAATAA
ATATTACTAATAAATAATAAAATGTATGTTGAAAAAATATGTTACCACTATTTTTTAATTTGTTC
TTCATTATAAAACTTAGAAATTTTATTTATATTAACAAAGATATACTACTTAAAAAAAAATAATC
AAACTAGGAACTTGGAAGTAAACATTAGAAATAAGCGAATGAACTTAACTTTTATATTTGTAT
AATCATTACTTTCAGTTCTTATACTTAAAAATTACTTGTTTTAATCTATGTACATGTAGGAACC
AAAATGTGTTCAACATTGTATGTATATGAATTAAAATGAGTAATTTCTCATAAGAATTAAAATG
TATAAATTGTGCAGCTATAAACTCTAAAAAATGGGACTACACCTCAAAGAGAGATATTTCCC
TCATTATTGTTATAAATAACATTTGATATAGTTTTTTAAATAATTAGAAGAAAAAACAAAAATTA
AACAAGAGATATTGTCCTCTCATTAAAGGTATAGAAAAATTGGAGTGATTAAGCACAAGGAA
TAGAGGGGGGAAACAAGAAAAAATATGGTAAATAACTATTATTCTAGCGTGTTTGCTGTCC
AAAATAAATGTTCATTTTAAATAATTAAAATTTATAATCAATAAATACTTATAACTATAAAAATAT
ACTATAATTAAAAGTCTTAATAAATAAATATTTTTAACATATAAATTATATTTGAAATTTATGATAT
ATAATTTGTTTTGTGATATAATGTTGATTTTAGTTATTGATAATTTAAGTTAGAACGGAAGATA
TTGAAAATTATAGATTGAAAGAGATAAAAAAAAAAAGTTAAGAAGATAAAATTATAAAGATATT
CTAAATAAAAGTCTTTTAAAATAGTGTTCCCGTTAAAGAATAAATTTAAAAATATTCTTATCAA
AATATCTATTTTAGTTTATATAGAGAGAGGAACGTAATTAAAGTAGAATAAGTAGAAGATTAAT
TCAATGTATAGATATAATAATTACAAAAAAAAAAAAAAAACAAATACTTATAGAATAGAGTGG
GTAAAAAGTTAAAGAAAAATTAAAGAAACCGTGATTCCAACTTTTATATTCCACGAATGTGA

ACCTTTGAAAAGTCCACTTTCTTTCTCTCTCTCTCCTCCCCCACTTCGATAGCGCACACAA
TTTGACTTCCATAGTCCCCACTTTCTCAGTTTCTCTATATATACACACTTCACAGTTCACAC
TACCCTAATTAAACACCGCGCCCAACCCTTTTCCTCTCCCTTCTCCCACCTCAAACGCCG
TCGTTTCGACCACCTTCTTCGGTCGCGGCACAACCAATAACC
>GmAMT1.6
TTTCCCAGGGAACAAACCTATTAAATTTTAATTTAAAAACAATAACAAATATAAATTGTTACTA
TTCACAAGTTAAAATTGTCAAACTTAAATTAACCACATGATCAGGATTAAATTAAATTAATTTT
AAAGAAGATTAGATTGAACCAAAAACAATTATAGGACTAAATCGAACCAATTTTTAAAATTAG
AAAATCAAATCAAACATTAAAAAATAAATTAGAGAAAAAAACTAATTTAAAAAAAATGATCTC
GAAGCTTCTTTAGCACGAGTAACAATCATTAAAGATGATAGTAAGTTCGTCATACTGTTATT
TGGTTATTATCTGTTAAACTAAATTACATTTGAGCTGGATCTTTTAAGTTAATTAAACTATGAT
CCAGAATAAATAATAATTACATCTAGTTTGAATCTGGAGATCAACATGCTTAAACAACAATAA
TAATAAAAAAAAATAGGAAAAAATATCTCTTGTCCCTTTTTCAAAATATCGGAATATATATAAA
ATTTAACGGACATGGGGTTAATATTGGACTTGTAACTCTCCATGCACCTATCAGCTGGGAA
TTACGGGAGAAAGATTACTGCTGTCTCAGAACTGTGCTGTAAGATTATTGATAAGGATGGG
TGTATCTAGAGGTTCTTTTTAGTACCAGGCCATAAATATCTTTCACAACAACGAATATTGTTC
GACATAAATATACTTCTTATAGGTGGCAATTTCTTTAAATATTTAATATTTTAAAACTGTATATT
TAAAGTTTAAATTAATTTGAAACTATTAATGGAATTGAAAGCTTAAGGAGAAAGAATAGATTT
TGAAATGTAGATAAACCACCTTTTTTTACTAAATAAGACTTAAATATATTTTTTGTTTATGTAAT
TTAATATTTTTTTTTGTTCTTATAATTTTTTATTTTAATACTAAATTAAAAAAATAAAAAATATATT
TAAACCTTAAATAAAGATAATATGTTCAATTTTCTAAAAATTAACCATCCAAAGAATAATTAAC
ACTAATATACCGAGCATAATACTTATTCAATATATACAAAAATTAATTAAAAAAACACAAATATA
TAGATAGATAGATATTAGTTTAGCTAAAATGGTAGTAATGTAAGGTATATTTTTTTTTTAATTTG
TTAATTTAAAAGATTAAGATAATATTAATTATTTTTCAATTATATCCTTTTCTATCTGATTTTTAA
ATATTTTATATACTTATTGCACAAGGAGAGAAAGAATGGTGAAAATGAAGTTAAAAATAATTT
AAATACAAACAAAAATATTAGTTAATATGTTTTTAAATTGGATGTTATCTTTCAATATGGACAT
GCAGATGAAAAGAAACGAGAATGTTAAATTATTAACTATTCCCCTCAGGTACTATTTAAGAA
GTAAAAGTGTAAATCTTTTATTAGAAACATAATATTTACGCTGCCAACTCCATCAAAAGTTTT
AATACAAAAATTATTTATTGATTTCTTAACTAATATTCTCACCAAATTGTTTAAACATAGGTTT
GAGGTTTATGAGAGAAAAAGAAGGTTTTTTTGCTCTCTCTCTTTCAATTTAGAGAGTTATCT
GATATTTATAAAAACAAACAAAACAGCAAAACATTAAATTTATACAAATAGGGGAGTGACTG
AGCTAGAAAAAAAAAAGGAGAGGAAACGTGTCTAAATACGTAGGGCTGAGGAGAAGCCA
ACGAAAGAATCCCAAACTCAGCAGTGTGTAGTATATCAACACAATATTTGAGGAGGATCTA
TTCGCGCATATGATTTAAATTCGAAGGTTCCCTCTAAAGCTCTCAAAACCACTAACCTAACA
GCATGTGGCGCAATGACTTCCAATTCCCATCATACTCTTCTCTCTCTTGCTCCGCCAACCA
CCTTGCACCCCTCTTCAACGACACCGCCGCCGCAAACTACCTCTGCGCCCAATTCGATT
CCATTTCCAAGGGCCTCGCCGACACCACCTACTTTCTGTTTTCGCC
>GmAMT4.4
TCAGAATAACAATCAGATTAGGCATATTAATCAATATTTTGTTTATTATTATGTTAGTTCTAAGT
TTTGTTTGCTTTCTTATTTTTTTTTAAGAGCTTAGTATAATGTTATGCTTGTAACGATTAAAAGA
TGGGGAAGATGAAGATTAAATTATATTTTAAGTAAATAGTCATTTTTGTCCTTGAATGTTTAG
AGCGTCGACAAATTTGTTCCTGAATGTGTCACATAAGTTATTTCATTAATGACAACGGACAA
AAATTAATGGATTGATGGTTTTGTTGTATTTTTTTTTCATTTTTGGAAATTAAAATTTTAATTTT
TGTATTTCGGGAACGAATTTATCAATATTCTAAACATTCAGAGAGCAAAATGATTATTTATTC

TAAAAAATTAGAGTTATAACTTATAACGGTTAATAAAAAAAGTTAATTTAAACAAACTTGAAG
GTGTGAATCGTTGAAAACAAAAAAGTTAATCAAAATAAAAGTCAATCTAAATTCTTTCAAAA
AAATTGTTAATCTAAACAAACTTGAAGGTGTGGCGTGTTCAAAATAACGTATGTATGTGTAA
ATCTCGTGTTCTTGAGAGCTTTGTGCACCTCGCAAATAATTCTTTGAGAATCGGTGATAGT
GGTTTGTGATTTTTTCTTTTTTCTTTTTTTATGAATTGGCCAGCATGCAAGAGTCTTTAATTC
CTATCTAGACGCAGCAAGAAATTAGTACCTACGAACTAGTAACAAAAGTAATTCCATAGCTA
ATTCATAATTTTCATAACTAATTAATGTGCAATTTTTTCGTAGTCAATCTCGTAAAGGTCTCTT
ACTAGTCTTCATGAAGAAAATCAGGCTGAAAATTGCGGAGATGGTGAGATTAAAGAAAGA
CGGGGATTTCAATACAAGATGCAGTTTCTAAAATGTTCTTTTGTTGTGATATTATCTAATTAA
TAAGTTTTACTTTAAGAATCTATACTAATGCAAATTTTTATACAAAGATTCCTGTAATAAAGCT
TCATATTTAAAGCAGAATAATATTAAAAAAATTAGGGAACTATGTATAAATTTTGTCCCAAATC
ATTTTGTTTTTAATCCCGTTTAGCCTCAAGCGTTAATAAATTATTACTGGGCCAATTCTGTCA
TAGTGTACGTTCTGATTATTCCTACCAGCCACAAAAAACATGCGCGAATACAACATTGCAG
CAATGCAGCCCCTTGCTCTCTAGTTCTAGCTAAAATTTTGAAATGGACGTGGAAACCAGCT
AGTTATCGTCTCATTAATAATATACTTTAACCAGATTTTTTTTGCTTTAAGTATGTCATATGTAA
CAACTACGTGACCCACTTCCATTAATAATAATAAATAATTTTTTTTGGGGGGGGGGGGGGG
GCAATACAATAATAATTAACCTACGAGATTTTGTCCATCATGACATCAATTATTTATTTATTTAA
TTAACCTCCAGAAAACTAGATAAAATTCGAACACGAAGAGTTGAAAGTTGAAACGAAATCA
TACATGAAAGCGATTAGAACTAAGCCCTTAACGTCACAAGTGAATTGCTTATATCCCCCGT
CTCAATTGCAAATTATTAAATATAAAGAGAAATGCTGGGAATATTTTTCAGATTTAGTTTTTTA
ATACACTTTTTTATTCGTTGAAATTCATTAAAATTTTATATATAGATCTTACATTATATTAAAAAA
GTGTGTCTAAAAATATGTACGCAATATGAAGTAAAACTTTAACCTGAAACTTCTTTGAAGAA
GTGGCTTAAGATTCTCGAGTTTAGCTTCTATATGACAGAATTTTCAAATGAATCTAATTGCG
TCGACAGCATAGAATGTCGAATCACCGCAACAAGAAACCACACCTTAGCGATAATTCCAAA
GATTCCTTAACAAAATGTCCAAAAATGTGTCACTCTTTAAATTCCAAACTCCACCACACCAT
CCAGAAAATTTTCGAAATTCTCGAGTGACATTAATTTGGTTTCATTATTATTACT
>GmAMT4.5
ACAAAACATAATTTAAAATATTATTTCTTATTTTAAATTTTTTAAATGAAATTAGGTGTATTTTTA
TACAATATATATTTATATCTTAAATACTTTTACCATATAAGATATTATTACACCTTGTCCCATAAA
ATAGCAAAACCAACTCTAAGTGTTTTCCTCTGTGTGTGCAAGGGAGAGAGAGAGCATAGA
CAATTCAGCCTTGGCATGGCAACATGACACATGGACAGCTTTGGCTCTCTGCGTTCTTATT
TCCACAAAACACTTTTTTTTTTGTCGGCCAAAGAAAATATATTGATATAGGCACAAGAGATA
CCTAAACCCATATACAAGAGGCTATTTGTTATTTTTACCATCCTTACAATAAAAATAGTTAATA
ACCCCAAATACAAAAACATATCCATCCTTAGCTACCAAATCTACCCTAACAACATTATAATGT
TCCTGTATCTACCCACAAATACCAGCTGCCAAAACAGGACACATGACCCATCCTCTACACA
TGGTTGGTTTTGGTGTAGGTGGGACAATGATAACTTAAGCCCAATCATGTTTATGAGTTTTT
AAAGGTTTGGAAAAATCCTTGTAAAACTCGTCTCATATTTTTAGTTAAATATTTAATTTCATTA
TTTATTTAATTATTGTTATAATTTAAATTATTTAATTATTAAAATAGTTAATTTGTATTTTAACAAT
TATAAGAAGTGAAAATACAAAAAAAAAAATCTATATAGAGTAAGAGAGAGAGATAATGTGGG
GTGGGTTACATCTCTTCCCCTGCCTCCGCTTTTACCAATGGAGAACAAAATGCAAACCAG
ATTCTTGAGTGGACTGTTGCTTCCCTGCACCTTCAATATTGCTTCTTGCACTCCATTGTATT
TCAAAAAGTTTAATACAAAATGTAAAATTACATTTTGAATTGAGCTTTTCAAATGCAATGGAG
GTGGAGGAAGTTTTACCCCAAACTCCTCTGTTCCAGTTAAAGTTGATTTTCTCTTTCAAAG
TCGAGGCCCAATTGAATTGTATGGTATCTACCAATTTCAGATTTAATTATCGTATCAACCAAT

TTTAAAAGTGACATACGTACAATAGAAAAAAATATTAGTGTATTTGTTGGGTTGGTTGGATA
GTGTTCATATGGGACAAAGTTAATAACACATCAGATATAACAAGTTTATGAAATATATTCAATT
AAAAAAATGCTTATACGGTTATACCTTTTCCTTTTGCGTATATTGTTACAATTCATTATCTAAC
GTTAAAAACCTGACATAAAAGGTGGCATCGTCATATATAATTAATTAAGTCTATTTTGATTTTC
CTCTATTTTTATATTCACATTTCAAAATTATATCTTAAGACTAATTAAATAAATATATCTGTTATA
TCCCATTTTTTAACGACGTTAGGTAAGTTTTAAAAAAATATGGGATACTACAGACCATTTTTT
GGATATTGTGAGGAGTGTCACTCTCTTGTTCCTTGTTTAGCATTTAAAATATACGTGATTGA
TGTTAATGTACACTACTTTATGGTCCCCTCAGACTTGCCTTTTGGTTAGATTAGACAAGCCC
AAGTCATGATCAGATTATAAATATAAGTGAGCACAATAATGTTAGCATCAATGGCGAAACTC
CTTGTTTAAATTTAAATATTCACAATACAAGAAGCAAGTACAACTCATGATCAGATTATTATAG
TTTAAATGTTACAACCAATGGCTTGGTTAAAAGATTAATTCACATTACAAGAATCGACGGGC
TTTGTTAGGCCTTGGATTCTCCGTTCTCAAATACGCCAACCTTGTCAATCTCAAATTCCAAT
CATATAATATAGAATATAGAGCTAGCGACACAAATACATATTAATACACTATTGTTTGCCAAAT
ACTATATAATCGCTCTCTGTTGTTTATTCTGGATCCTTAAACCTCCACTTGAAGCTACGTGG
TTATATATTCTGGGAATACATGCATATATAGAACAGTATATATATCAGAA
>GmAMT1.2
ATTAAATTAAAAATTGAATTTAATTTATTGTTAACGTTTTTATTGTTGTAAAAACTTATTATATG
GATATCATTACTAAAAAATTCACTTATCTCTCGTGGTGGCTACAACGAAATCTGGTAACTAC
ATAAAAGGCATCATGAAACATTTCCTTTTTACTTTTATGTTTAACTTTTGATTTTAGACATCAA
GAATCATTAATCATTAATTTATTACAACTATCAAATCAAAATAATCATATTAGTTTATCATAATAA
TTTTAAATAATCAATGGAACGAGATCTAGATGTCATATTCGCATCAGGCTTAGCAGTGCCCA
AACGATCATAGCTCCAAAACAAAAAGATTCAAACCACCAAATACTAATATAAAATTACGTCT
CCCATCCCAAGTCTCCTCATTGTTTTATTACATTGAAACACAAATACAGAAGAATTATAGAAT
GGTACTTATATAATTTATGGTTTTAATAAATAATAAAAAGAAAGTCAGCTGAAAAAACATGTTA
CCACAATACCATTATTTTTAAGACTTTTTAAATATATTCTTCATCATAAAACTTAGAAATTTTCT
TTATATCAACATAAAGATTTGTTTTTCAGGAAATACATAAAGATAGACTATATTTTTTGAAATA
GGAACTGAATTTTAGAAATTCATTTAATTCCTATACTTATAATCTTTACATTTTAGTTTCCATAA
TTCAAAATTACTCATTTTAGTCTCTATTATACATTTCTTAATTTGTTTTATTCCCTATAATTTTG
AACAACATAATTAAAAGCCTGAAATGGATTAAAAATGTATGTATATATAGGAAATAAGACAAG
TAAAAAAAAAATATACATATAAACTGAAATGAGTAGTTTCCAAACATAAAAATTAAAGTGTAA
CGATTGTTTCTGTTATAAAGAAAAAATAAAAATACTAAGAAACTATAGTTTAATTGATTGAGC
AGACCGGATCATGTATCGTATTGTTATTTATAAAATAAATAATTAAACCAACAAAAAAGGAAA
TAATACTTTGTTAGTTACCAAATCTTTTTTTTACTGCTTTACCAAAACAGTTATATTTTAATGA
GTTTTTATTTTGTGAATTTATTCTAATGAAAATTTAACAGAACGGGAAGAGAGGAAAATATAG
ATAGAATAAAAATATGATGAAAATATAATCAGTAATATATTGTTAAAGGGTAAGATAGGTAAAA
AAAATATAGATCCAAGATGATAGAAATAATTAAACCAACAAAAAACGGAAGTAAAACTTGTTA
GTTACCAAATCAGTTATATTCTAAAATTTAAAACAATGGAAATAAAAATGAATATAACTGAATA
AAAAGATGATAAAAAAAAATCAATTATAATGATTAAGATTTATGATAAACAATTTATAATGATAA
AATGTTATTGATAATTTTTTTATAAAACTATTGAAATTCAAGTTAAAATTAAAGATATAAAAAAG
AATAGATCTAAAGAGATAATGAATCATGAATATTTTTTAAAGAGATTTTTGTTGGGAAAAAAA
TCCTTTAAAAATATTTTCATTGAAGAATAAGAAAAAAAATATTTTTTCTCTCAAAATAGCTATT
CTCAGAAATAGCTATTCTGATATTATTTAATATTTACAGAGGGATGTAACTAAAAAAGACAGA
AAGAAAATAGAAAAGAAATTCAATGTATAGAATCAATAGATATACTAATTACTAAAACAAATAC
TTATAAATTAAAGTAGGTTAAAAGATAAAGAAAAATTATAATTATAATTAAGATTCCATTCTTTT

CCACCTTTGAAAAGTCCATTTTCTCGTTCTCTCTCTCTCTCTTCCCCCAGTCCGATAGCGC
ACACAATTTGATTTCCATAGTCCCCACTTTCTCTGTTTCTCTATATATACACACACTTCACAG
TTCACACTTCCCTAATTAGCCACCACTCCCAACCCCCACCCGTAGTTTCTACCACCTTCAG
TCACGGCGTAATACACTAACCAACCCACC
>GmAMT1.5
TATTGAAACATAACAAATATACATAAATAACAAAAAGTAAATTTTAAGTAATAAATTAATAATAA
TTTATTTTTTTAGATATGATAAATATTTTAAAGTTTTCTTTTAAAGTAGATTTATGTTTTTTTCAG
TTAATAGAAGCTAACTGATATATATAGAAAATCGGTAGTCTTTGACCCAAAATTATGATAAATT
ATTGATTTTTATAAAATTATCTTAAAAATCATAAAATTAATAATTTATAATTAAATGATAATATATA
TATAAAACGATTTTATATGATCATTTTGAGTCTCAGGGTTAAACTCTTGTTATTTTTGTTCATA
ATATATATACATGATGGGAGTTTGAATTTGTTTAATATTATGATATTGCACTATTTCGATTGGG
GTAATTATCCTGAAATCCTAATTTTTCCTTAACCAAAATAAATTAATAATAATTACATCTAGTGT
GAATATGGAGATCAACCTGCTTAAATATCGGAATATATATAAAATTTAAGGGACATGAGGTTA
ATATTCGGCTAGTAAGTCTCCATGCACCTATCACGCCCAATCCTTATTGGATTGGACTTGG
TGCGTGTCGCAACGGAAACGAAGACGAGGTCCCAAGGTCAGAACCTGCTCTGATACCAT
GTTAGATTTCATGTTAAAACCAATTGGCATTAAGTGAAGTTGCCCAACAGATATATAAGCCG
TACTCCAAAGATTGAGACAGTCGATGTGGGACTTTGGTTATTTCCCAATAGATTATTGCTGT
CTCAGAACTGTGATATGAAATTGTTGAGAGAATATTAATAAGGATGGGTGTATCTAGAAGCT
TTGTTTAGTACGAGGCGGCAAATATCTTTCACCAGAATAATATTGTTCGACTTAAAATATCTT
TATTACAGGTGACAATTTTTTTAAATATTTAATCTCAAACTGTATATTTAAAGTTTAAATTTAAA
ACTATTAGTTAAATTGAAAGCTTAATTAAGGAGAGAGTTTTAGAAGAAATAAAGTATAATGCT
ATAAGGAGAAAGAATGGATTTTGAAATGTAGGTAAAACACTTTTTATTATTACACAAACATAT
AGTGTGTTCAGTTTTCTAAAAATGAACCATCCAAAGCTTAATTAACATTAATATACTGAACAT
ATAATACTTATTTGATATATATAACAATTAATAACATATATATATATATATATATATATATATATA
TATATATATATATATATATATATATATATATATATTAGTTTAGCTAAAATGGTTGTAATGTAAGGTAT
ATTCTCTATCTGTTTATTTATTTATTTTACTTATCCACTTTTAATTTTTAATTTAAAAGACTAAGA
TAATATTAATTATTTTTCAATTATATCCTTTTCGTATCTTACATATATATTACTATTTAAGAAATAA
AAGTGAAAATGTTTCATAAGAAACATAATATTTACGCAGCCAACTCCATGAAAAAATTTGATA
CAAAATTTATTTATTGATTTGTTAACTAATGTACTCACCATAATGTTTAAAAATATATATGTTTG
ACGTTTAGAGAGTTATCTGATATTTATAAAAACAAACAAAGCAGCGAAACATTGAATTTATAA
TATTTTTTATTTTAAAAAATAAAAAATTATCATCCATTGAAATATATATAATTTCACTGTCAAAG
ACTAAGAAAGGCAAAGAAGAAGAGTTGTAGCAGAGTGAGTGAGCCAGAAAAAAGGAGAG
GAAAGATATCTAAATGCGTAGGGTTGAGGAGGAGCCAACGAATCCCGAACTCAATCTGTA
GGATTCCAACACAATATTTGAGGAGGATTTATTCGCGCATGTGATTTATAATTGAAAGATTC
CCTCTGCAGCTCTCAAAACCACTAACCCTAATAGCTGAACTCCAAAGAGATAAAATAACAG
CAAGTGGCCCTCATCAATTCTAAGTATATATTAAATCATTGCCGCCATCACTTCTTCTCCATA
CTCTTGTCTCACTCTAACAGCCAAAGC
>GmAMT1.3
TTTTGTGCTTTGCCTTCTCTGTTCTGTTGTTTTAACTCTTGGAATTTTAAGATGAAGGGTAG
AAAAGGATGAAGGTGGCCTAAAGGTGTAGTTGGGAACGGTAACTTAGTTCGTGTGATGAG
AGAGAAGAGAGAATTAGGGTCAAATGGGAAAATAATGAGAAAAAAAAAAACGTGACTTTTAT
TTATTTTTTTAATTTTATTAGTTAATAAAATATAATAATTAAAATTTTAAAATAAATTAAATAATAAAT
AATAATATAACTTCAACTTAATTGATAATATGATATTTTGTTTGTTACATACTAACTTAAATTATA
TTGAAACATAACAAATATATATAAATAACAAAAAGTAAATTTTAAGTAATAAATTAAAAATAATT

TATTTGTTTAGATATGATAAATATCTTTATGTTTTTTTTAAGTAGATTTATGTTTTTTTCAGTTAA
TAGAAGCTAACTGATATATATAGAAAATCGGTAGTCTTTGACCCAAAATTATGATAAATTATTT
ATTTTTATAAAATTATCTTAAAAATCATAAAATTAATAATTTTTAATTAAATGATAGTATATATATA
AAACGATTTTATATGATCATTGTAAGTCTCACGGTTAAACTCTTGTTATTTTTGTTCATAATAT
ATATACATGATCGGAGTTTGAATTTGTTTAATATTATGATATTGCACTATTTCGATTGGGGTAA
TTATCCTGAAATCCTAATTTTTCCTTAACCAAAATAAATTAATAATAATTACATCTAGTGTGAA
TATGGAGATCAACCTGCTTAAATATCGGAATATATATAAAATTTAAGGGACATGGGGTTAATA
TTCGGCTAGTAAGTCTCCATGCACCTATCAACATGGCTTTGGCTTGTTAAGGGGTCTCAAT
TTTGCGGCACGAAATTGTGACAGGATAGCATGCCAGTTAAAGAGCAAACAAGAAAGTAAA
TCCGAATGAACAGATGTGACAAAAGAATGGCTAGGAATTACGGGAAGGATGGGTGTATCT
AGAGGTTCTTTTTAGTACGAGGCGGCAAATATCTTTCACCAGAATAATATTGTTCGACTTAA
AATATCTTTATTACAGGTGACAATTTTTTTAAATATTTAATCTCAAACTGTATATTTAAAGTTTA
AATTTAAAATTATTAGTTAAATTGAAAGCTTAATTAAGGAGAAAGTTTTAGAAGAAATAAAGT
ATAATGCTATAAGGAGAAAGAATGGATTTTGAAATGTAGGTAAAACACTTTTTATTATTACAC
AAACATATAGTGTGTTCAGTTTTCTAAACATCAACCATCCAAAGCTTAATTAACATTAATATA
CTGAACATATAATACTTATTTGATATATATAACAATTAATAACACACACATAATATTTACTATTTA
AGAAATAAAAGTGAAAATGTTTCATAAGAAACATAATATTTACGCAGCCAACTCCATGAAAA
AATTTGATACAAAATTTATTTATTGATTTGTTAACTAATGTACTCACCATAATGTTTAAAAATAT
ATATGTTTGACGTTTAGAGAGTTATCTGATATTTATAAAAACAAACAAAGCAGCGAAACATTA
AATTTATAATATTTTTTATTTTAAAAAATAAAAAATTATCATCCATTGAAACATATATAATTTCAC
TGTCAAATTAAGACTAAGAAAGGCAAAGAAGAAGAGTTGTAGCGGAGTGAGTGAGCCAG
AAAAAAAGGAGAGGAAAGATATCTAAATACGTAGGGTTGAGGAGGAGCCAACGAATCCCG
AACTCAATCTGTAGGATTCCAACACAATATTTGAGGAGGATTTATTCGCGCATGTGATTTAT
AATTGAAAGATTCCCTCTGCAGCTCTCAAAACCACTAACCCTAATAGCTGAACTCCAAAGA
GATAAAATAACAGCAAGTGGCCCTCATCAATTCTAAGTATATATATAAAATCATTGCCGCCAT
CACTTCTTCTCCATACTCTTGTCTCACTCTAACAGCCAAAGC
>GmAMT1.4
TTTTTCTGATGCTTATATTTGTTTACTTATAAAGATTTCATTTACCATTTTAAGGGAATCTCGT
TTCTTTTTCTTATACTCTTACTGGGTTCACAATTATGTTTCAATTCTTATGGTATCTTTCTTCT
TTTTTGTTTCTGTGTGTTGTGGAGGTGGGATGCTCTTTTCTAAATTGTTAAATTCTCTTTCT
TCATTATTAATTCTATGCAAGGTAAATTATCTTTAATTTGTTACATAACAGATTACATGAAGCC
TGATACAGAGACATACAATTGGGTTATTCAAGCTTATACTAGAGCTGAATCTTATGACAGGT
TAGACATTTAAATAGCTGTGAAACCTCCCATTGGAATTCTTAAATTGATTATTTCATGGATTG
AGTTAAATTTGCTATTAGGTGATGTGATTGTATTAAACTTGTTTGTGTGTGGCTTATAGAGTA
CAAGATGTTGCTGTGTTACTTGGCATGATGGTTGAGGATCACAAACGTATACAGCCAAATG
CGAAGACCCATGCGTAAGACACGTATTAATTTTCTTTTACAAACCTGGCTAACATGTTGGAT
GTAAAATTGTTTAAACTTAGGTTTAAATACCTTTTTAGTCCTTGCAATTTAGTGTTTTTTTTTT
CTTTTTCATCCTTACAAAATTGTTTTTTTGTTTTTAGTTCTTGCAAATTATGTTTGTTTTGTTT
TTAGTCCTTAAAACACTTTAGATAATGTTTTAAACAGTAAAAAAATGTTTTCTAAAGCTTTATA
AGGACAAAAAATGAAACAAGCATAATTTGCAAGGATTAAGAAAGATTATTGTTGTCTCAAAA
CTGTAATATAAAATTGTTGAGAGAATATTAATAAGGATGGGTGTATCTAGAAGTTCTTTTTAG
TACGAGGCGGCAAATATCTTTCACCAGAATAATATTGTTCGACTTAAAATATATTTATTACAG
GTGACAATTTCTTTAAATATTTAATCTCAAACTGTATATTTAAAGTTTAAATTTAAAACTATTAG
TTAAATTGAAAGCTTAATTGAGGAGAAAGTTTTAGAAGAAATAAAGTATAATGCTATAAGGA

GAAAGAATGGATTTTGAAATGTAGGTAAAACACTTTTTATTATTACACAAACATATAGTGTGT
TCAGTTTTCTAAAAATGAACCATCCAAAGCTTAATTAACATTAATATACTGAACATATAATACT
TATTTGATATATATAACAATTAATAACATATATATATATATATATATATTAGTTTAGCTAAAATGGTT
GTAATGTAAGGTATATTCTCTATCTGTTTATTTATTTATTTTACTTATCCACTTTTAATTTTTAAT
TTAAAAGACTAAGATAATATTAATTATTTTTCAATTATATCCTTTTCGTATCTTACATATATATTA
CTATTTAAGAAATAAAAGTGAAAATGTTTCATAAGAAACATAATATTTACGCAGCCAACTCCA
TGAAAAAATTTGATACAAAATTTATTTATTGATTTGTTAACTAATGTACTCACCATAATGTTTA
AAAATATATATGTTTGACGTTTAGAGAGTTATCTGATATTTATAAAAACAAACAAAGCAGCGA
AACATTAAATTTATAATATTTTTTATTTTAAAAAATAAAAAATTATCATCCATTGAAACATATATA
ATTTCACTGTCAAAGACTAAGAAAGGCAAAGAAGAAGAGTTGTAGCAGAGTGAGTGAGCC
AGAAAAAAGGAGAGGAAAGATATCTAAATGCGTAGGGTTGAGGAGGAGCCAACGAATCC
CGAACTCAATCTGTAGGATTCCAACACAATATTTGAGGAGGATTTATTCGCGCGATTTATAA
TTGAAAGATTCCCTCTGCAGCTCTCAAAACCACTAACCCTAATAGCTGAACTCCAAAGAGA
TAAAATAACAGCAAGTGGCCCTCATCAATTCTAAGTATATATATAAAATCATTGCCGCCATCA
CTTCTTCTCCATACTCTTGTCTCACTCTAACAGCCAAAGC
>GmAMT4.6
TTTTATTAAAAAAATTAGTTAAAAAATTTGAACCGATTATCCCTTAACTTTTTTCATTTTCTCA
AATACTGAATCAATCTTTTACAAGAATATATATATATATAAGAAATGAGTCGCCTACCATCAAA
CCTGTTGCCAGTTGATGGAAGCCCGGAGTGGATGAACAGGGGTGACAACACGTGGCAG
CTGACGTCAGCTACTCTGGTGGGCCTGCAGAGCGTTCCGGGCTTGGTCATCCTCTACGG
CAGCATAGTGAAGAAGAAATTAATGGGCTTTGAACTCTGCCTTCATGGCCCTGTACGCTTT
TGCGGCGGTTCTTGTTTTCTGGGTGGGGTGGTGCTACAAAATGTCATTCGGCGAGAGGA
TCCTTCCATTTCTGGGGCAGCCAAACACGGCCTTGGACAGGGAATTCGTTTTTCGCCAA
GCTTTTGCAGGGGAATTTCCCAACGCCACCATGGTGTACTTTCAGTTGGCTTTTGCTGCC
ATCAGGTTGATTCTCATGGCAGGGGCATAGCTTGCAGGTAGGGATGGTAATAAGGCGAGT
GCCTTTTGAGAAAGTCTTTGGTTCAACCTATTTAAGACTTGACTTAAGGTTAAAGTCAAACT
ATAAAAAAAGCTTGTTAAGCTTGATAAGTCAACTTATTTAAGTAATAATAATAATAATATTATTA
TTATTATTTATACAATTTTTATGACATTATGAATGACAAGGTGAAGTGGCATAAAGTGCTTAA
GTGACAAAAAAATTTTAAAAAGAAAGAGTCGAGTTAAAAGGATAAAGCAAACAGATTAATAC
TTTCAAAGAAAAAAAAATGTTTTGTAAAGACATTCAGACAATTTAAATATTTTTATTTAACTATA
TTAGTATAAATCATCTCTAATCCATATATTTTTTTAATATTAGGTTCTTTTTTTCATTTTCTTTTC
ATATACATTATGTTTTAACAACTTAAATTCAATATGATTTTGTTTATCAATTATTTTTGAATTTAT
ACATTACTTATACGAAATTTTATAAGTTTCTTTTTTTAGTTAGTATTTTACTAGGTTTTAAAAAA
TTAATTGATCAAAAACATCTTTAAATATACTTTCAAATAGGCTCATATGTCAAGCCAAACTTTT
ATATAGGTTATCTTTAAGAAAAGTCTTAAGAAAAAAATGCTTATTACAATGAGCAAGTTTAAG
CTCTTGCATATTCAACTTAAGCCTTCAGTATATGGAGAACTGATGGACGGTTGTTCAAGTT
GGGCCTCATTGATTACTCCGGTGGCTTTGTTATTCATCTTTCTTCTGGGGTTGCTGGTTTC
GTCGCAGCATGTTGGCTATATAGTTTCTAATTATTTTGCATTATCTTTCATGCATATGATCTC
CCTAATCAGTGTAATTTACTTTATTTTTTGAGAATACATGCAAGTATAGGGTGTTTGAAAATC
ACATGGATGCACTTATATGATATAGAGTTTTTCTTTTTTATCAATAAATATTAGTTTATTAATTT
TATTAAAAATGTCAATGAAGAAATTATAATCCTACAAAACTATTCACCGATACAGATTTGTTA
GGAATGAAAACTTTGTCACTTTTAATAATTCTACCGGGTTTAATGAGTAGTTGCACTCTCTT
ATTTTTTAAAATTTATTAAATTTGTAAATAAAATTTTATATTTTCATATGATTTTATTTTTTATAAT
TTATCCTTACTGATTTAGGGTTTTTATCCGCCACAGTCAAATAGGGATTACTTGTTGCAGAT

GTAAAAAAAGGGTGTTTTGAAGAGTTGTACCAAATTTATACAAGTTTAATTGGCATGAAAAT
ATACTATAATTCTTATTTTGGCTTACTTTAATAATATTTTTGAAAAATAAGTTCATTCGTGATAA
AATTTATTACGTAAGTTTGGTGGTAACACATGGTGAATGAAGTGGGGATATGACTTTTTATG
CAGGTAGGACCAAGGGCAGTGAAGGATAGAGAGAGGTTCCCTCCAAACAACATACTTCT
G
>GmAMT2.3
GAAATAAATTTATTTTTTTACGAAACTACACCCTCAAGTTGCCAATTGTCCTGATTTAACAGA
TGTAGTTATGTACGGAATAATAATAAATAAATAAATACTACGTAATAAAACTATGGATAACAGA
AGTACAGATATAATTTCATTTTCAGCTACTTATTTGTACGGAAGAAACCAAGAAATGAATGAT
GGTCACACTGTCATGTAATATATCCGGTGCCTTAGTCAAGCAGATATAAAATATGGTATCTTT
ATAATAAAACCATAAAAATATTTCCACTTTTACTTAGACCCACCAATCAGAGCAACCTTCCC
CGTTGAACTCTTGAAGTCTTTTATAATCGCCAGCACACTATTCTACAACCATGCCCATCATT
TTTTTTTATACTTCTTTTTTGCAAATGGGGCACAACATCATTATCAGCTAAAATTCAAAGTTT
TATTTATAATTTCCCCAACTTGTGAAAAATCAATGATAATTCACAGAAAGCTTCAAAAGATCC
AAACTAAGTATCTTAACTAACTTCCAACAGCTTTTATGTTTGAATAGAGAGTGTTCAGTCTA
ATGTCAAATTAAAATTACTGACTTATCTTTTAAATTACTAAAATTAATTTGAAATCTGAGTTTC
TCATAAAAAAATATTTTTTTATATATACATTGATATGAAAATCAAATCCTCATTACATAATTAAGA
CATATAGTTATCTTTCAAACATAAGATCTCATATTCATAATTTTCAACTCCTATACATAGAAGA
CAGAAATAAAAGTTTACCAACCCACCAAAGCAAAAAGACACCTAGACATAAGGACTCACAA
ACTATAGTCTCATAAAAATAGGGCTGAACACTGTTTGAATGCTTAAGCAAAATCTAACTGAA
ACGAAATAAATCATGATATAAAAACAGTTTGATTAACTGATATATTTTTATAAAAGTGGATTTG
TTCAATTTTTTGAAAACGGATCATTTTTTTTTATTGGATTTAGTTTAGTTCAATTTTTTTTATTT
CATAATCATATATGATTCAACATTTTTAAAGAATCAAACCGAGCAAACCTGAACCACTTTTTT
AAAAACAATTTCGTTAATCAAACAATTTTTATAAAATTGCTTGATTTTGTAGATCGATTTATTT
TTTTATTTTTTATTTTTACACCCTTGCATAAAAAGATCATAGGCTTGTGAGTTCAAACTAATTT
AGCACCAATACCGAAATTGGTCTTACCATGGCAAGAAGTTTCTTATGCATGGAGCCAAAGT
CCATGGCTTCCTTCATAAATATTATTTTCATCTTTAAATATAAGTCTCTTAATTTATATTTTTTAA
GAAAATTAATTAATTTAGTTCATAACACTAAATGAGAAAATGCATTTATCTTTTTAATTCATAG
CATTTATTGTAAAGATATTTTTAAAAAGTCTTATGAAAAAAATAAACAAATTTAAAAAAATAATC
TCATAAGAACAAAGGTAAATACAATTTAGTAAACTTTAATTATCCCACCTCTTCGCTCATTAT
GATGATAATGAAATTCTGCAAGAGTCAAGATGTATTTACCACTACTTACAATATATCTTTTAAT
TTTCCATCTATTTTTTTAGAAGACTTTCCATCTACTTATATTAATATTTAAAAATTTGGATTGGT
TTTCTAAATAAAGAATTTCGACTAATTAACTTATGTAAAACTTATACATAAGTTAATTTTAATTT
TAAACATATTTATTTTTTTATATTTTTCTCCATTAAATTCTTATAAAAAAAAATTCACTACTTTTC
AACAAATCCATGTCTCAGATAAGATATCATATTCATACTTTTCAACTCCTAAAGACAAAGACC
GGAAGACATGCAAGTGTATACCAACCATCAAAGCAAAAGATATCAAGACACAAAGATTCAC
AAACTCCAGTTTCATAAAATGATCATATATATACACTAGTGAGTCCAAAGCTTGATTACAAAA
CTAAGATCTTCCAATTAAGAACTTGTGCTACCC
>GmAMT2.1
TTAAAAAAAAACAAATTTACGAAAAAAAAGATTAACAAATTAAAAATAGGCTGCAAATAGTAA
AATAACGTTTAAAAAAAAAGGAAGATTACGACTTCAAAGTCCTATGCCTCAAAAAAAAAATT
TAAAAAAAATAAGACAATTTAACGACTTTAAAGTTGTAAAACAAAATACGACTTTGGTTTTTT
TCTTTAAAAAAAATATAACTTTAAAGTCGTAAAAAAAATTATATTTACACTGAAGTCGTAAATA
TTTTTACGACTTCAGTTAAAAAATAAAAAATTAAAAAGTCGTAACAGGTGTTACGACTTCTC

ACTTCAGAAGTCGTAACAATTGTTATGACTTATCCCATTTTTGGTAATTTTTTAAAATATACC
CCCAAATAGAAAATTGTGAATTTTTTTATCCCTATTGGTAAAAAAGCCTAACTTTGGTCTCC
CGTTAATCTTAATTCAAGAATTTAGTCTTTTTATTTTTTTTCCTCACCATTTCAATCTCTCTAT
TTTATAAAATTTGTAATTTTTATTCAATTTTTAATTTCACGAATAATTATTTTTTATTTTTATATTT
AATTAGCTAGATTATTTTTTAGAGTATCTTAAAAAATAAATATATACAAAATTAAGAATTTGAAA
TTACAAAATTTTAAAATTAGAAGATCAAAATTACAGAAAAATTGGAAGATCAAGACTATAATT
TAGCTTCTTTTTTTATCATTCATCCTTCATGGAGTATTGCCTGACAGGAGAAGGACCATGAA
TTTTGGGACTCGTTTAGTTCTGTTTTCACTTTTAATTTATAAATTTACTACTTTGTTTTCATTC
TATTCTCTGTTTTCATATATTTATACAAAAAAAAAACATTAAAAACAAAATTCTTTTTTTTCTCT
TTTTTTTTGTTTTGAAAACTTTGAAAATAATATTTTTTTTTTTGAAGACATGGTCATCGTTTTG
TAAATTAAAAGTGAAAATAGAACCTTTCTTGCACAGCAGCTTCATGAGAAAGAAGTTGTAA
AAACAAAAGTAAACATACCCTTATTTTTTTCTACCCTCTTCCATAATTTTTCTCTCATTCATTA
TAAATGGTTAATCAAGTAATATCATAGCAAAAAAGATATTTGGACAAAGTATACACCAAAAGT
GTGATAAAAAATAATTGATTAATATGATTGATAATGTATAGCTAGAAAAGAAAGATAGAAAAAT
TATGAGTTTAAATAAAGTGAGTCATTTACTTGAATGAGTAAAAGTTTATTTCAATAAAATTACT
ATACATTGATTATTGTAATTAAATTGTCAGTGAAAATTTTCTTACACATTAAGTACTATTTTAAT
TAAATTCTAATTAATGCTTTGTAACAACCATCAATAAGAAACTAATTTCTTTAAAGTTTCATTT
TCCAATTTTCATCTACTCCAGTGTGTCTTGAGACCCACCAATCAAAGTACTCCCCATCCAT
TCACACCACGTGGTGGCAATTAACATTCTCCGGTCCTCTTGCATTCCAAAACCACACCCA
TCATTTTCAACTAAAAGTCAAAGTTGGCCTACGAAACTTTGACTTTTCCTTATAACTTTAGT
GAGTTGCTGCAGCCAGTTACGAAAAACCAACGACAGTTTACACGTAGCTTCCAAAGATTC
AATCAAACCGAGTATCCGAGTTTTCAAATTACTATGAAGCTATATATATACTCATACCATCGC
CCCCCAAAAAAAAACAGTAAAATTGCCACAAACAATTCATCAGCTCATACACGTAATTTCTT
TTCCTCTTTTCCTCTTATCCAATTCTAATCACGATCAGACATTAAATGTAAACACTTCTCTAT
CAAAAATTTGAACTTAGTTCGCCTCACACTTTTGTTTTGTCACCTTGTGAGAGACTAATTC
CCTCTAATAAACGCAACGTTGTTCATCAGTGGCACATACATATACAGCATCACAATTCTTTG
AAGGGTGAAAAAGCTTGATCAAGAATTGAAGCATATTGATCTTCAGCC
>GmAMT4.1
TGCATAGTACAAGTATAAAAATCGCATGATGCAATGGTCACTTACCCTAGTGGGTAATATATT
TCTTTATCATTAAAATAATGTAGGGGAAATGGTACGTGAAAAAACACCTATGATTAGAGTGA
ATGTATGCTGTTTTAATTATATTCTAGTACAAAGAAGATTCTGAATATATATTACAATAGCCTT
CTTGTTACTGAAAGACAGTTGCGTACTAAGACACCAACGAGCTTTGTTGATCCCGTTGGC
AAAAAATGTTTGTAATACCCGCCTAAGTTTAATCATTAATATTATGAGCGACATGGCCTAGAA
CAATTTAAGAGTTGTTTTTTGACAGAAAGAACAATTTAAGAGTTATGACAACACTTCTACAG
CTTGTCGTGAGAATCAAAACAAGGTCCTACAAATTAAATTCATATAAGGTACCACCACTAAC
CCTTGATCTAATAATTATTCTATGTATATATATAGCTTCATCTATTAGTCTTAAAAACACTCAAT
CCATTATTCACTCAAACAAAACAAAACAATAAAAAAAAAGTGGAAAAAATGTCTGGCGTGC
CATTACCCTCAAACCTTCTGCCTGGTGATGGAAGCCCTGAATGGTTTAGCAGAGCAGACA
ATGCATGGCAGCTAATAGCTGCAACCCTAGTTGGTCTCCAAAGTGTTCCAGGGCTCATAAT
CTTGTACGGTGGAGCAGTGAAGAAGAAATGGGCAGTGAACTCAGCATTCATGTCACTCTA
TGCTTTTGCATGTGTGTTTTTCTGTTGGGTTGTGTGGGGATACAGAATGTCTTTTGGTGAT
GAGCTTCTTCCTTTCTGGGGAAAGCCTGCTATGTCACTTGAACATACTTACCTTTTCAAGA
GGGCCTTTTTGGGGGCATTCCCAAATGCCACAATGGTGTATTTCCAGTGTGTCTTTGCTG
CCATCACTTTGATTCTCATTGCTGGGGCTGTGTTGGGGCGCATGAACTTCTATGCTTGGA

TGTTGTTTGTGCCTTTGTGGCTCACTTTCTCATACACCTTCACAGCTTTCAGCATATGGAG
TTCTAGTGGGTTTTTGACCAAAATGGGGATAATAGACTACTCAGGTGGATATGTGATCCATT
TGTCTTCCGGGGTTGCAGGATTCACGGCTGCATATTGGGTAAGCAATAAGATTTTTGTATG
CTTTGATTAATTCGTCCAATATATTTTTAGGCTAGGCTTAATTAATTCCTTTTACTATGATTAAT
ATTTAGTTTATTGTGTCTTTTCTTTGTCACAATCTTCACCTATTTAACCTTTTGTTTGTTCTTA
GAATGCATGTACAACACAAATAAAGTTAAAACCAAAGTTGGAAACACTAACGTTGCAAAAC
ACTCCATTGAGACTCAAAGTTTTTCTCTATTTTTTTTCCTTAGGTCATATCACTTAACATATTT
ACACTACTTCTATATATATTTTCTATTTGTTTCTCTCTCTCTGAGTGTTTATTACTAGCATATG
GATGTTCAAGAATCACTTTTCTTAAAAAATAATTGCACATTCTTTCACTTAATTTTTCTTCTAT
CTCACTTTCATCCCATTTTTCTTCTTGATTATCTTTTCATCACTTGCTACATTCATCACTTTTC
TCTCTCTGTTTTTTTTTTCTTACTATATATTTTTTCCCTCCCCCATACCTTCGCATAGCGAAG
AGCTCTGGACAATGGGTACGAAGTTTTTTATATTTTTTCCCTCCATCTCAAAATAGGGGTAG
TCATCATTTACAATTTTTCAAAATAAAATTCATGGACACCCATCACTAGATGATATCTAGAGA
GAGAAAGAGAAAAAATATATGGTGTGTACAGTATTATACATAAGTGTCTATATATGATGACTC
TAAGCATGCACCAAGCAATCAAAGTAACATAGAAAAAGAATAACGATGAGTGCATACACAT
GTAAATGATTTGACAGGTTGGGCCACGGCTAAACAAGGACAGGGAAAGGTTCCCTCCAA
ACAACATTCTTCTG
>GmAMT3.1
ATCGCTGAAATGCTCCTTCAAGATCATTGTAGAGATTAATGGGGGAATCTTAATTAAATTAAT
CCTTGAAAAACGCAGTGAGATGGAATTACAATATTTTTTCCTAATAAAATAGTGTTCAGTGT
CAGTTCACAAAAAAATTGTATTGCGTGACATATATATATATATATATATACATTTCTCTTCTTTG
ACAAAGCAGGATATCTTTAAGTTTAAGATCGGATTAACATCTGGCTGATTCATGACTTTGAC
AAATTAATAAGCCCTTAATAATAATAAACGATCGATGATTCAGGTTAGCTGGCAGTTAACGTT
AATTGCTGCTGAGGTTTTGCTTATTAAAACACCAAACTACAGATAACTTATATAAACGACAA
CTAATTATACTATTATATACATGCTAATCGCGCAATTACAGATAATTAAAAACACAGCTTCCAA
TCTCTATTATCATCATCATGTCATCTTCGTGCATGAGAGAATAATGAGGGTTGAGTCTAGTC
AAAAGTGTTATTCCTTTGTAATAGTTGCTTTTGCATCATTTTTTTTATATGATTTTATGTGAAA
CCAAACACGCATCCAATGATAAACAATGTTGGCTGCAAAATTCTATAATATTTATGTTGTCTT
TGTTTTAGCCGTTAGTTGCCTTATACTGTTGCCCTATGTTTTAATGGGTTATCAGTGTGAAT
TGATATGATTGGGATAGCCTAATTTATATTCAGAACCTGTGCATTGGTTTTGATCTGATTGGT
ATATAGCTTAATGTCATGGTCTAAATTCATTCAGGATACGTACTTACAAAATACAGAATATTG
CATATAATATTTAGAAAGTGAGAATCAAAGACAAAATAACTAAGTAACTACATAATAAAATGTA
TACATTTTTATGAGAAAAATATGCTTTTTTAATTAATGTAAAAATGTTTAATTTTAATTTCATTTT
ACATAATTTTAAAAAACATTAAAATAATTTTATTATTTTTATTAATTAATATATTTAACTGTATCCT
ATTAGATGTCAATATCATCACGATGTTCATATAGTTAAATATGTAATATCAACATTAATTATTAA
TAAAGATTATTTTCAACAAACTTAAAATTTACAACAATAAAAATCAATAAATTTTAAAGAAATT
AGAATTAAATTTAACTATATTTATTAGAATTTAAAAACATATTTAAGTTCCTTTAAAAAATAAATT
TATGTCTTTATATTATTTAGAATTATAAGATTAATTGATGGTAAAATAAAAAAAATTATGAATTC
GATTTTTCAATCTAAAAGAAACTAATAATTAATGCAACAATTAATACACGACTAGTAGTAGTG
GGTCTGGTTCATGTGCCAAGCTGGCTAATACGACAACCCGCAGCGCTCTAATTCGTGCCA
CTATCCTCGTAATCGTATGCCTCCAATTGTCAGCTTTGGAACCTAAGAAGGAATCTATCTAT
ATCCTTGTGACATTTGGGCCAGATGCCCCACCAGATTATCATTTTTCTTAATATAATATATAC
GATTCTAAATTATTCTCATAATTTAAAATGTAGTAAAACAGTTAACTTATAATATAAATGTTGTT
CACAAATTTATATATGTCAGTATTGTTTTTATAATTTAAAATTTCTAAACCCACCAATTATATAG

TATTCTCATTAGACACTTAAGGAATATATATATTTTTTATAGCTTAATGAAGTGCATAAGCTCA
CTTGTCCTATAATATTATTATCCTCAATATTAGAAACTTAATTAAGGAAAATAAAGAAAGAATA
TAAATGTGCAAGAATCCTGGGATGTCAACGTGGAGGATCACCTTTTACTTAGACAAGTAGA
TGGAATCAGTAGGGAGATGCATCTTCTCTTATATCTTAAAAAATACGTAATTATTATTAGGAG
CCAGGCGTACACTTGAAGTATGAATAAAAAGACACACCAAAGATACCATTGTTAAAAGTTT
GGGCAGCCCTAGGCACAAAAGAAAGTGGTGATTG
>GmAMT4.3
TATATATATATATATATATATATATATATATATATATATATATATATATATATATATATATATATGCTTCA
CAAGGGGTTGATACGTACTGAGTGGAACTAAAGAATTGATCTGCAATATATTAATGGTGGT
TGTTAAAAGCTTCCCAACAACTACAATGTAAAGAGATTTGCCTGCAAAAATTAAACTATGAC
ACTCGGAGTTAGATATCAAAAGGTGACGGGCATCAGAGTTTAAATTCATATATCTCATAGTG
TTGAATCCGAAATTTATTTAAGTTGGTTGACGTATCATATAACAGTAAATGCAATTGACTGTT
TTGAAAATATTTTTAGCATGTCAATTGAATAAAGTGACTGAATTATCATAAATCCTCTAACATA
ATTTAAACAATTGCACTATTATTTGTCACGTATTGAATATTTCTGGAAGGTGTTGTCCGCTCA
CTCTCGAACGTTAGGTCAATTGAAACTTGTATTAATTCCATTCTTGCATACCACAATTAGAT
CGAATGTAGTCTAAAACAATTTTTGACATCGTATATTATTTTATCTATCCCTTTTTTCATTTAAT
TATATAAACAACACCAAAACATTCTATAAATTAAATGTGACCACTAAAGTTGTCAAATAAGTT
GTACCAAAAATAATTATATTATATTTTTAATATATCAGTAAAACATATTTCAAATTATTATGGAAA
ATACGAAGATAGGTTTTGCTGTGCAATAAATGTGGTTTATCTGAATGCATTTATAAAAAAAGT
TATTAAATTTTTATAATTTAATAAGGATTAATTATAATTAATCATAATAATTCTCATAAAATGTTAC
AAACATACTCTCTAACACACTTTTATGGACATATTCTTTTATTGGTTGAAATCGATTGAAAAA
CAAAACTTTGTCCTACATTCTATTTAATAATTTTTTCTCTTAATTTTTTAATTTTCAATAAAATT
TAACCAATAAAAAATATTCTATTTGTAAGAGTGTGTTAAGATGTGTGAATTTAGTACTTCTCA
ACTTATAATATATAGCAAAATAGTACCTTGCTACAGTTTAGTAATATTTTTTAATTAAATATTTAT
TAAAAATGTTACTAATGATAACATTTAAAAATATTATCAAATATGAATAAATATTATTAATATGTT
TTTATAATATTTTATTAAATGTTTGCATAGTTCATATAACATTTGATAAAAGATTCGTGATATATT
AATAATATTTATTTATATTTGATAATATTTTTAAATATTTACATTTTTAATTTAATAAGTGTTAGAA
AAAAAAATATTACTAAAGATTACCTGTGTGTTGTGTTGTACTATACCAATAATACAAAATCAA
GCACAATTAAAGCTGACAAATACACCACCAATGAATAGTCGTTATTTATGATCTTGAAAACA
GAAAGCAATACAAACTTGCATTGTAGGCTCGAAGAAGGCCGCACAAGCTTACAATATTGTC
TACTCGCTCGCAGAAAGAGTACGTACAAGGGAATTTACATTGACCTTAATTTAAAGAGATG
AGTAAAAACTTGAACTTACAACATGTTAATCGGAGTATTTAGTACAATATTTATTTCAAAATG
GAATGAGAAGCATATTAGTTTATTAGGGAATATATGAAGCAAAATCAAATGGAGCGCGTCTA
CTTGAACAGAGTTCACGTCATAAATAGATCGTACTCTACACCGCGACATACATACGATGAAT
CAATGAATTCAGAAAATTCATTCTTCCACCTACGTCTGCCAACAAAGCCGGCCTCATAATG
TTCAATCCAAATGAATAAAACGACAAATCGATGTTATTATATATGTCGTATATCCCCCCAAAA
ATTATTACTTGGATTCTATAAACCTGCATGCAAATAAATAACTCGAGTGTTAGCAAGCCTTG
GCCTTGAACACCCACAACCAAACCTAAGCAGCAACTGAAGTACTAGTCATATATCCAACAA
TCAAGAATAAATATAAAGGAGCGAACGATCG
>GmAMT2.2
AATTTTATTTTTTTTATTTTTACTCCCATAATTTTGGTTTTCTTATTTTAGAAAATTTGTAATCTT
GATGAAATCCTCAATTTTACATACATTTATTCTTTTATTTTTTATATTGTAATTAACTAAAACATT
TTTTTAATATCTTAAGTGAATAGGTTAGGTTTAGAGTTTAATTGTACCAAAATAAAAAAAAATG
TATGAAAATGAAAAATTGAGCAAAATTGTGAATTTTTTAAAATAAAAAAAAATAAAACAACCA

AAATTATAATTTAGTCTTTTTTAACACTCATTGTTCATGGAGTATTACCAAACAAGAGAAGGA
CCGTGAATTTTAGGACTCATTTACTTCGGTTATTCACTTCTAATTTATAAATTTTATTTTCATG
TATTTGTACAAAAACATTAAAAACAACATTTTTTTCTCTATTTCCTAGAAAACTTTGAAAATAA
AAAATAGATTAAAAATAAAGTGATAATTTTTTTTAATTAAAAGTGAAAGAAGAAAATGAAATAT
AAAATAAAAACAAAAGTAAGCATACTCTTATATTTTTCTATTCCTCCTCCATCATTCCTCTTTC
ATTCATTACAAATGGTTAATCAAAAAATTATTTGGACACTAAAAAAATACTACTCCTTCCCAT
ATCTTTTGATATTTTAGTTTTCTATTTTTTTTCTTCAAAACAATTGATATTTTAGGATTTGAAGA
TCTAGTTAATATATTTTTCTCAAATATATCCTTATTTAATGCACACTACCAGTGATATAAGTATC
AAATACTCTTTCTCTCTCAAAATAAGTGTTATATAGATGAAAGAGGATAATAGATTTATAGAAT
TAACTTTATTAATTAATGAAAGAAAGTAATATAGATATTAAATTCAAAAACTAAAGTGAACATT
TATTAAGGATATTTATGGAAAAGAATAATTAATGTTACATTTAAAAGTCAAATGTAACACACTT
TATTTTGGAATGAAAGGGATATCAAAGTATTAAATAAGGATATTTTAATTTAGATATAATAATTT
TTAGCTTTATTAAATATTTCTTGATCTATGTACAATAACAAACATAAAATAAATACAAAAATGA
GATATTGTATACCTAAAGAAAGATAAGAGATAATAGATAAATAATTAATACAAATGTATAACAA
GAAAAGAAAAAAAAAGAAATTATGAGTGTCAATGGAGTGTGTTATTTTATTTAGGTGTTTAA
AAGTTCTCACTCATTATCATAAAGTCAATAAATTCCCTATATATTCATTATATATATTGATGATT
TGTAATTAAATATCAAAAAATTTAAACATGGAGTACTATTTTAATTAATGCTCTTTAACAACTAT
TAATAAGAAACTAATTTCTTTTAAGTTTAATTTTCCAATTTTCATCTACTCCACCGTGTCTTTA
GACCCACCCCAATCAAAGTACTTCCCTATCCATTCACACCACGTGGTGGTAATTACATTCC
CCAGTCCTCATGAATTCCAAAACCGTACCCATCATTTTCAACTAAAAGTCAAAGTTGGCCT
AAGAAACTTTTACTTTTCCTTTTAACTTTAGTGAGTTGCTGCAGCCTGGAAAGCCAACGAC
AGTTTACACGTAGCTTCCAAAGATTCAATCAAACCGAGTATCATCCGAGTTTTCAAATTACC
ATGAAGCTCCATATACGCATACCATCCCACAAAAACACAAAGTAAAATTGCCACAAACAATT
CACTAGCACATACACATACCCTCTTTCTCATATCCTATTCAAGTCACCATCAGATATATTAAA
TGTAAACACTTGTCCATCAAGGATTCGAACTTCATTAGTTCTCAAACTTTATTTTATCTCATC
GTGAGGGACTAATTCCTTCTTATAGACGCAACTTTCATCGGCCACAAATGCACATGTATAAT
TAATTTCATATATGTATATATAGCATCAGAGAGAGAACAATTCTTTGAAGGGTGAAAAACCTT
GATCAAGAATTGAAGCATTAATCTTCAACC
